# Electrochemical Activation of Bicyclo[1.1.0]butanes

**DOI:** 10.1021/acs.orglett.5c05325

**Published:** 2026-02-13

**Authors:** Jeremy T. Maddigan-Wyatt, Daniil A. Knyazev, Daniel B. Werz

**Affiliations:** Institute of Organic Chemistry, Albert-Ludwigs-Universität Freiburg, 79104 Freiburg, Germany

## Abstract

Herein, we describe a novel, light, and catalyst-free electrochemical method for the activation of bicyclo[1.1.0]­butanes (BCBs) enabling cycloaddition with aldehydes and arylation with arenes. This exceptionally mild strategy for BCB functionalization leverages preliminary anodic oxidation in concert with strain release to facilitate the formation of oxabicyclohexanes (oBCHs) and bis-arylated cyclobutane products. A plausible electrolytic mechanism is proposed, alongside insights garnered from mechanistic studies.

## Graphical abstract

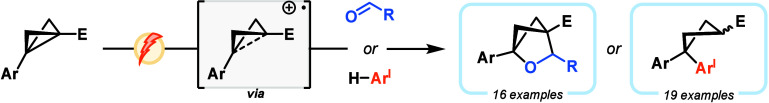

Strained cyclic compounds have enabled a wide range of varied transformations in organic synthesis.^1,2^ Since the first accounts over 100 years ago of bicyclo[1.1.0]­butane (BCB) synthesis, their chemistry has intrigued chemists.^3^ Typified by less commonly encountered bond geometries, high π character of the central bond, and strain release energy of ∼64 kcal mol^–1^, this molecular framework enables a wide array of diverse functional handles allowing the generation of valuable sp^3^-rich architectures.^2^ In the ever-expanding realm of sp^2^-omitting pharmacaphores and drugs, exploiting these saturated alternatives to unsaturated compounds has shown increased drug activity, enhancing solubility and stability through their three-dimensional structures.^4–6^ Canonically, their diverse reactivity stems from the inherent ring strain, coupled with the various reaction modes through which this distinct C_3_ synthon may be activated.^7^ Methods employing Lewis acid catalysts,^8^ nucleophilic and radical attack,^9,10^ reduction^11^ or oxidation,^12^ and energy transfer^13^ have been well-studied, establishing a diverse array of reactive manifolds for the synthesis of sp^3^-rich cyclic products (Figure 1A).

**Figure 1:**
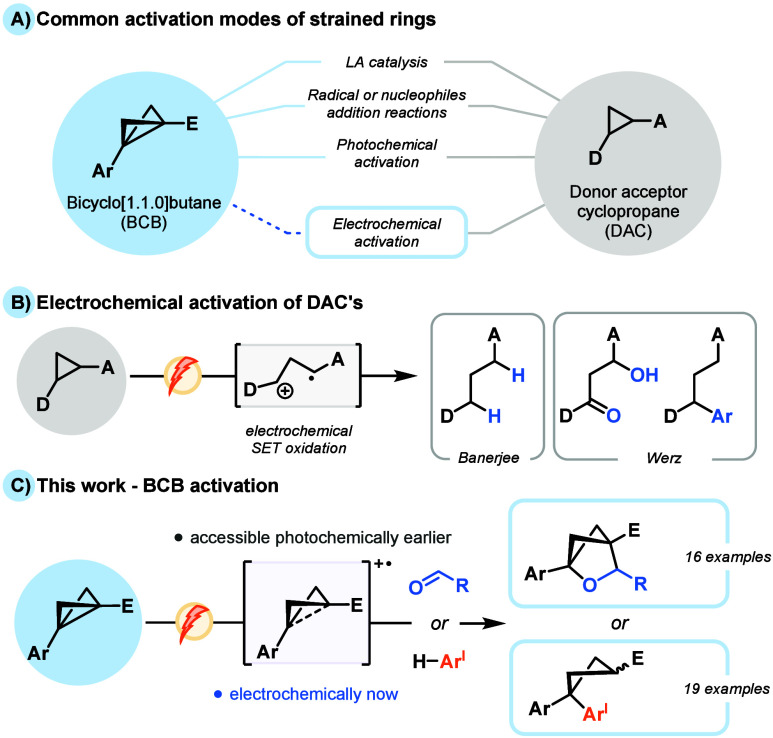
(A) Common activation modes of strained systems. (B) Electrochemical activation of D–A cyclopropanes. (C) Electrochemical oxidative activation of BCB substrates.

Clear similarities in the reactivity of donor–acceptor (D–A) cyclopropanes and BCBs abound, often proceeding through analogous ring-opening strategies.^14^ Of specific interest to us is the preliminary oxidative single-electron transfer (SET) of D–A cyclopropanes, which has been used in the delivery of an array of functionalization strategies (Figure 1B).^15^ Electrochemistry, though a classical field,^16^ has recently re-emerged as an enabling technology supplanting the need for oxidative photocatalysts, necessitating the assessment of substrate photostability or harsh settings, instead facilitating SET under mild conditions directly at the electrode bilayer.^17^ Notable, recent examples by Maji and Hari detail unsubstituted bicyclo[1.1.0]­butane spirocyclization cascades, wherein electrolysis facilitates radical generation.^18^

Within the rich landscape of BCB photochemical oxidation,^19^ we sought to expand upon this mechanistic ideal using electrochemistry in concert with aryl-substituted bicyclo[1.1.0]­butanes; a reactive method as yet unexplored. Specifically, we sought to leverage the oxidative capacity of these substrates to affect the formation of radical cationic intermediates (Figure 1C). Herein, these highly reactive intermediates engage in cycloaddition or arylative transformations for the synthesis of a diverse array of oxabicyclo[2.1.1]­hexanes (oBCH) and quaternary benzhydrylic cyclobutanes.

To this end, our investigation into the electrolytic annulation began through coupling of ester **1** and benzaldehyde **2** at the glassy carbon (GC) anode and cathode with 1.2 equiv of TBABF_4_ as the supporting electrolyte in dichloromethane at room temperature. These conditions yielded **3a** in 30% yield, which could be increased to 41% by the use of analogous salt TBAPF_6_ (Table 1, entry 1). A myriad of alternate electrolytes were examined (see Tables SI-1 and SI-2 of the Supporting Information), with inorganic salt LiBF_4_ performing modestly with the addition of solubilizing acetamides as the co-solvent (Table 1, entries 2 and 3). A systematic solvent screen with LiBF_4_ as the electrolyte (and DMA co-solvent; see Table SI-1) uncovered THF as the optimal solvent for this annulation, delivering **3a** in 66% yield (Table 1, entry 4). The product was formed in 39% yield with dual graphite electrodes, with examination of alternate electrodes delivering yields lower than those with GC (Table 1, entry 5, and Table SI-2). Notably, although Lewis-acid-mediated/reductive manifolds are implicated when using sacrificial anodes, these pathways are precluded through the use of solely carbonaceous electrodes. Sequential variation of electron delivery (amperage) and stoichiometry (F/mol) failed to increase the product yield, proving reactively deleterious (Table 1, entries 6–8). Following this, a suite of commonly employed electrochemical mediators was examined (Table SI-2) with CoTPP forming **3a** in moderately enhanced yields under exceedingly mild conditions (Table 1, entry 9). Finally, no oBCH **3a** was detected when the current or electrolyte was omitted from the reaction conditions. Notably, polarity inversion of the electrodes at 1 minute intervals successfully avoided electrode passivation, while altering the timed intervals, electrolytic pulsing, or without polarity inversion delivered **3a** in lower than optimal yields.

**Table 1:**
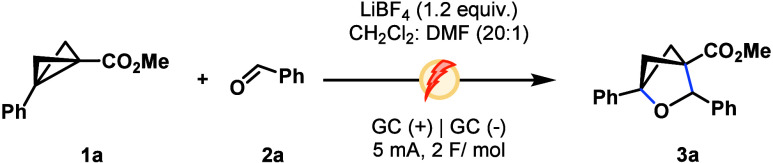
BCB Annulation Optimization^a^

entry	deviation from conditions	yield (%)^b^
1	TBABF_4_/TBAPF_6_	30/41
2	none [LiBF_4_ and CH_2_Cl_2_/DMA (20:1)]	43
3	LiBF_4_ and CH_2_Cl_2_/DMA (7:1)	57
4	THF/DMA (20:1), 10 mA	66 (61)
5	graphite (+)|(−) and THF/DMA (20:1)	39
6	5 mA/2 mA	65/66
7	5 mA/1 mA with 1 F/mol	42/39
8	5 mA with 10 F/mol	1
9	CoTPP (2.5 mol %), GC (+)|(−), and 2.5 mA with 1.1 F/mol	68
10	no current/elecrolyte	n.d.

a Reaction conditions: **1a** (0.10 mmol), benzaldehyde (0.50 mmol), electrolyte (0.11–0.13 mmol), solvent volume (single solvent = 4 mL and mixture = 4.2 mL), glassy carbon (GC) anode and cathode, constant current = 5 mA, and 2 F/mol.

b Yields were determined by analysis of the crude residue with CH_2_Br_2_ as the internal standard. Isolated yields are represented in parentheses.

While conditions described in entry 4 (Table 1) delivered methyl ester oBCH **3a** optimally, we found these unsuitable for the generalization of substrate scope. Dichloromethane, which has been used to great effect as a reductive sink for oxidative cascades^20^ performed more reliably on a 0.1 mmol scale of **1**. Specific conditions of the solvent system and electrolyte are noted for individual compounds and outlined in Scheme 1 and the Supporting Information. Additionally, DMF was chosen as the co-solvent over DMA due to equivalent reaction outcomes, with these conditions finely balancing the formation of the desired products **3** over cyclobutene and alcohol addition byproducts, alongside minimizing decomposition of **1** at higher current densities.

**Scheme 1:**
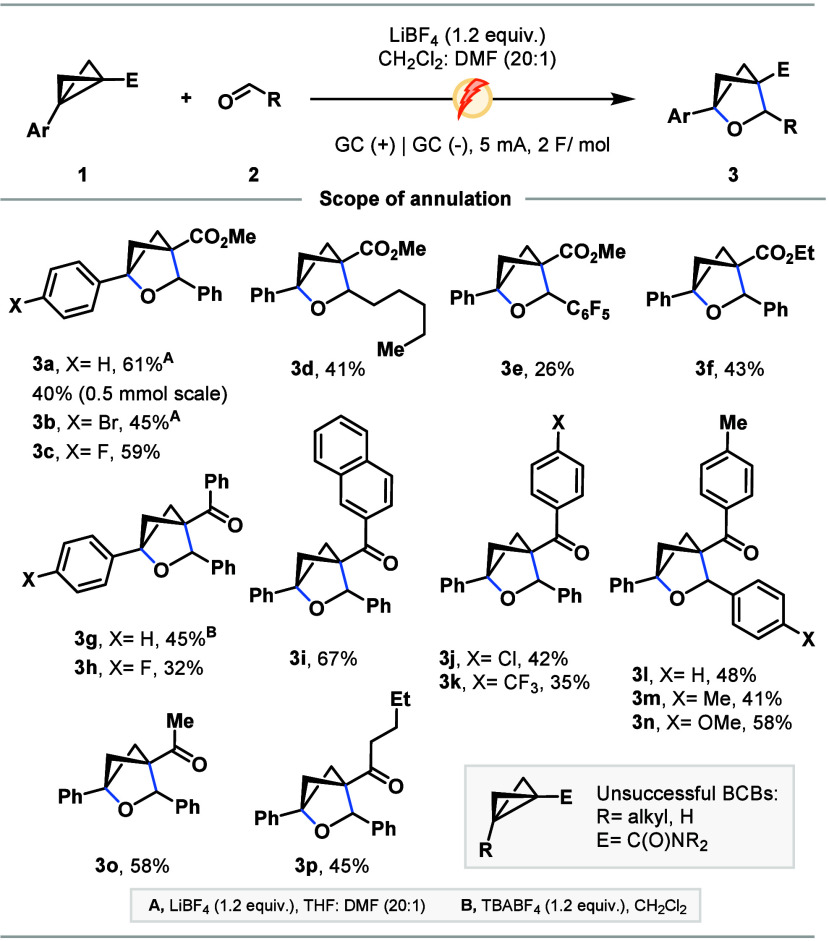
Scope of BCB Cycloaddition with Aldehydes^a^ ^,^ ^b^

a Reaction conditions: **1** (0.10 mmol), **2** (0.50 mmol), LiBF_4_ (0.12 mmol), CH_2_Cl_2_/DMF (20:1, 4.2 mL total), glassy carbon (GC) anode and cathode, constant current = 5 mA, and 2 F/mol.
b Isolated yields are represented.

With our attention turned to reaction generality, alternate aryl substitution appended to BCB substrates was examined, with both *para*-bromo and fluoro delivering **3b** and **3c** in 45 and 59% yields, respectively (Scheme 1). Alternative aldehydes were amenable to annulation with esters, delivering **3d** and **3e** in 41 and 26% yields from hexanal and pentafluorobenzaldehyde. Ethyl ester **3f** was furnished in 43% yield. Variation of the electron-withdrawing moiety of BCB substrates to ketones was next examined, where **3g** and **3h** were generated in 45 and 32% yields, respectively. Pleasingly, naphthyl ketone **3i** was amenable to reaction, yielding 67% of the desired product. *para*-Substitution about the electron-withdrawing aryl moiety of **1** was well-tolerated, with electron-poor (**3j** and **3k**) and moderately electron-rich (**3l**) being well-tolerated in 35–58% yields. Further variation of the aldehyde coupling partner uncovered that *p*-tolyl and *p*-methoxy benzaldehyde furnished **3m** and **3n** in 41 and 58% yields, respectively. Finally, examination of alkyl ketone BCBs enabled the synthesis of **3o** and **3p** in 58 and 45% yields, respectively. The reaction of BCBs **1** bearing an alkyl or protic substitution was unsuccessful. Similarly, BCBs appended with amides were unsuitable for cycloaddition. This methodology represents a marked increase in reaction efficiency compared to recently reported LA-catalyzed cycloadditions of BCBs and aldehydes.^21^

The generation of highly reactive radical cation intermediates inspired us to examine alternate capture modes. Arylation of bicyclo[1.1.0]­butanes is underexplored, and as such, we aimed to broaden this synthetic utility through the addition of arenes via Friedel–Crafts-type reactions. Pleasingly, we found methyl ester BCB **1a** could be coupled effectively with an excess of anisole in HFIP, delivering **5a** in 73% yield as a 5:1 mixture of *p*/*o*-arylated products, with a 3.6:1 diastereomeric ratio of the *para*-substituted compound (Scheme 2). A detailed optimization for the synthesis of products **5** over cyclobutene or HFIP addition products is appended in Table SI-4 of the Supporting Information. Notably, in light of recent reports of BCB activation by HFIP,^22^ we confirmed that the bis-arylated product (**5a**) can be formed without a current, however in a significantly reduced yield (Table SI-4). Other arenes were also tested in current-free conditions showing only trace product (**5m** and **5u**) or no product formation (**5s**), indicating that highly activated arenes may react through non-electrochemical means but highlighting the necessity of the current for less activated substrates. For this reason, we limited our investigation to *N*,*N*-dimethylaniline and dimethoxybenzene as the most reactive substrates, since increasing arene nucleophilicity was expected to enhance the contribution of the HFIP-mediated pathway (Table SI-4).

**Scheme 2:**
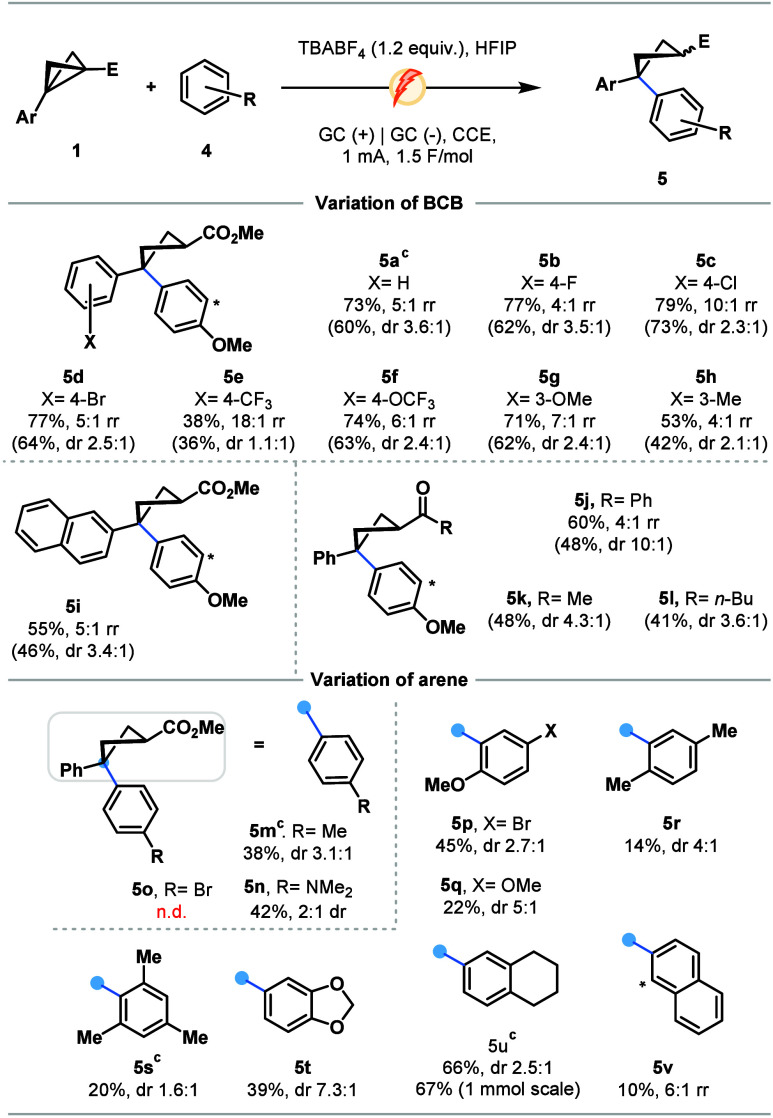
Scope of BCB Arylation^a^ ^,^ ^b^

a Reaction conditions: **1** (0.10 mmol), **4** (1.00 mmol), TBABF_4_ (0.12 mmol), HFIP, glassy carbon (GC) anode and cathode, constant current = 1 mA, and 1.5 F/mol.
b Yields of regioisomeric mixtures are reported, and isolated yields of the major regioisomer are represented in parentheses.
c Reactivity in the absence of a current: **5a**, 32%; **5m**, trace; **5s**, n.d.; and **5u**, trace.

First, we examined the effect of substitution about arene of **1** on arylative coupling with anisole. The reaction proceeded smoothly using a variety of weakly and strongly electron-withdrawing *p*-substituted aryl moieties, delivering **5b**–**5f** in moderate to high yields, with the highest furnishing *p*-Cl bis-arene **5c** in 73% isolated yield. Pleasingly, *meta*-methoxy and methyl analogues delivered **5g** and **5h** in 62 and 42% yields. Pleasingly, naphthyl-substituted **1** furnished **5i** in 46% yield. Next, analysis of alternate electron-withdrawing moieties upon **1** enabled the effective synthesis of phenyl (**5j**), methyl (**5k**) and *n*-butyl (**5l**) ketones in modest yields and diastereomeric ratio. Next, we turned our attention to alternate arene coupling partners in the reaction with **1a**. Pleasingly, toluene and *N*,*N*-dimethylaniline enabled the formation of **5m** and **5n**, albeit in lower yields than those with anisole (*vide infra*). Unsurprisingly, bromobenzene was insufficiently nucleophilic to partake in arylation. Next, reaction with 4-bromoanisole and 1,4-dimethoxybenzene delivered **5p** and **5q** in 45 and 22% yields, respectively, while *p*-xylene formed **5r** in a low 14% yield, demonstrating the clear steric effect of *ortho*-substituents on the reaction outcome. Pleasingly, highly encumbered mesitylene was efficacious for arylation, forming **5s** in 20% yield. Finally, benzodioxoles delivered **5t** in 39% yield, while tetralin and naphthalene formed **5u** and **5v** in 66 and 10% yields, respectively.

Due to the success of aldehydes and electron-rich arenes at enabling annulation and C–C arylation, respectively, we sought to better understand the role of the presumed radical cation formed from BCB oxidation. We speculated benzaldehyde oxidation/reduction potentials to be within this reactive manifold range,^23,24^ evidenced by the detection of alcohol byproducts of aldehydes upon reaction completion and the cycloaddition reactions proclivity for CH_2_Cl_2_ as the solvent (Figure 2A).^20^ We examined the possibility of these alternate coupling partners/intermediates experimentally by subjecting **1a** to reaction with benzyl alcohol or 4-methoxybenzoic acid; however, neither **3a** nor **6** was detected. Additionally, styrene oxide has been shown to undergo Lewis-acid-catalyzed cycloaddition with BCBs.^21^ However, under our described conditions, no oxo-cyclohexane analogue **7** was detected, thus precluding Lewis acidic activation. Notably, minimal conversion of all alternate coupling partners was observed, indicating that preliminary electrochemical activation of aldehydes is not an operative pathway.

**Figure 2:**
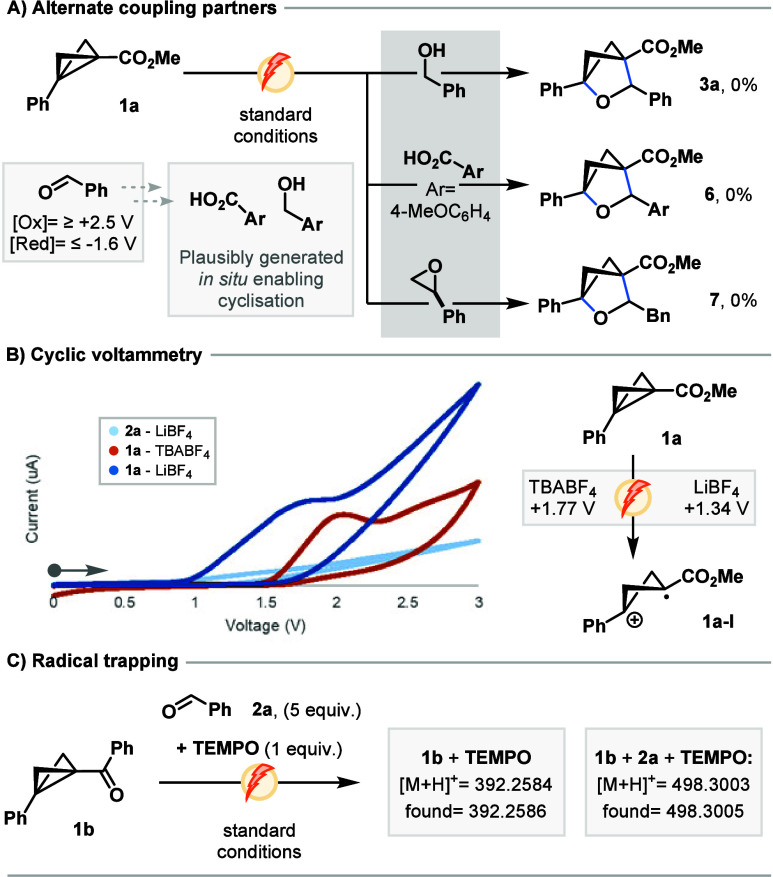
Mechanistic insights. (A) Reaction conditions: **1a** (0.1 mmol), alternate coupling partners (0.5 mmol), LiBF_4_ (0.12 mmol), CH_2_Cl_2_/DMF (20:1), 5 mA, and 2 F/mol. (B) Cyclic voltammetry studies: 0.1 mmol of BCB or benzaldehyde with TBABF_4_ (0.025 M) in MeCN (orange) or LiBF_4_ (0.025 M) in CH_2_Cl_2_/DMF (20:1) (light and dark blue), with IUPAC convention and the anodic scan direction first. (C) Radical trapping experiment, with the reaction conditions as in panel A.

The oxidative generation of BCB radical cations is well-studied photochemically,^25^ though their presence solely through electrochemical means has not been reported. Cyclic voltammograms of benzaldehyde displayed minimal redox activity through the anodic region (Figure 2 and section 4.1 of the Supporting Information), while oxidation of **1a** occurred within an easily accessed redox window with TBABF_4_ as the electrolyte (*E*_1/2_ = +1.74 V). Notably, the potential required for **1a** oxidation is significantly lower with LiBF_4_ in the CH_2_Cl_2_/DMF mixture (*E*_1/2_ = +1.34 V; Figure 2B).

Next, single-electron oxidation of BCB substrates **1** is the expected operative pathway here, with the generation of a radical α to the electron-withdrawing moiety predicated. To this end, we examined the reaction of **1b** and benzaldehyde with 1 equivalent of 2,2,6,6-tetramethylpiperidine 1-oxyl (TEMPO). The formation of **3g** was suppressed, while the mass of TEMPO-trapped **1b** was detected alongside that of an adduct of **1b**, **2a**, and TEMPO (Figure 2C). Notably, *N*-oxyl radicals have widespread use as electrochemical mediators within electrosynthesis; thus complete cessation of reactivity was not expected (Table SI-2).^26^

Taken together, our working hypothesis begins through anodic single-electron oxidation of aromatic systems in BCB substrates (**1**) forming **I** and then common radical cation **II** following ring opening (Scheme 3), highlighting the necessity of an Ar-embedded BCB for successful reactions. Coupling with either aldehydes or arenes generates oxonium **III** or aryl cation **IV**, respectively. Annulation and proton transfer mediated by cathodic single-electron reduction furnishes oBCHs **3**, while biaryl cyclobutanes **5** are the product of cathodic reduction followed by H^+^ coupling or through capture by H^•^, generated at the cathode through reduction of protic HFIP.

**Scheme 3:**
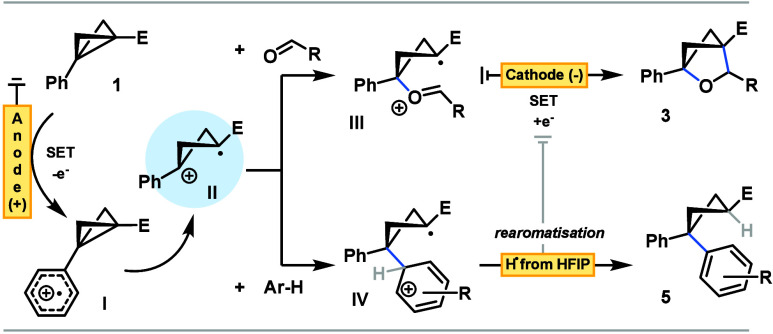
Mechanistic Postulate

In conclusion, strain release chemistry has been a cornerstone of practical chemical transformations for decades.^1,2,7,14^ Employing electrochemical means, we have identified and developed a novel approach to the single-electron oxidative activation of bicyclo[1.1.0]­butane substrates. This catalyst-free approach offers an operationally simple protocol establishing a highly effective avenue for the synthesis of a broad array of oxobicyclohexanes and geminal bis-arylated cyclobutanes. Our mechanistic investigation confirmed the generation and harnessing of highly reactive bicyclo[1.1.0]­butane radical cation intermediates. We are convinced that this method for accessing such reactive manifolds provides a bedrock for future discoveries via these privileged intermediates.

## Supplementary Material



## Data Availability

The data underlying this study are available in the published article and its Supporting Information.

## Notes

Detailed experimental procedures, analytical data, and ^1^H, ^13^C, and ^19^F NMR spectra of all new compounds (PDF)

## References

[1] Turkowska J., Durka J., Gryko D. (2020). Strain release—An old tool
for new transformations. Chem. Commun..

[2] Golfmann M., Walker J. C. L. (2023). Bicyclobutanes as unusual building blocks for complexity
generation in organic synthesis. Commun. Chem..

[3] Perkin W. H., Simonsen J. L. (1905). The synthetical
formation of bridged rings. Part II.
Some derivatives of dicyclobutane. Proc. Chem.
Soc. London.

[4] Levterov V. V., Panasyuk Y., Pivnytska V. O., Mykhailiuk P. K. (2020). Water-soluble
non-classical benzene mimetics. Angew. Chem.,
Int. Ed..

[5] Brown D. G., Boström J. (2016). Analysis of Past and Present Synthetic
Methodologies on Medicinal Chemistry: Where Have All the New Reactions
Gone?. J. Med. Chem..

[6] Attard F. C., Slobodianyk A., Bychek R., Panasiuk Y., Neigenfind P., Massaro L., Gardiner M. G., Levterov V. V., Baran P. S., Mykhailiuk P. K., Malins L. R. (2025). Dibromocarbene addition
to bicyclo[1.1.0]­butanes: A facile route to substituted bicyclo[1.1.1]­pentanes. Proc. Natl. Acad. Sci. U.S.A..

[7] Knyazev D. A., George M., Werz D. B. (2025). (3 + 2)-Cycloaddition
of bicyclobutanes
and thioketones: access to 2-thiabicyclo[2.1.1]­hexanes without the
use of catalysts or light. Chem. Sci..

[8] Radhoff N., Daniliuc C. G., Studer A. (2023). Lewis acid
catalyzed
formal (3 + 2)-cycloaddition of bicyclo[1.1.0]­butanes with ketenes. Angew. Chem., Int. Ed..

[9] Lopchuk J. M., Fjelbye K., Kawamata Y., Malins L. R., Pan C. M., Gianatassio R., Wang J., Prieto L., Bradow J., Brandt T. A., Collins M. R., Elleraas J., Ewanicki J., Farrell W., Fadeyi O. O., Gallego G. M., Mousseau J. J., Oliver R., Sach N. W., Smith J. K., Spangler J. E., Zhu H., Zhu J., Baran P. S. (2017). Strain-release
heteroatom functionalization: development, scope, and stereospecificity. J. Am. Chem. Soc..

[10] Ma X., Sloman D. L., Han Y., Bennett D. J. A. (2019). Selective Synthesis
of 2,2-Difluorobicyclo[1.1.1]­pentane
Analogues: “BCP-F2”. Org. Lett..

[11] Agasti S., Beltran F., Pye E., Kaltsoyannis N., Crisenza G. E. M., Procter D. J. (2023). A catalytic alkene
insertion approach to bicyclo[2.1.1]­hexane bioisosteres. Nat. Chem..

[12] Golfmann M., Reinhold M., Steen J. D., Deike M. S., Rodemann B., Golz C., Crespi S., Walker J. C. L. (2024). Photocatalytic Oxidative Activation of Bicyclo[1.1.0]­butanes
for Formal [2σ + 2π] Cycloadditions. ACS Catal..

[13] Guo R., Chang Y.-C., Herter L., Salome C., Braley S. E., Fessard T. C., Brown M. K. (2022). Strain-Release
[2π + 2σ] Cycloadditions for the Synthesis of Bicyclo[2.1.1]­hexanes
Initiated by Energy Transfer. J. Am. Chem. Soc..

[14] a Donor Acceptor Cyclopropanes in Organic Synthesis; Biju, A. , Banerjee, P. , Eds.; Wiley-VCH GmbH: Weinheim, Germany, 2024; 10.1002/9783527835652.

[15] Kolb S., Ahlburg N. L., Werz D. B. (2021). Friedel–Crafts-type
reactions with electrochemically generated electrophiles from donor–acceptor
cyclopropanes and-butanes. Org. Lett..

[16] Yan M., Kawamata Y., Baran P. S. (2017). Synthetic Organic Electrochemical
Methods Since 2000: On the Verge of a Renaissance. Chem. Rev..

[17] Kingston C., Palkowitz M. D., Takahira Y., Vantourout J. C., Peters B. K., Kawamata Y., Baran P. S. (2020). A Survival Guide
for the “Electro-curious”. Acc.
Chem. Res..

[18] Maity R., Maji B. (2025). Electrophotocatalytic
Radical Cascade Reaction for the Synthesis of Trifluoromethylated
Spirocyclobutyl Oxindoles. Org. Lett..

[19] Zhou X., Hu Y., Huang Y., Xiong Y. (2024). Recent advances in photochemical strain-release reactions of bicyclo[1.1.0]­butanes. Chem. Commun..

[20] Xiang J., Shang M., Kawamata Y., Lundberg H., Reisberg S. H., Chen M., Mykhailiuk P., Beutner G., Collins M. R., Davies A., Del Bel M., Gallego G. M., Spangler J. E., Starr J., Yang S., Blackmond D. G., Baran P. S. (2019). Hindered dialkyl ether synthesis with electrogenerated
carbocations. Nature.

[21] Liang Y., Paulus F., Daniliuc D. G., Glorius F. (2023). Catalytic formal [2π
+ 2σ] cycloaddition of aldehydes with bicyclobutanes: expedient
access to polysubstituted 2-oxabicyclo[2.1.1]­hexanes. Angew. Chem., Int. Ed..

[22] Gupta P., Roy P., Biju A. T. (2025). HFIP-Mediated
Ring Opening of Bicyclo[1.1.0]­butanes with Hydroperoxides for Diastereoselective
Access to Peroxycyclobutanes. Org. Lett..

[23] Doherty A. P., Brooks C. A. (2004). Electrosynthesis in room-temperature ionic liquids:
benzaldehyde reduction. Electrochim. Acta.

[24] Kiss L., Kunsagi-Mate S. (2019). Electrochemical
oxidation of benzaldehyde and hydroxybenzaldehydes
in acetonitrile on platinum and glassy carbon electrodes. C. R. Chimica.

[25] Dutta S., Lee D., Ozols K., Daniliuc C. G., Shintani R., Glorius F. (2024). Photoredox-enabled
dearomative [2π + 2σ] cycloaddition of phenols. J. Am. Chem. Soc..

[26] Nutting J. E., Rafiee M., Stahl S. S. (2018). Tetramethylpiperidine *N*-Oxyl (TEMPO), Phthalimide *N*-Oxyl (PINO), and Related *N*-Oxyl Species: Electrochemical Properties and Their Use
in Electrocatalytic Reactions. Chem. Rev..

