# Supplementary material for: Electrochemical Activation of Bicyclo[1.1.0]butanes
Source: Org Lett. 2026 Feb 13;28(8):2573–8. doi: 10.1021/acs.orglett.5c05325 (PMC12954854; doi:10.1021/acs.orglett.5c05325)
Supplement: Supplementary file 1 [file ol5c05325_si_001.pdf]

Electrochemical Activation of Bicyclo[1.1.0]butanes

Supporting Information

Jeremy T. Maddigan-Wyatt, Daniil A. Knyazev, and Daniel B. Werz*

Albert-Ludwigs-Universität Freiburg, Institute of Organic Chemistry, Albertstr. 21, 79104, Freiburg, Germany

Table of Contents

Table of Contents....................2
1. General Experimental....................3
2. Optimization for electrochemical cyclisation....................4
Table SI-1: Optimization of electrolyte and solvent with methyl ester BCB 1a....................4
Table SI-2: Optimization of electrochemical parameters with methyl ester BCB 1a....................5
Table SI-3: Optimization with phenyl ketone BCB....................6
3. Optimization for electrochemical Friedel-Crafts arylation....................7
Table SI-4: Optimization with methyl ester BCB....................7
4. General procedures:....................8
General procedures for cycloaddition with aldehydes....................8
General method for BCB arylation....................9
Synthesis of bicyclo[1.1.0]butanes....................9
Photographs of experimental set-up....................10
Scale up procedures....................12
5. Mechanistic experiments:....................13
5.1. Cyclic voltammetry....................13
5.2. Alternate coupling partners – benzaldehyde equivalents....................15
5.3. Radical trapping experiment....................16
6. Tabulated data....................17
6.1. Oxabicyclo[2.1.1]hexane products....................17
6.2. Arylation products....................33
7. Spectral data of products....................54
8....................100
References....................100

1. General Experimental

All solvents were distilled before use and stored over molecular sieves unless otherwise stated. For electrochemical reactions commercially available hexafluoroisopropanol (HFIP, 99%) and dichloromethane, DMF, THF and acetonitrile (MeCN, p.a.) were used. Air- and moisture-sensitive reactions were carried out in oven-dried or flame-dried glassware, septum-capped under atmospheric pressure of argon or N_2. Commercially available compounds were used without further purification unless otherwise stated. For all purifications by column chromatography silica gel Geduran Si 60 (40-63 µm pore size) from Merck and mixtures of pentane/ethyl acetate were used. Proton (^{1}H), carbon (^{13}C) and fluorine (^{19}F) NMR spectra were recorded on a Bruker AVIII400, Bruker AVIIIHD500 or Bruker AVII600 instrument using the residual signals from $CHCl_3$, δ = 7.26 ppm and δ = 77.16 ppm or CD_3OD δ = 3.31 ppm and δ = 49.00 ppm, as internal reference for ^{1}H and ^{13}C chemical shifts, respectively. Additionally, tetramethylsilane (TMS; δ = 0.00 ppm; 0.03%) was added to NMR samples. The following abbreviations were used for ^{1}H and ^{13}C NMR chemical shifts: s = singlet, d = doublet, t = triplet, m = multiplet. The chemical shift δ is given in ppm. ESI-HRMS was carried out on an FTICR instrument and EIHRMS was carried out on a Jeol AccuTOF GC JMS-T100GC instrument. IR spectra were recorded on an ATR spectrometer Tensor 27 from Bruker. IR data is reported as follows: w = weak, m = medium, s = strong, br = broad or combinations thereof. Melting points of solid products were recorded on a Büchi Melting Point M-560. Cyclic voltammetry was performed with a Gamry Instrument Interface 1010 Potentiostat/ Galvanostat/ZRA). A self-made three electrode setup with two platinum wires (0.5 mm diameter) as working and counter electrodes and a silver wire (0.5 mm diameter) as a quasi-reference electrode was used. For all electrochemical reactions an IKA ElectraSyn 2.0 apparatus was used with electrodes purchased through IKA. Exact reaction conditions are given in the following procedures.

2. Optimization for electrochemical cyclisation

Table SI-1: Optimization of electrolyte and solvent with methyl ester BCB 1a

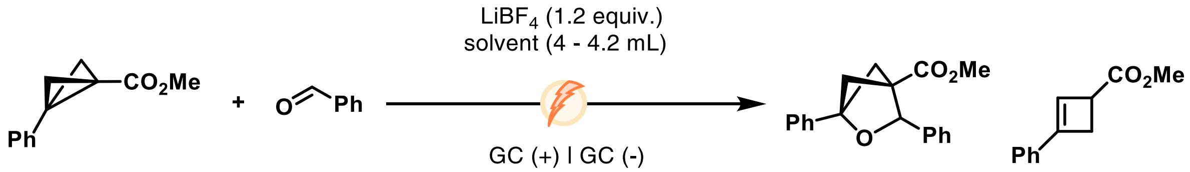

1a **2** **3a** **P2**

Entry	Variation from section conditions	Yield 3a (%) [a]	Yield P2 (%) [a]
**Electrolyte evaluation:** 1.2 equiv. electrolyte, CH_2Cl_2, CCE= 2 mA, 2F/ mol, no polarity switching			
1	$TBABF_4$	30	18
2	TBA-ClO_4	26	28
3	TBA-Br	0	79
4	TBA-Ac	0 (NR)	0
5	TBA-NO_3	0	95
6	$TBAPF_6$	41	28
7	H_4NBr	0 (NR)	0
8	$LiBF_4$ (+0.2 mL DMA)	43	trace
9	$LiBF_4$ (CH_2Cl_2: DMA = 3.5 : 0.5 mL)	57	8
**Solvent system evaluation:** solvent + DMA (4 + 0.2 mL), CCE= 10 mA, 2F/ mol, polarity switch= 1 min^{-1}			
10	DMA	60	6
11	DMF (no DMA)	56	10
12	CH_2Cl_2	57	6
13	MeCN	50	10
14	Acetone	47	trace
15	$MeNO_2$	45	5
16	HFIP	18 + HFIP addition	trace
17	THF	66 (61)	7
18	THF (8 mL)	27	trace

[a] Yield estimated from the ^{1}H-NMR of the crude reaction mixture relative to CH_2Br_2 as internal standard. Isolated yield represented in parantheses.

Table SI-2: Optimization of electrochemical parameters with methyl ester BCB 1a

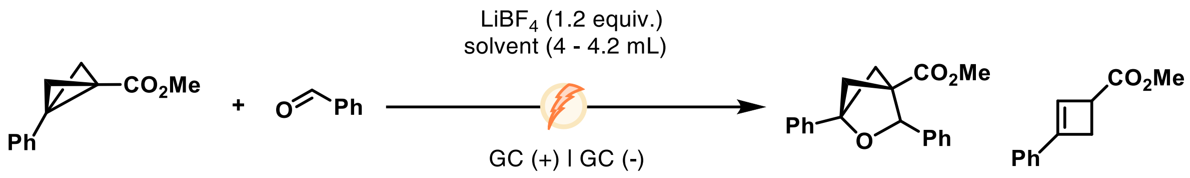

1a | 2 | 3a | P2

Entry	Variation from section conditions	Yield 3a (%) [a]	Yield P2 (%) [a]
	**Electrode evaluation:** THF: DMF (4: 0.2 mL), CCE= 5 mA, 2 F/ mol, no polarity switch		
1	Mg (+) | GC (-)	53	7
2	Ni (+) | GC (-)	42	10
3	GC (+) | Ni (-)	37	3
4	GC (+) | stainless steel (-)	47	1
5	graphite (+) | graphite (-)	39	7
	**Electrochemical parameter evaluation:** THF: DMA (4: 0.2 mL), CCE= 10 mA, 2 F/ mol, polarity switch= 1 min^{-1}		
6	CCE, 0.3 mA, 1F/ mol (≈ CVE, 2.5 V, 1F/ mol)	35	4
7	20 mA, 1F/ mol	25	5
8	Pol switch = 2 s^{-1}	35	3
9	NPS	45	9
10	5 mA	65 (61)	6
11	2 mA	66	10
12	5 mA, 1 F/ mol	42	2
13	1 mA, 1 F/ mol	39	5
14	5 mA, 10 F/ mol	1	2
15	0.2 mmol scale, 5 mA, 2 F/ mol	41	3
	**Additive evaluation:** THF: DMF (4: 0.2 mL), CCE= 10 mA, 2 F/ mol, polarity switch= 1 min^{-1}		
17	+ quinuclidine (1 equiv.)	0	n.d.
18	+ Ag_2O (1 equiv.)	22	n.d.
19	O=PPh_3 (1 equiv.)	Trace	n.d.
20	+ DDQ (1 equiv.) with MeCN	8	n.d.
21	TEMPO (1 equiv.)	44*	n.d.
22	CoTPP (2.5 mol%), GC (+/-), 5 mA, 2 F/ mol	55	n.d.
23	CoTPP (2.5 mol%), GC (+/-), 2.5 mA, 1.1 F/ mol	68	n.d.
24	CoTPP (3 mol%), GC (+/-), 1.5 mA, 0.9 F/ mol	20	n.d.
25	CoTPP (3 mol%), GC (-), Mg (+), 2.5 mA, 1.1 F/ mol	19	n.d.

[a] Yield estimated from the ^{1}H-NMR of the crude reaction mixture relative to CH_2Br_2 as internal standard. Isolated yield represented in parantheses.

Table SI-3: Optimization with phenyl ketone BCB

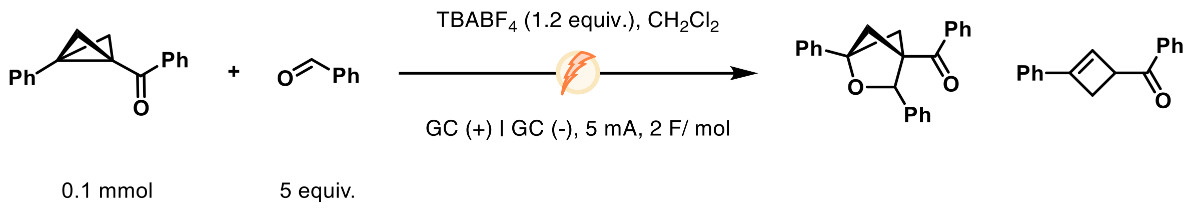

Entry	BA equiv.	Yield (%)	Cyclobutene (%)
1	1	7	21
2	3	15	63
3	7.5	26	52
4	10	33	37
	**Current (mA)**	*with 5 equiv. benzaldehyde, 2 F/ mol*	
5	1	16	nd
6	2	30	nd
7	5	25	nd
	**Electrolyte equiv.**	*with 5 equiv. benzaldehyde, 2 mA, 2 F/ mol*	
8	0.5	25	nd
9	1	45 (39)	nd
10	2	22	nd

[a] Yield estimated from the ^{1}H-NMR of the crude reaction mixture relative to CH_2Br_2 as internal standard.

3. Optimization for electrochemical Friedel-Crafts arylation

Table SI-4: Optimization with methyl ester BCB

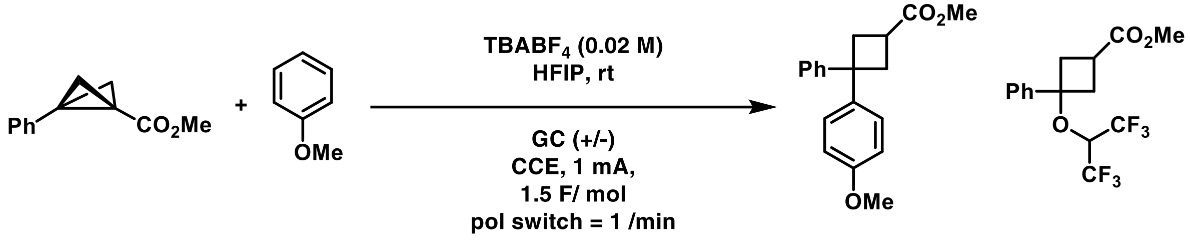

Entry	Variation from standard conditions	Yield P1 (%) [a]	Yield P2 (%) [a]
	**Standard conditions**		
1	No changes	65 (60)	trace
2	Anisole (3 equiv.)	34	15
	**Solvent system evaluation**		
3	MeCN	n.d.	n.d.
4	CH_2Cl_2	n.d.	n.d.
	**Electrochemical parameter evaluation**		
5	Graphite (+/-)	47	
6	RVC (+/-)	56	
7	CVE= 10 V	50	trace
8	CVE= 3 V	40	trace
9	CCE= 5 mA	48	trace
10	Divided cell	42	
	**Additive evaluation**		
11	Open to air	45	trace
12	Ar atmosphere (no purging)	54	trace
13	No current, 4 h, rt	32	trace
14	No current, toluene (10 equiv.) instead of anisole, 4 h, rt	trace	trace
15	No current, mesitylene (10 equiv.) instead of anisole, 4 h, rt	n.d.	trace
16	No current, tetrahydronaphthalin (10 equiv.) instead of anisole, 4 h, rt	trace	trace
	**Mesitylene optimization (Mesitylene (10 equiv.) instead of anisole)**		
17	No changes	26 (20)	trace
18	DDQ (25 mol%), CVE= +1.3V	n.d.	16
19	HFIP: CH_2Cl_2 (1:1)	trace	trace
20	HFIP: MeCN (1:1)	trace	trace
21	Graphite (+/-)	10	14
22	RVC (+/-)	18	15
23	10 V	23	trace
24	Divided cell	Detected	Detected

[a] Yield estimated from the ^{1}H-NMR of the crude reaction mixture relative to CH_2Br_2 as internal standard. Isolated yield represented in parantheses.

4. General procedures:

General procedures for cycloaddition with aldehydes

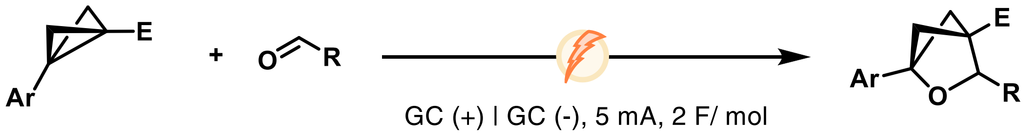

1 **2** **3**

Conditions:
[A]: $LiBF_4$, THF: DMF (20:1)
[B]: $LiBF_4$, CH_2Cl_2: DMF (20:1)
[C]: $TBABF_4$, CH_2Cl_2

General method 1A (GP-1[A]):

To an oven dried (cooled to room temperature) 5 mL ElectraSyn vial was added BCB (0.10 mmol, 1.0 eq.), $LiBF_4$ (1.0 – 1.3 equiv.) and benzaldehyde (0.50 mmol, 5.0 eq.). A vial cap fitted with dual glassy carbon electrodes was fitted and the vial was evacuated and backfilled with N_2 three times. The material was solubilized in THF: DMF (4: 0.2 mL) and the solution was electrolyzed using the alternating current mode at a constant current as detailed (alternation of current = 1 min^{-1}). The reaction mixture was stirred at 1500 rpm at room temperature. After reaction completion, the reaction mixture was concentrated *in vacuo* and the residue was purified by column chromatography.

General method 1B (GP-1[B]):

To an oven dried (cooled to room temperature) 5 mL ElectraSyn vial was added BCB (0.10 mmol, 1.0 eq.), $LiBF_4$ (1.0 – 1.3 equiv.) and benzaldehyde (0.50 mmol, 5.0 eq.). A vial cap fitted with dual glassy carbon electrodes was fitted and the vial was evacuated and backfilled with N_2 three times. The material was solubilized in CH_2Cl_2: DMF (4: 0.2 mL) and the solution was electrolyzed using the alternating current mode at a constant current as detailed (alternation of current = 1 min^{-1}). The reaction mixture was stirred at 1500 rpm at room temperature. After reaction completion, the reaction mixture was concentrated *in vacuo* and the residue was purified by column chromatography.

General method 1C (GP-1[C]):

To an oven dried (cooled to room temperature) 5 mL ElectraSyn vial was added BCB (0.10 mmol, 1.0 eq.), $TBABF_4$ (1.1 equiv.) and benzaldehyde (0.50 mmol, 5.0 eq.). A vial cap fitted with dual glassy carbon electrodes was fitted and the vial was evacuated and backfilled with N_2 three times. The material was solubilized in CH_2Cl_2 (4 mL) and the solution was electrolyzed using the alternating current mode at a constant current as detailed (alternation of current = 1 min^{-1}). The reaction mixture was stirred at 1500 rpm at room temperature. After reaction completion, the reaction mixture was concentrated *in vacuo* and the residue was purified by column chromatography.

Note: any variation in electrochemical conditions is noted for specific compounds in tabulated data.

General method for BCB arylation

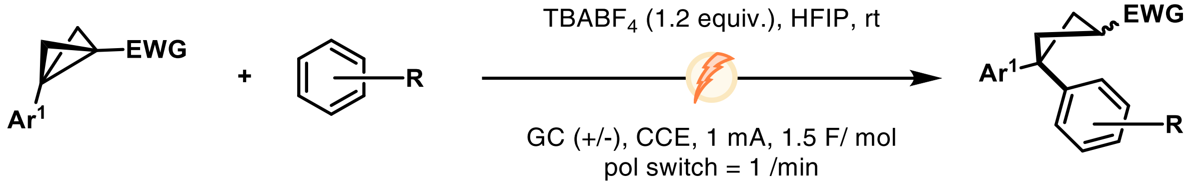

0.1 mmol **10 equiv.**

General method 2 (GP-2):

To an oven dried (cooled to room temperature) 5 mL ElectraSyn vial was added BCB (0.10 mmol, 1.0 eq.), $TBABF_4$ (1.2 equiv.) and arene (10.0 eq.). A vial cap fitted with dual glassy carbon electrodes was fitted and the vial was evacuated and backfilled with N_2 three times. The material was solubilized with HFIP (4 mL) and a vial cap fitted with dual GC electrodes was fitted. The solution was sparged for 1 minute with N_2, then electrolyzed using the alternating constant current mode at 1 mA, 1.5 F/ mol (alternation of current = 1 min^{-1}). The reaction mixture was stirred at 400 rpm at room temperature. After reaction completion, the reaction mixture was concentrated *in vacuo* and the residue was adsorbed on silica gel. The desired products **5** were purified by column chromatography.

Synthesis of bicyclo[1.1.0]butanes

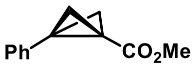

BCB-1

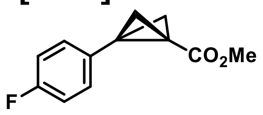

BCB-2

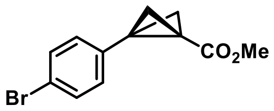

BCB-3

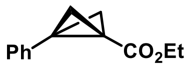

BCB-4

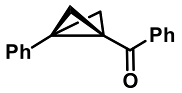

BCB-5

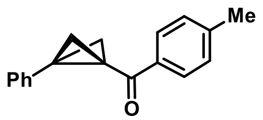

BCB-6

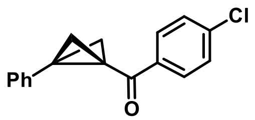

BCB-7

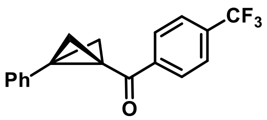

BCB-8

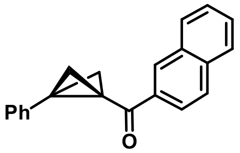

BCB-9

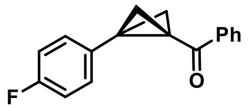

BCB-10

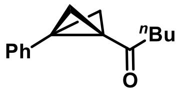

BCB-11

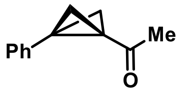

BCB-12

Figure SI-x: Bicyclo[1.1.0]butanes synthesized in accordance with literature

BCB-1, 2, 5, 11, were synthesized and data in accordance with literature. [1]

BCB-6, 7, 8, 9, 12 were synthesized and data in accordance with literature. [2]

Photographs of experimental set-up

Representative of annulation chemistry (BCB + aldehydes)

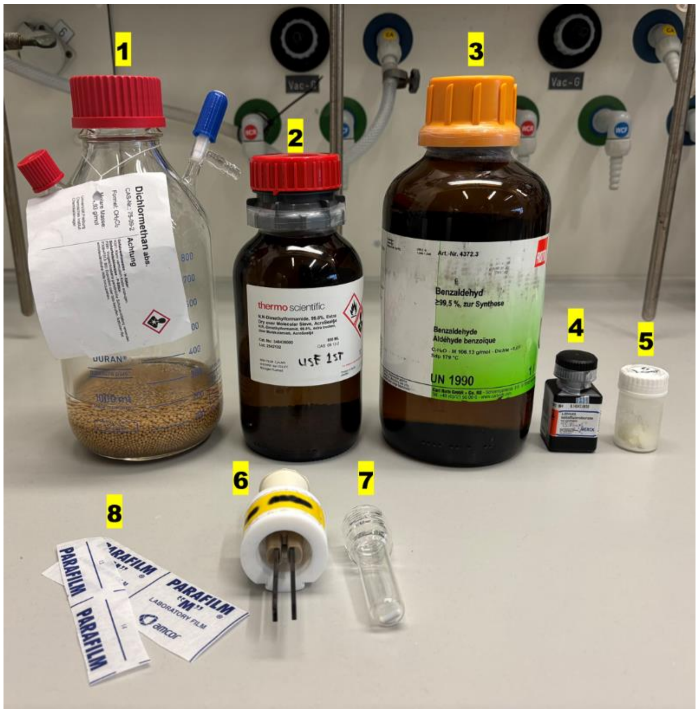

Picture 1. Reagents, solvents and glassware used:

1. Solvent (CH_2Cl_2 used here).
2. DMF.
3. Benzaldehyde.
4. $LiBF_4$.
5. Bicyclobutane substrate (here BCB-2, for synthesis of **3h**.
6. IKA-Syn vial cap, fitted with dual glassy carbon electrodes.
7. 5 mL IKA-Syn vial with magnetic stir bar.
8. Parafilm.

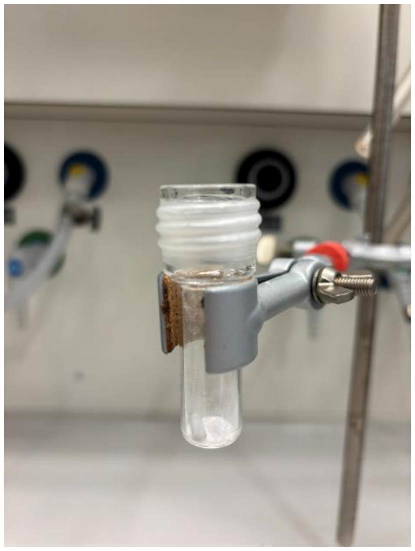

Picture 2. Reaction vial containing solid reagents, parafilm used for greater sealing ability of flask.

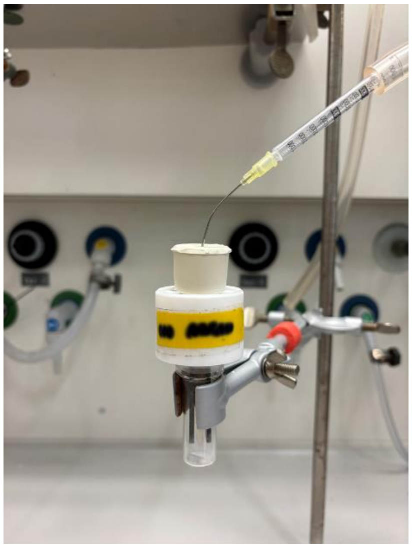

Picture 3. Vial cap attached and flask evacuated/ refilled (N_2) 3 times.

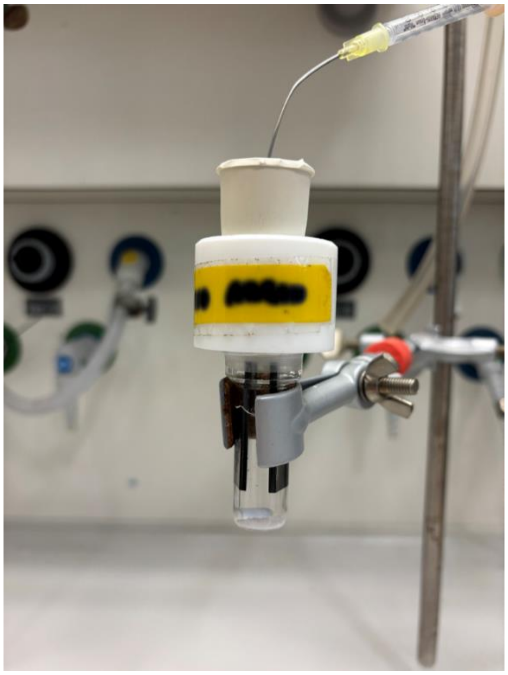

Picture 4. Liquid/ oil substrates and solvent(s) added to flask. $LiBF_4$ electrolyte dissolution occurs upon stirring of solution with DMF added.

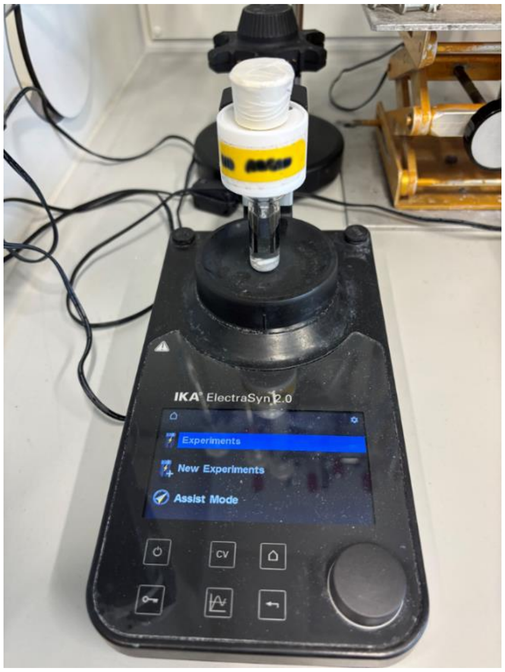

Picture 5. Suba-seal carefully sealed with more parafilm, and reaction flask attached to IKA-Syn.

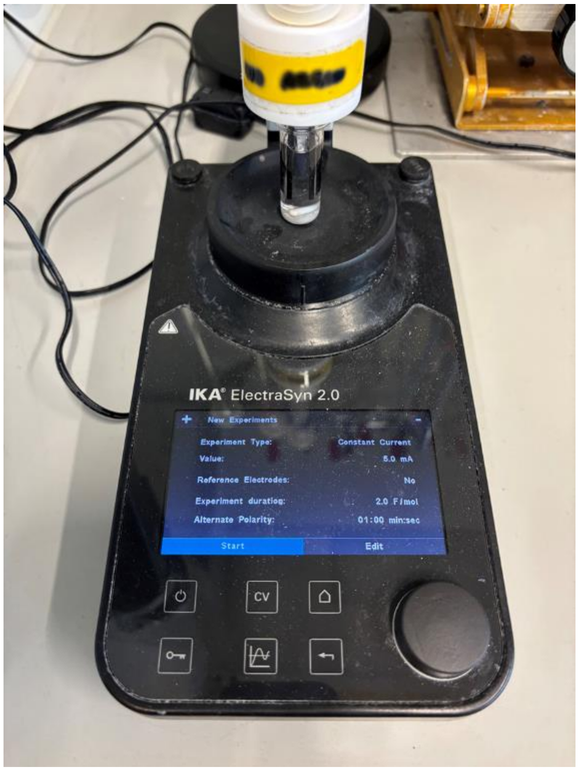

Picture 6. Electrochemical reaction conditions for 0.1 mmol reaction: constant current: 5 mA, 2 F/ mol, alternation of electrode polarity= 1 min^{-1}, 1500 rpm.

Scale up procedures

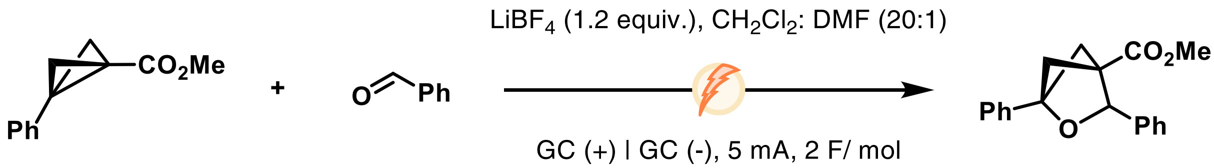

1a **2** **3a**

Scale up procedure for cyclisation:

Following GP-1B to an oven dried (cooled to room temperature) 10 mL ElectraSyn vial was added **1a** (0.50 mmol, 1.0 eq.), $LiBF_4$ (1.2 equiv.) and benzaldehyde (2.50 mmol, 5.0 eq.). A vial cap fitted with dual glassy carbon electrodes was fitted and the vial was evacuated and backfilled with N_2 three times. The material was solubilized in CH_2Cl_2: DMF (11.5: 0.5 mL – overfilled) and the solution was electrolyzed using the alternating current mode at a constant current of 5 mA, for 2 F/ mol (alternation of current = 1 min^{-1}). The reaction mixture was stirred at 400 rpm at room temperature. After reaction completion, the reaction mixture was concentrated *in vacuo* and the residue was purified by column chromatography delivering **3a** in 40% yield (59 mg, 0.20 mmol).

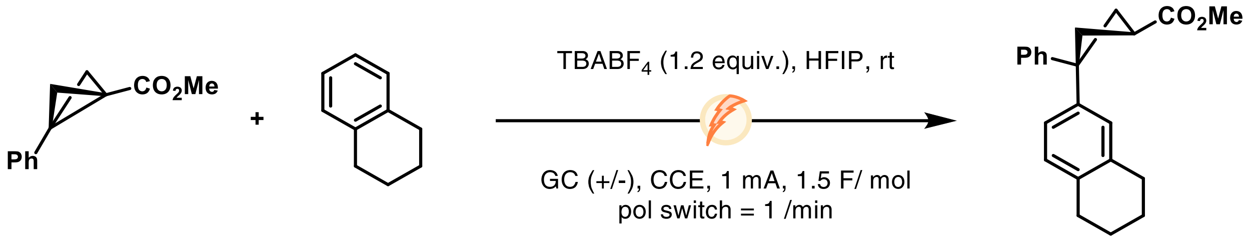

1a
1.0 mmol **10 equiv.** **5u**

Scale up procedure for arylation:

Following GP-2, to an oven dried (cooled to room temperature) a 10 mL ElectraSyn vial was added **1a** (1.0 mmol, 1.0 eq.), $TBABF_4$ (1.2 equiv.) and tetralin (10.0 eq.). A vial cap fitted with dual glassy carbon electrodes was fitted and the vial was evacuated and backfilled with N_2 three times. The material was solubilized with HFIP (12 mL – overfilled) and a vial cap fitted with dual GC electrodes was fitted. The solution was sparged for 1 minute with N_2, then electrolyzed using the alternating constant current mode at 1 mA, 1.5 F/ mol (alternation of current = 1 min^{-1}). The reaction mixture was stirred at 400 rpm at room temperature. After reaction completion, the reaction mixture was concentrated *in vacuo* and the residue was adsorbed on silica gel, with column chromatography delivering **5u** in 67% yield (197 mg, 0.669 mmol).

5. Mechanistic experiments:

5.1. Cyclic voltammetry

Cyclic voltammetry recorded using the following conditions: scan rate = 100 mV/ s (anodic scan first, beginning at 0 V); **1** [0.025 M] or benzaldehyde [0.125 M]; where $TBABF_4$ used as supporting electrolyte = 0.03 M in MeCN (4 mL), where $LiBF_4$ used as supporting electrolyte = 0.03 M in CH_2Cl_2: DMF (4: 0.2 mL); glassy carbon working electrode (3 mm diameter), platinum wire counter and quasi-reference electrodes. Sample mixtures were degassed by N_2 sparging for 30 seconds prior to CV recording. Voltammograms are represented according to IUPAC plotting convention. Ferrocene was used as internal standard in subsequent scans, and oxidation potentials calculated using the local maxima or the points of inflection respectively and are corrected against Fc/Fc^+ redox couple.

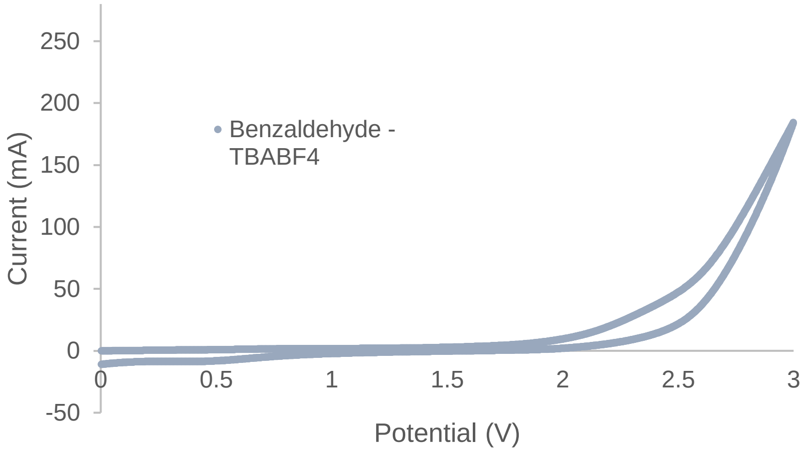

CV-1. Cyclic voltammogram of benzaldehyde [0.125 M] in MeCN with $TBABF_4$ as electrolyte.

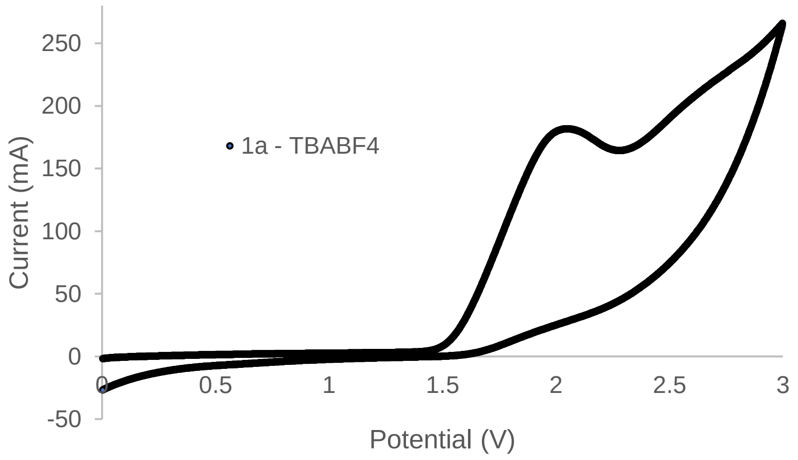

CV-2. Cyclic voltammogram of **1a** [0.025 M] in MeCN with $TBABF_4$ as electrolyte.

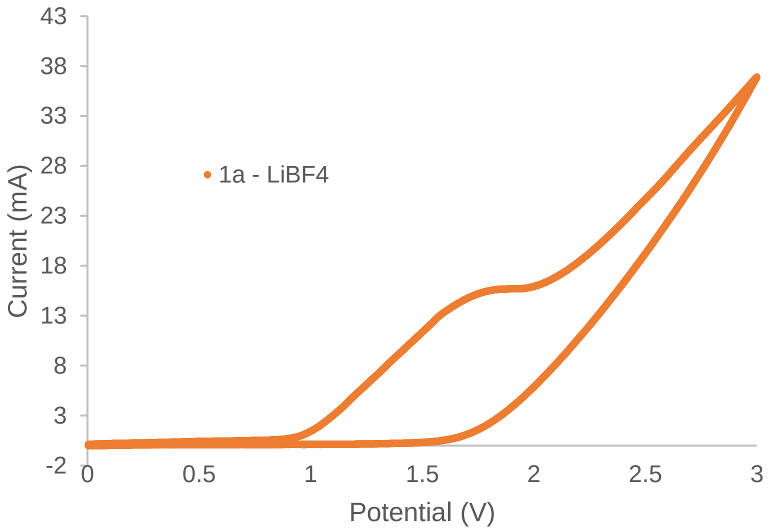

CV-3. Cyclic voltammogram of **1a** [0.025 M] in CH_2Cl_2: DMF with $LiBF_4$ as electrolyte.

Electrodes (GC and Pt) were washed with acetonitrile, then isopropanol and Kimtech Kimwipes were used to carefully wipe and clean the outside of electrodes. No significant amounts of residue build up on electrodes was observed.

5.2. Alternate coupling partners – benzaldehyde equivalents

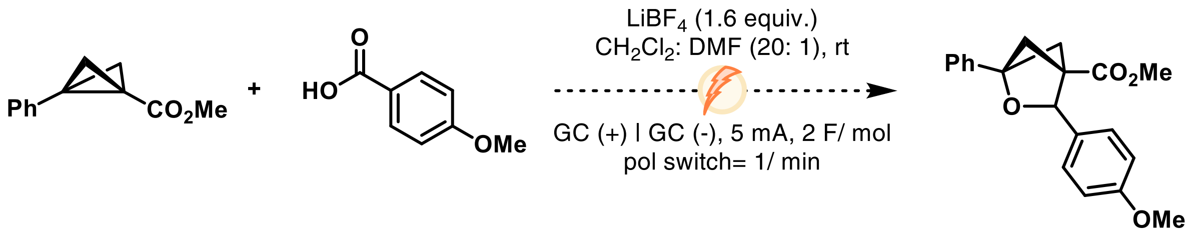

1a 3q

Method: Following GP-1B, a reaction solution comprised of methyl 3-phenylbicyclo[1.1.0]butane-1-carboxylate (18.0 mg, 0.10 mmol), 4-methoxybenzoic acid (74.4 mg, 0.49 mmol) $LiBF_4$ (14.8 mg, 0.16 mmol) in CH_2Cl_2: DMF (4: 0.2 mL) was electrolyzed at dual glassy carbon electrodes under constant current conditions= 5 mA, 2 F/ mol (polarity switch= 1 min^{-1}). After electrolysis, the reaction mixture was reduced *in vacuo* and the crude mixture analyzed by ^{1}H-NMR with CH_2Br_2 as internal standard.

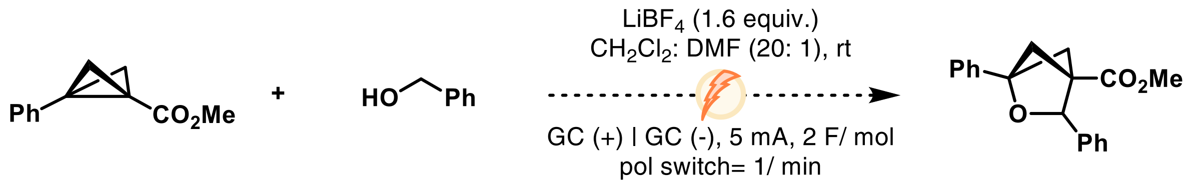

1a 6

Method: Following GP-1B, a reaction solution comprised of methyl 3-phenylbicyclo[1.1.0]butane-1-carboxylate (18.1 mg, 0.10 mmol), benzyl alcohol (59.4 mg, 0.55 mmol), $LiBF_4$ (14.1 mg, 0.15 mmol) in CH_2Cl_2: DMF (4: 0.2 mL) was electrolyzed at dual glassy carbon electrodes under constant current conditions= 5 mA, 2 F/ mol (polarity switch= 1 min^{-1}). After electrolysis, the reaction mixture was reduced *in vacuo* and the crude mixture analyzed by ^{1}H-NMR with CH_2Br_2 as internal standard.

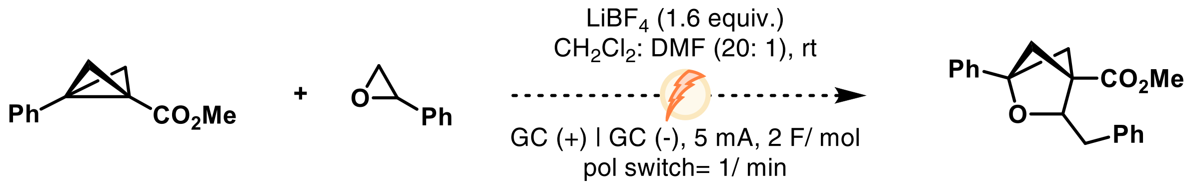

1a (+) 6

Method: Following GP-1B, a reaction solution comprised of methyl 3-phenylbicyclo[1.1.0]butane-1-carboxylate (19.3 mg, 0.10 mmol), (+)-styrene oxide (75.4 mg, 0.63 mmol), $LiBF_4$ (14.8 mg, 0.16 mmol) in CH_2Cl_2: DMF (4: 0.2 mL) was electrolyzed at dual glassy carbon electrodes under constant current conditions= 5 mA, 2 F/ mol (polarity switch= 1 min^{-1}). After electrolysis, the reaction mixture was reduced *in vacuo* and the crude mixture analyzed by ^{1}H-NMR with CH_2Br_2 as internal standard.

5.3. Radical trapping experiment

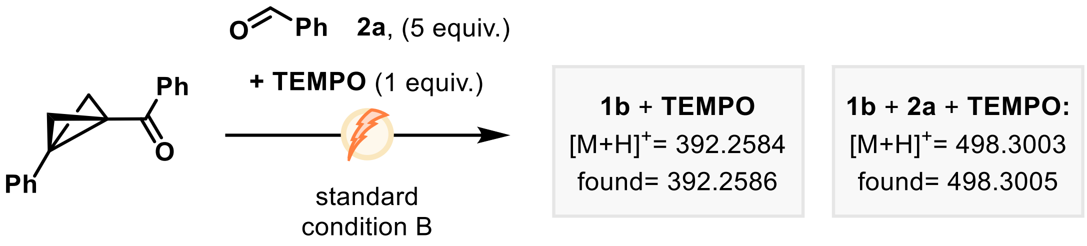

1b

Method: Following GP-1B, a reaction solution comprised of phenyl(3-phenylbicyclo[1.1.0]butan-1-yl)methanone **1b** (24.5 mg, 0.11 mmol), benzaldehyde **2a** (58.3 mg, 0.55 mmol), TEMPO (15.0 mg, 0.10 mmol), $LiBF_4$ (14.5 mg, 0.16 mmol) in CH_2Cl_2: DMF (4: 0.2 mL) was electrolyzed at dual glassy carbon electrodes under constant current conditions= 5 mA, 2 F/ mol (polarity switch= 1 min^{-1}). After electrolysis, the crude reaction mixture was analyzed by HRMS and ^{1}H-NMR with CH_2Br_2 as internal standard.

6. Tabulated data

6.1. Oxabicyclo[2.1.1]hexane products

Methyl 1,3-diphenyl-2-oxabicyclo[2.1.1]hexane-4-carboxylate (3a)

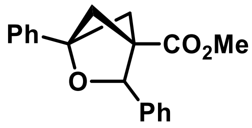

Following GP-1A the title compound was prepared by reaction of methyl 3-phenylbicyclo[1.1.0]butane-1-carboxylate (18.8 mg; 0.10 mmol), benzaldehyde (54.1 mg; 0.51 mmol), $LiBF_4$ (10.3 mg, 0.11 mmol) in a mixture of THF (4 mL) and DMF (0.2 mL); where the solution was electrolyzed at room temperature under alternating constant current conditions, 2 mA and 2 F/ mol (current alternation= 1 min) using glassy carbon anode and cathode. Column chromatography (SiO_2, EtOAc/ *n*-pentane 1: 10) afforded the desired product **3a** (17.9 mg, 0.061 mmol, 61%) as a colorless oily solid. Spectral data matches literature reports. [1]

R_f (EtOAc/ *n*-pentane 1: 10) = 0.31.

^{1}H-NMR (300 MHz, $CDCl_3$): δ = 7.57 – 7.28 (m, 10H), 5.51 (s, 1H), 3.74 (s, 3H), 2.61 (ddd, *J* = 9.7, 7.0, 1.1 Hz, 1H), 2.55 (d, *J* = 7.1 Hz, 1H), 2.36 – 2.28 (m, 1H), 2.55 (d, *J* = 7.0 Hz, 1H).

^{13}C NMR (176 MHz, $CDCl_3$): δ= 170.6, 139.6, 137.1, 128.6, 128.5, 128.2, 127.8, 126.6, 126.3, 86.9, 80.4, 54.8, 51.9, 51.0, 40.0.

IR (ATR): $\tilde{\nu}$ (cm^{-1}) = 3031, 2952, 1730, 1449, 1436, 1356, 1314, 1251, 1200, 1111, 1010, 974, 755, 698.

HRMS (ESI) m/z: $[M+H]^+$ calculated for $C_{19}H_{19}O_3$, 295.1329; found: 295.1324.

Methyl 1-(4-bromophenyl)-3-phenyl-2-oxabicyclo[2.1.1]hexane-4-carboxylate (3b)

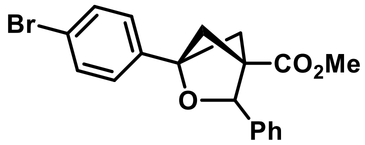

Following GP-1B the title compound was prepared by reaction of methyl 3-(4-bromophenyl)bicyclo[1.1.0]butane-1-carboxylate (28.9 mg; 0.11 mmol), benzaldehyde (55.1 mg; 0.52 mmol), $LiBF_4$ (13.7 mg, 0.15 mmol) in a mixture of CH_2Cl_2: DMF (4: 0.2 mL); where the solution was electrolyzed at room temperature under alternative current conditions, 5 mA and 2 F/ mol (current alternation= 1 min) using glassy carbon anode and cathode. Column chromatography (SiO_2, EtOAc: *n*-pentane, 6:94 – 12:82) afforded the desired product **3b** in 45% yield (18.0 mg, 0.05 mmol) as a colorless solid. Spectral data matches literature reports. [2]

R_f (EtOAc/ *n*-pentane, 1:9) = 0.44.

^{1}H-NMR (300 MHz, $CDCl_3$): δ 7.54 (d, *J* = 8.6 Hz, 2H), 7.43 – 7.41 (m, 2H), 7.39 – 7.37 (m, 2H), 7.36 – 7.34 (m, 2H), 7.32 – 7.27 (m, 1H), 5.49 (s, 1H), 3.73 (s, 3H), 2.57 (ddd, *J* = 10.2, 7.1, 1.0 Hz, 1H), 2.52 (dd, *J* = 7.1, 0.7 Hz, 1H), 2.28 (ddd, *J* = 10.3, 8.1, 0.8 Hz, 1H), 2.21 (dt, *J* = 8.2, 0.7 Hz, 1H).

^{13}C NMR (176 MHz, $CDCl_3$): δ 170.4, 139.4, 136.2, 131.7, 128.3, 128.1, 127.9, 126.6, 122.5, 86.4, 80.5, 54.8, 51.9, 51.1, 40.1.

IR (ATR): $\tilde{\nu}$ (cm^{-1}) = 1733, 1493, 1451, 1436, 1396, 1357, 1314, 1251, 1200, 1111, 1071, 1006, 973, 934, 914, 815, 794, 762, 736, 701.

HRMS (ESI) m/z: $[M+Na]^+$ calculated for $C_{19}H_{17}BrO_3Na$, 395.0253; found: 395.0250.

Methyl 1-(4-fluorophenyl)-3-phenyl-2-oxabicyclo[2.1.1]hexane-4-carboxylate (3c)

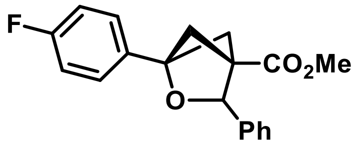

Following GP-1A the title compound was prepared by reaction of methyl 3-(4-fluorophenyl)bicyclo[1.1.0]butane-1-carboxylate (21.2 mg; 0.10 mmol), benzaldehyde (57.0 mg; 0.54 mmol), $LiBF_4$ (10.9 mg, 0.12 mmol) in a mixture of THF (4 mL) and DMF (0.2 mL); where the solution was electrolyzed at room temperature under alternating constant current conditions, 2 mA and 2 F/ mol (current alternation= 1 min) using glassy carbon anode and cathode. Column chromatography (SiO_2, EtOAc/ *n*-pentane 8: 92) afforded the desired product **3c** (18.8 mg, 0.060 mmol, 59%) as a colorless oil.

R_f (EtOAc/ *n*-pentane 1: 9) = 0.35.

^{1}H-NMR (500 MHz, $CDCl_3$): δ= 7.50 – 7.46 (m, 2H), 7.45 – 7.42 (m, 2H), 7.37 – 7.29 (m, 2H), 7.32 – 7.27 (m, 1H), 7.14 – 7.06 (m, 2H), 5.50 (s, 1H), 3.74 (s, 3H), 2.58 (ddd, *J* = 10.2, 7.1, 1.0 Hz, 1H), 2.52 (d, *J* = 6.3 Hz, 1H), 2.29 (ddd, *J* = 10.2, 8.1, 0.8 Hz, 1H), 2.21 (d, *J* = 8.1 Hz, 1H).

^{13}C NMR (126 MHz, $CDCl_3$) δ 170.5, 162.8 (d, *J* = 246.9 Hz), 139.5, 133.1 (d, *J* = 3.2 Hz), 128.3, 128.2 (d, *J* = 8.2 Hz), 127.9, 126.6, 115.5 (d, *J* = 21.7 Hz), 86.4, 80.5, 54.7, 51.9, 51.11, 40.1.

^{19}F NMR (471 MHz, $CDCl_3$) δ -113.43.

IR (ATR): $\tilde{\nu}$ (cm^{-1})= 2954, 1732, 1516, 1451, 1436, 1359, 1315, 1250, 1221, 1200, 1157, 1125, 1111, 1010, 974, 915, 834, 813, 736, 702, 598.

HRMS (ESI) m/z: $[M+H]^+$ calculated for $C_{19}H_{17}FO_3Na$, 335.1054; found: 335.1055.

Methyl 3-pentyl-1-phenyl-2-oxabicyclo[2.1.1]hexane-4-carboxylate (3d)

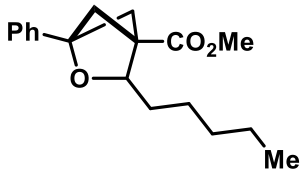

Following GP-1B the title compound was prepared by reaction of methyl 3-phenylbicyclo[1.1.0]butane-1-carboxylate (17.8; 0.10 mmol), hexanal (68.8 mg; 0.69 mmol), $LiBF_4$ (10.7 mg, 0.11 mmol) in CH_2Cl_2 (4 mL) and DMF (0.2 mL); where the solution was electrolyzed at room temperature under alternative current conditions, 10 mA and 2 F/ mol (current alternation= 1 min) using glassy carbon anode and cathode. Column chromatography (SiO_2, EtOAc/ *n*-pentane 4:96) afforded the desired product **3d** (11.8 mg, 0.041 mmol, 41% y) as a colorless oil. Spectral data matches literature reports. [3]

R_f (EtOAc/ *n*-pentane 4:96) = 0.43.

^{1}H-NMR (300 MHz, $CDCl_3$): δ 7.41 – 7.38 (m, 2H), 7.37 – 7.34 (m, 2H), 7.32 – 7.29 (m, 1H), 4.28 (dd, *J* = 8.7, 4.1 Hz, 1H), 3.76 (s, 3H), 2.39 (d, *J* = 7.0 Hz, 1H), 2.33 (ddd, *J* = 10.2, 7.0, 0.9 Hz, 1H), 2.28 (dt, *J* = 7.6, 0.8 Hz, 1H), 2.22 (dd, *J* = 10.0, 7.5 Hz, 1H), 1.72 – 1.64 (m, 1H), 1.62 – 1.55 (m, 2H), 1.43 – 1.30 (m, 5H), 0.90 (t, *J* = 7.2 Hz, 3H).

^{13}C NMR (176 MHz, $CDCl_3$): δ = 171.2, 137.4, 128.5, 128.3, 126.2, 86.4, 80.6, 52.8, 51.9, 50.1, 41.8, 33.2, 32.2, 25.9, 22.7, 14.2.

IR (ATR): $\tilde{\nu}$ (cm^{-1}) = 2941, 2859, 1735, 1440, 1358, 1265, 1197, 1110, 1009, 904, 793, 754, 697.

HRMS (ESI) m/z: $[M+H]^+$ calculated for $C_{18}H_{25}O_3$, 289.1798; found: 289.1797.

Methyl 3-(perfluorophenyl)-1-phenyl-2-oxabicyclo[2.1.1]hexane-4-carboxylate (3e)

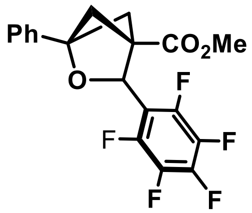

Following GP-1B the title compound was prepared by reaction of methyl 3-phenylbicyclo[1.1.0]butane-1-carboxylate (20.8 mg; 0.11 mmol), pentafluorobenzaldehyde (94.6 mg; 0.48 mmol), $LiBF_4$ (13.6 mg, 0.14 mmol) in a mixture of CH_2Cl_2: DMF (4: 0.2 mL); where the solution was electrolyzed at room temperature under alternating current conditions, 5 mA and 2 F/ mol (current alternation= 1 min) using glassy carbon anode and cathode. Column chromatography (SiO_2, Et_2O: CH_2Cl_2: *n*-pentane, 6: 6: 88) afforded the desired product **3e** in 26% yield (11.1 mg, 0.029 mmol) as a colorless oil.

Due to complex ^{19}F-^{13}C splitting observed in ^{13}C, both coupled and decoupled spectra attached for clarity.

R_f (SiO_2, Et_2O: CH_2Cl_2: *n*-pentane, 8: 8: 84)= 0.21.

^{1}H-NMR (400 MHz, $CDCl_3$): δ 7.48 – 7.32 (m, 5H), 5.57 (s, 1H), 3.76 (s, 3H), 2.59 (d, *J* = 7.4 Hz, 1H), 2.54 (d, *J* = 8.3 Hz, 1H), 2.50 (ddd, *J* = 10.5, 7.5, 1.0 Hz, 1H), 2.35 (ddd, *J* = 10.5, 8.3, 1.0 Hz, 1H).

^{13}C NMR (126 MHz, $CDCl_3$) δ 169.6, 145.3, 141.1, 137.8, 136.0, 128.8, 128.6, 126.3, 112.7, 88.0, 74.1, 54.0, 52.4, 49.3, 43.9.

^{19}F NMR (471 MHz, $CDCl_3$) δ -140.2 (dd, *J* = 20.2, 7.0 Hz, 2F), -154.1 (t, *J* = 20.1, 1F), -161.82 (dd, *J* = 20.9, 14.6 Hz).

IR (ATR): $\tilde{\nu}$ (cm^{-1})= 2966, 1737, 1524, 1498, 1438, 1362, 1321, 1309, 1255, 1202, 1149, 1120, 1011, 996, 988, 964, 940, 929, 878, 755, 697.

HRMS (ESI) m/z: $[M+NH_4]^+$ calculated for $C_{19}H_{17}F_5NO_3$, 402.1123; found: 402.1119.

Ethyl 1,3-diphenyl-2-oxabicyclo[2.1.1]hexane-4-carboxylate (3f)

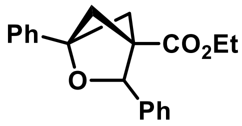

Following GP-1B the title compound was prepared by reaction of ethyl 3-phenylbicyclo[1.1.0]butane-1-carboxylate (21.8 mg; 0.11 mmol), benzaldehyde (60.9 mg; 0.57 mmol), $LiBF_4$ (14.5 mg, 0.14 mmol) in a mixture of CH_2Cl_2: DMF (4: 0.2 mL); where the solution was electrolyzed at room temperature under alternating current conditions, 5 mA and 2 F/ mol (current alternation= 1 min) using glassy carbon anode and cathode. Column chromatography (SiO_2, EtOAc: *n*-pentane, 2: 97 to 5: 95) afforded the desired product **3f** (14.4 mg, 0.47 mmol, 43% yield) as a colorless oil.

R_f (SiO_2, EtOAc: *n*-pentane, 5: 95)= 0.21.

^{1}H-NMR (400 MHz, $CDCl_3$) δ 7.52 (dd, *J* = 8.3, 1.3 Hz, 2H), 7.49 – 7.45 (m, 2H), 7.43 – 7.40 (m, 2H), 7.37 – 7.33 (m, 3H), 7.31 – 7.26 (m, 1H), 5.50 (s, 1H), 4.24 – 4.17 (m, 2H), 2.59 (ddd, *J* = 10.3, 7.0, 1.0 Hz, 1H), 2.54 (d, *J* = 7.0 Hz, 1H), 2.31 (ddd, *J* = 10.5, 8.2, 0.8 Hz, 1H), 2.22 (d, *J* = 8.1 Hz, 1H), 1.27 (t, *J* = 7.1 Hz, 3H).

^{13}C NMR (176 MHz, $CDCl_3$) δ 170.2, 139.7, 137.2, 128.6, 128.5, 128.2, 127.8, 126.7, 126.3, 86.9, 80.5, 60.9, 54.9, 51.0, 39.9, 14.3.

IR (ATR): $\tilde{\nu}$ (cm^{-1}) = 3066, 1726, 1471, 1449, 1372, 1351, 1310, 1250, 1196, 1107, 1032, 1020, 1006, 996, 972, 933, 919, 754, 736, 698, 673.

HRMS (ESI) m/z: $[M+H]^+$ calculated for $C_{20}H_{21}O_3$, 309.1485; found: 309.1487.

(1,3-Diphenyl-2-oxabicyclo[2.1.1]hexan-4-yl)(phenyl)methanone (3g)

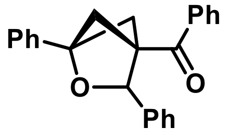

Following GP-1B the title compound was prepared by reaction of phenyl(3-phenylbicyclo[1.1.0]butan-1-yl)methanone (18.8 mg; 0.10 mmol), benzaldehyde (54.1 mg; 0.51 mmol), $LiBF_4$ (10.3 mg, 0.11 mmol) in a mixture of THF (4 mL) and DMF (0.2 mL); where the solution was electrolyzed at room temperature under alternating constant current conditions, 2 mA and 2 F/ mol (current alternation= 1 min) using glassy carbon anode and cathode. Column chromatography (SiO_2, EtOAc/ *n*-pentane 5:95) afforded the desired product **3g** (12.6 mg, 0.037 mmol, 32%) as a colorless solid. Following GP-1C the title compound was prepared in 45% yield (16.1 mg, 0.047 mmol). Spectral data matches literature reports. [2]

$\mathbf{R_f}$ (EtOAc/ *n*-pentane 7: 93) = 0.32.

^{1}**H-NMR** (300 MHz, $CDCl_3$): δ = 7.79 – 7.72 (m, 2H), 7.59 – 7.52 (m, 3H), 7.48 – 7.34 (m, 5H), 7.24 – 7.20 (m, 5H), 5.75 (s, 1H), 2.91 (ddd, *J* = 10.3, 7.4, 0.8 Hz, 1H), 2.69 (d, *J* = 7.4 Hz, 1H), 2.57 (dd, *J* = 10.2, 8.1 Hz, 1H), 2.31 (d, *J* = 8.1 Hz, 1H).

^{13}**C NMR** (176 MHz, $CDCl_3$) δ 198.7, 139.4, 137.1, 136.9, 133.4, 128.8, 128.7, 128.6, 128.5, 128.2, 127.9, 126.4, 126.3, 86.7, 82.3, 61.4, 52.4, 41.7.

IR (ATR): $\tilde{\nu}$ (cm^{-1})= 3061, 3029, 1665, 1598, 1448, 1353, 1317, 1301, 1252, 1205, 1177, 1003, 996, 968, 950, 933, 920, 848, 748, 714, 696, 673.

HRMS (ESI) m/z: $[M+Na]^+$ calculated for $C_{24}H_{20}O_2Na$, 363.1356; found: 363.1355.

(1-(4-Fluorophenyl)-3-phenyl-2-oxabicyclo[2.1.1]hexan-4-yl)(phenyl)methanone (3h)

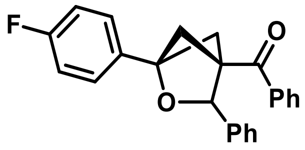

Following GP-1B the title compound was prepared by reaction of (3-(4-fluorophenyl)bicyclo[1.1.0]butan-1-yl)(phenyl)methanone (23.9 mg; 0.11 mmol), benzaldehyde (59.2 mg; 0.56 mmol), $LiBF_4$ (12.5 mg, 0.13 mmol) in a mixture of CH_2Cl_2: DMF (4: 0.2 mL); where the solution was electrolyzed at room temperature under alternating current conditions, 5 mA and 2 F/ mol (current alternation= 1 min) using glassy carbon anode and cathode. Column chromatography (SiO_2, Et_2O: CH_2Cl_2: *n*-pentane, 6: 6: 88) afforded the desired product **3h** in 32% yield (12.2 mg, 0.034 mmol) as a colorless oil.

R_f (SiO_2, Et_2O: CH_2Cl_2: *n*-pentane, 8: 8: 84)= 0.26.

^{1}H-NMR (400 MHz, $CDCl_3$): δ 7.74 (dd, *J* = 8.4, 1.3 Hz, 2H), 7.58 – 7.48 (m, 3H), 7.41 (dd, *J* = 8.2, 7.4 Hz, 2H), 7.26 – 7.18 (m, 5H), 7.15 – 7.07 (m, 2H), 5.74 (s, 1H), 2.92 – 2.86 (m, 1H), 2.66 (d, *J* = 7.3 Hz, 1H), 2.55 (dd, *J* = 10.3, 8.1 Hz, 1H), 2.29 (d, *J* = 8.1 Hz, 1H).

^{13}C NMR (126 MHz, $CDCl_3$) δ 198.5, 163.8, 161.9, 139.2, 136.8, 133.4, 133.1 (d, *J* = 3.0 Hz), 128.9, 128.8 (d, *J* = 13.3 Hz), 128.7, 128.3, 128.2(4), 128.1(9), 127.9, 126.37, 126.29, 115.62, 115.45, 86.1, 82.3, 61.4, 52.4, 41.7.

^{19}F NMR (282 MHz, $CDCl_3$) δ -113.43.

IR (ATR): $\tilde{\nu}$ (cm^{-1}) = 2952, 1665, 1599, 1580, 1516, 1449, 1356, 1306, 1223, 1179, 1158, 1001, 969, 918, 834, 812, 748, 719, 699, 671, 597.

HRMS (ESI) m/z: $[M+Na]^+$ calculated for $C_{24}H_{19}FO_2Na$, 381.1261; found: 381.1261.

(1,3-Diphenyl-2-oxabicyclo[2.1.1]hexan-4-yl)(naphthalen-2-yl)methanone (3i)

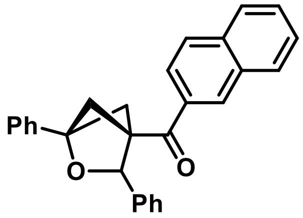

Following GP-1B the title compound was prepared by reaction of naphthalen-2-yl(3-phenylbicyclo[1.1.0]butan-1-yl)methanone (28.8 mg; 0.10 mmol), benzaldehyde (56.5 mg; 0.53 mmol), $LiBF_4$ (11.9 mg, 0.13 mmol) in a mixture of CH_2Cl_2: DMF (4: 0.2 mL); where the solution was electrolyzed at room temperature under alternating current conditions, 5 mA and 2 F/ mol (current alternation= 1 min) using glassy carbon anode and cathode. Column chromatography (SiO_2, EtOAc: *n*-pentane, 6:94) afforded the desired product **3i** in 67% yield (26.4 mg, 0.67 mmol) as a colorless solid.

$\mathbf{R_f}$ (*n*-pentane/EtOAc 5:95) = 0.24.

^{1}H-NMR (400 MHz, $CDCl_3$): δ = 8.10 (s, 1H), 7.90 – 7.85 (m, 3H), 7.80 (dd, *J* = 8.2, 0.6 Hz, 1H), 7.25 – 7.19 (m, 3H), 7.54 (ddd, *J* = 8.1, 6.9, 1.2 Hz, 1H), 7.49 – 7.43 (m, 2H), 7.42 – 7.36 (m, 1H), 7.29 – 7.25 (m, 2H), 7.25 – 7.19 (m, 3H), 5.83 (s, 1H), 3.01 (ddd, *J* = 10.4, 7.2, 0.8 Hz, 1H), 2.75 (d, *J* = 7.2 Hz, 1H), 2.66 (dd, *J* = 10.2, 8.1 Hz, 1H), 2.37 (d, *J* = 8.1 Hz, 1H).

^{13}C-NMR (101 MHz, $CDCl_3$): δ = 198.5, 139.4, 137.1, 135.7, 134.1, 132.5, 130.7, 129.9, 128.9, 128.7, 128.6, 128.5, 128.2, 127.9(5), 127.9(1), 126.9, 126.4(2), 126.4(1), 124.3, 86.7, 82.4, 61.6, 52.5, 41.9.

IR (ATR): $\tilde{\nu}$ (cm^{-1}) = 3063, 1661, 1626, 1466, 1449, 1357, 1306, 1190, 1114, 1016, 1004, 968, 915, 864, 820, 792, 776, 756, 726, 698.

HRMS (ESI) m/z: $[M+Na]^+$ calculated for $C_{28}H_{22}O_2Na$, 413.1512; found: 413.1512.

(4-Chlorophenyl)(1,3-diphenyl-2-oxabicyclo[2.1.1]hexan-4-yl)methanone (3j)

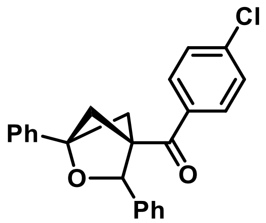

Following GP-1B the title compound was prepared by reaction of (4-chlorophenyl)(3-phenylbicyclo[1.1.0]butan-1-yl)methanone (27.2 mg; 0.10 mmol), benzaldehyde (52.2 mg; 0.49 mmol), $LiBF_4$ (11.4 mg, 0.12 mmol) in a mixture of CH_2Cl_2: DMF (4: 0.2 mL); where the solution was electrolyzed at room temperature under alternating current conditions, 5 mA and 2 F/ mol (current alternation= 1 min) using glassy carbon anode and cathode. Column chromatography (SiO_2, EtOAc: *n*-pentane, 6: 94) afforded the desired product **3j** in 42% yield (15.1 mg, 0.040 mmol) as a colorless oil.

$\mathbf{R_f}$ (SiO_2, EtOAc: *n*-pentane, 1: 9)= 0.58.

^{1}**H-NMR** (400 MHz, $CDCl_3$) δ 7.63 (d, *J* = 8.7 Hz, 2H), 7.46 – 7.41 (m, 2H), 7.46 – 7.41 (m, 2H), 7.40 – 7.35 (m, 3H), 7.26 – 7.21 (m, 5H). 5.70 (s, 1H), 2.88 (dd, *J* = 10.4, 7.3 Hz, 1H), 2.67 (d, *J* = 7.2 Hz, 1H), 2.58 (dd, *J* = 10.1, 8.1 Hz, 1H), 2.31 (d, *J* = 8.0 Hz, 1H).

^{13}**C NMR** (176 MHz, $CDCl_3$) δ 197.6, 139.8, 139.2, 136.9, 135.1, 130.1, 129.1, 128.6, 128.5, 128.3, 128.0, 126.4, 126.3, 86.7, 82.3, 61.3, 52.3, 41.8.

IR (ATR): $\tilde{\nu}$ (cm^{-1})= 3032, 1667, 1585, 1449, 1399, 1356, 1310, 1253, 1205, 1174, 1092, 1014, 1004, 996, 969, 934, 921, 845, 756, 737, 698

HRMS (ESI) m/z: $[M+Na]^+$ calculated for $C_{24}H_{19}ClO_2Na$, 397.0966; found: 397.0967.

(1,3-Diphenyl-2-oxabicyclo[2.1.1]hexan-4-yl)(4-(trifluoromethyl)phenyl)methanone (3k)

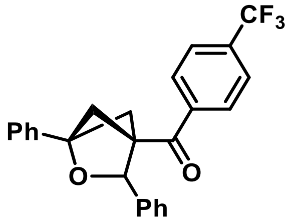

Following GP-1B the title compound was prepared by reaction of (3-phenylbicyclo[1.1.0]butan-1-yl)(4-(trifluoromethyl)phenyl)methanone (30.8 mg; 0.10 mmol), benzaldehyde (54.6 mg; 0.52 mmol), $LiBF_4$ (12.2 mg, 0.13 mmol) in a mixture of CH_2Cl_2: DMF (4: 0.2 mL); where the solution was electrolyzed at room temperature under alternating current conditions, 5 mA and 2 F/ mol (current alternation= 1 min) using glassy carbon anode and cathode. Column chromatography (SiO_2, EtOAc: *n*-pentane, 6: 94) afforded the desired product **3k** in 35% yield (14.6 mg, 0.036 mmol) as a colorless oil. Spectral data matches literature reports. [2]

R_f (SiO_2, EtOAc: *n*-pentane, 1: 9)= 0.55.

^{1}H-NMR (400 MHz, $CDCl_3$) δ 7.75 (d, *J* = 8.8 Hz, 2H), 7.64 (d, *J* = 8.2 Hz, 2H), 7.56 – 7.53 (m, 2H), 7.46 – 7.42 (m, 2H), 7.40 – 7.36 (m, 1H), 7.26 – 7.22 (m, 5H), 5.72 (s, 1H), 2.90 (ddd, *J* = 10.3, 7.2, 0.8 Hz, 1H), 2.68 (d, *J* = 7.2 Hz, 1H), 2.61 (dd, *J* = 10.3, 8.0 Hz, 1H), 2.32 (d, *J* = 8.0 Hz, 1H).

^{13}C NMR (176 MHz, $CDCl_3$) δ 198.1, 139.5, 139.1, 136.8, 134.5 (q, *J* = 32.7 Hz), 128.9, 128.7, 128.6, 128.4, 128.2, 126.4, 126.3, 125.8 (q, *J* = 3.7 Hz), 123.6 (q, *J* = 272.8 Hz), 86.9, 82.3, 61.4, 52.3, 41.9.

^{19}F NMR (659 MHz, $CDCl_3$) δ -63.21.

IR (ATR): $\tilde{\nu}$ (cm^{-1})= 2922, 2849, 1673, 1409, 1357, 1325, 1312, 1253, 1205, 1169, 1129, 1066, 1016, 1004, 969, 921, 854, 772, 756, 745, 711, 697

HRMS (ESI) m/z: $[M+H]^+$ calculated for $C_{25}H_{20}F_3O_2$, 409.1410; found: 409.1418.

(1,3-Diphenyl-2-oxabicyclo[2.1.1]hexan-4-yl)(p-tolyl)methanone (3l)

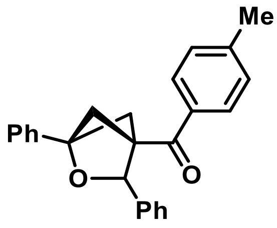

Following GP-1B the title compound was prepared by reaction of (3-phenylbicyclo[1.1.0]butan-1-yl)(p-tolyl)methanone (28.8 mg; 0.12 mmol), benzaldehyde (56.5 mg; 0.53 mmol), $LiBF_4$ (11.9 mg, 0.13 mmol) in a mixture of CH_2Cl_2: DMF (4: 0.2 mL); where the solution was electrolyzed at room temperature under alternating current conditions, 5 mA and 2 F/ mol (current alternation= 1 min) using glassy carbon anode and cathode. Column chromatography (SiO_2, EtOAc: *n*-pentane, 6:94) afforded the desired product **3l** in 48% yield (21.3 mg, 0.06 mmol) as a colorless oil.

$\mathbf{R_f}$ (*n*-pentane/EtOAc 1:20) = 0.22.

^{1}H-NMR (400 MHz, $CDCl_3$): δ = 7.67 (d, *J* = 8.2 Hz, 2H), 7.56 – 7.53 (m, 2H), 7.46 – 7.41 (m, 2H), 7.39 – 7.35 (m, 1H), 7.24 – 7.20 (m, 6H), 5.74 (s, 1H), 2.90 (ddd, *J* = 10.4, 7.2, 0.9 Hz, 1H), 2.67 (d, *J* = 7.2 Hz, 1H), 2.54 (dd, *J* = 10.3, 8.1 Hz, 1H), 2.41 (s, 3H), 2.29 (d, *J* = 8.1 Hz, 1H).

^{13}C-NMR (101 MHz, $CDCl_3$): δ = 198.1, 144.3, 139.5, 137.2, 134.3, 129.5, 128.9, 128.6, 128.5, 128.2, 127.8, 126.4, 126.3, 86.6, 82.3, 61.4, 52.4, 41.7, 21.8.

IR (ATR): $\tilde{\nu}$ (cm^{-1}) = 2927, 1661, 1605, 1494, 1449, 1355, 1309, 1253, 1205, 1179, 1017, 1004, 968, 920, 845, 789, 755, 734, 699, 613.

HRMS (ESI) m/z: $[M+Na]^+$ calculated for $C_{25}H_{22}O_2Na$, 377.1512; found: 377.1512.

(1-Phenyl-3-(p-tolyl)-2-oxabicyclo[2.1.1]hexan-4-yl)(p-tolyl)methanone (3m)

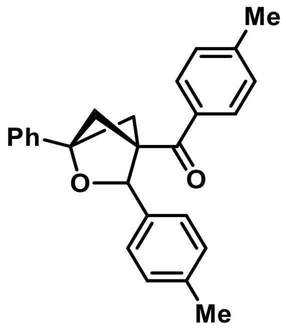

Following GP-1B the title compound was prepared by reaction of (3-phenylbicyclo[1.1.0]butan-1-yl)(p-tolyl)methanone (24.5 mg; 0.10 mmol), 4-methyl benzaldehyde (63.2 mg; 0.53 mmol), $LiBF_4$ (14.2 mg, 0.15 mmol) in a mixture of CH_2Cl_2: DMF (4: 0.2 mL); where the solution was electrolyzed at room temperature under alternating current conditions, 5 mA and 2 F/ mol (current alternation= 1 min) using glassy carbon anode and cathode. Column chromatography (SiO_2, EtOAc: *n*-pentane, 3: 97 to 5: 95) afforded the desired product **3m** in 41% yield (14.9 mg, 0.41 mmol) as a colorless solid.

R_f (SiO_2, EtOAc: *n*-pentane, 5: 95)= 0.21.

^{1}H-NMR (400 MHz, $CDCl_3$) δ 7.70 (d, *J* = 8.2 Hz, 2H), 7.54 (d, *J* = 7.0 Hz, 2H), 7.45 – 7.40 (m, 2H), 7.40 – 7.34 (m, 1H), 7.23 (d, *J* = 7.9 Hz, 2H), 7.10 (d, *J* = 8.0 Hz, 2H), 7.03 (d, *J* = 8.4 Hz, 2H), 5.72 (s, 1H), 2.89 (ddd, *J* = 10.4, 7.2, 0.9 Hz, 1H), 2.67 (d, *J* = 7.1 Hz, 1H), 2.53 (dd, *J* = 10.2, 8.0 Hz, 1H), 2.42 (s, 3H), 2.28 (s, 3H), 2.27 (d, *J* = 8.0 Hz, 1H).

^{13}C NMR (176 MHz, $CDCl_3$) δ 198.2, 144.2, 137.4, 137.3, 136.4, 134.4, 129.5, 128.9, 128.8, 128.6, 128.4, 126.4, 126.2, 86.5, 82.3, 61.5, 52.4, 41.6, 21.8, 21.2.

IR (ATR): $\tilde{\nu}$ (cm^{-1})= 3032, 2926, 1662, 1605, 1514, 1448, 1357, 1314, 1304, 1253, 1206, 1179, 1017, 1004, 968, 929, 846, 804, 770, 756, 747, 697.

HRMS (ESI) m/z: $[M+Na]^+$ calculated for $C_{26}H_{24}O_2Na$, 391.1669; found: 391.1669.

(3-(4-Methoxyphenyl)-1-phenyl-2-oxabicyclo[2.1.1]hexan-4-yl)(p-tolyl)methanone (3n)

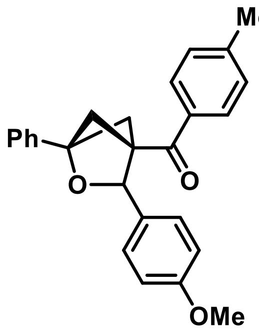

Following GP-1B the title compound was prepared by reaction of (3-phenylbicyclo[1.1.0]butan-1-yl)(p-tolyl)methanone (23.9 mg; 0.10 mmol), 4-methoxybenzaldehyde (70.2 mg; 0.52 mmol), $LiBF_4$ (10.9 mg, 0.12 mmol) in a mixture of CH_2Cl_2: DMF (4: 0.2 mL); where the solution was electrolyzed at room temperature under alternating current conditions, 5 mA and 2 F/ mol (current alternation= 1 min) using glassy carbon anode and cathode. Column chromatography (SiO_2, acetone: *n*-pentane, 4:96) afforded the desired product **3n** in 58% yield (21.4 mg, 0.055 mmol) as a colorless oil.

$\mathbf{R_f}$ (*n*-pentane/acetone 96:4) = 0.32.

^{1}H-NMR (400 MHz, $CDCl_3$): δ 7.68 (d, *J* = 8.2 Hz, 2H), 7.54 (dd, *J* = 8.3, 1.3 Hz, 2H), 7.44 – 7.41 (m, 2H), 7.39 – 7.33 (m, 1H), 7.25 – 7.20 (m, 2H), 7.14 (dd, *J* = 8.9, 0.7 Hz, 2H), 6.76 (d, *J* = 8.8 Hz, 2H), 5.70 (s, 1H), 3.75 (s, 3H), 2.88 (ddd, *J* = 10.3, 7.2, 0.8 Hz, 1H), 2.66 (d, *J* = 7.2 Hz, 1H), 2.53 (dd, *J* = 10.2, 8.0 Hz, 1H), 2.41 (s, 3H), 2.29 (d, *J* = 8.0 Hz, 1H).

^{13}C-NMR (101 MHz, $CDCl_3$): δ = 198.3, 159.3, 144.3, 137.3, 134.5, 131.6, 129.6, 128.9, 128.6, 128.4, 127.5, 126.4, 113.7, 86.5, 82.1, 61.5, 55.4, 52.4, 41.7, 21.9.

IR (ATR): $\tilde{\nu}$ (cm^{-1}) = 2930, 1661, 1606, 1512, 1455, 1355, 1302, 1251, 1177, 1032, 967, 845, 811, 752, 698.

HRMS (ESI) m/z: $[M+Na]^+$ calculated for $C_{26}H_{24}O_3Na$, 407.1618; found: 407.1617.

1-(1,3-Diphenyl-2-oxabicyclo[2.1.1]hexan-4-yl)ethan-1-one (3o)

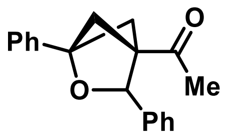

Following GP-1B the title compound was prepared by reaction of 1-(3-phenylbicyclo[1.1.0]butan-1-yl)ethan-1-one (17.8 mg; 0.10 mmol), benzaldehyde (65.0 mg; 0.61 mmol), $LiBF_4$ (16.6 mg, 0.18 mmol) in a mixture of CH_2Cl_2: DMF (4: 0.2 mL); where the solution was electrolyzed at room temperature under alternating current conditions, 5 mA and 2 F/ mol (current alternation= 1 min) using glassy carbon anode and cathode. Column chromatography (SiO_2, Et_2O: CH_2Cl_2: *n*-pentane, 6: 6: 88) afforded the desired product **3o** (16.6 mg, 0.06 mmol, 58% yield) as a colorless oil. Spectral data matches literature reports.[2]

R_f (SiO_2, EtOAc: *n*-pentane, 1: 9)= 0.28.

^{1}H-NMR (400 MHz, $CDCl_3$): δ 7.54 – 7.49 (m, 2H), 7.45 – 7.39 (m, 4H), 7.39 – 7.33 (m, 3H), 7.32 – 7.27 (m, 1H), 5.49 (s, 1H), 2.61 (ddd, *J* = 10.3, 7.2, 0.9 Hz, 1H), 2.47 (d, *J* = 7.2 Hz, 1H), 2.39 (dd, *J* = 10.3, 8.0 Hz, 1H), 2.16 (d, *J* = 7.9 Hz, 1H), 2.09 (s, 3H).

^{13}C NMR (126 MHz, $CDCl_3$) δ 206.8, 139.5, 137.0, 128.6, 128.5, 128.4, 128.0, 126.6, 126.3, 86.5, 81.0, 61.8, 50.7, 40.2, 28.4.

IR (ATR): $\tilde{\nu}$ (cm^{-1})= 3065, 3002, 2954, 2884, 1701, 1495, 1470, 1449, 1428, 1362, 1247, 1190, 1177, 1089, 1019, 997, 974, 954, 932, 915, 852, 751, 698, 677.

HRMS (ESI) m/z: $[M+Na]^+$ calculated for $C_{19}H_{18}O_2Na$, 301.1199; found: 301.1201.

1-(1,3-Diphenyl-2-oxabicyclo[2.1.1]hexan-4-yl)pentan-1-one (3p)

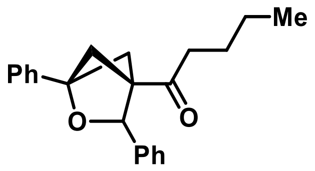

Following GP-1B the title compound was prepared by reaction of 1-(3-phenylbicyclo[1.1.0]butan-1-yl)pentan-1-one (24.2 mg; 0.11 mmol), benzaldehyde (55.1 mg; 0.52 mmol), $LiBF_4$ (13.7 mg, 0.15 mmol) in a mixture of CH_2Cl_2: DMF (4: 0.2 mL); where the solution was electrolyzed at room temperature under alternative current conditions, 5 mA and 2 F/ mol (current alternation= 1 min) using glassy carbon anode and cathode. Column chromatography (SiO_2, EtOAc: *n*-pentane, 6:94 – 12:82) afforded the desired product **3p** in 45% yield (16.3 mg, 0.05 mmol) as a colorless oil.

$\mathbf{R_f}$ (EtOAc/ *n*-pentane, 1:9) = 0.44.

^{1}H-NMR (700 MHz, $CDCl_3$): δ 7.51 (dd, *J* = 8.3, 1.3 Hz, 2H), 7.44 – 7.33 (m, 7H), 7.30 – 7.27 (m, 1H), 5.49 (s, 1H), 2.61 (ddd, *J* = 10.4, 7.2, 0.9 Hz, 1H), 2.46 – 2.37 (m, 3H), 2.22 – 2.17 (m, 1H), 2.16 (dd, *J* = 8.2, 0.8 Hz, 1H), 1.55 – 1.46 (m, 2H), 1.24 (h, *J* = 7.5 Hz, 2H), 0.85 (t, *J* = 7.4 Hz, 3H).

^{13}C NMR (176 MHz, $CDCl_3$): δ = 209.0, 139.6, 137.2, 128.6, 128.5, 128.3, 127.9, 126.7, 126.3, 86.6, 81.1, 61.4, 50.8, 40.7, 40.1, 25.1, 22.3, 13.9.

IR (ATR): $\tilde{\nu}$ (cm^{-1}) = 3031, 2956, 2931, 2872, 1699, 1495, 1466, 1449, 1361, 1250, 1175, 1126, 1047, 1027, 971, 931, 853, 752, 698, 675.

HRMS (ESI) m/z: $[M+Na]^+$ calculated for $C_{22}H_{24}O_2Na$, 343.1669; found: 343.1669.

6.2. Arylation products

Methyl 3-(4-methoxyphenyl)-3-phenylcyclobutane-1-carboxylate (5a)

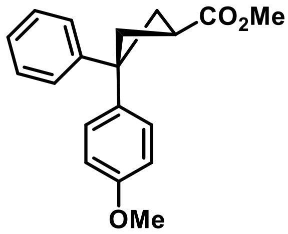

Following GP-2 the title compound was prepared by reaction of methyl 3-phenylbicyclo[1.1.0]butane-1-carboxylate (18.8 mg; 0.10 mmol), anisole (108.1 mg, 110 µL; 1.0 mmol), $TBABF_4$ (20.5 mg, 0.06 mmol, 0.02 M) in HFIP (3 mL); where the solution was electrolyzed at room temperature under alternating constant current conditions, 2 mA and 1.5 F/ mol (current alternation= 1 min) using glassy carbon anode and cathode. Column chromatography (SiO_2, EtOAc/ *n*-pentane 1: 20) afforded the desired product **5a** (21.6 mg, 0.073 mmol, 73%, *p*:*o* 6:1) as a colorless oil.

R_f (EtOAc/ *n*-pentane 1: 20) = 0.38.

Analytical data were recorded of the pure p-substituted regioisomer **5a**. Pure p-substituted regioisomer was isolated as mixture of diastereomers in 3.6:1 dr.

^{1}H-NMR (700 MHz, $CDCl_3$): Major diastereomer δ = 7.41 – 7.37 (m, 2H), 7.33 – 7.29 (m, 2H), 7.20 – 7.17 (m, 1H), 7.10 – 7.07 (m, 2H), 6.81 – 6.77 (m, 2H), 3.75 (s, 3H), 3.66 (s, 3H), 3.16 – 3.09 (m, 1H), 3.03 – 2.92 (m, 4H). Minor diastereomer δ = 7.31 – 7.29 (m, 2H), 7.25 – 7.23 (m, 2H), 7.16 – 7.13 (m, 2H), 7.13 – 7.10 (m, 1H), 6.86 – 6.83 (m, 2H), 3.78 (s, 3H), 3.66 (s, 3H), 3.16 – 3.12 (m, 1H), 3.02 – 2.92 (m, 4H).

^{13}C NMR (176 MHz, $CDCl_3$): Major diastereomer δ= 175.4, 157.7, 147.4, 142.2, 128.5, 126.9, 126.6, 126.1, 113.8, 55.3, 51.8, 46.9, 38.2, 32.9. Minor diastereomer δ = 175.4, 157.9, 150.2, 139.1, 128.4, 127.7, 125.7, 125.6, 55.3, 51.8, 46.8, 38.1, 33.0.

IR (ATR): $\tilde{\nu}$ (cm^{-1}) = 3057, 3023, 2989, 2949, 2907, 2835, 1730, 1610, 1580, 1511, 1494, 1463, 1435, 1362, 1246, 1196, 1176, 1155, 1117, 1080, 1053, 1033.

HRMS (APCI+) m/z: $[M+NH_4]^+$ Calculated for $C_{19}H_{24}NO_3^+$ 314,1751; Found: 314.1749.

Methyl 3-(4-fluorophenyl)-3-(4-methoxyphenyl)cyclobutane-1-carboxylate (5b)

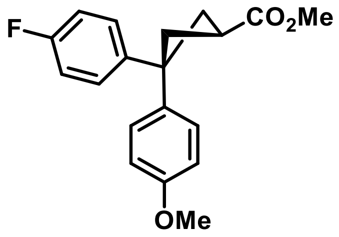

Following GP-2 the title compound was prepared by reaction of methyl 3-(4-fluorophenyl)bicyclo[1.1.0]butane-1-carboxylate (20.6 mg; 0.10 mmol), anisole (108.1 mg, 110 µL; 1.0 mmol), $TBABF_4$ (20.5 mg, 0.06 mmol, 0.02 M) in HFIP (3 mL); where the solution was electrolyzed at room temperature under alternating constant current conditions, 2 mA and 1.5 F/ mol (current alternation= 1 min) using glassy carbon anode and cathode. Column chromatography (SiO_2, EtOAc/ *n*-pentane 1: 20) afforded the desired product **5b** (24.1 mg, 0.077 mmol, 77%, *p*:*o* 4:1) as a colorless oil.

R_f (EtOAc/ *n*-pentane 1: 20) = 0.35.

Analytical data were recorded of the pure p-substituted regioisomer **5b**. Pure p-substituted regioisomer was isolated as mixture of diastereomers in 3.5:1 dr.

^{1}H-NMR (500 MHz, $CDCl_3$): Major diastereomer δ = 7.36 – 7.31 (m, 2H), 7.08 – 7.03 (m, 2H), 7.01 – 6.95 (m, 2H), 6.82 – 6.77 (m, 2H), 3.75 (s, 3H), 3.66 (s, 3H), 3.14 – 3.07 (m, 1H), 2.98 – 2.88 (m, 4H). Minor diastereomer δ = 7.30 – 7.27 (m, 2H), 7.12 – 7.09 (m, 2H), 6.95 – 6.90 (m, 2H), 6.87 – 6.84 (m, 2H), 3.78 (s, 3H), 3.66 (s, 3H), 3.15 – 3.06 (m, 1H), 2.99 – 2.88 (m, 4H).

^{13}C NMR (126 MHz, $CDCl_3$): Major diastereomer δ= 175.3, 161.2 (d, J = 244.7 Hz), 157.7, 143.1 (d, J = 3.3 Hz), 141.9, 128.2 (d, J = 8.2 Hz), 126.8, 115.3 (d, J = 21.3 Hz). 113.8, 55.3, 51.8, 46.5, 38.3, 32.8. Minor diastereomer δ = 175.4, 161.0 (d, J = 244.1 Hz), 157.9, 145.9 (d, J = 3.3 Hz), 138.8, 127.6, 127.3 (d, J = 8.2 Hz), 115.1 (d, J = 19.6 Hz), 113.9, 55.3, 51.8, 46.3, 38.2, 32.9.

^{19}F NMR (471 MHz, $CDCl_3$): Major diastereomer δ= 117.3. Minor diastereomer δ = -117.7.

IR (ATR): $\tilde{\nu}$ (cm^{-1}) = 2990, 2950, 2906, 2836, 1729, 1608, 1507, 1463, 1435, 1363, 1301, 1246, 1222, 1196, 1177, 1163, 1116, 1053, 1034.

HRMS (ESI+) m/z: $[M+Na]^+$ Calculated for $C_{19}H_{19}FO_3Na$ 337.1210; Found: 337.1212.

Methyl 3-(4-chlorophenyl)-3-(4-methoxyphenyl)cycloutane-1-carboxylate (5c)

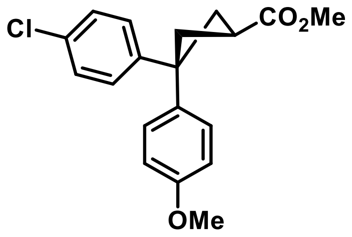

Following GP-2 the title compound was prepared by reaction of methyl 3-(4-chlorophenyl)bicyclo[1.1.0]butane-1-carboxylate (22.3 mg; 0.10 mmol), anisole (108.1 mg, 110 µL; 1.0 mmol), $TBABF_4$ (20.5 mg, 0.06 mmol, 0.02 M) in HFIP (3 mL); where the solution was electrolyzed at room temperature under alternating constant current conditions, 2 mA and 1.5 F/ mol (current alternation= 1 min) using glassy carbon anode and cathode. Column chromatography (SiO_2, EtOAc/ *n*-pentane 1: 20) afforded the desired product **5c** (24.1 mg, 0.079 mmol, 79%, *p*:*o* 10:1) as a colorless oil.

$\mathbf{R_f}$ (EtOAc/ *n*-pentane 1: 20) = 0.35.

Analytical data were recorded of the pure *p*-substituted regioisomer **5c**. Pure *p*-substituted regioisomer was isolated as mixture of diastereomers in 2.3: 1 dr.

^{1}H-NMR (500 MHz, $CDCl_3$): Major diastereomer δ = 7.33 – 7.25 (m, 4H), 7.07 – 7.03 (m, 2H), 6.82 – 6.77 (m, 2H), 3.75 (s, 3H), 3.66 (s, 3H), 3.15 – 3.06 (m, 1H), 2.98 – 2.88 (m, 4H). Minor diastereomer δ = 7.33 – 7.25 (m, 2H), 7.23 – 7.19 (m, 2H), 7.10 – 7.07 (m, 2H), 6.88 – 6.84 (m, 2H), 3.78 (s, 3H), 3.66 (s, 3H), 3.14 – 3.05 (m, 1H), 3.00 – 2.87 (m, 4H).

^{13}C NMR (126 MHz, $CDCl_3$): Major diastereomer δ= 175.2, 157.8, 145.9, 141.6, 131.9, 128.7, 128.0, 126.8, 113.9, 55.3, 51.9, 46.6, 38.1, 32.8. Minor diastereomer δ = 175.3, 158.0, 148.7, 138.5, 131.6, 128.5, 127.6, 127.2, 114.0, 55.4, 51.9, 46.3, 38.0, 32.9.

IR (ATR): $\tilde{\nu}$ (cm^{-1}) = 2990, 2950, 2906, 2835, 1730, 1609, 1581, 1511, 1490, 1462, 1435, 1397, 1363, 1246, 1197, 1176, 1155, 1122, 1094, 1053, 1034, 1012.

HRMS (ESI+) m/z: $[M+Na]^+$ Calculated for $C_{19}H_{19}ClO_3Na$ 353.0915; Found: 353.0916.

Methyl 3-(4-bromophenyl)-3-(4-methoxyphenyl)cyclobutane-1-carboxylate (5d)

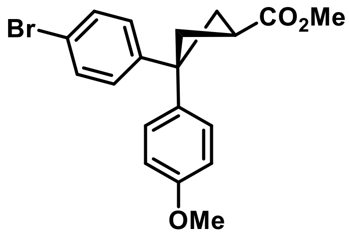

Following GP-2 the title compound was prepared by reaction of methyl 3-(4-bromophenyl)bicyclo[1.1.0]butane-1-carboxylate (26.7 mg; 0.10 mmol), anisole (108.1 mg, 110 µL; 1.0 mmol), $TBABF_4$ (20.5 mg, 0.06 mmol, 0.02 M) in HFIP (3 mL); where the solution was electrolyzed at room temperature under alternating constant current conditions, 2 mA and 1.5 F/ mol (current alternation= 1 min) using glassy carbon anode and cathode. Column chromatography (SiO_2, EtOAc/ *n*-pentane 1: 20) afforded the desired product **5d** (29.0 mg, 0.077 mmol, 77%, *p*:*o* 5:1) as a colorless oil.

$\mathbf{R_f}$ (EtOAc/ *n*-pentane 1: 20) = 0.31.

Analytical data were recorded of the pure *p*-substituted regioisomer **5d**. Pure *p*-substituted regioisomer was isolated as mixture of diastereomers in 2.5:1 dr.

^{1}H-NMR (500 MHz, $CDCl_3$): Major diastereomer δ = 7.44 – 7.41 (m, 2H), 7.29 – 7.23 (m, 2H), 7.08 – 7.01 (m, 2H), 6.82 – 6.77 (m, 2H), 3.75 (s, 3H), 3.66 (s, 3H), 3.16 – 3.05 (m, 1H), 2.99 – 2.87 (m, 4H). Minor diastereomer δ = 7.38 – 7.34 (m, 2H), 7.29 – 7.26 (m, 2H), 7.04 – 7.02 (m, 2H), 6.87 – 6.83 (m, 2H), 3.78 (s, 3H), 3.66 (s, 3H), 3.18 – 3.08 (m, 1H), 3.00 – 2.88 (m, 4H).

^{13}C NMR (126 MHz, $CDCl_3$): Major diastereomer δ= 175.2, 157.8, 146.6, 141.5, 131.6, 128.4, 126.8, 119.9, 113.9, 55.3, 51.9, 46.6, 38.1, 32.8. Minor diastereomer δ = 13C NMR (126 MHz, CDCl3) δ 175.3, 158.1, 149.2, 138.4, 131.5, 127.6, 127.5, 119.6, 114.0, 55.4, 51.9, 46.4, 37.9, 32.9.

IR (ATR): $\tilde{\nu}$ (cm^{-1}) = 2995, 2950, 2906, 2835, 1731, 1597, 1584, 1488, 1462, 1435, 1364, 1245, 1210, 1194, 1177, 1154, 1126, 1073, 1060, 1045, 1030, 1008.

HRMS (ESI+) m/z: $[M+Na]^+$ Calculated for $C_{19}H_{19}BrO_3Na$ 397.0410; Found: 397.0410.

Methyl 3-(4-methoxyphenyl)-3-(4-(trifluoromethyl)phenyl)cyclobutane-1-carboxylate (5e)

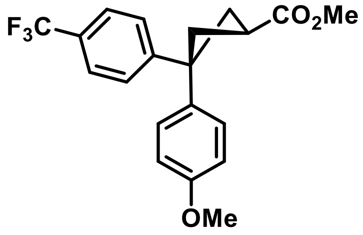

Following GP-2 the title compound was prepared by reaction of methyl 3-(4-(trifluoromethyl)phenyl)bicyclo[1.1.0]butane-1-carboxylate (25.6 mg; 0.10 mmol), anisole (108.1 mg, 110 µL; 1.0 mmol), $TBABF_4$ (20.5 mg, 0.06 mmol, 0.02 M) in HFIP (3 mL); where the solution was electrolyzed at room temperature under alternating constant current conditions, 2 mA and 1.5 F/ mol (current alternation= 1 min) using glassy carbon anode and cathode. Column chromatography (SiO_2, EtOAc/ *n*-pentane 1: 20) afforded the desired product **5e** (14.0 mg, 0.038 mmol, 38%, *p*:*o* 18:1) as a colorless oil.

$\mathbf{R_f}$ (EtOAc/ *n*-pentane 1: 20) = 0.28.

Analytical data were recorded of the pure *p*-substituted regioisomer **5e**. Pure *p*-substituted regioisomer was isolated as mixture of diastereomers in 1.1:1 dr.

^{1}H-NMR (500 MHz, $CDCl_3$): Both diastereomers δ = 7.59 – 7.54 (m, 2H), 7.53 – 7.48 (m, 4H), 7.32 – 7.26 (m, 4H), 7.11 – 7.06 (m, 2H), 6.90 – 6.85 (m, 2H), 6.83 – 6.79 (m, 2H), 3.78 (s, 3H), 3.76 (s, 3H), 3.66 (s, 6H), 3.18 – 3.06 (m, 2H), 3.04 – 2.91 (m, 8H).

^{13}C NMR (126 MHz, $CDCl_3$): Both diastereomers δ= 175.2, 175.0, 158.2, 157.9, 154.1, 151.6, 141.1, 138.0, 128.5, 128.4, 127.7, 126.9, 126.8, 126.0, 125.6, 125.5, 55.3(7), 55.3(5), 51.9, 47.1, 46.7, 38.1, 37.9, 32.9, 32.8.

^{19}F NMR (471 MHz, $CDCl_3$): Major diastereomer δ= -62.44. Minor diastereomer δ = -62.39.

IR (ATR): $\tilde{\nu}$ (cm^{-1}) = 2994, 2952, 2909, 2838, 1731, 1614, 1581, 1511, 1463, 1436, 1407, 1326, 1248, 1198, 1164, 1120, 1069, 1034, 1016.

HRMS (ESI+) m/z: $[M+H]^+$ Calculated for $C_{20}H_{20}F_3O_3$ 365.1359; Found: 365.1354.

Methyl 3-(4-methoxyphenyl)-3-(4-(trifluoromethoxy)phenyl)cyclobutane-1-carboxylate (5f)

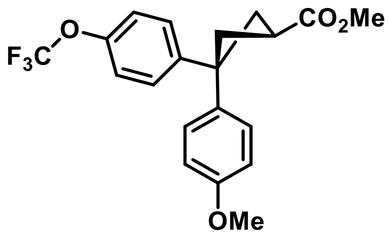

Following GP-2 the title compound was prepared by reaction of methyl 3-(4-(trifluoromethoxy)phenyl)bicyclo[1.1.0]butane-1-carboxylate (27.2 mg; 0.10 mmol), anisole (108.1 mg, 110 µL; 1.0 mmol), $TBABF_4$ (20.5 mg, 0.06 mmol, 0.02 M) in HFIP (3 mL); where the solution was electrolyzed at room temperature under alternating constant current conditions, 2 mA and 1.5 F/ mol (current alternation= 1 min) using glassy carbon anode and cathode. Column chromatography (SiO_2, EtOAc/ *n*-pentane 1: 20) afforded the desired product **5f** (28.0 mg, 0.074 mmol, 74%, *p*:*o* 6:1) as a colorless oil.

$\mathbf{R_f}$ (EtOAc/ *n*-pentane 1: 20) = 0.17.

Analytical data were recorded of the pure *p*-substituted regioisomer **5f**. Pure *p*-substituted regioisomer was isolated as mixture of diastereomers in 2.4:1 dr.

^{1}H-NMR (700 MHz, $CDCl_3$): Major diastereomer δ = 7.41 – 7.38 (m, 2H), 7.16 – 7.13 (m, 2H), 7.08 – 7.05 (m, 2H), 6.83 – 6.79 (m, 2H), 3.76 (s, 3H), 3.66 (s, 3H), 3.14 – 3.08 (m, 1H), 3.00 – 2.91 (m, 4H). Minor diastereomer δ = 7.31 – 7.28 (m, 2H), 7.18 – 7.16 (m, 2H), 7.10 – 7.08 (m, 2H), 6.88 – 6.85 (m, 2H), 3.78 (s, 3H), 3.66 (s, 3H), 3.15 – 3.09 (m, 1H), 3.00 – 2.90 (m, 4H).

^{13}C NMR (126 MHz, $CDCl_3$): Major diastereomer δ= 175.1, 157.8, 147.4 (q, *J* = 2.1 Hz), 146.2, 141.5, 127.9, 126.8, 120.9, 120.6 (q, *J* = 257.1 Hz), 113.9, 55.3, 51.9, 46.6, 38.2, 32.8. Minor diastereomer δ = 175.3, 158.1, 148.9, 147.2 (q, J = 1.9 Hz), 128.0, 127.7, 127.1, 120.9, 120.6 (q, *J* = 257.1 Hz), 114.1, 55.3(4), 55.3(3), 51.9, 46.6, 46.4, 38.2, 38.1, 32.9, 32.8.

^{19}F NMR (659 MHz, $CDCl_3$): Major diastereomer δ= -57.86. Minor diastereomer δ = -57.90.

IR (ATR): $\tilde{\nu}$ (cm^{-1}) = 2992, 2952, 2907, 2837, 1731, 1610, 1509, 1463, 1436, 1364, 1248, 1205, 1160, 1120, 1054, 1035, 1017.

HRMS (APCI+) m/z: $[M+H]^+$ Calculated for $C_{20}H_{20}F_3O_4^+$ 381.1264; Found: 381.1269.

Methyl 3-(3-methoxyphenyl)-3-(4-methoxyphenyl)cyclobutane-1-carboxylate (5g)

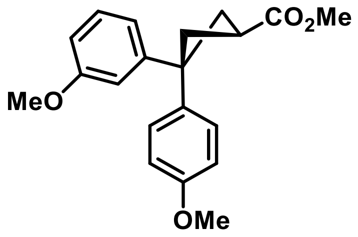

Following GP-2 the title compound was prepared by reaction of methyl 3-(3-methoxyphenyl)bicyclo[1.1.0]butane-1-carboxylate (21.8 mg; 0.10 mmol), anisole (108.1 mg, 110 µL; 1.0 mmol), $TBABF_4$ (20.5 mg, 0.06 mmol, 0.02 M) in HFIP (3 mL); where the solution was electrolyzed at room temperature under alternating constant current conditions, 2 mA and 1.5 F/ mol (current alternation= 1 min) using glassy carbon anode and cathode. Column chromatography (SiO_2, EtOAc/ *n*-pentane 1: 20) afforded the desired product **5g** (23.2 mg, 0.071 mmol, 71%, *p*:*o* 7:1) as a colorless oil.

$\mathbf{R_f}$ (EtOAc/ *n*-pentane 1: 20) = 0.12.

Analytical data were recorded of the pure *p*-substituted regioisomer **5g**. Pure *p*-substituted regioisomer was isolated as mixture of diastereomers in 2.4: 1 dr.

^{1}H-NMR (500 MHz, $CDCl_3$): Major diastereomer δ = 7.23 (t, J = 8.0 Hz, 1H), 7.11 – 7.07 (m, 2H), 6.98 (ddd, J = 8.0, 2.1, 0.9 Hz, 1H), 6.92 (t, J = 2.1 Hz, 1H), 6.81 – 6.77 (m, 2H), 6.72 (ddd, J = 8.0, 2.1, 0.9 Hz, 1H), 3.78 (s, 3H), 3.75 (s, 3H), 3.65 (s, 3H), 3.16 – 3.07 (m, 1H), 3.02 – 2.90 (m, 4H). Minor diastereomer δ = 7.34 – 7.30 (m, 2H), 7.17 (t, J = 8.0 Hz, 1H), 6.86 – 6.83 (m, 2H), 6.76 (ddd, J = 8.0, 1.7, 0.8 Hz, 1H), 6.70 – 6.68 (m, 1H), 6.66 (ddd, J = 8.0, 2.5, 0.8 Hz, 1H), 3.77 (s, 3H), 3.75 (s, 3H), 3.66 (s, 3H), 3.17 – 3.07 (m, 1H), 3.01 – 2.90 (m, 4H).

^{13}C NMR (126 MHz, $CDCl_3$): Major diastereomer δ= 175.4, 159.7, 157.7, 149.1, 142.0, 129.5, 126.9, 119.1, 113.8, 113.2, 110.7, 55.3(1), 55.2(5), 51.8, 46.9, 38.2, 32.9. Minor diastereomer δ = 175.4, 159.6, 157.9, 151.9, 138.9, 129.4, 127.7, 118.4, 113.9, 112.0, 110.6, 55.3, 55.2, 51.8, 46.7, 38.0, 32.9.

IR (ATR): $\tilde{\nu}$ (cm^{-1}) = 2993, 2950, 2908, 2835, 1730, 1607, 1581, 1511, 1484, 1463, 1434, 1362, 1287, 1246, 1202, 1176, 1151, 1035.

HRMS (ESI+) m/z: $[M+Na]^+$ Calculated for $C_{20}H_{22}O_4Na^+$ 349.1410; Found: 349.1411.

Methyl 3-(4-methoxyphenyl)-3-(m-tolyl)cyclobutane-1-carboxylate (5h)

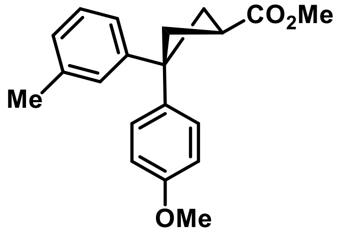

Following GP-2 the title compound was prepared by reaction of methyl 3-(m-tolyl)bicyclo[1.1.0]butane-1-carboxylate (20.2 mg; 0.10 mmol), anisole (108.1 mg, 110 µL; 1.0 mmol), $TBABF_4$ (20.5 mg, 0.06 mmol, 0.02 M) in HFIP (3 mL); where the solution was electrolyzed at room temperature under alternating constant current conditions, 2 mA and 1.5 F/ mol (current alternation= 1 min) using glassy carbon anode and cathode. Column chromatography (SiO_2, EtOAc/ *n*-pentane 1: 20) afforded the desired product **5h** (16.5 mg, 0.053 mmol, 53%, *p*:*o* 4:1) as a colorless oil.

$\mathbf{R_f}$ (EtOAc/ *n*-pentane 1: 20) = 0.41.

Analytical data were recorded of the pure *p*-substituted regioisomer **5h**. Pure *p*-substituted regioisomer was isolated as mixture of diastereomers in 2.1:1 dr.

^{1}H-NMR (500 MHz, $CDCl_3$): Major diastereomer δ = 7.22 – 7.17 (m, 2H), 7.11 – 7.07 (m, 2H), 7.01 – 6.97 (m, 1H), 6.97 – 6.91 (m, 1H), 6.81 – 6.76 (m, 2H), 3.75 (s, 3H), 3.65 (s, 3H), 3.15 – 3.07 (m, 1H), 3.02 – 2.88 (m, 4H), 2.33 (s, 3H).Minor diastereomer δ = 7.33 – 7.30 (m, 2H), 7.23 – 7.17 (m, 2H), 7.16 – 7.12 (m, 1H), 6.95 – 6.91 (m, 1H), 6.87 – 6.83 (m, 2H), 3.77 (s, 3H), 3.65 (s, 3H), 3.15 – 3.07 (m, 1H), 3.02 – 2.89 (m, 4H), 2.29 (s, 3H).

^{13}C NMR (126 MHz, $CDCl_3$): Major diastereomer δ= 175.5, 157.6, 147.3, 142.3, 138.0, 128.4, 127.7, 127.4, 126.9, 126.9, 123.6, 113.8, 55.3, 51.8, 46.8, 38.2, 32.9, 21.7. Minor diastereomer δ = 175.4, 157.8, 150.3, 139.1, 137.9, 128.3, 126.8(8), 126.8(5), 126.5, 126.3, 122.7, 113.9, 55.3, 51.8, 46.7, 38.1, 33.1, 21.6.

IR (ATR): $\tilde{\nu}$ (cm^{-1}) = 3028, 2988, 2949, 2908, 2835, 1731, 1608, 1581, 1510, 1462, 1435, 1362, 1247, 1201, 1177, 1152, 1115, 1053, 1034.

HRMS (ESI+) m/z: $[M+Na]^+$ Calculated for $C_{20}H_{22}O_3Na$ 333.1461; Found: 333.1462.

Methyl 3-(4-methoxyphenyl)-3-(naphthalen-2-yl)cyclobutane-1-carboxylate (5i)

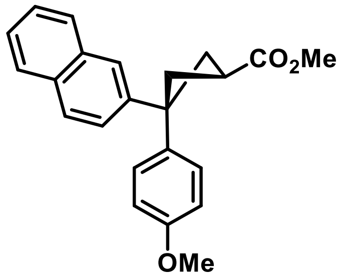

Following GP-2 the title compound was prepared by reaction of methyl 3-(naphthalen-2-yl)bicyclo[1.1.0]butane-1-carboxylate (23.8 mg; 0.10 mmol), anisole (108.1 mg, 110 µL; 1.0 mmol), $TBABF_4$ (20.5 mg, 0.06 mmol, 0.02 M) in HFIP (3 mL); where the solution was electrolyzed at room temperature under alternating constant current conditions, 2 mA and 1.5 F/ mol (current alternation= 1 min) using glassy carbon anode and cathode. Column chromatography (SiO_2, EtOAc/ *n*-pentane 1: 20) afforded the desired product **5i** (19.0 mg, 0.055 mmol, 55%, *p*:*o* 5:1) as a colorless oil.

$\mathbf{R_f}$ (EtOAc/ *n*-pentane 1: 20) = 0.43.

Analytical data were recorded of the pure p-substituted regioisomer **5i**. Pure p-substituted regioisomer was isolated as mixture of diastereomers in 3.4:1 dr.

^{1}H-NMR (500 MHz, $CDCl_3$): Major diastereomer δ = 7.91 (d, J = 2.1 Hz, 1H), 7.85 – 7.81 (m, 1H), 7.80 – 7.73 (m, 2H), 7.50 – 7.34 (m, 3H), 7.16 – 7.10 (m, 2H), 6.82 – 6.77 (m, 2H), 3.74 (s, 3H), 3.67 (s, 3H), 3.23 – 2.98 (m, 5H). Minor diastereomer δ = 7.79 – 7.73 (m, 2H), 7.71 (d, J = 8.9 Hz, 1H), 7.62 (d, J = 2.4 Hz, 1H), 7.50 – 7.34 (m, 4H), 7.24 (dd, J = 8.9, 1.8 Hz, 1H), 6.86 – 6.83 (m, 2H), 3.77 (s, 3H), 3.66 (s, 3H), 3.24 – 2.98 (m, 5H).

^{13}C NMR (126 MHz, $CDCl_3$): Major diastereomer δ= 175.4, 157.7, 144.4, 141.8, 133.3, 131.9, 128.5, 127.9, 127.6, 127.1, 126.3, 126.2, 125.8, 123.8, 113.8, 55.3, 51.8, 47.0, 37.9, 32.9. Minor diastereomer δ = 175.4, 157.9, 147.4, 138.9, 133.4, 131.8, 128.3, 127.9, 127.8, 127.6, 126.2, 125.6, 124.8, 123.4, 113.9, 55.3, 51.8, 46.9, 37.9, 33.0.

IR (ATR): $\tilde{\nu}$ (cm^{-1}) = 3053, 2989, 2949, 2907, 2835, 1729, 1609, 1580, 1510, 1462, 1434, 1362, 1297, 1272, 1246, 1198, 1176, 1153, 1128, 1053, 1034.

HRMS (ESI+) m/z: $[M+Na]^+$ Calculated for $C_{23}H_{22}O_3Na^+$ 369.1461; Found: 369.1464.

3-(4-Methoxyphenyl)-3-phenylcyclobutyl)(phenyl)methanone (5j)

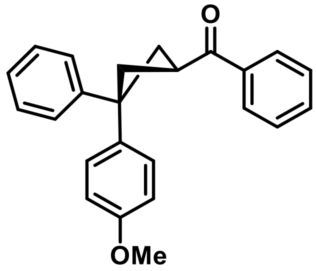

Following GP-2 the title compound was prepared by reaction of phenyl(3-phenylbicyclo[1.1.0]butan-1-yl)methanone (23.4 mg; 0.10 mmol), anisole (108.1 mg, 110 µL; 1.0 mmol), $TBABF_4$ (20.5 mg, 0.06 mmol, 0.02 M) in HFIP (3 mL); where the solution was electrolyzed at room temperature under alternating constant current conditions, 2 mA and 1.5 F/ mol (current alternation= 1 min) using glassy carbon anode and cathode. Column chromatography (SiO_2, EtOAc/ *n*-pentane 1: 20) afforded the desired product **5j** (20.5 mg, 0.060 mmol, 60%, *p*:*o* 4:1) as a colorless oil.

$\mathbf{R_f}$ (EtOAc/ *n*-pentane 1: 20) = 0.32.

Analytical data were recorded of the pure *p*-substituted regioisomer **5j**. Pure *p*-substituted regioisomer was isolated as mixture of diastereomers in 10:1 dr.

^{1}**H-NMR** (700 MHz, $CDCl_3$): δ = 7.91 – 7.86 (m, 2H), 7.57 – 7.52 (m, 1H), 7.50 – 7.47 (m, 2H), 7.46 – 7.42 (m, 2H), 7.38 – 7.34 (m, 2H), 7.24 – 7.21 (m, 1H), 7.10 – 7.06 (m, 2H), 6.79 – 6.76 (m, 2H), 3.99 – 3.93 (m, 1H), 3.74 (s, 3H), 3.13 – 3.05 (m, 4H).

^{13}**C NMR** (176 MHz, $CDCl_3$): δ= 200.6, 157.6, 147.9, 142.3, 135.7, 133.1, 128.7, 128.6, 128.5, 126.9, 126.7, 126.1, 113.8, 55.3, 46.6, 38.3, 38.2, 36.9.

IR (ATR): $\tilde{\nu}$ (cm^{-1}) = 3083, 3057, 3026, 2983, 2936, 2834, 1677, 1609, 1597, 1580, 1511, 1493, 1463, 1447, 1352, 1299, 1271, 1247, 1225, 1178, 1034, 1002.

HRMS (APCI+) m/z: $[M+H]^+$ Calculated for $C_{24}H_{23}O_2^+$ 343,1693; Found: 343.1693.

1-(3-(4-Methoxyphenyl)-3-phenylcyclobutyl)ethan-1-one (5k)

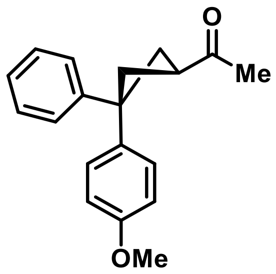

Following GP-2 the title compound was prepared by reaction of 1-(3-phenylbicyclo[1.1.0]butan-1-yl)ethan-1-one (17.1 mg; 0.10 mmol), anisole (107.1 mg; 0.99 mmol), $TBABF_4$ (42.3 mg, 0.13 mmol) in HFIP (4 mL); where the solution was electrolyzed at room temperature under alternating constant current conditions; 1 mA and 1.5 F/ mol (current alternation= 1 min^{-1}) using glassy carbon anode and cathode. Analysis by ^{1}H-NMR of the crude reaction mixture revealed the desired product formed as a 3.6: 1 diastereomeric mixture. Purification by column chromatography (SiO_2, EtOAc: *n*-pentane= 1: 9) afforded product **5k** (13.3 mg, 0.047 mmol, 48%) as a colorless oil, as mixture of diastereomers in 4.3: 1 dr.

$\mathbf{R_f}$ (EtOAc: *n*-pentane= 1: 9) = 0.30.

^{1}H-NMR (700 MHz, $CDCl_3$) δ (major diastereomer) 7.43 – 7.40 (m, 2H), 7.36 – 7.31 (m, 2H), 7.22 – 7.17 (m, 1H), 7.07 (d, *J* = 8.9 Hz, 2H), 6.78 (d, *J* = 8.9 Hz, 2H), 3.75 (s, 3H), 3.21 (dtt, *J* = 11.2, 9.7, 8.4 Hz, 1H), 2.96 (ddd, *J* = 9.5, 8.5, 2.5 Hz, 1H), 2.87 (ddd, *J* = 9.5, 8.5, 2.6 Hz, 1H), 2.09 (s, 3H); (minor diastereomer) 7.36 – 7.30 (m, 1H), 7.25 – 7.22 (m, 2H), 7.16 – 7.13 (m, 2H), 7.12 – 7.09 (m, 2H), 6.86 (d, *J* = 8.9 Hz, 2H), 3.78 (s, 3H), 3.24 – 3.18 (m, 1H), 2.98 – 2.92 (m, 2H), 2.90 – 2.85 (m, 2H), 2.09 (s, 3H).

^{13}C NMR (176 MHz, $CDCl_3$) δ (major diastereomer) 209.5, 157.7, 147.7, 142.2, 128.6, 126.9, 126.7, 126.2, 113.9, 55.4, 46.1, 40.9, 37.5, 27.5; (minor diastereomer) 209.5, 157.9, 150.2, 139.4, 128.5, 127.8, 125.8, 125.7, 114.0, 55.4, 45.9, 41.0, 37.4, 27.5.

IR (ATR): $\tilde{\nu}$ (cm^{-1}) = 2962, 1707, 1610, 1511, 1494, 1463, 1444, 1361, 1297, 1247, 1180, 1117, 1032, 833, 774, 759, 727, 700, 569, 541.

HRMS (ESI) m/z: $[M+Na]^+$ calculated for $C_{19}H_{20}O_2Na$, 303.1356; found: 303.1357.

1-(3-(4-Methoxyphenyl)-3-phenylcyclobutyl)pentan-1-one (5l)

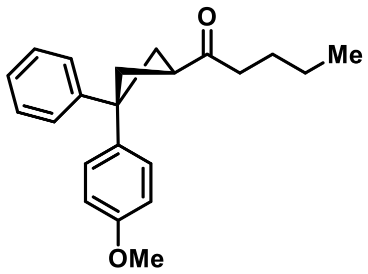

Following GP-2 the title compound was prepared by reaction of 1-(3-phenylbicyclo[1.1.0]butan-1-yl)pentan-1-one (24.3 mg; 0.11 mmol), anisole (108.1 mg; 1.00 mmol), $TBABF_4$ (38.9 mg, 0.12 mmol) in HFIP (4 mL); where the solution was electrlyzed at room temperature under alternating constant current conditions; 1 mA and 1.5 F/ mol (current alternation= 1 min^{-1}) using glassy carbon anode and cathode. Analysis by ^1H-NMR of the crude reaction mixture revealed the desired product formed as a 3.6: 1 diastereomeric mixture. Purification by column chromatography (SiO_2, EtOAc: *n*-pentane= 1: 20) afforded product **5l** (14.9 mg, 0.046 mmol, 41%) as a colorless oil, as mixture of diastereomers in 3.6: 1 dr.

R_f (EtOAc: *n*-pentane= 1: 20) = 0.37.

^{1}H-NMR (700 MHz, $CDCl_3$) δ (major diastereomer) 7.44 – 7.40 (m, 2H), 7.36 – 7.30 (m, 2H), 7.21 – 7.18 (m, 1H), 7.07 (d, *J* = 9.0 Hz, 2H), 6.78 (d, *J* = 9.0 Hz, 2H), 3.75 (s, 3H), 3.25 – 3.16 (m, 1H), 2.97 – 2.90 (m, 2H), 2.87 (ddt, *J* = 11.6, 9.5, 2.1 Hz, 2H), 2.38 – 2.34 (m, 2H), 1.56 – 1.48 (m, 2H), 1.32 – 1.24 (m, 2H), 0.89 (t, *J* = 7.4 Hz, 3H); (minor diastereomer) 7.37 – 7.30 (m, 2H), 7.25 – 7.22 (m, 2H), 7.16 – 7.13 (m, 2H), 7.12 – 7.09 (m, 1H), 6.88 – 6.85 (m, 2H), 3.78 (s, 3H), 3.30 – 3.17 (m, 1H), 2.97 – 2.84 (m, 4H), 2.36 (t, *J* = 7.3 Hz, 2H), 1.56 – 1.50 (m, 2H), 1.32 – 1.23 (m, 2H), 0.89 (t, *J* = 7.4 Hz, 3H).

^{13}C NMR (176 MHz, $CDCl_3$) δ (major diastereomer) 211.8, 157.7, 147.7, 142.3, 128.6, 126.9, 126.7, 126.1, 113.8, 55.4, 46.2, 40.3, 40.1, 37.5, 25.9, 22.6, 13.9; (minor diastereomer) 211.8, 157.9, 150.3, 139.4, 128.4, 127.8, 126.1, 125.7, 113.9, 55.4, 46.1, 40.3, 40.2, 37.4, 25.9, 22.6, 13.9.

IR (ATR): $\tilde{\nu}$ (cm^{-1}) = 2955, 2933, 1705, 1610, 1511, 1494, 1463, 1444, 1299, 1247, 1179, 1160, 1035, 832, 777, 759, 727, 699, 569, 543.

HRMS (ESI) m/z: $[M+Na]^+$ calculated for $C_{22}H_{26}O_2Na$, 345.1825; found: 345.1826.

Methyl 3-phenyl-3-(p-tolyl)cyclobutane-1-carboxylate (5m)

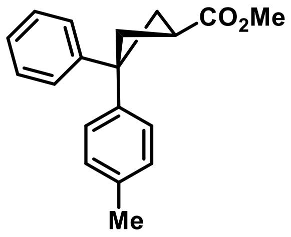

Following GP-2 the title compound was prepared by reaction of methyl 3-phenylbicyclo[1.1.0]butane-1-carboxylate (18.8 mg; 0.10 mmol), toluene (92.1 mg, 110 µL; 1.0 mmol), $TBABF_4$ (20.5 mg, 0.06 mmol, 0.02 M) in HFIP (3 mL); where the solution was electrolyzed at room temperature under alternating constant current conditions, 2 mA and 1.5 F/ mol (current alternation= 1 min) using glassy carbon anode and cathode. Column chromatography (SiO_2, EtOAc/ *n*-pentane 1: 50) afforded the desired product **5m** (10.5 mg, 0.038 mmol, 38%) as a colorless oil, as mixture of diastereomers in 3.1:1 dr.

$\mathbf{R_f}$ (EtOAc/ *n*-pentane 1: 50) = 0.21.

^{1}H-NMR (500 MHz, $CDCl_3$): δ = 1H NMR (500 MHz, CDCl3) δ 7.44 – 7.37 (m, 2H), 7.34 – 7.27 (m, 2H), 7.21 – 7.15 (m, 1H), 7.06 (s, 4H), 3.65 (s, 3H), 3.18 – 3.07 (m, 1H), 3.05 – 2.92 (m, 4H).

^{13}C NMR (126 MHz, $CDCl_3$): δ= 175.4, 147.2, 147.0, 135.3, 129.1, 128.6, 126.6, 126.1, 125.7, 51.8, 47.1, 38.1, 33.0, 21.0.

IR (ATR): $\tilde{\nu}$ (cm^{-1}) = 3052, 3021, 2988, 2949, 2923, 2867, 1732, 1599, 1513, 1494, 1445, 1435, 1363, 1267, 1223, 1196, 1177, 1155, 1117, 1079, 1051, 1021.

HRMS (ESI+) m/z: $[M+Na]^+$ Calculated for $C_{19}H_{20}O_2Na$ 303.1356; Found: 303.1358.

Methyl 3-(4-(dimethylamino)phenyl)-3-phenylcyclobutane-1-carboxylate (5n)

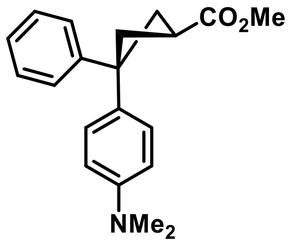

Following GP-2 the title compound was prepared by reaction of methyl 3-phenylbicyclo[1.1.0]butane-1-carboxylate (18.8 mg; 0.10 mmol), *N,N*-dimethylaniline (121.2 mg, 125 µL; 1.0 mmol), $TBABF_4$ (20.5 mg, 0.06 mmol, 0.02 M) in HFIP (3 mL); where the solution was electrolyzed at room temperature under alternating constant current conditions, 2 mA and 1.5 F/ mol (current alternation= 1 min) using glassy carbon anode and cathode. Column chromatography (SiO_2, EtOAc/ *n*-pentane 1: 20) afforded the desired product **5n** (13.0 mg, 0.042 mmol, 42%) as a colorless oil, as mixture of diastereomers in 2.0:1 dr.

R_f (EtOAc/ *n*-pentane 1: 20) = 0.18.

^{1}H-NMR (500 MHz, $CDCl_3$): δ = 7.41 – 7.34 (m, 2H), 7.32 – 7.26 (m, 2H), 7.24 – 7.21 (m, 1H), 7.19 – 7.14 (m, 2H), 7.12 – 7.07 (m, 1H), 7.06 – 7.02 (m, 2H, (minor diast.)), 6.75 – 6.63 (m, 2H), 3.66 (s, 3H, (minor diast.)), 3.65 (s, 3H), 3.20 – 3.06 (m, 1H), 3.03 – 2.86 (m, 10H).

^{13}C NMR (126 MHz, $CDCl_3$): Major diastereomer δ= 175.6, 150.6, 140.7, 128.3, 126.7, 125.7, 125.5, 112.8, 51.8, 46.5, 40.9, 38.0, 33.1. Minor diastereomer δ = 175.5, 150.6, 140.7, 128.5, 127.4, 126.7, 125.9, 112.8, 51.8, 46.7, 40.9, 38.2, 32.9.

IR (ATR): $\tilde{\nu}$ (cm^{-1}) = 3022, 2985, 2948, 2883, 2847, 2797, 1730, 1612, 1519, 1493, 1444, 1435, 1349, 1223, 1196, 1177, 1081, 1054.

HRMS (ESI+) m/z: $[M+H]^+$ Calculated for $C_{20}H_{24}O_2N$ 310.1802; Found: 310.1806.

Methyl 3-(5-bromo-2-methoxyphenyl)-3-phenylcyclobutane-1-carboxylate (5p)

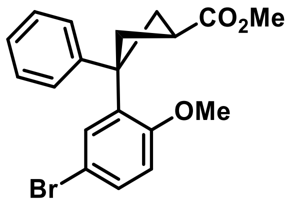

Following GP-2 the title compound was prepared by reaction of methyl 3-phenylbicyclo[1.1.0]butane-1-carboxylate (18.8 mg; 0.10 mmol), 4-bromoanisole (187.0 mg, 125 µL; 1.0 mmol), $TBABF_4$ (20.5 mg, 0.06 mmol, 0.02 M) in HFIP (3 mL); where the solution was electrolyzed at room temperature under alternating constant current conditions, 2 mA and 1.5 F/ mol (current alternation= 1 min) using glassy carbon anode and cathode. Column chromatography (SiO_2, EtOAc/ *n*-pentane 1: 20) afforded the desired product **5p** (17.0 mg, 0.045 mmol, 45%) as a colorless oil, as mixture of diastereomers in 2.7:1 dr.

R_f (EtOAc/ *n*-pentane 1: 20) = 0.23.

Analytical data were recorded of the pure major diastereomer.

^{1}H-NMR (700 MHz, $CDCl_3$): δ = 7.46 – 7.44 (m, 2H), 7.34 (d, J = 2.5 Hz, 1H), 7.30 – 7.27 (m, 2H), 7.23 (dd, J = 8.7, 2.5 Hz, 1H), 7.18 – 7.15 (m, 1H), 6.60 (d, J = 8.7 Hz, 1H), 3.66 (s, 3H), 3.65 (s, 3H), 3.13 – 3.07 (m, 1H), 3.05 – 3.01 (m, 2H), 2.90 – 2.85 (m, 2H).

^{13}C NMR (176 MHz, $CDCl_3$): δ = 175.4, 155.8, 145.5, 139.8, 130.1, 129.7, 128.2, 126.7, 126.1, 112.9, 112.8, 55.4, 51.8, 45.7, 37.9, 33.5.

IR (ATR): $\tilde{\nu}$ (cm^{-1}) = 2995, 2949, 2842, 1770, 1758, 1732, 1486, 1462, 1436, 1392, 1366, 1291, 1245, 1205, 1178, 1156, 1137, 1052, 1028.

HRMS (APCI+) m/z: $[M]^+$ Calculated for $C_{19}H_{19}O_3Br$ 374.0512; Found: 374.0514.

Methyl 3-(2,5-dimethoxyphenyl)-3-phenylcyclobutane-1-carboxylate (5q)

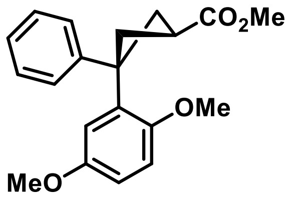

Following GP-2 the title compound was prepared by reaction of methyl 3-phenylbicyclo[1.1.0]butane-1-carboxylate (18.8 mg; 0.10 mmol), 1,4-dimethoxybenzene (138.1 mg, 1.0 mmol), $TBABF_4$ (20.5 mg, 0.06 mmol, 0.02 M) in HFIP (3 mL); where the solution was electrolyzed at room temperature under alternating constant current conditions, 2 mA and 1.5 F/ mol (current alternation= 1 min) using glassy carbon anode and cathode. Column chromatography (SiO_2, EtOAc/ *n*-pentane 1: 20) afforded the desired product **5q** (7.0 mg, 0.022 mmol, 22%) as a colorless oil, as mixture of diastereomers in 5:1 dr.

R_f (EtOAc/ *n*-pentane 1: 20) = 0.10.

^{1}H-NMR (500 MHz, $CDCl_3$): δ = 7.49 – 7.46 (m, 2H), 7.29 – 7.25 (m, 2H), 7.16 – 7.13 (m, 1H), 6.83 (dd, J = 2.4, 1.0 Hz, 1H), 6.67 – 6.63 (m, 2H), 3.78 (s, 3H), 3.65 (s, 3H), 3.63 (s, 3H), 3.11 – 3.00 (m, 3H), 2.93 – 2.87 (m, 2H).

^{13}C NMR (126 MHz, $CDCl_3$): δ = 175.6, 153.5, 151.1, 146.1, 138.9, 128.1, 126.7, 125.9, 113.8, 112.2, 110.9, 55.8, 55.7, 51.7, 45.9, 38.1, 33.6.

IR (ATR): $\tilde{\nu}$ (cm^{-1}) = 2995, 2949, 2907, 2832, 1770, 1732, 1588, 1493, 1463, 1444, 1421, 1364, 1280, 1244, 1223, 1201, 1175, 1155, 1047, 1029.

HRMS (ESI+) m/z: $[M+Na]^+$ calculated for $C_{20}H_{22}O_4Na$ 349.1410; Found: 349.1412.

Methyl 3-(2,5-dimethylphenyl)-3-phenylcyclobutane-1-carboxylate (5r)

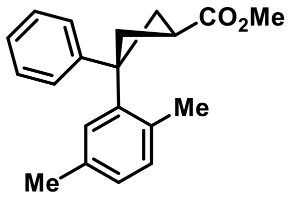

Following GP-2 the title compound was prepared by reaction of methyl 3-phenylbicyclo[1.1.0]butane-1-carboxylate (18.8 mg; 0.10 mmol), p-xylene (109.0 mg, 125 µL; 1.0 mmol), $TBABF_4$ (20.5 mg, 0.06 mmol, 0.02 M) in HFIP (3 mL); where the solution was electrolyzed at room temperature under alternating constant current conditions, 2 mA and 1.5 F/ mol (current alternation= 1 min) using glassy carbon anode and cathode. Column chromatography (SiO_2, EtOAc/ *n*-pentane 1: 50) afforded the desired product **5r** (4.1 mg, 0.014 mmol, 14%) as a colorless oil, as mixture of diastereomers in 4:1 dr.

$\mathbf{R_f}$ (EtOAc/ *n*-pentane 1: 50) = 0.12.

^{1}H-NMR (500 MHz, $CDCl_3$): δ = 7.37 – 7.33 (m, 2H), 7.30 – 7.27 (m, 2H), 7.19 – 7.14 (m, 2H), 6.96 – 6.91 (m, 1H), 6.90 (d, J = 7.5 Hz, 1H), 3.66 (s, 3H), 3.30 – 3.22 (m, 1H), 3.01 – 2.90 (m, 4H), 2.38 (s, 3H), 1.92 (s, 3H).

^{13}C NMR (126 MHz, $CDCl_3$): δ = 175.3, 146.7, 145.8, 134.9, 132.5, 131.3, 128.4, 127.4, 127.1, 126.5, 125.9, 51.8, 47.6, 38.1, 33.1, 21.3, 19.6.

IR (ATR): $\tilde{\nu}$ (cm^{-1}) = 3019, 2987, 2949, 2867, 1732, 1599, 1494, 1444, 1435, 1364, 1268, 1234, 1195, 1178, 1156, 1081, 1055, 1028.

HRMS (APCI+) m/z: $[M]^+$ Calculated for $C_{20}H_{22}O_2$ 294.1614; Found: 294.1614.

Methyl 3-mesityl-3-phenylcyclobutane-1-carboxylate (5s)

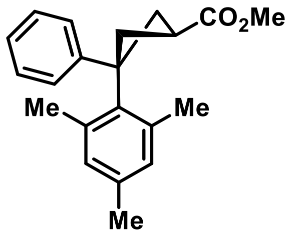

Following GP-2 the title compound was prepared by reaction of methyl 3-phenylbicyclo[1.1.0]butane-1-carboxylate (18.8 mg; 0.10 mmol), mesitylene (120.0 mg, 140 µL; 1.0 mmol), $TBABF_4$ (20.5 mg, 0.06 mmol, 0.02 M) in HFIP (3 mL); where the solution was electrolyzed at room temperature under alternating constant current conditions, 2 mA and 1.5 F/ mol (current alternation= 1 min) using glassy carbon anode and cathode. Column chromatography (SiO_2, EtOAc/ *n*-pentane 1: 50) afforded the desired product **5s** (6.0 mg, 0.020 mmol, 20%) as a colorless oil, as mixture of diastereomers in 1.6: 1 dr.

R_f (EtOAc/ *n*-pentane 1: 50) = 0.13.

^{1}H-NMR (700 MHz, $CDCl_3$): Major diastereomer δ = 7.37 – 7.34 (m, 2H), 7.33 – 7.26 (m, 2H), 7.18 – 7.14 (m, 1H), 7.11 (s, 1H), 6.79 (s, 1H), 3.65 (s, 3H), 3.29 – 3.21 (m, 1H), 3.08 (ddt, J = 12.4, 10.1, 2.5 Hz, 1H), 2.99 – 2.90 (m, 3H), 2.29 (s, 3H), 2.19 (s, 3H), 1.90 (s, 3H). Minor diastereomer δ = 7.33 – 7.26 (m, 4H), 7.22 – 7.18 (m, 1H), 6.82 – 6.81 (m, 2H), 3.66 (s, 3H), 3.45 – 3.37 (m, 1H), 2.99 – 2.90 (m, 4H), 2.26 (s, 3H), 2.11 (s, 6H).

^{13}C NMR (176 MHz, $CDCl_3$): Both diastereomers δ= 175.5, 175.3, 146.4, 146.0, 144.4, 143.3, 136.2, 135.4, 134.5, 133.4, 132.8, 132.7, 130.4, 128.7, 128.4, 127.9, 126.4, 126.2, 126.1, 125.9, 51.8, 51.7, 48.1, 47.3, 41.2, 38.5, 33.9, 33.2, 21.9, 20.7, 19.6, 19.5, 19.2.

IR (ATR): $\tilde{\nu}$ (cm^{-1}) = 2988, 2948, 2920, 2866, 1733, 1599, 1493, 1443, 1435, 1365, 1266, 1246, 1196, 1179, 1046, 1029.

HRMS (ESI+) m/z: $[M+Na]^+$ Calculated for $C_{21}H_{24}O_2Na$ 331.1669; Found: 331.1670.

Methyl 3-(benzo[d][1,3]dioxol-5-yl)-3-phenylcyclobutane-1-carboxylate (5t)

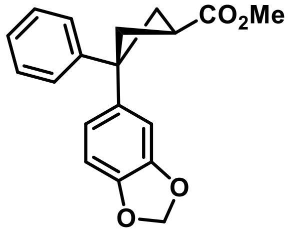

Following GP-2 the title compound was prepared by reaction of methyl 3-phenylbicyclo[1.1.0]butane-1-carboxylate (18.8 mg; 0.10 mmol), 1,3-benzodioxole (122.1 mg, 110 µL; 1.0 mmol), $TBABF_4$ (20.5 mg, 0.06 mmol, 0.02 M) in HFIP (3 mL); where the solution was electrolyzed at room temperature under alternating constant current conditions, 2 mA and 1.5 F/ mol (current alternation= 1 min) using glassy carbon anode and cathode. Column chromatography (SiO_2, EtOAc/ *n*-pentane 1: 20) afforded the desired product **5t** (12.0 mg, 0.039 mmol, 39%) as a colorless oil, as mixture of diastereomers in 7.3:1 dr.

R_f (EtOAc/ *n*-pentane 1: 20) = 0.20.

^{1}H-NMR (500 MHz, $CDCl_3$): Both diastereomers δ= 7.41 – 7.36 (m, 2H), 7.34 – 7.29 (m, 2H), 7.27 – 7.24 (m, 2H, minor diast.), 7.21 – 7.11 (m, 1H), 6.91 (dd, J = 8.1, 2.0 Hz, 1H, minor diast.), 6.84 (d, J = 1.5 Hz, 1H, minor diast.), 6.75 (d, J = 8.1 Hz, 1H, minor diast.), 6.70 (dd, J = 8.0, 0.5 Hz, 1H), 6.65 (dd, J = 7.9, 1.8 Hz, 1H), 6.63 (dd, J = 1.8, 0.6 Hz, 1H), 5.91 (s, 2H, minor diast.), 5.88 (s, 2H), 3.66 (s, 3H), 3.65 (s, 3H, minor diast.), 3.17 – 3.05 (m, 1H), 3.02 – 2.89 (m, 4H).

^{13}C NMR (126 MHz, $CDCl_3$): Major diastereomer δ 175.3, 147.7, 147.1, 145.6, 144.2, 128.6, 126.5, 126.2, 118.6, 108.0, 106.7, 100.9, 51.4, 47.3, 38.1, 32.3. Minor diastereomer δ = 175.3, 149.9, 147.9, 145.9, 141.1, 128.5, 125.9, 125.6, 119.3, 107.7, 106.7, 101.1, 51.8, 47.4, 38.1, 33.1.

IR (ATR): $\tilde{\nu}$ (cm^{-1}) = 3057, 2988, 2950, 2891, 1770, 1730, 1600, 1504, 1485, 1434, 1364, 1237, 1204, 1178, 1161, 1105, 1040.

HRMS (ESI+) m/z: $[M+Na]^+$ Calculated for $C_{19}H_{18}O_4Na$ 333.1097; Found: 333.1098.

Methyl 3-phenyl-3-(5,6,7,8-tetrahydronaphthalen-2-yl)cyclobutane-1-carboxylate (5u)

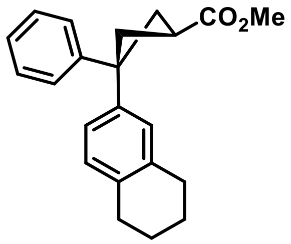

Following GP-2 the title compound was prepared by reaction of methyl 3-phenylbicyclo[1.1.0]butane-1-carboxylate (18.8 mg; 0.10 mmol), tetralin (132.2 mg, 135 µL; 1.0 mmol), $TBABF_4$ (20.5 mg, 0.06 mmol, 0.02 M) in HFIP (3 mL); where the solution was electrolyzed at room temperature under alternating constant current conditions, 2 mA and 1.5 F/ mol (current alternation= 1 min) using glassy carbon anode and cathode. Column chromatography (SiO_2, EtOAc/ *n*-pentane 1: 20) afforded the desired product **5u** (21.0 mg, 0.066 mmol, 66%, β:α >20:1) as a colorless oil.

$\mathbf{R_f}$ (EtOAc/ *n*-pentane 1: 20) = 0.36.

Analytical data were recorded of the pure 2-substituted regioisomer **5u**. Pure 2-substituted tetralin derivative was isolated as mixture of diastereomers in 2.5:1 dr.

^{1}H-NMR (700 MHz, $CDCl_3$): δ = 7.44 – 7.40 (m, 2H), 7.33 – 7.29 (m, 2H), 7.19 – 7.15 (m, 1H), 6.96 – 6.93 (m, 1H), 6.88 (dd, J = 7.9, 2.1 Hz, 1H), 6.87 – 6.85 (m, 1H), 3.65 (s, 3H), 3.15 – 3.08 (m, 1H), 3.04 – 2.92 (m, 4H), 2.72 – 2.65 (m, 4H), 1.79 – 1.71 (m, 4H).

^{13}C NMR (176 MHz, $CDCl_3$): δ = 175.4, 147.2, 147.2, 137.0, 134.6, 129.2, 128.5, 126.6, 126.3, 126.0, 123.0, 51.8, 47.1, 38.1, 33.1, 29.6, 29.0, 23.3, 23.3.

IR (ATR): $\tilde{\nu}$ (cm^{-1}) = 2988, 2927, 2855, 2836, 1770, 1732, 1599, 1580, 1494, 1435, 1363, 1240, 1196, 1177, 1052, 1028.

HRMS (ESI+) m/z: $[M+Na]^+$ Calculated for $C_{22}H_{24}O_2Na$ 343.1669; Found: 343.1669.

Methyl 3-(naphthalen-2-yl)-3-phenylcyclobutane-1-carboxylate (5v)

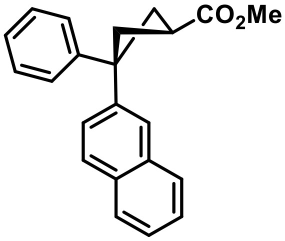

Following GP-2 the title compound was prepared by reaction of methyl 3-phenylbicyclo[1.1.0]butane-1-carboxylate (18.8 mg; 0.10 mmol), naphthalene (128.2 mg, 1.0 mmol), $TBABF_4$ (20.5 mg, 0.06 mmol, 0.02 M) in HFIP (3 mL); where the solution was electrolyzed at room temperature under alternating constant current conditions, 2 mA and 1.5 F/ mol (current alternation= 1 min) using glassy carbon anode and cathode. Column chromatography (SiO_2, EtOAc/ *n*-pentane 1: 50) afforded the desired product (3.1 mg, 0.01 mmol, 10%) as a colorless oil, as mixture of regioisomers β:α >10:1.

R_f (EtOAc/ *n*-pentane 1: 50) = 0.18.

Analytical data were recorded of the pure β-substituted regioisomer **5v**. Pure β-substituted regioisomer was isolated as mixture of diastereomers in 6:1 dr.

^{1}H-NMR (700 MHz, $CDCl_3$): Major diastereomer δ = 7.78 (dq, J = 8.1, 0.6 Hz, 1H), 7.76 – 7.74 (m, 1H), 7.71 (dq, J = 8.6, 0.6 Hz, 1H), 7.66 – 7.63 (m, 1H), 7.48 – 7.42 (m, 3H), 7.40 (ddd, J = 8.1, 6.9, 1.3 Hz, 1H), 7.33 – 7.30 (m, 2H), 7.27 – 7.24 (m, 1H), 7.20 – 7.17 (m, 1H), 3.67 (s, 3H, minor diast.), 3.66 (s, 3H), 3.25 – 3.19 (m, 1H), 3.12 – 3.08 (m, 4H).

^{13}C NMR (176 MHz, $CDCl_3$): Both diastereomers δ = 175.3, 149.5, 147.1, 147.0, 146.8, 144.1, 141.5, 133.4, 133.3, 131.8, 128.9, 128.6, 128.5, 128.4, 127.9, 127.9, 127.6, 126.7, 126.2, 126.2, 125.9, 125.6, 125.3, 124.8, 123.6, 51.9, 47.6, 37.9, 37.8, 33.1, 33.1.

IR (ATR): $\tilde{\nu}$ (cm^{-1}) = 3054, 2988, 2949, 1770, 1758, 1731, 1598, 1494, 1444, 1435, 1366, 1245, 1195, 1127, 1102, 1053.

HRMS (ESI+) m/z: $[M+Na]^+$ Calculated for $C_{22}H_{20}O_2Na$ 339.1356; Found: 339.1356.

7. Spectral data of products

Methyl 1,3-diphenyl-2-oxabicyclo[2.1.1]hexane-4-carboxylate (3a)

[^{1}H-NMR: 700 MHz, ^{13}C-NMR: 176 MHz]

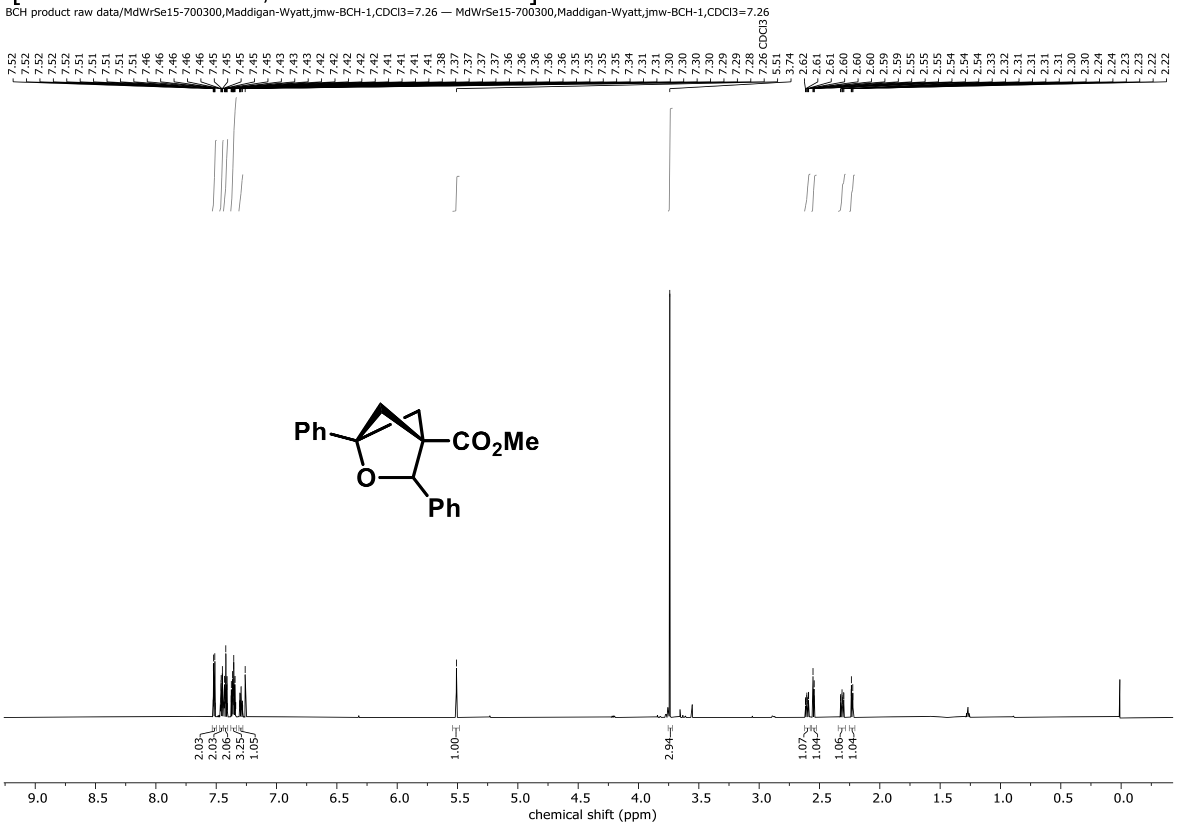

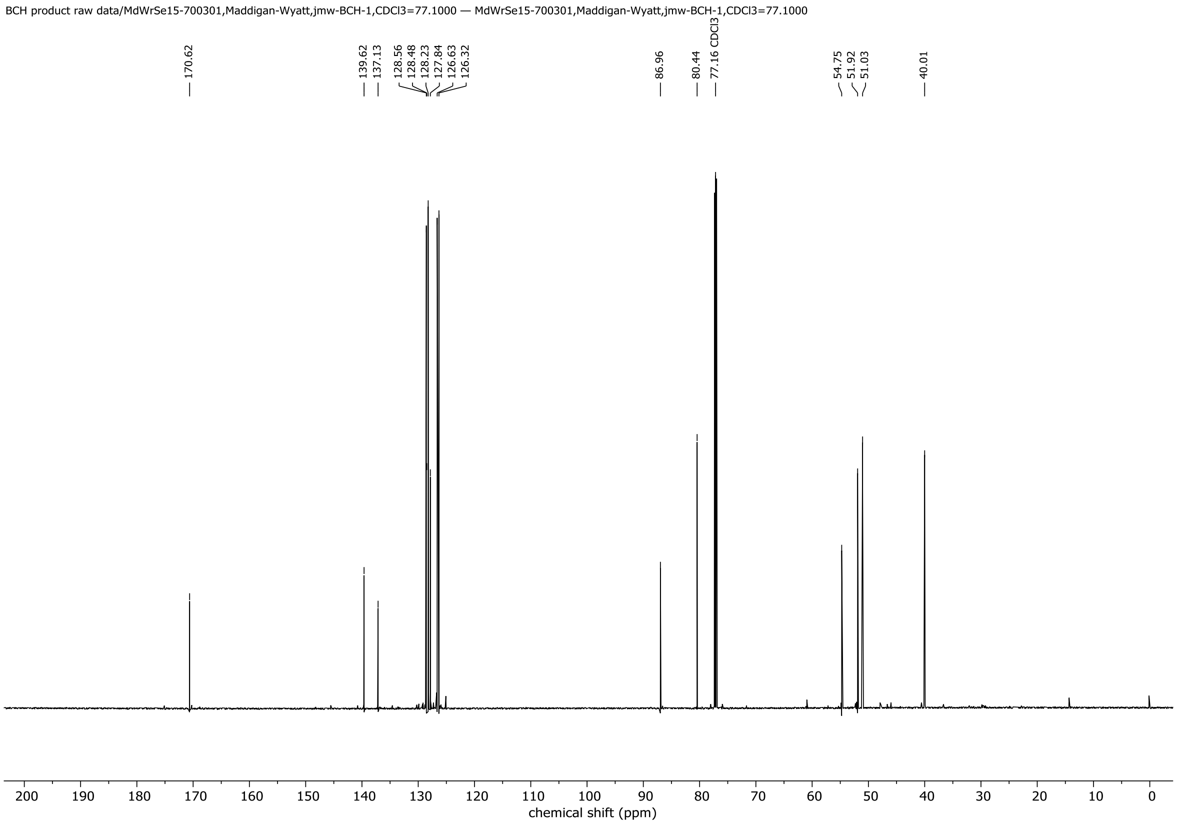

Methyl 1-(4-bromophenyl)-3-phenyl-2-oxabicyclo[2.1.1]hexane-4-carboxylate (3b)

[^{1}H-NMR: 700 MHz, ^{13}C-NMR: 176 MHz]

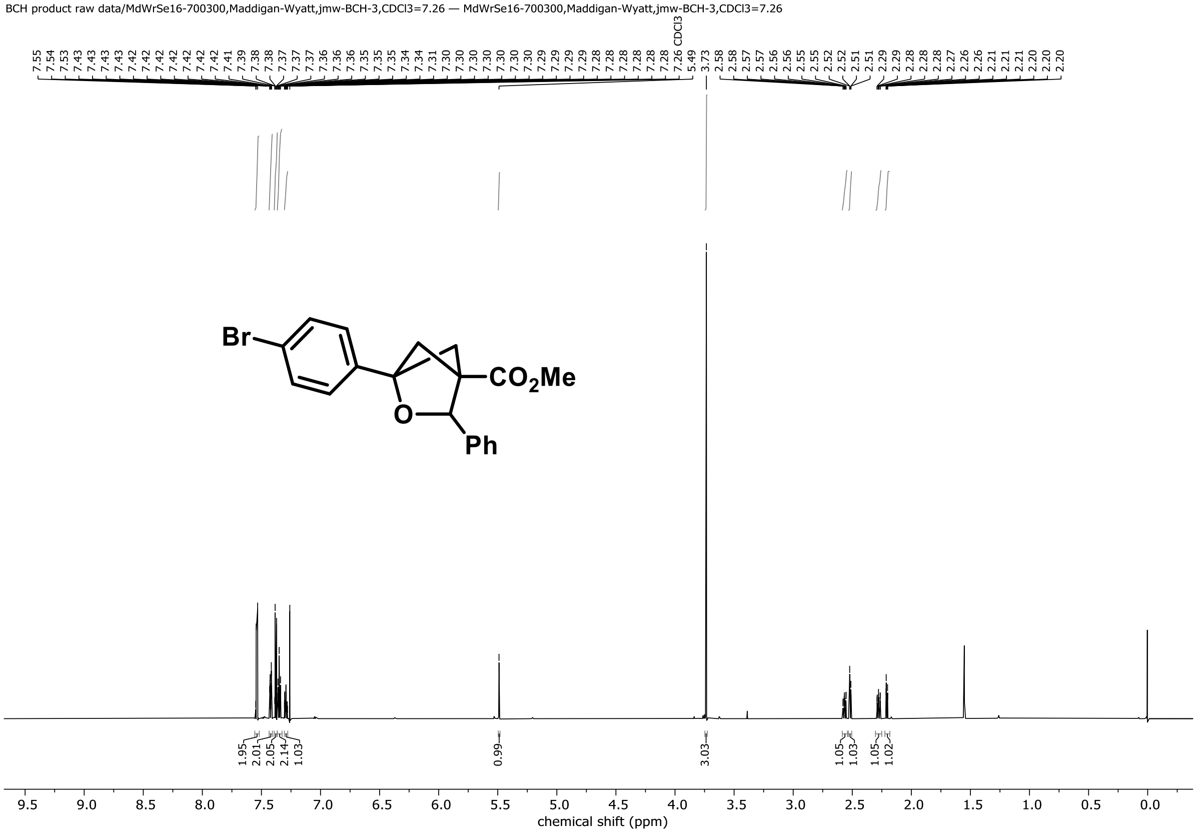

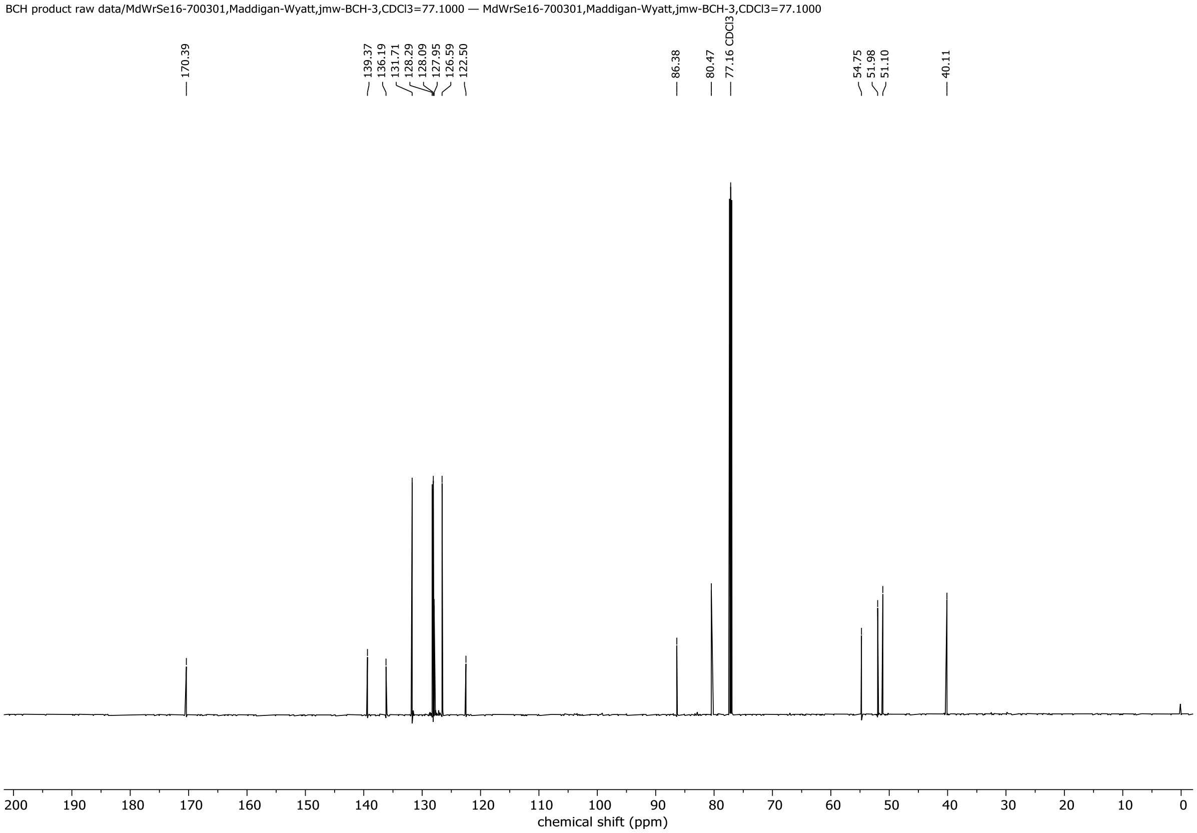

Methyl 1-(4-fluorophenyl)-3-phenyl-2-oxabicyclo[2.1.1]hexane-4-carboxylate (3c)
[^{1}H-NMR: 500 MHz, ^{13}C-NMR: 126 MHz, ^{19}F-NMR: 471 MHz]

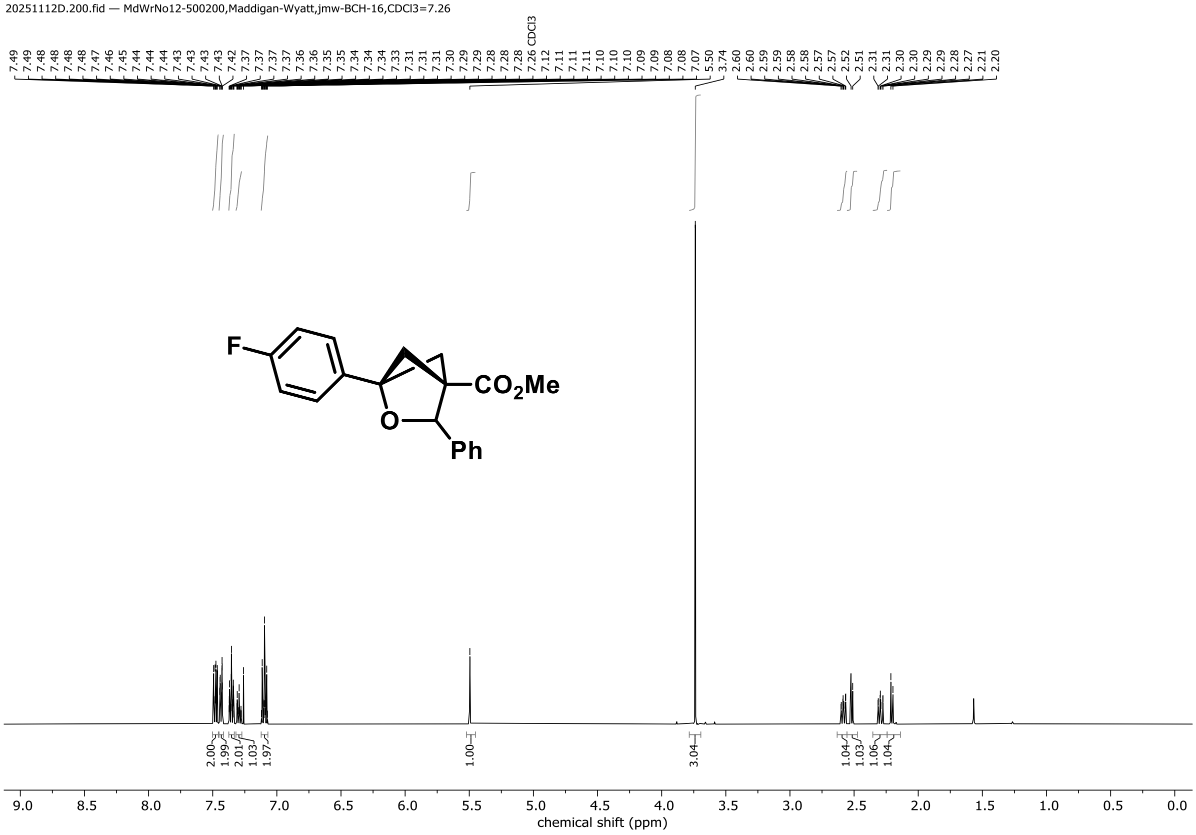

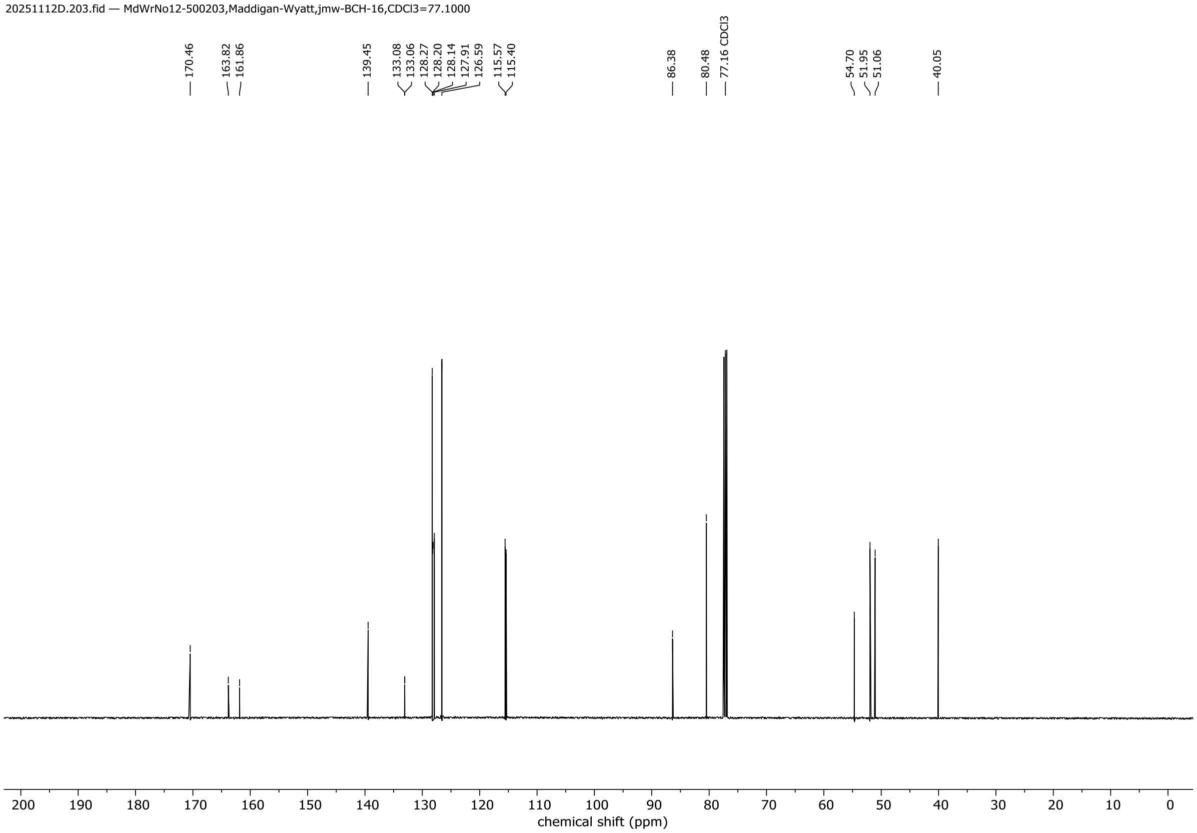

20251112D.201.fid — MdWrNo12-500201,Maddigan-Wyatt,jmw-BCH-16,CDCl3,19F_CPD

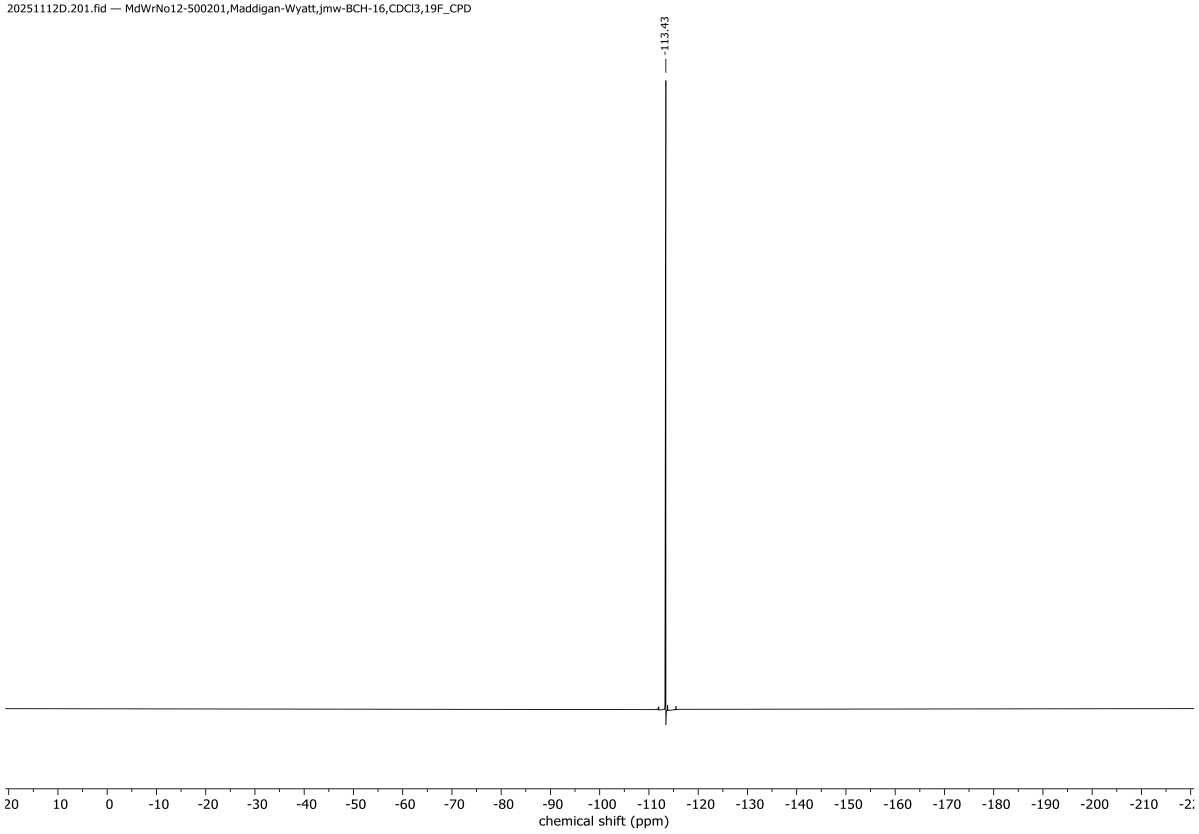

Methyl 3-pentyl-1-phenyl-2-oxabicyclo[2.1.1]hexane-4-carboxylate (3d)

[^{1}H-NMR: 700 MHz, ^{13}C-NMR: 176 MHz]

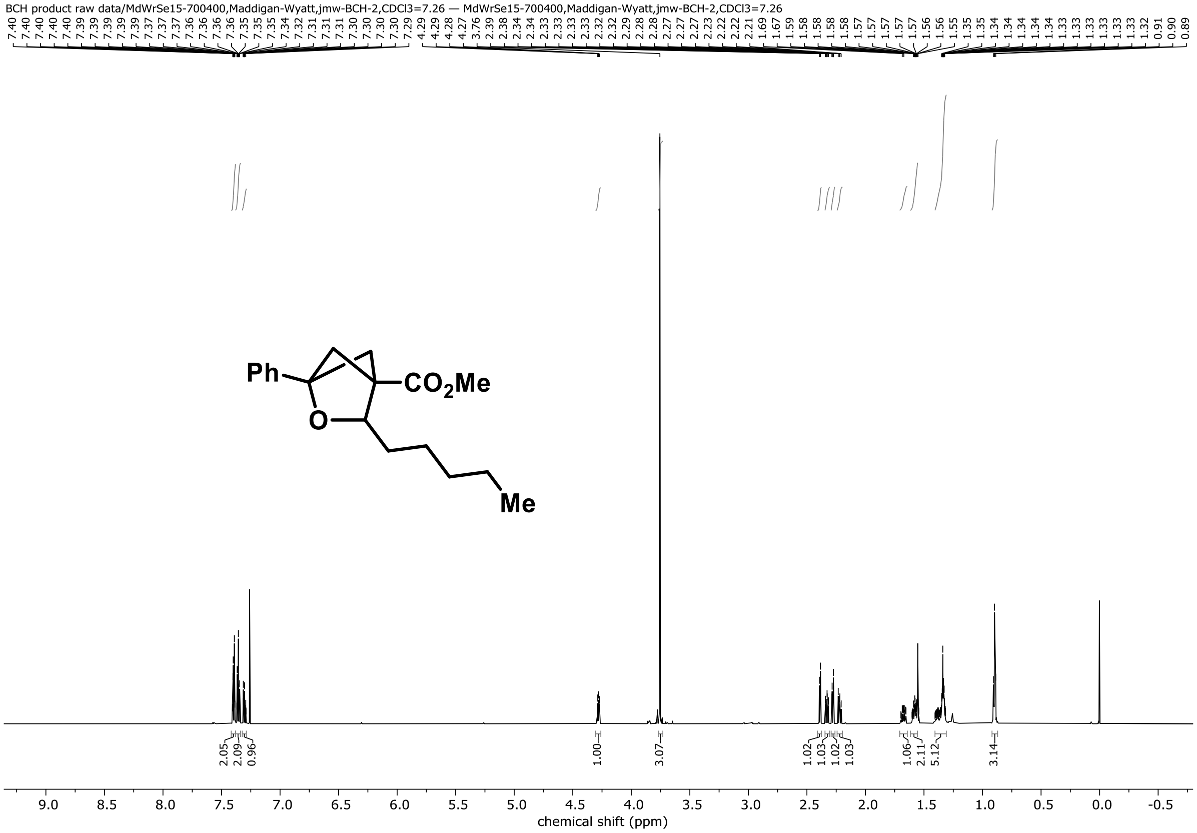

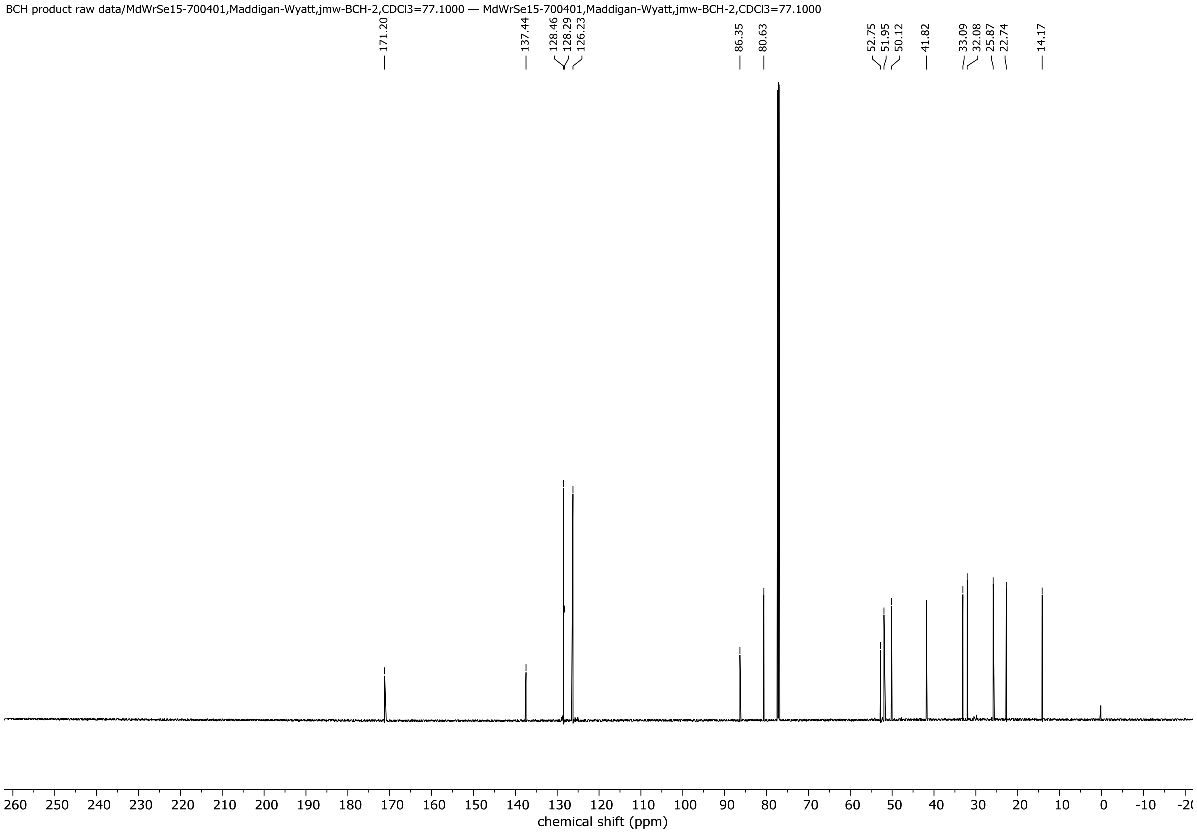

Methyl 3-(perfluorophenyl)-1-phenyl-2-oxabicyclo[2.1.1]hexane-4-carboxylate (3e)

[^{1}H-NMR: 700 MHz, ^{13}C-NMR: 176 MHz, ^{19}F-NMR: 659 MHz]

20251105D.200.fid — MdWrNo05-500200,Maddigan-Wyatt,jmw-BCH-13,CDCl3=7.26

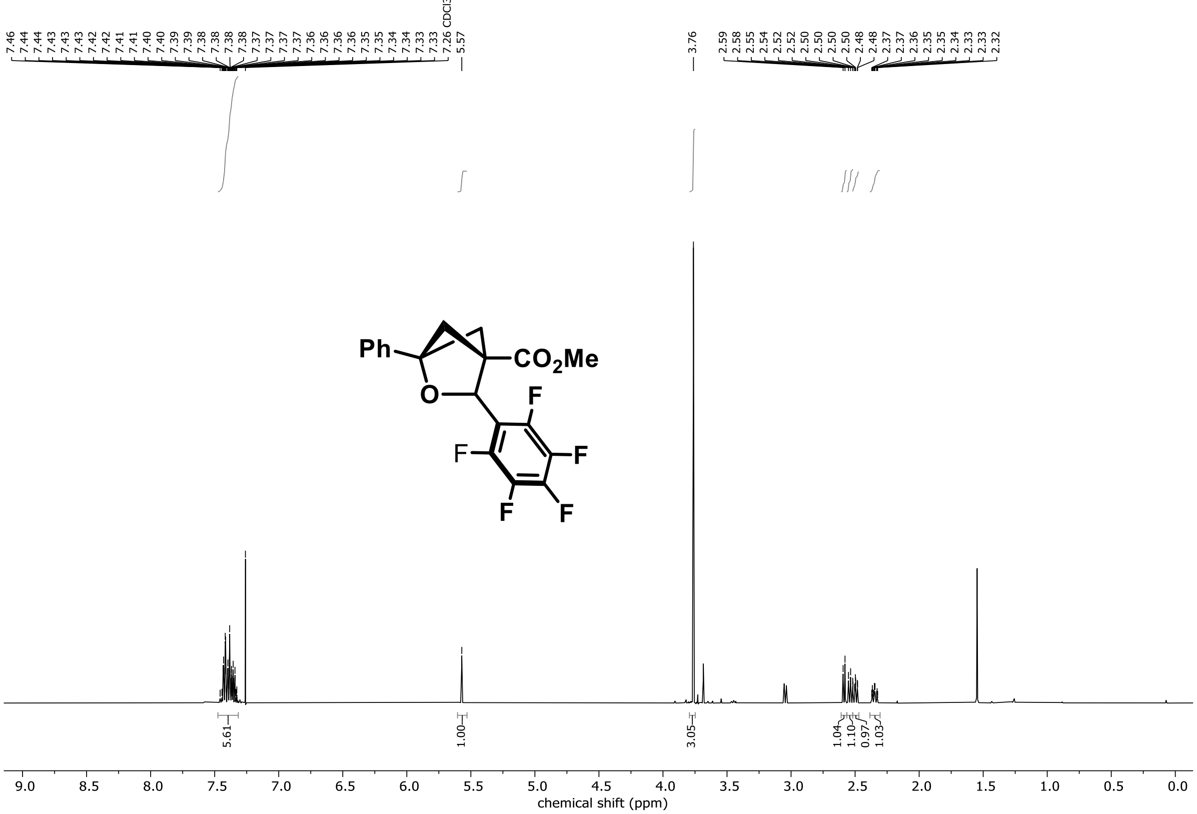

20251105D.203.fid — MdWrNo05-500203,Maddigan-Wyatt,jmw-BCH-13,CDCl3=77.1000{1H_pg,19F_ig=-150ppm},NewExperiment,TestPhase

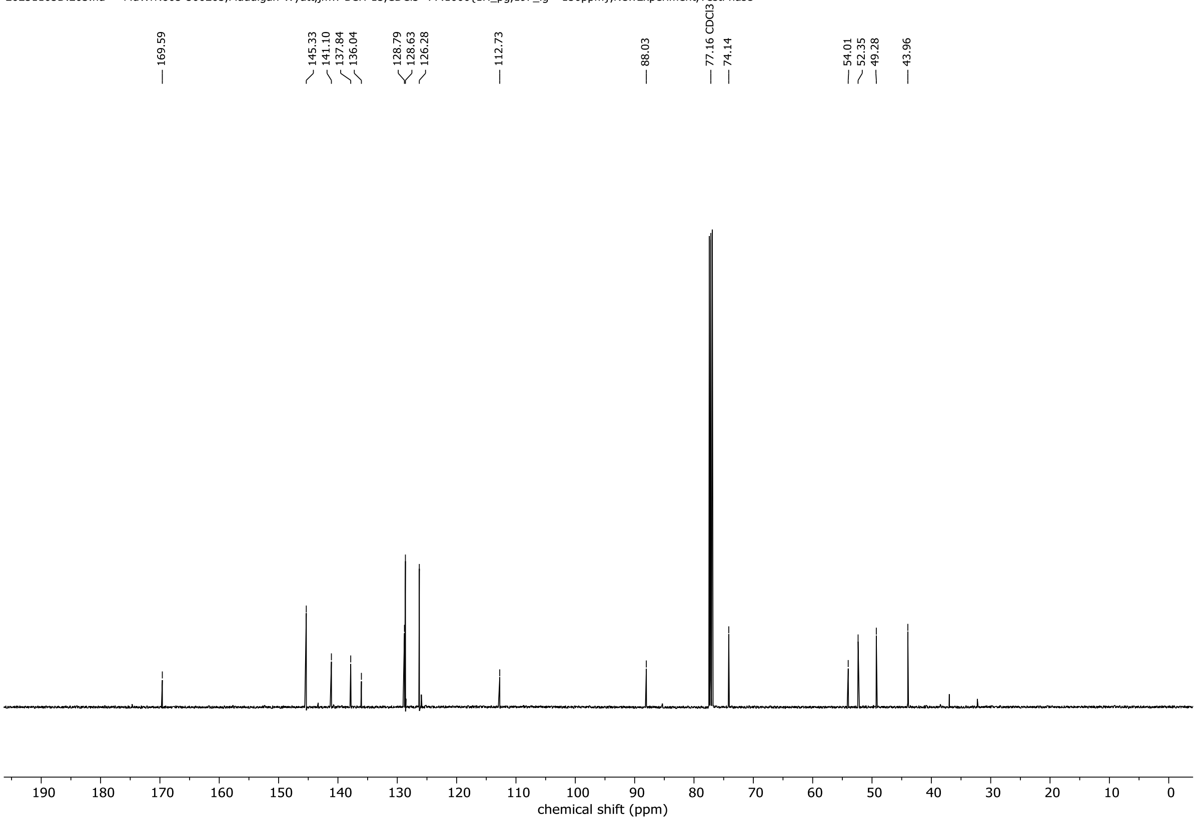

20251105D.202.fid — MdWrNo05-500202,Maddigan-Wyatt,jmw-BCH-13,CDCl3=77.1000

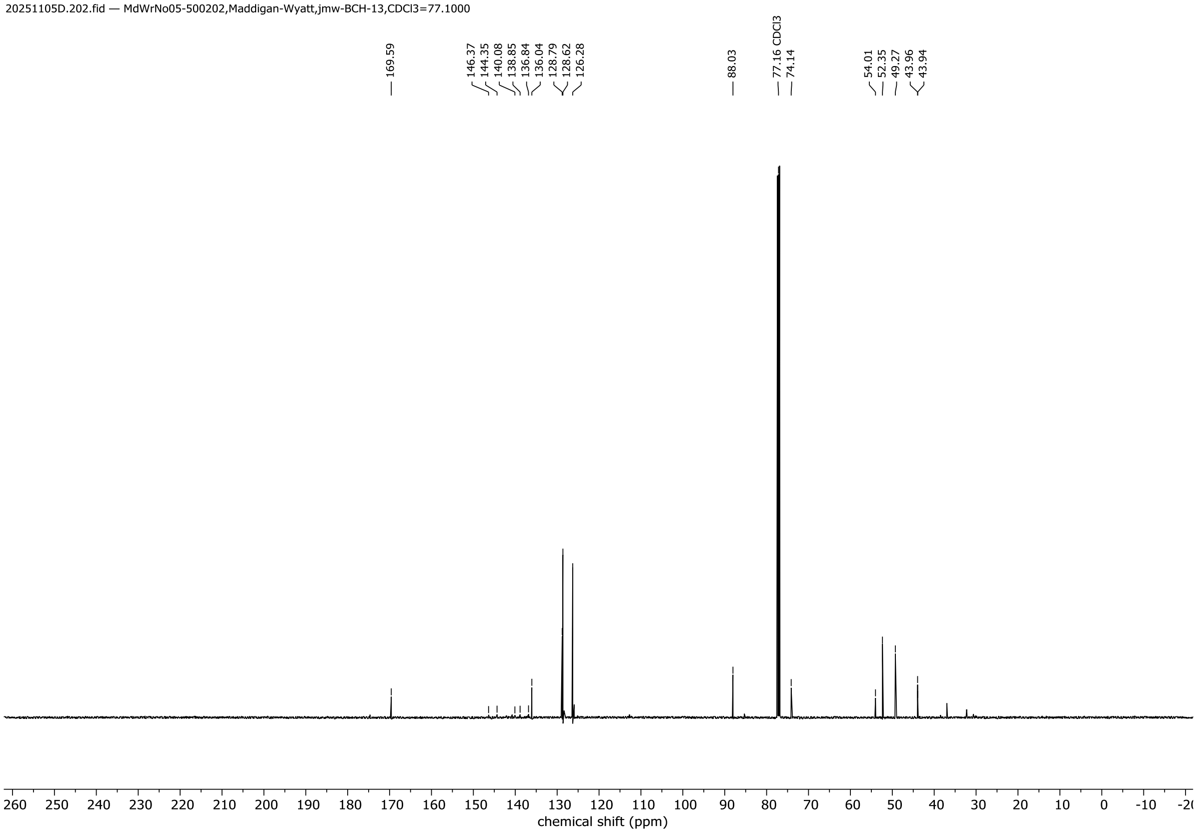

20251105D.201.fid — MdWrNo05-500201,Maddigan-Wyatt,jmw-BCH-13,CDCl3,19F

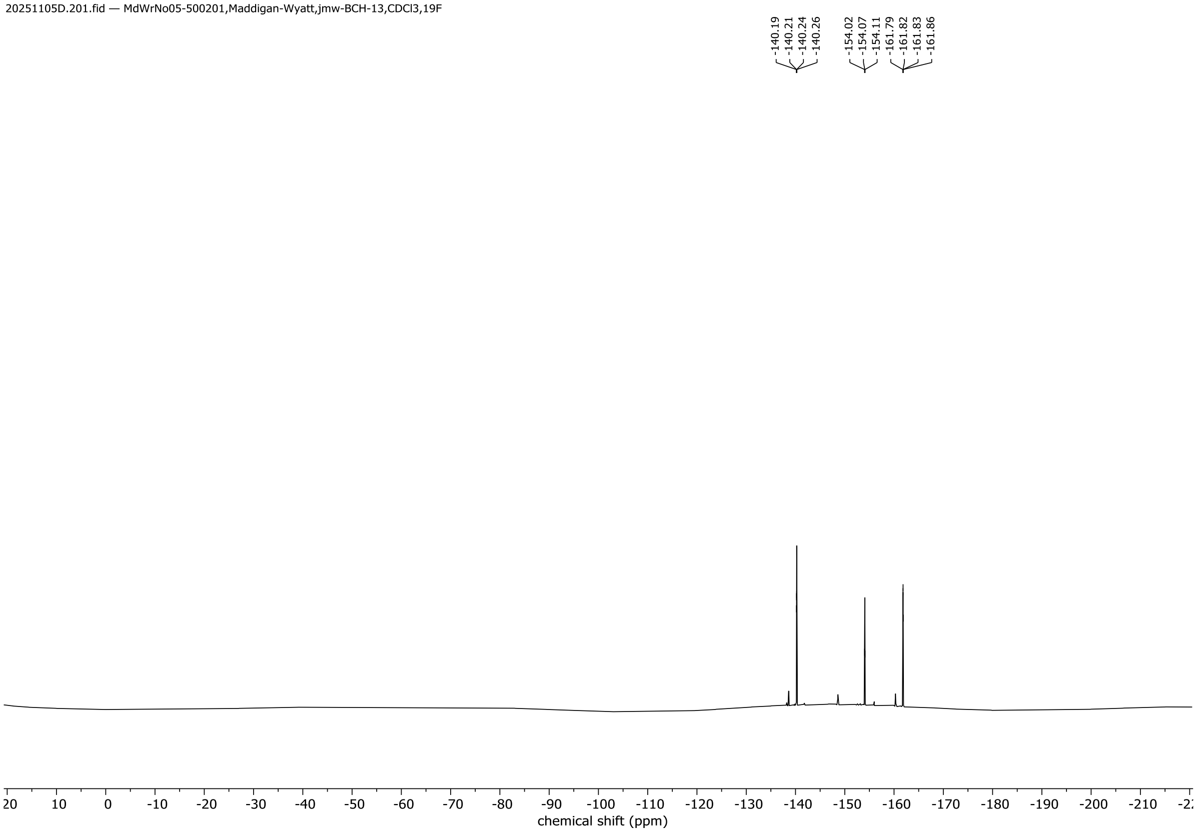

Ethyl 1,3-diphenyl-2-oxabicyclo[2.1.1]hexane-4-carboxylate (3f)

[^{1}H-NMR: 700 MHz, ^{13}C-NMR: 176 MHz]

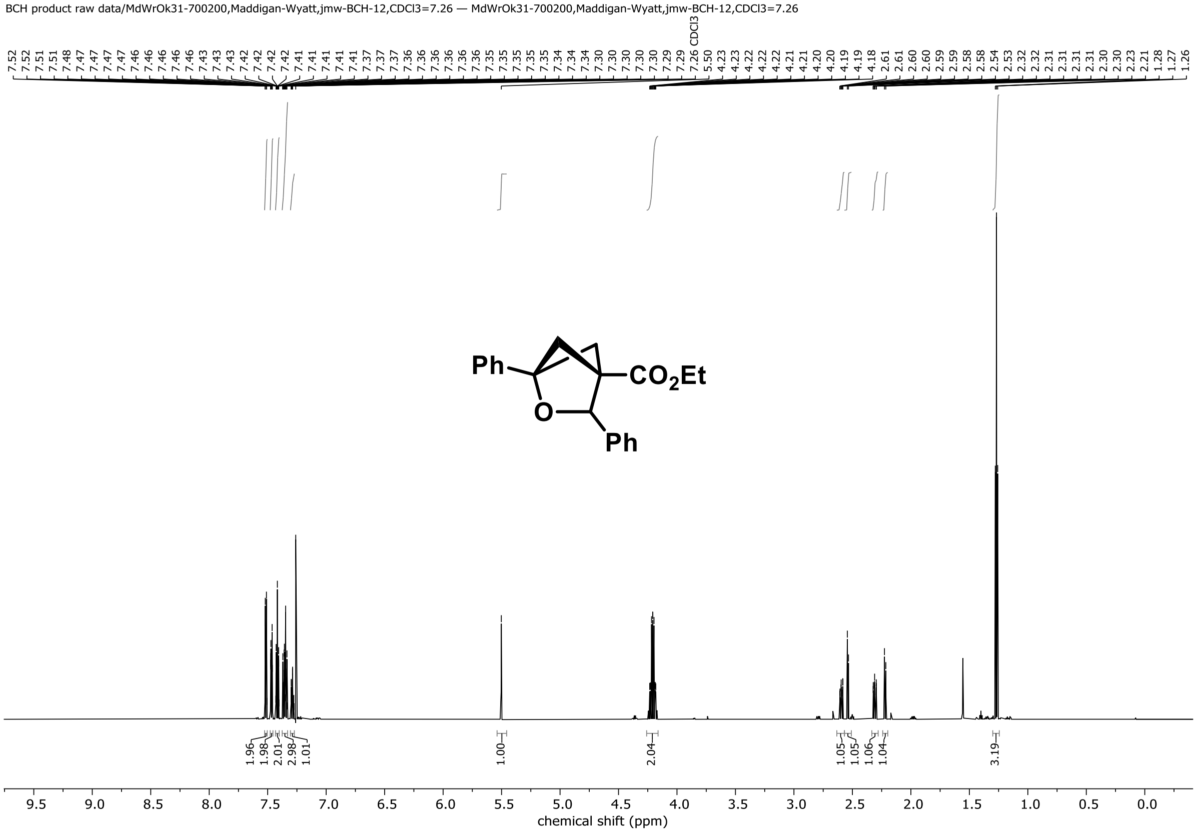

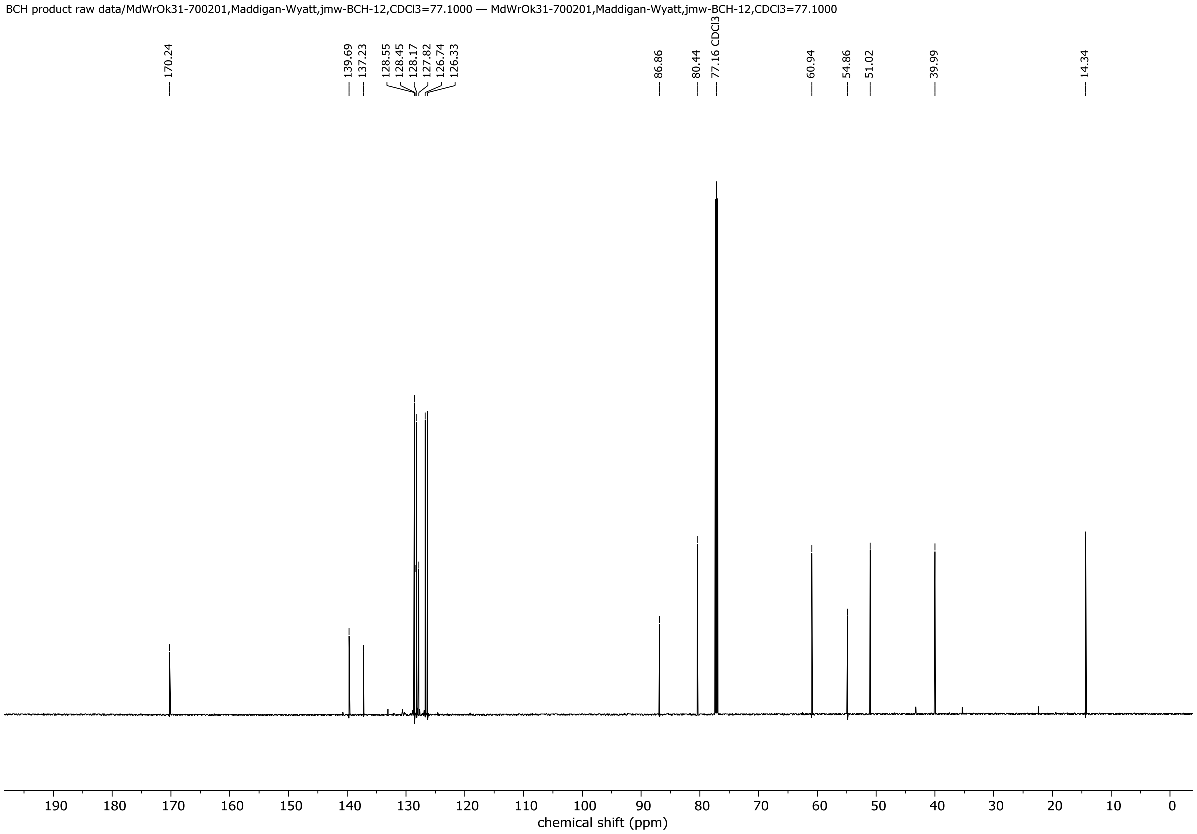

(1,3-Diphenyl-2-oxabicyclo[2.1.1]hexan-4-yl)(phenyl)methanone (3g)

[^{1}H-NMR: 300 MHz, ^{13}C-NMR: 176 MHz]

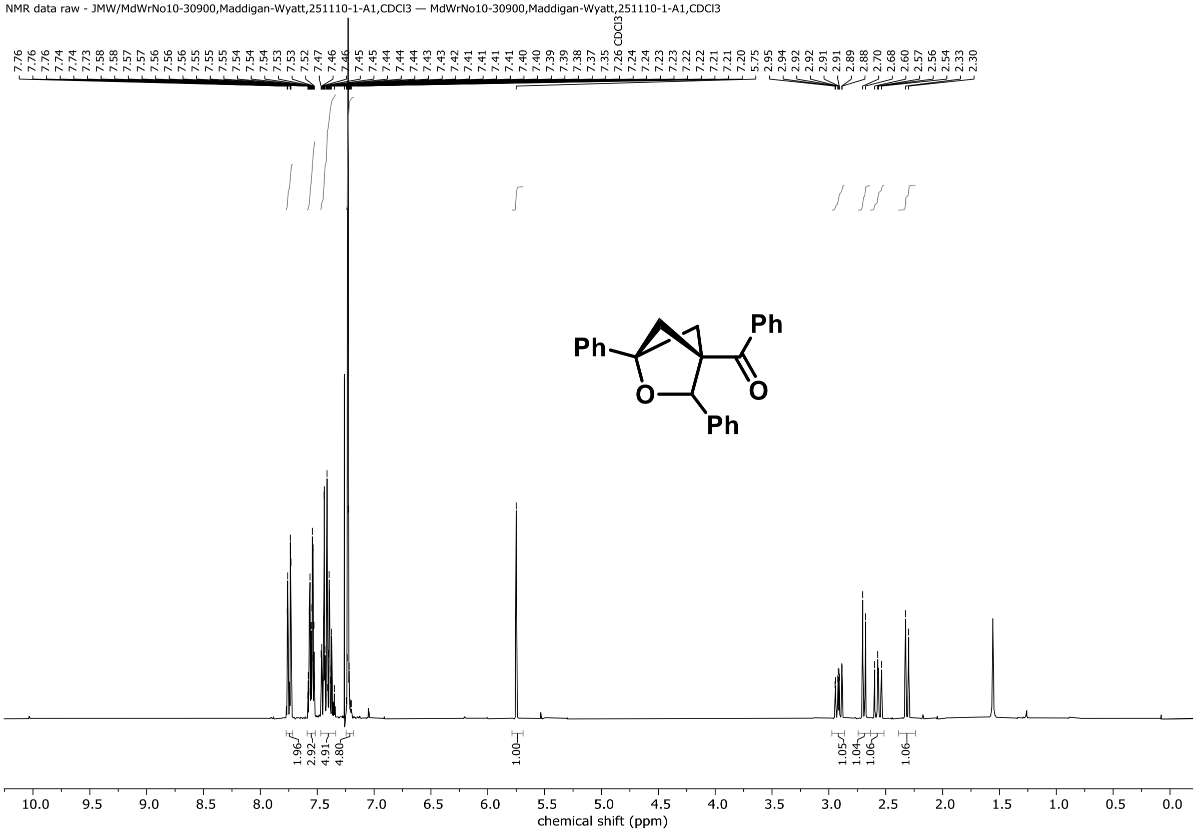

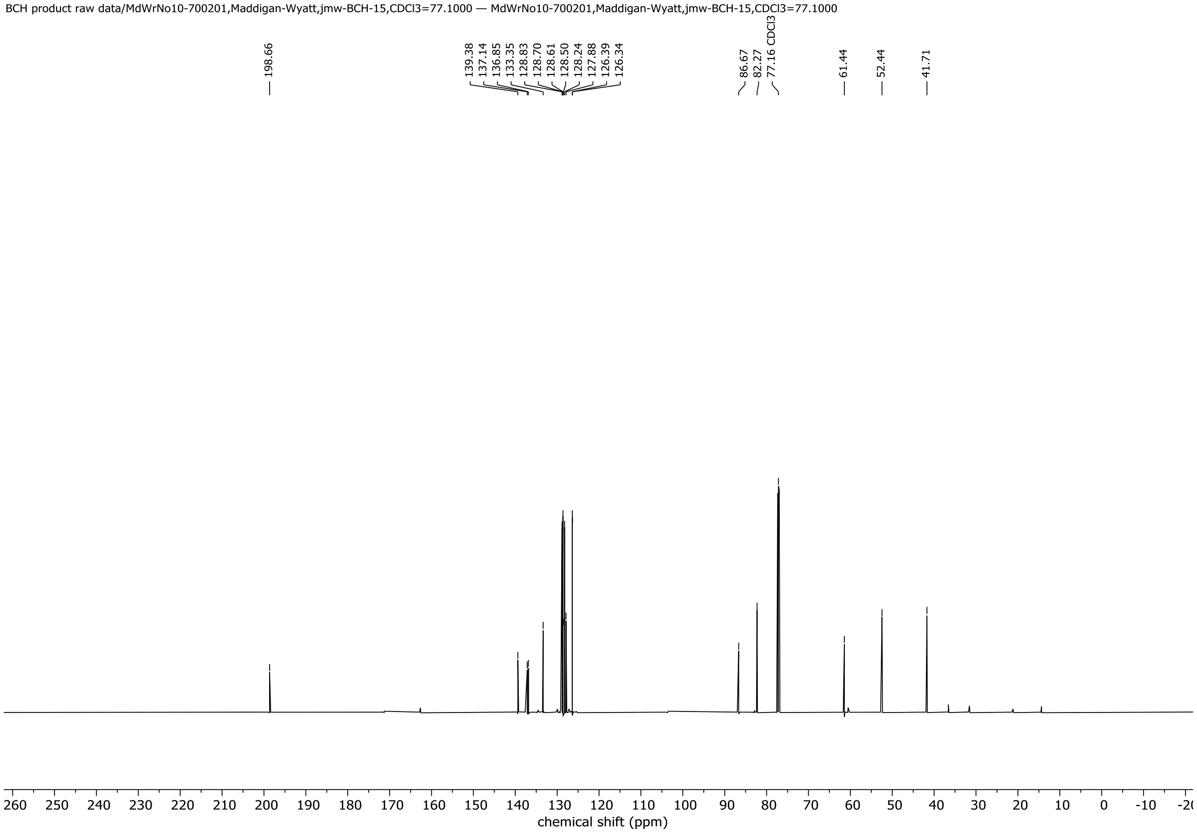

(1-(4-Fluorophenyl)-3-phenyl-2-oxabicyclo[2.1.1]hexan-4-yl)(phenyl)methanone (3h)

[^{1}H-NMR: 700 MHz, ^{13}C-NMR: 176 MHz, ^{19}F-NMR: 282 MHz]

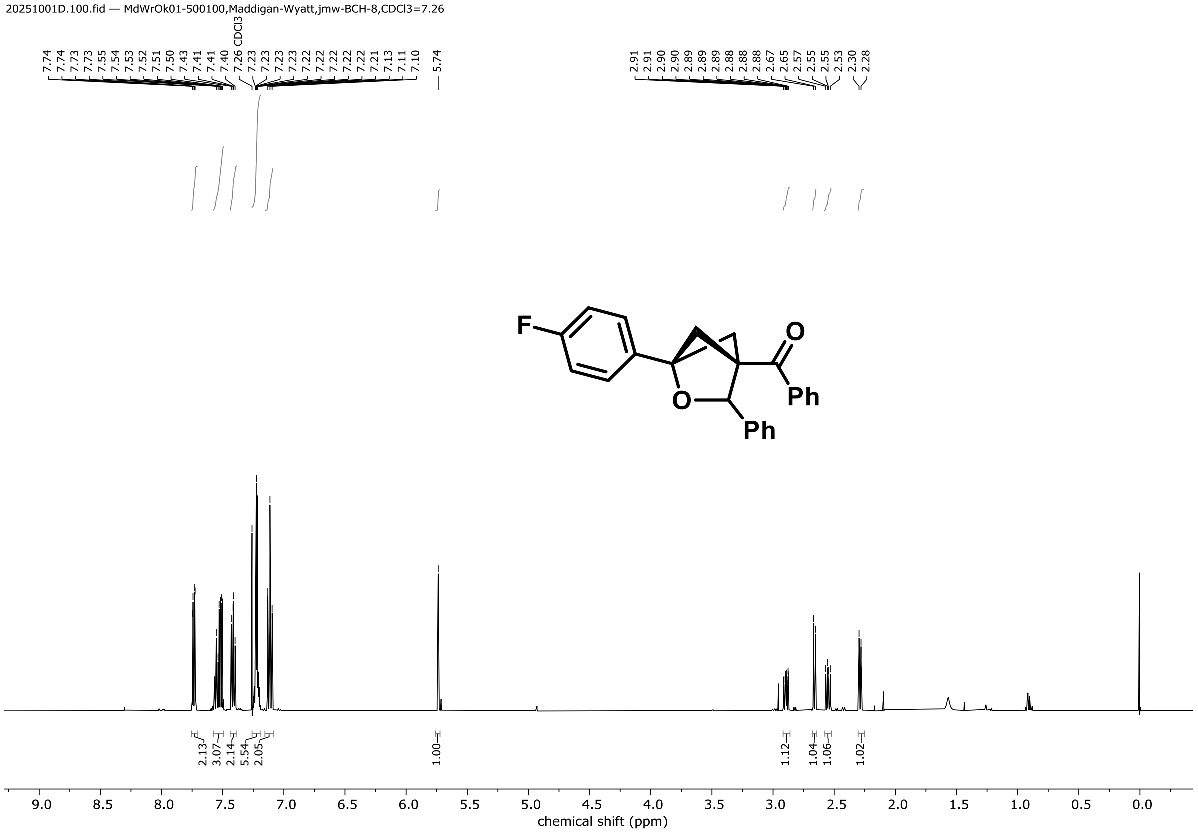

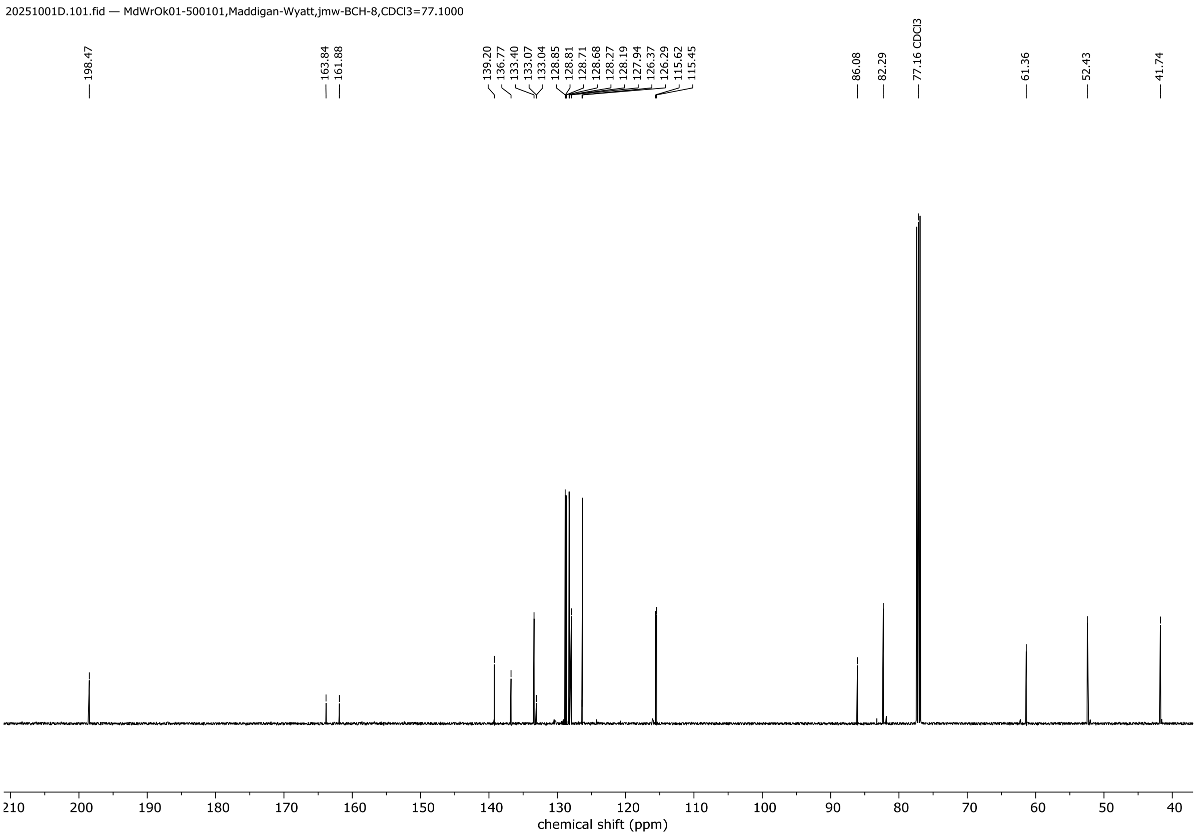

NMR data raw - JMW/MdWrDe05-30160,Maddigan-Wyatt,BCH-8-19F,CDCl3 — MdWrDe05-30160,Maddigan-Wyatt,BCH-8-19F,CDCl3

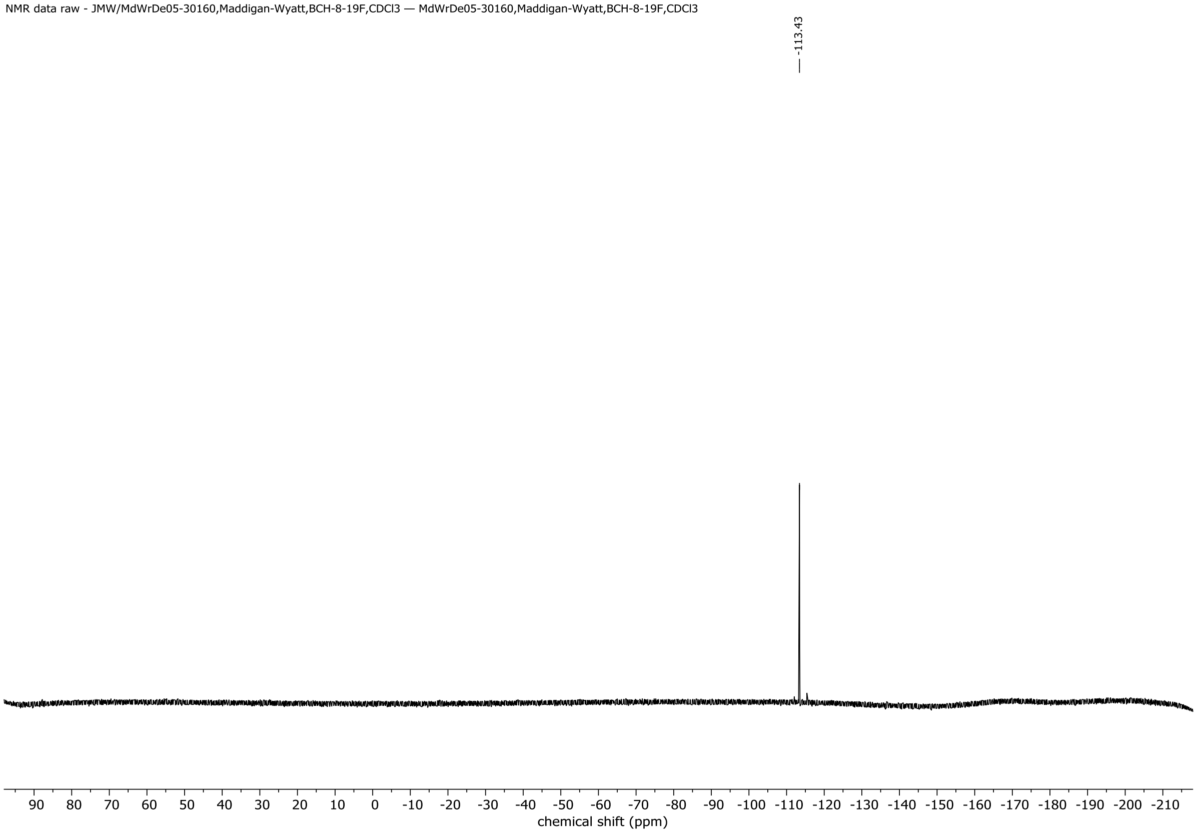

(1,3-Diphenyl-2-oxabicyclo[2.1.1]hexan-4-yl)(naphthalen-2-yl)methanone (3i)
[^{1}H-NMR: 700 MHz, ^{13}C-NMR: 176 MHz]

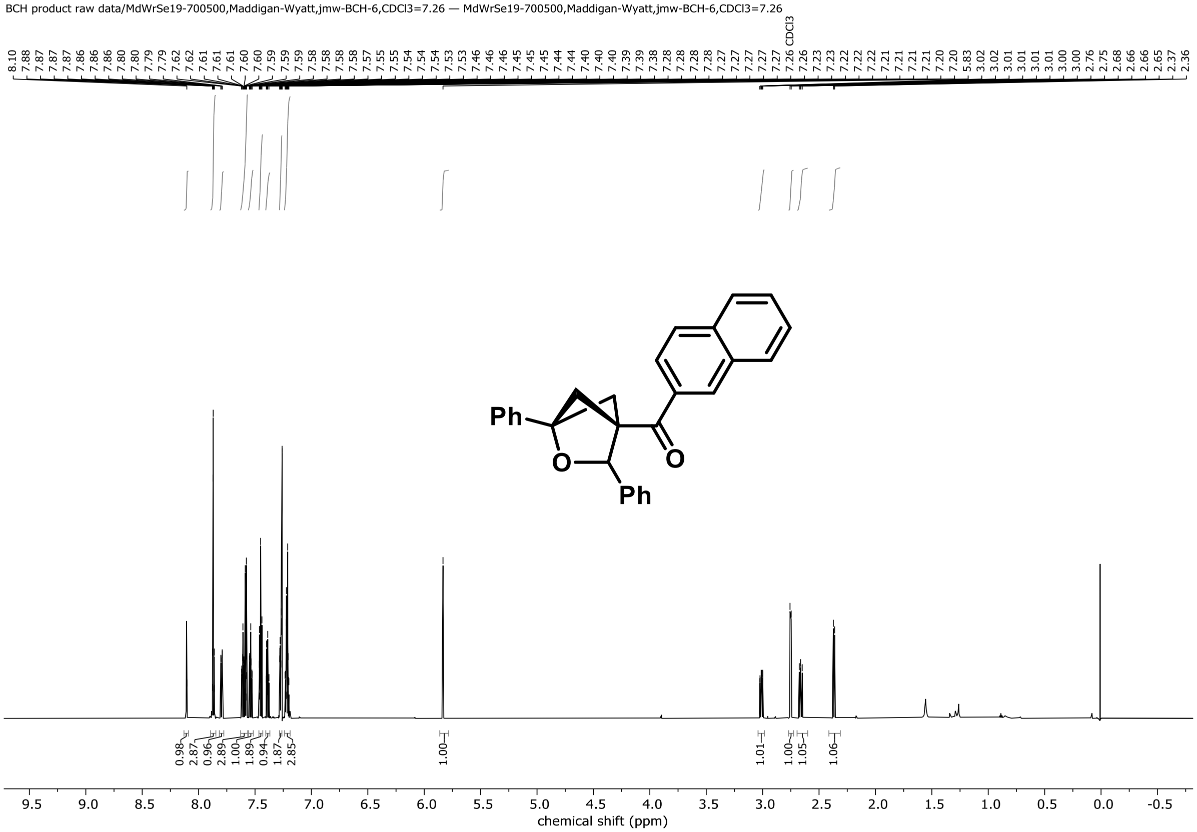

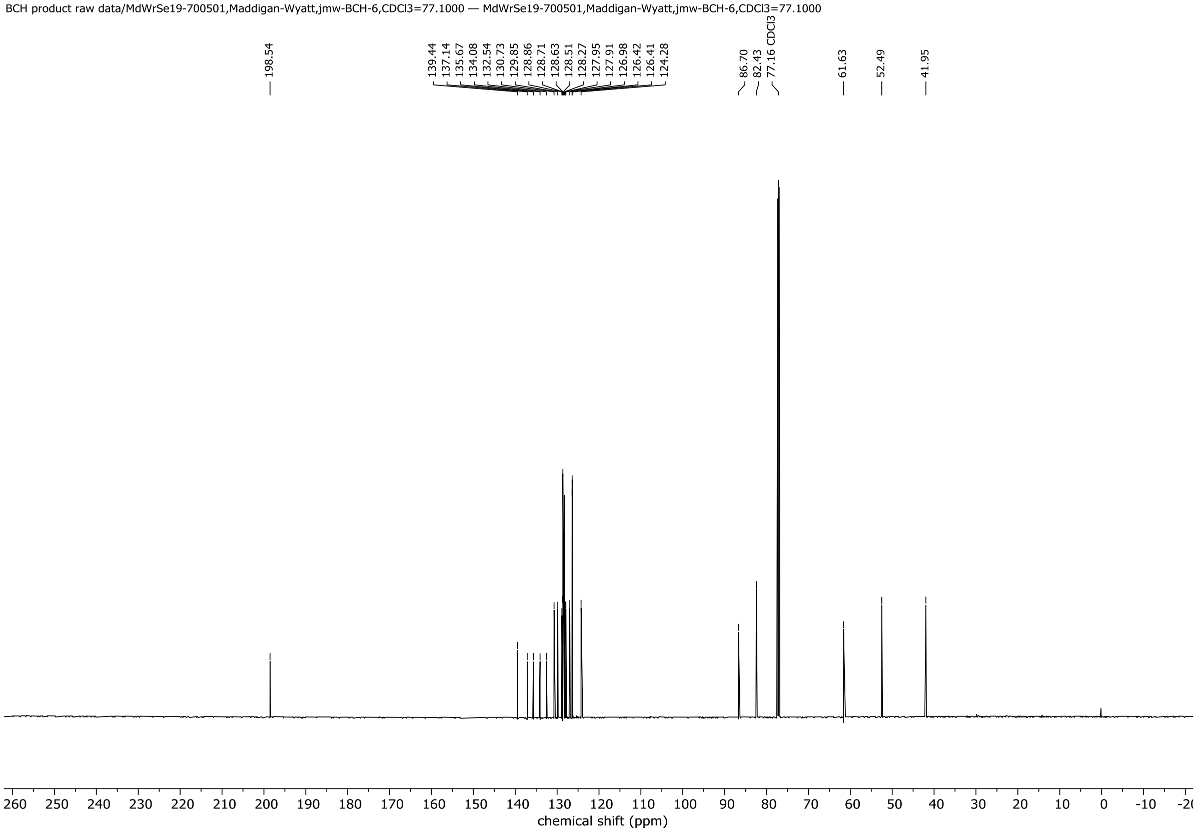

(4-Chlorophenyl)(1,3-diphenyl-2-oxabicyclo[2.1.1]hexan-4-yl)methanone (3j)

[^{1}H-NMR: 700 MHz, ^{13}C-NMR: 176 MHz]

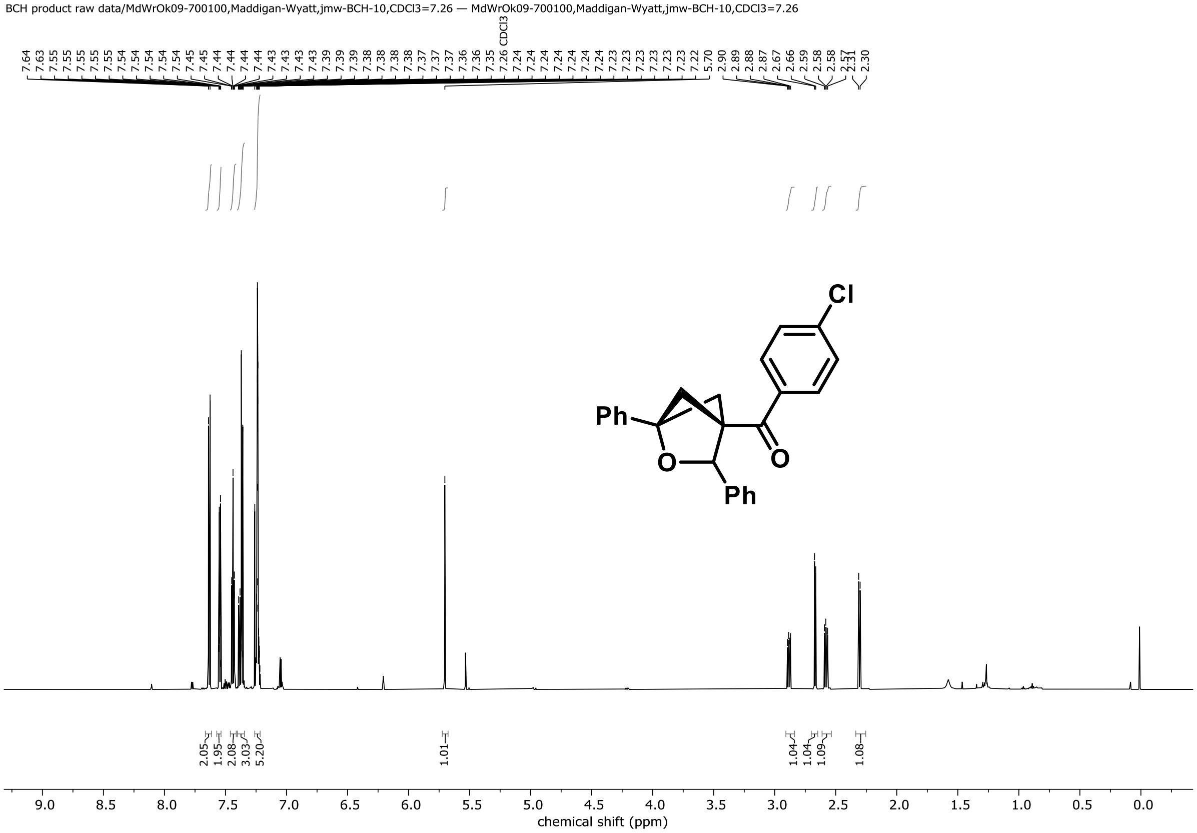

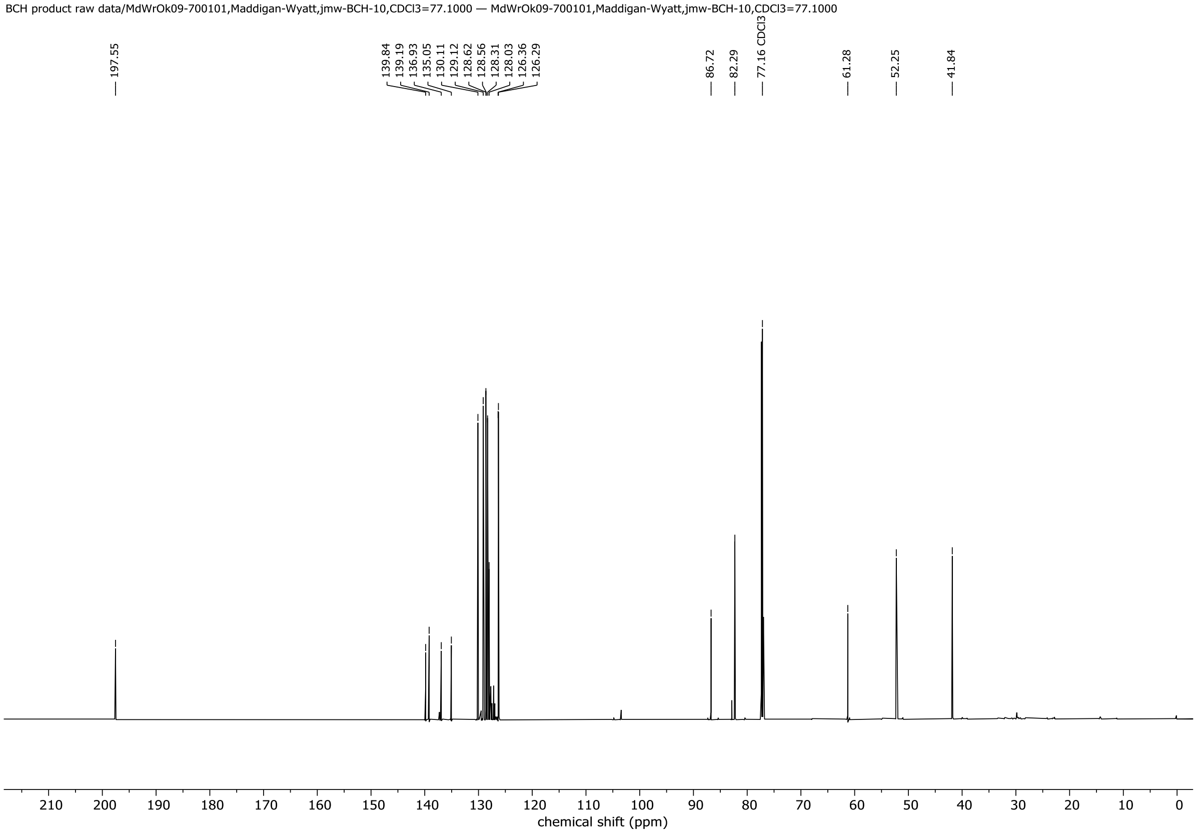

(1,3-Diphenyl-2-oxabicyclo[2.1.1]hexan-4-yl)(4-(trifluoromethyl)phenyl)methanone (3k)

[^{1}H-NMR: 700 MHz, ^{13}C-NMR: 176 MHz, ^{19}F-NMR: 659 MHz]

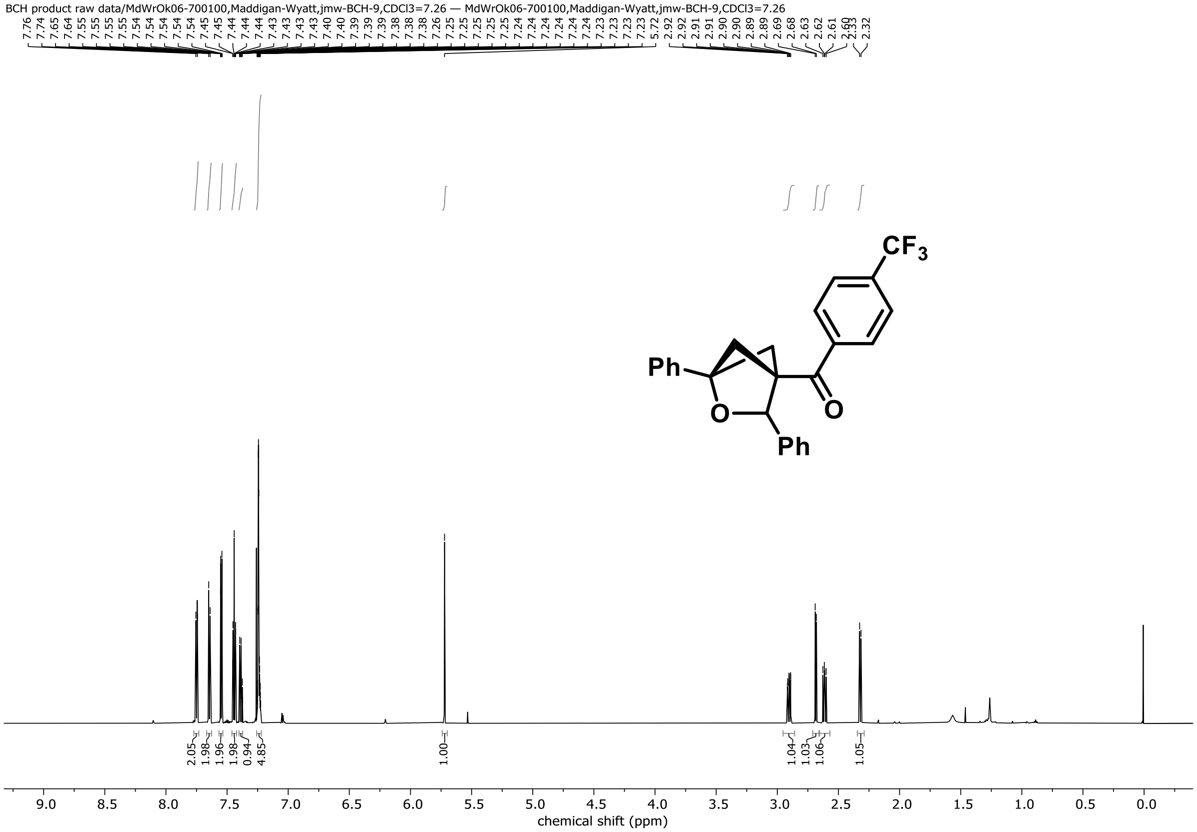

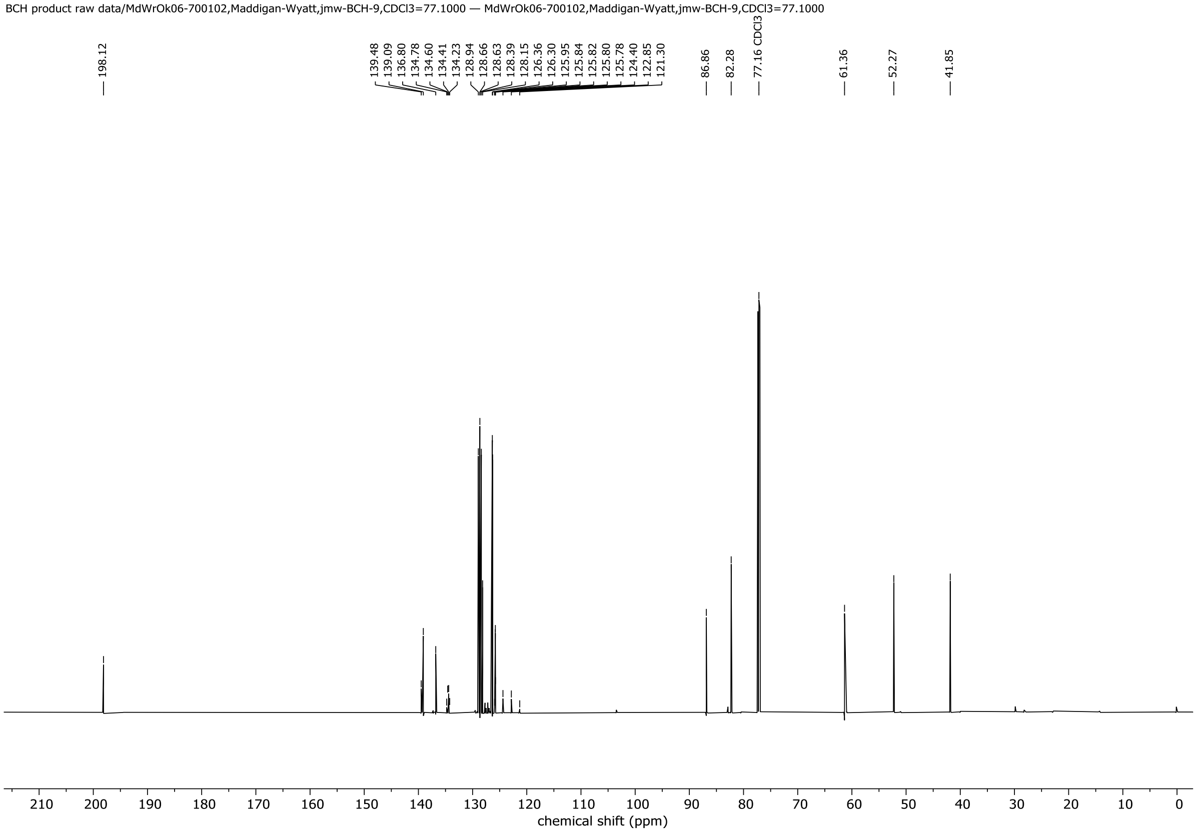

BCH product raw data/MdWrOk06-700101,Maddigan-Wyatt,jmw-BCH-9,CDCl3,19F — MdWrOk06-700101,Maddigan-Wyatt,jmw-BCH-9,CDCl3,19F

^{19}F NMR (659 MHz, $CDCl_3$) δ -63.21.

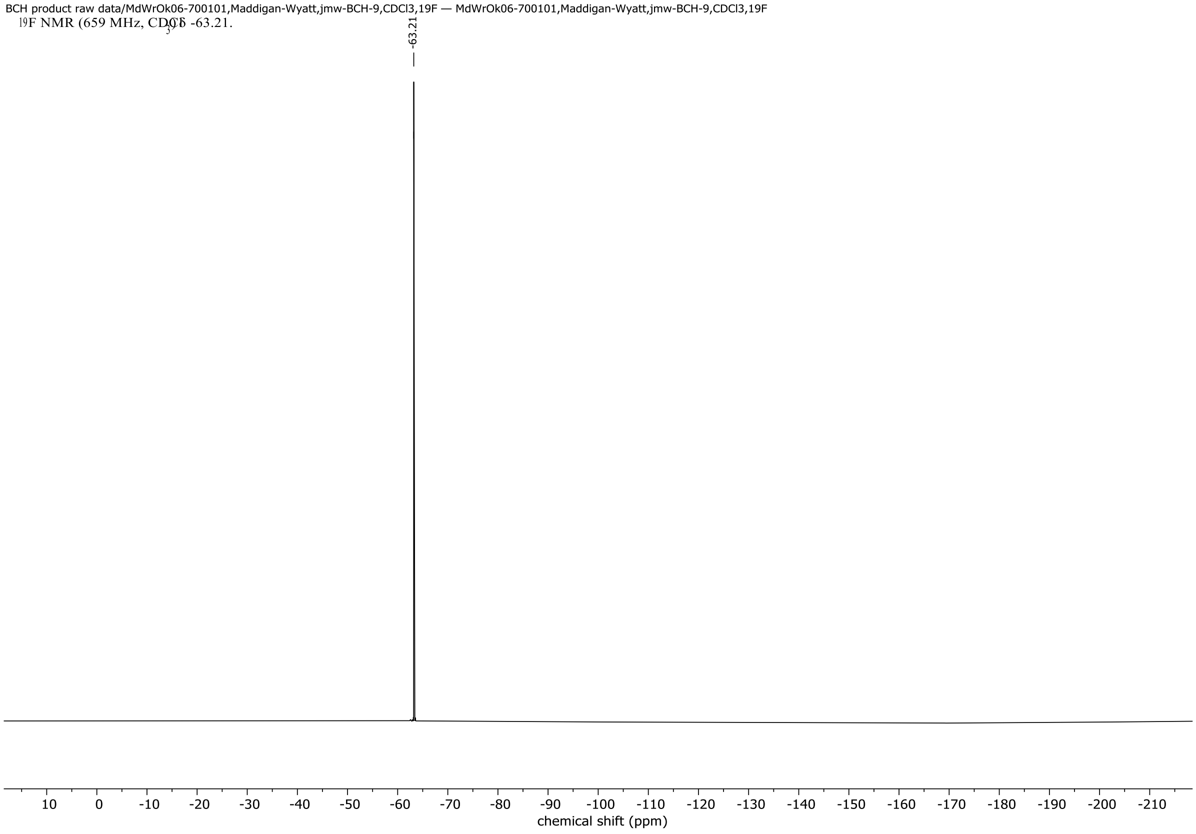

(1,3-Diphenyl-2-oxabicyclo[2.1.1]hexan-4-yl)(p-tolyl)methanone (3l)

[^{1}H-NMR: 700 MHz, ^{13}C-NMR: 176 MHz]

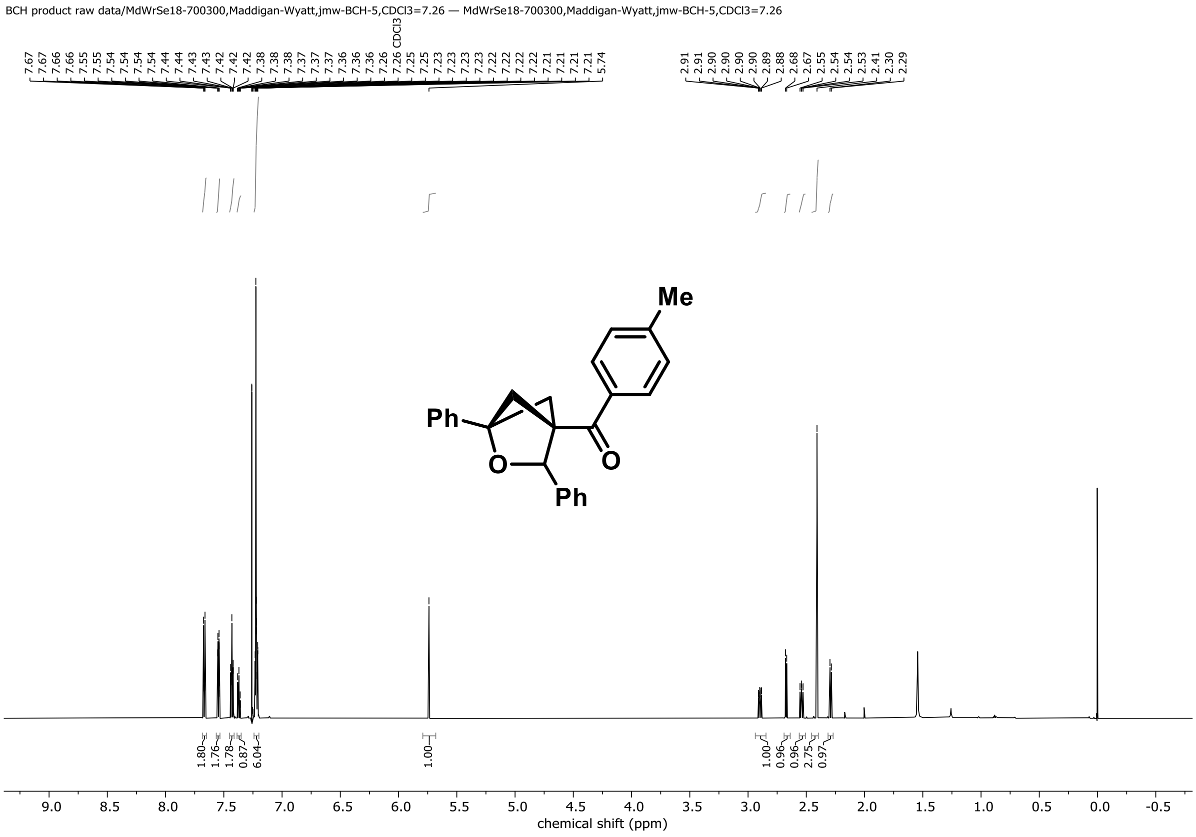

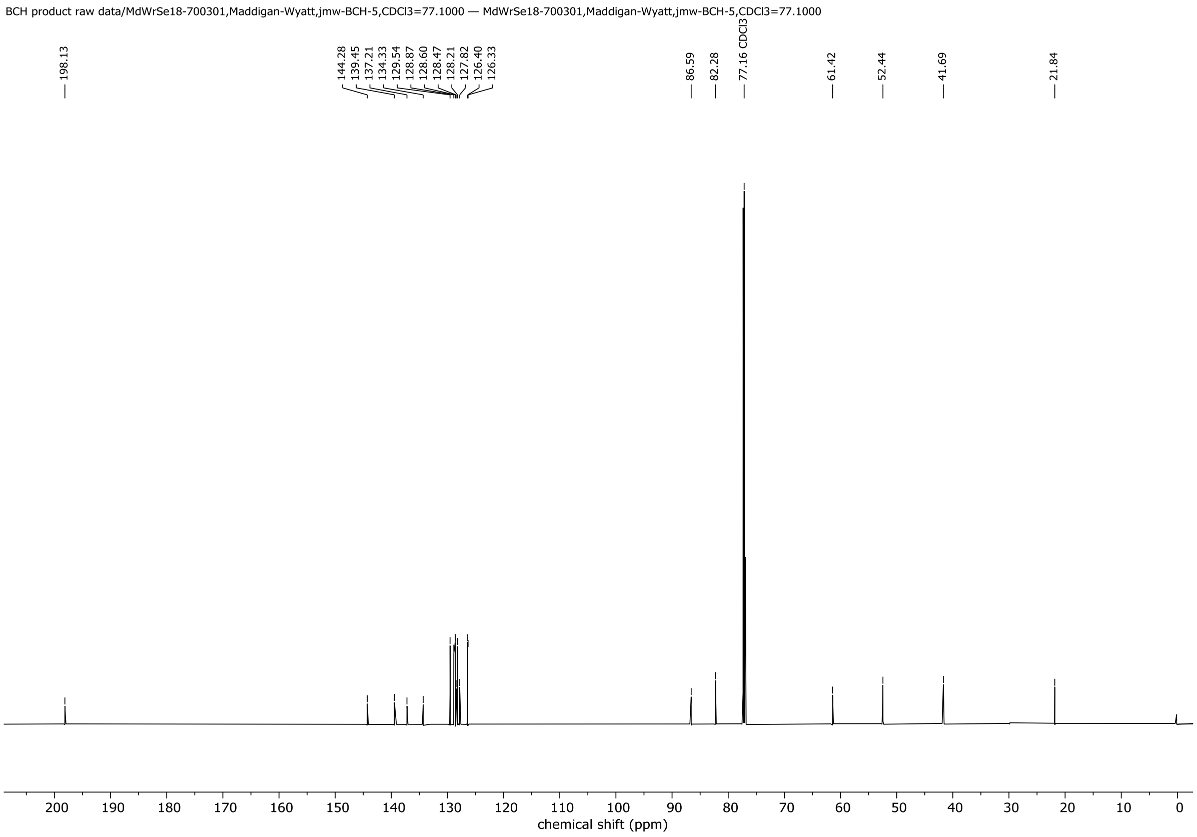

(1-Phenyl-3-(p-tolyl)-2-oxabicyclo[2.1.1]hexan-4-yl)(p-tolyl)methanone (3m)
[^{1}H-NMR: 700 MHz, ^{13}C-NMR: 176 MHz]

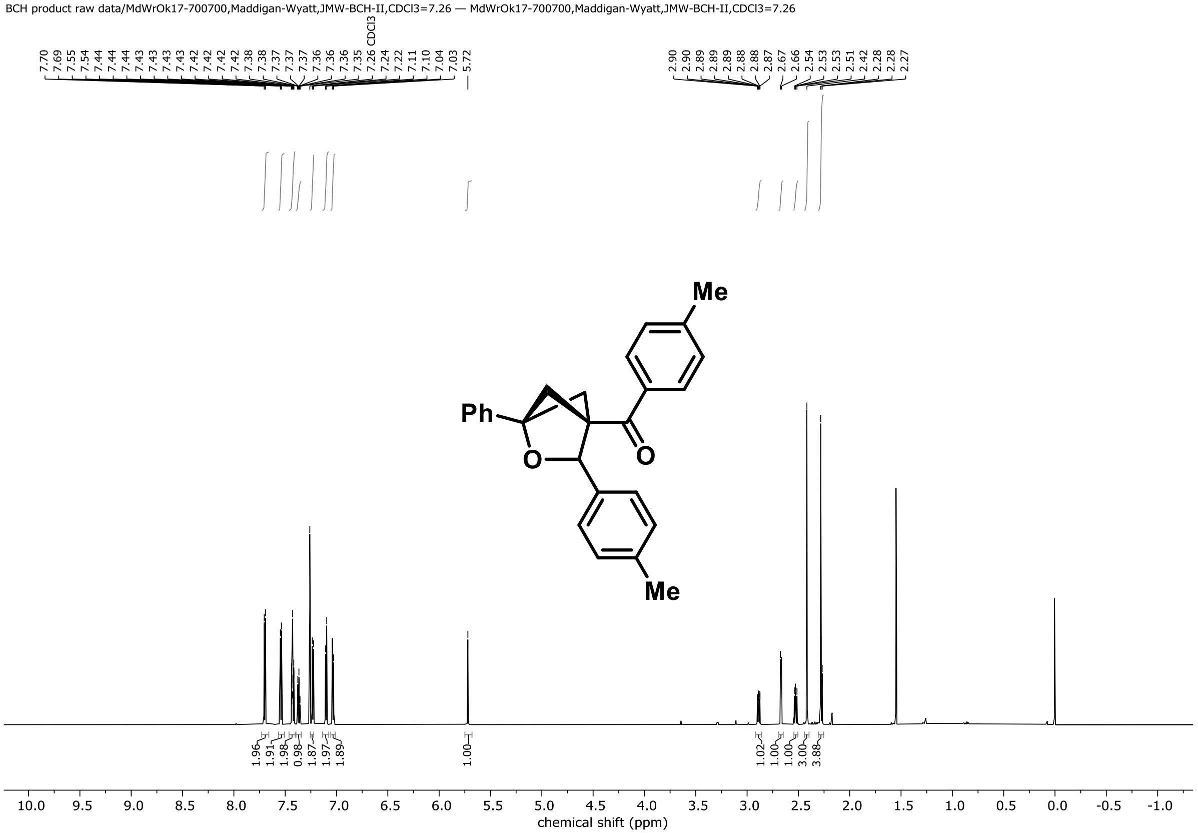

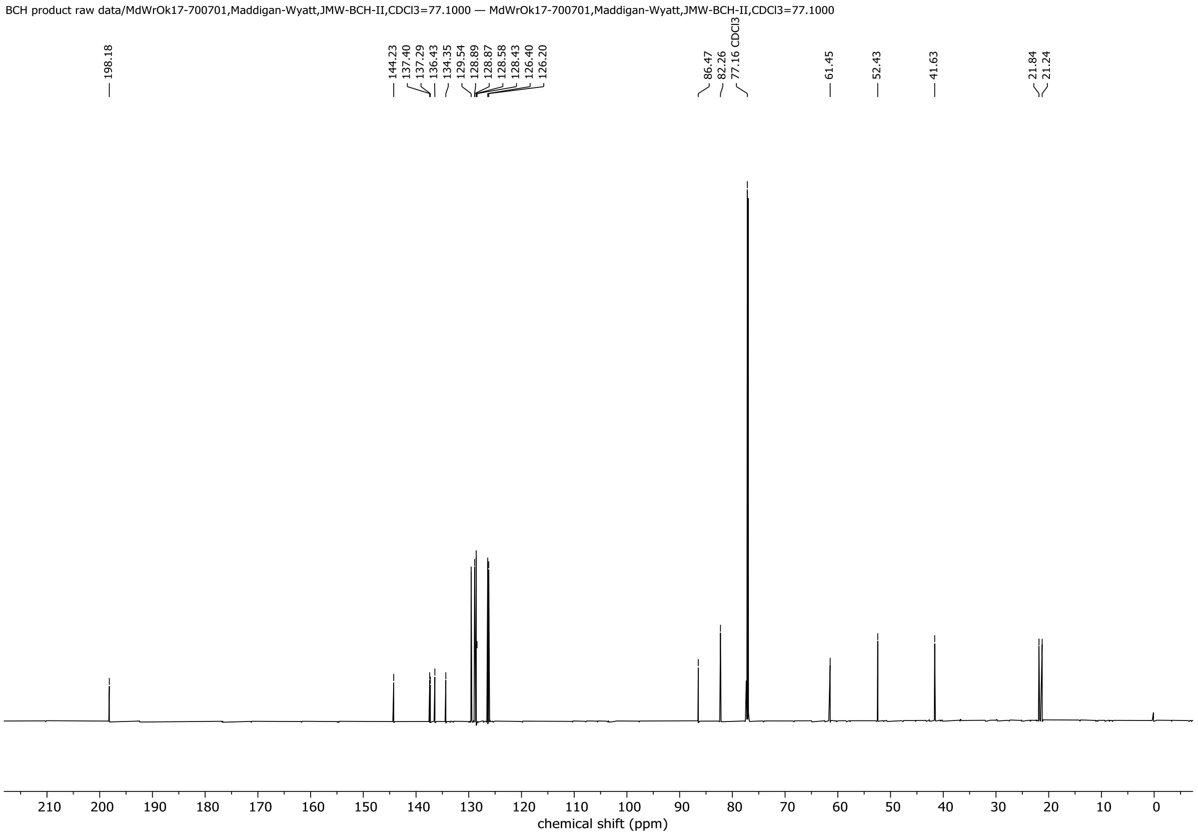

(3-(4-Methoxyphenyl)-1-phenyl-2-oxabicyclo[2.1.1]hexan-4-yl)(p-tolyl)methanone (3n)
[^{1}H-NMR: 700 MHz, ^{13}C-NMR: 176 MHz]

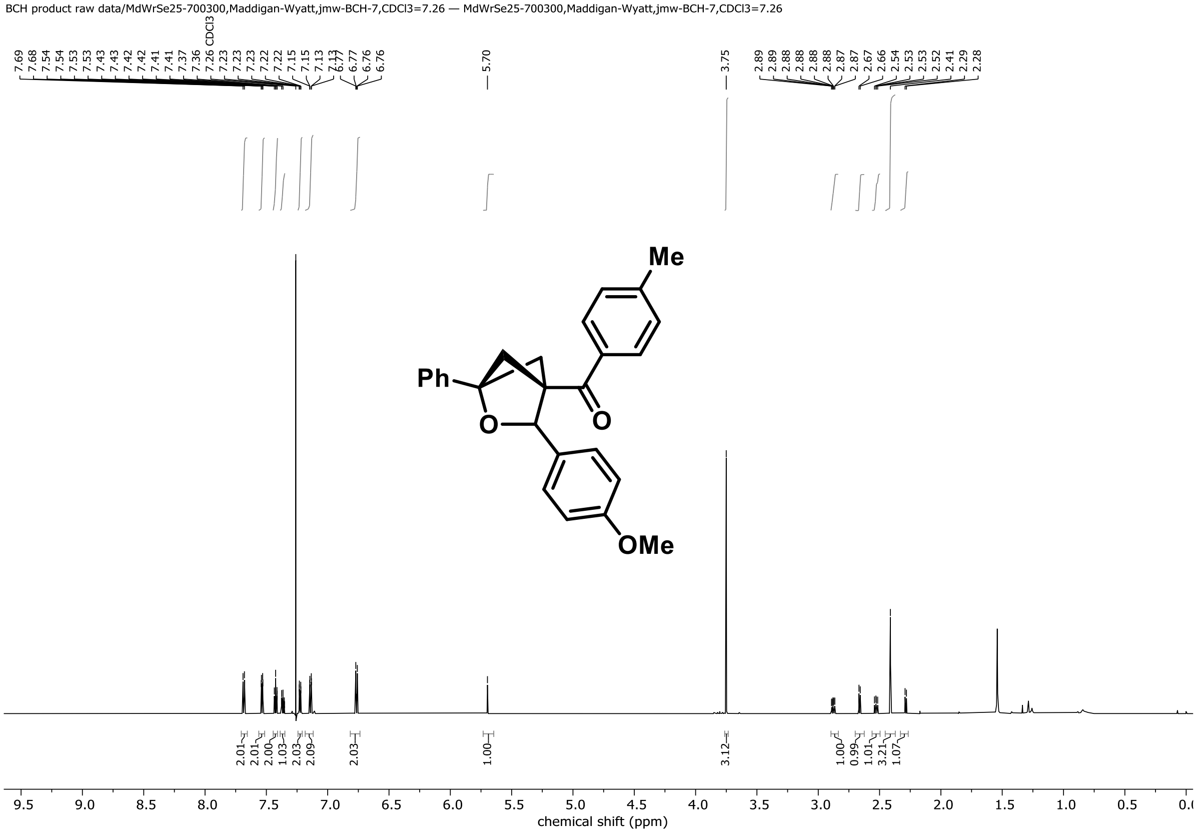

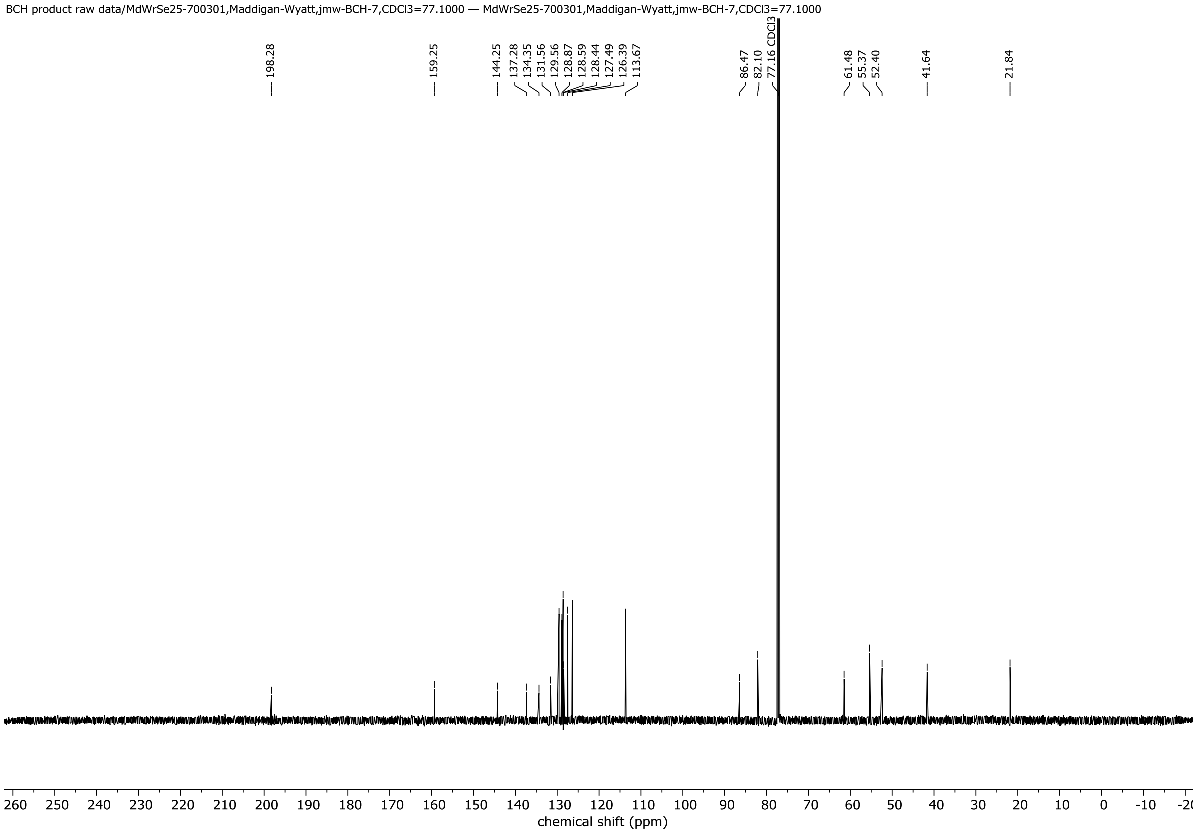

1-(1,3-Diphenyl-2-oxabicyclo[2.1.1]hexan-4-yl)ethan-1-one (3o)

[^{1}H-NMR: 700 MHz, ^{13}C-NMR: 176 MHz]

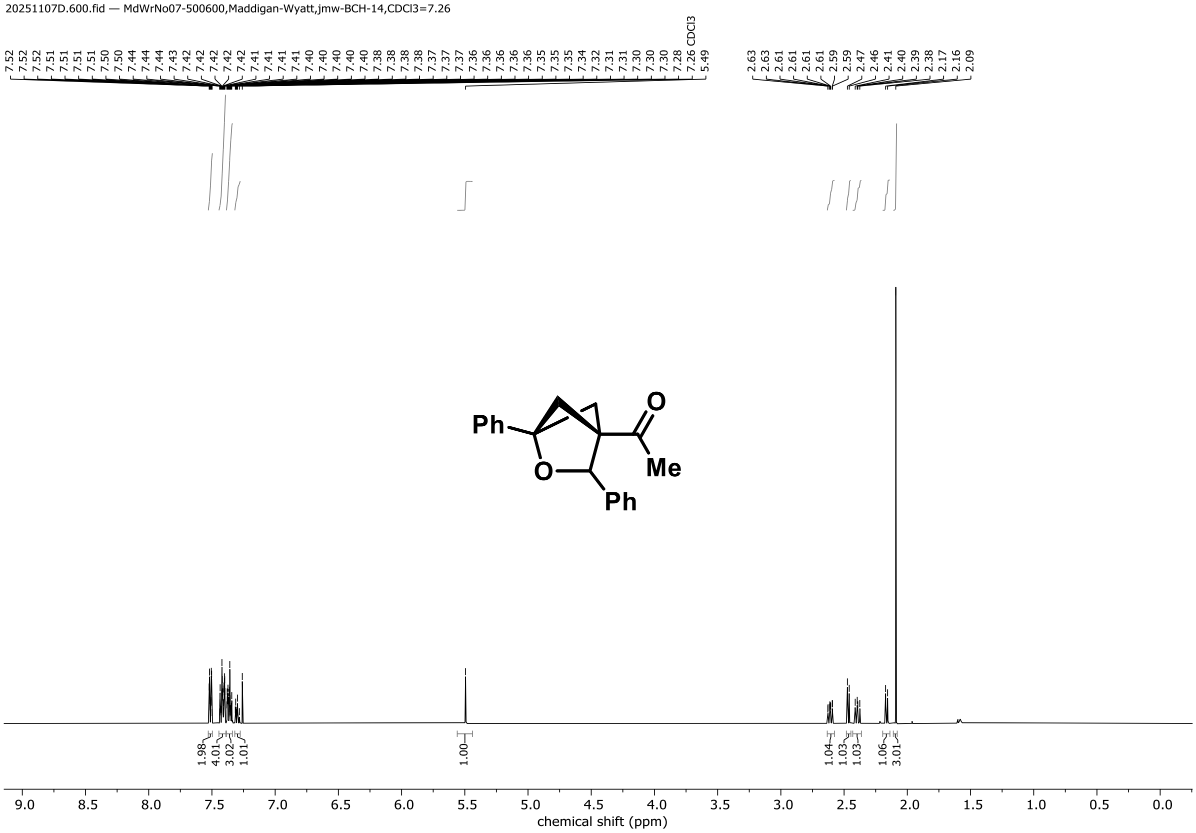

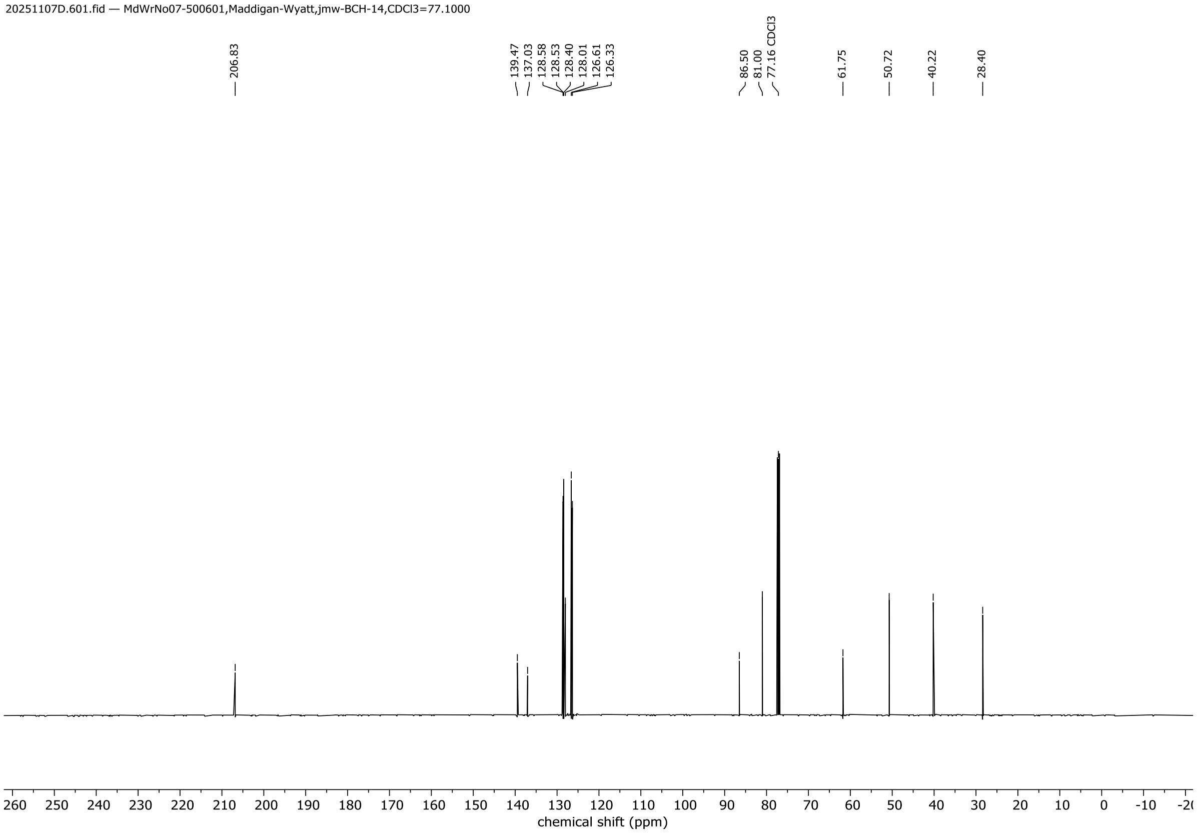

1-(1,3-Diphenyl-2-oxabicyclo[2.1.1]hexan-4-yl)pentan-1-one (3p)

[^{1}H-NMR: 700 MHz, ^{13}C-NMR: 176 MHz]

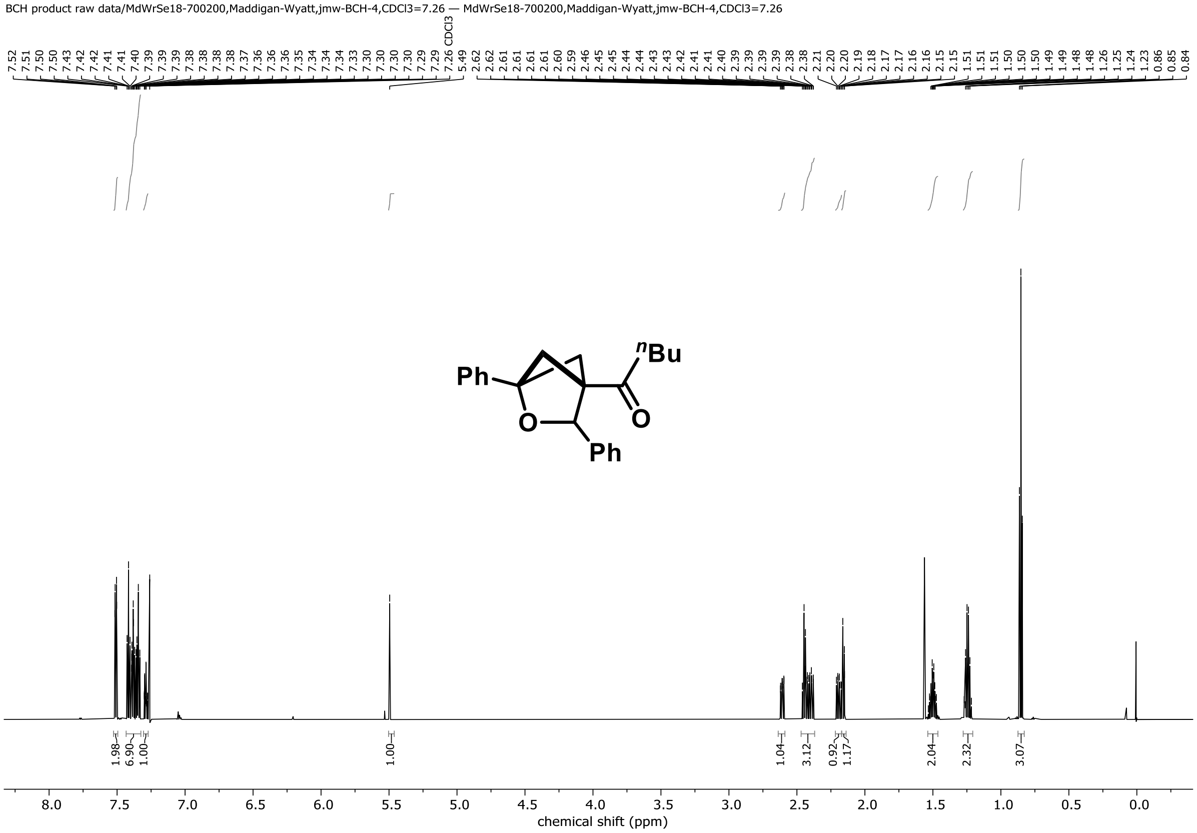

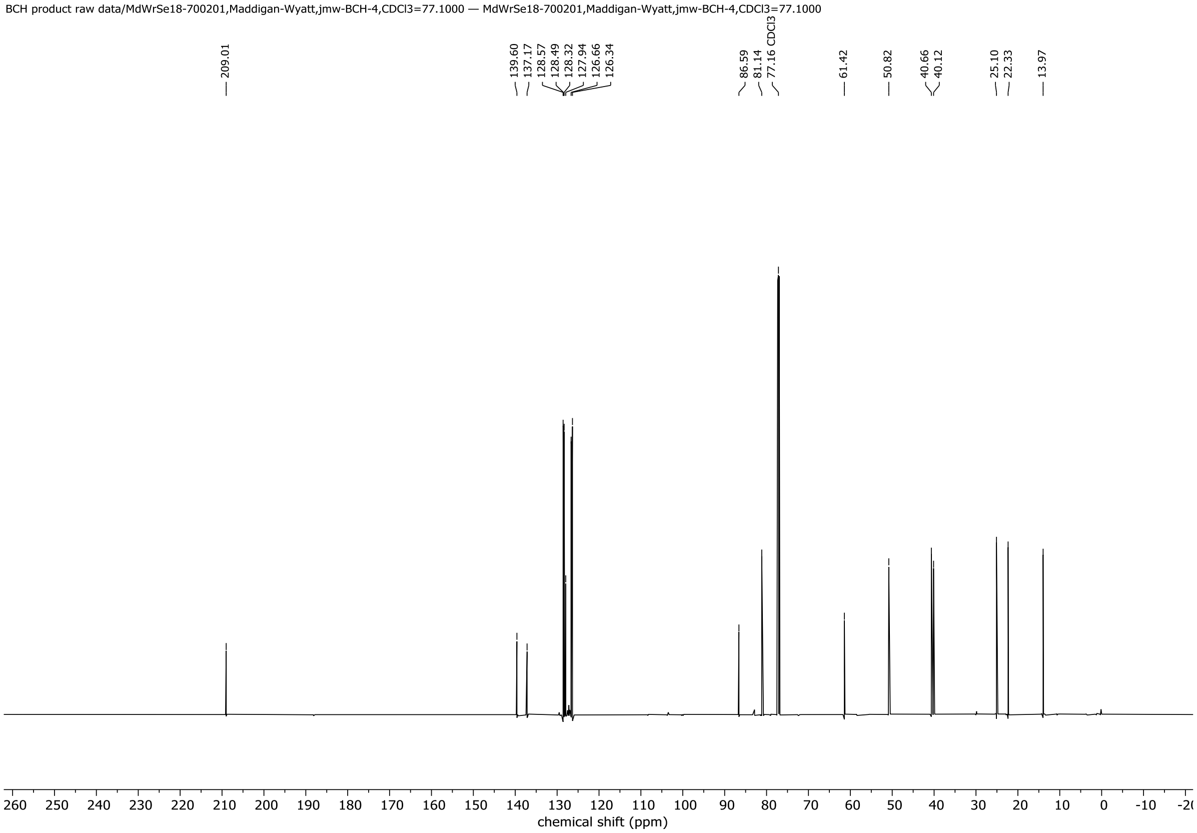

Methyl 3-(4-methoxyphenyl)-3-phenylcyclobutane-1-carboxylate (5a)

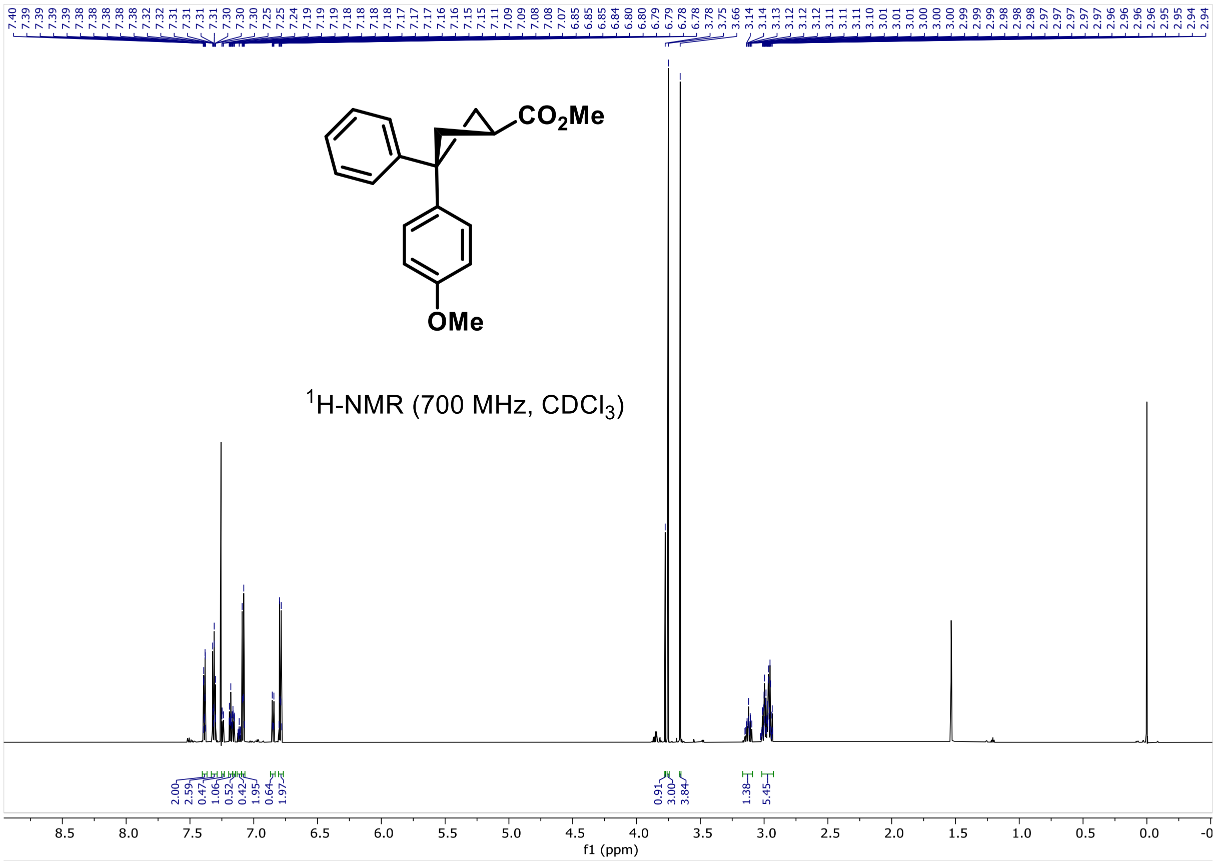

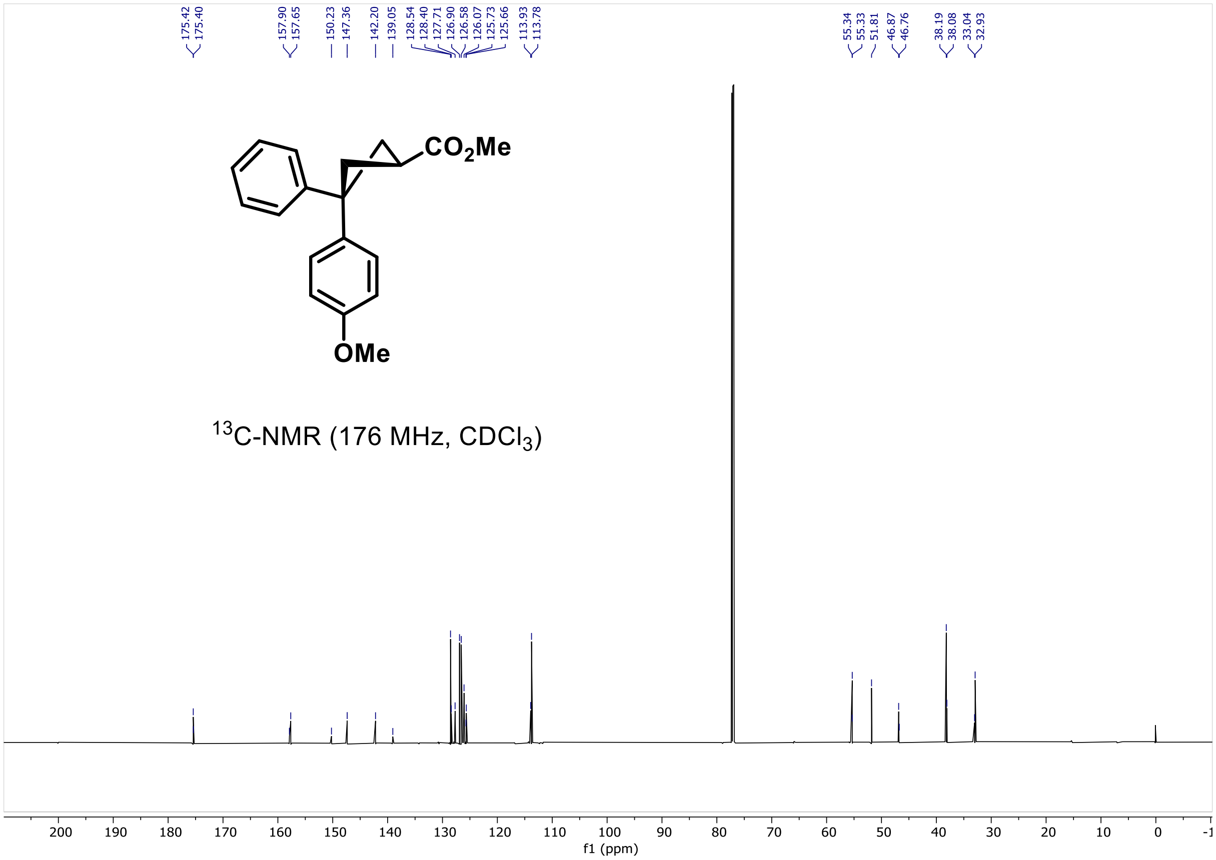

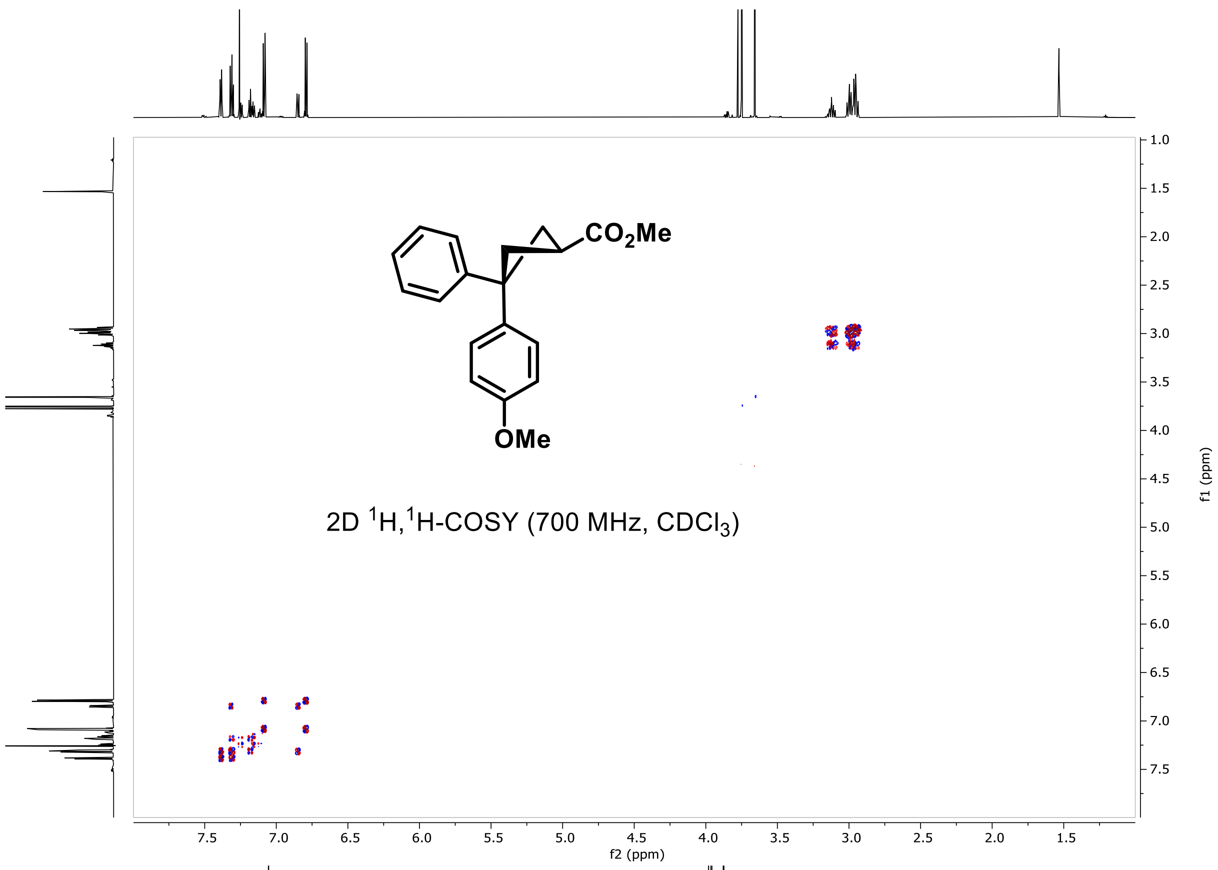
CO2Me
OMe
2D 1H,1H-COSY (700 MHz, CDCl3)
f1 (ppm)
f2 (ppm)

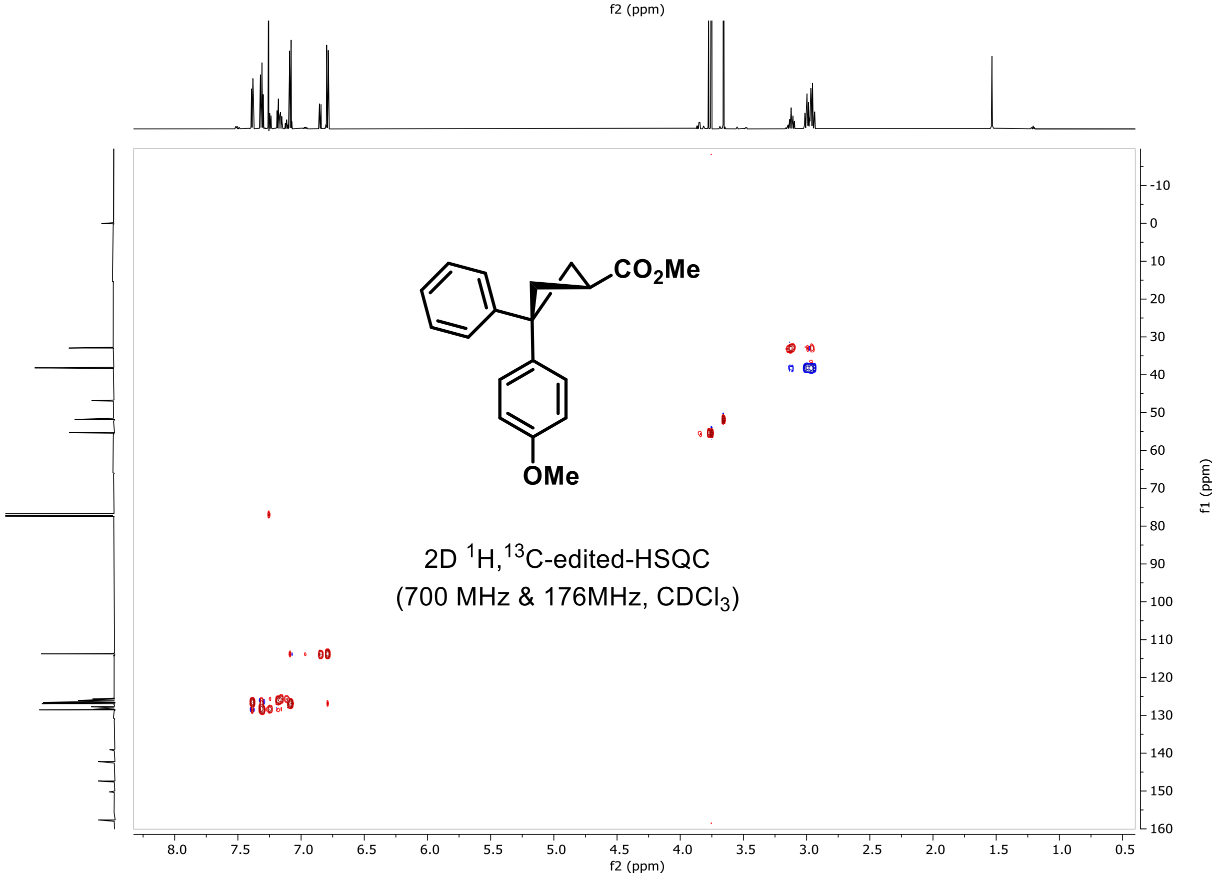
CO2Me
OMe
2D 1H,13C-edited-HSQC
(700 MHz & 176MHz, CDCl3)
f1 (ppm)
f2 (ppm)

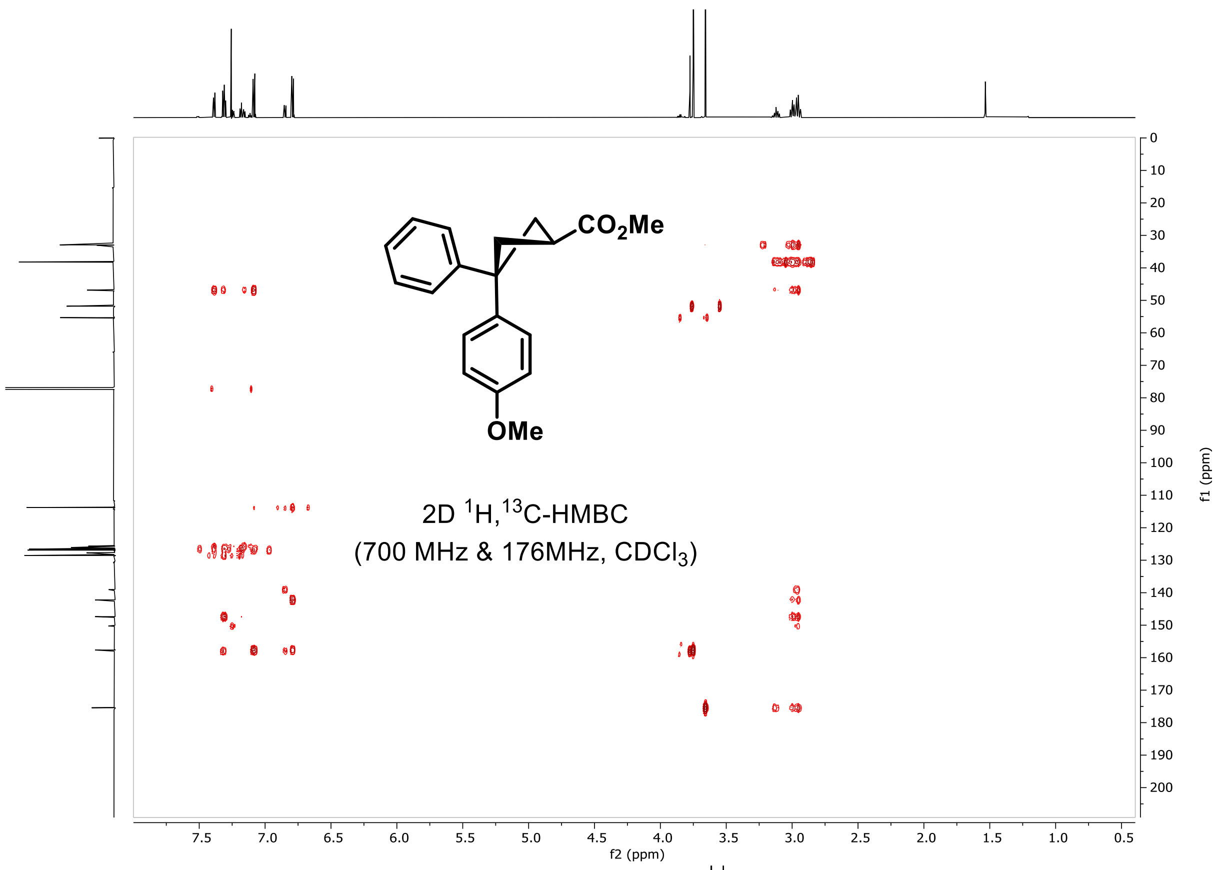

CO2Me
OMe
2D 1H,13C-HMBC
(700 MHz & 176MHz, CDCl3)
f1 (ppm)
f2 (ppm)

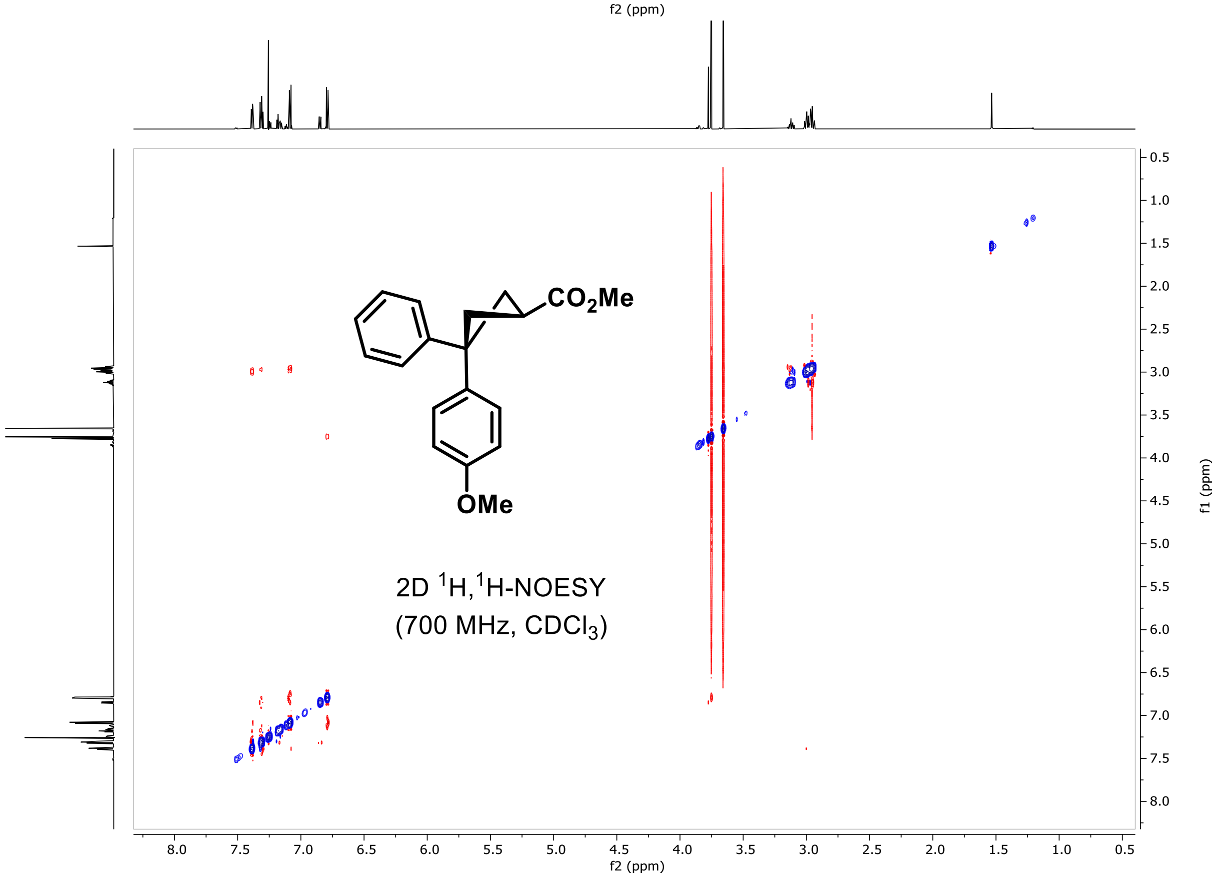

CO2Me
OMe
2D 1H,1H-NOESY
(700 MHz, CDCl3)
f1 (ppm)
f2 (ppm)

Methyl 3-(4-fluorophenyl)-3-(4-methoxyphenyl)cyclobutane-1-carboxylate (5b)
[^{1}H-NMR: 500 MHz, ^{13}C-NMR: 126 MHz, ^{19}F-NMR: 471 MHz]

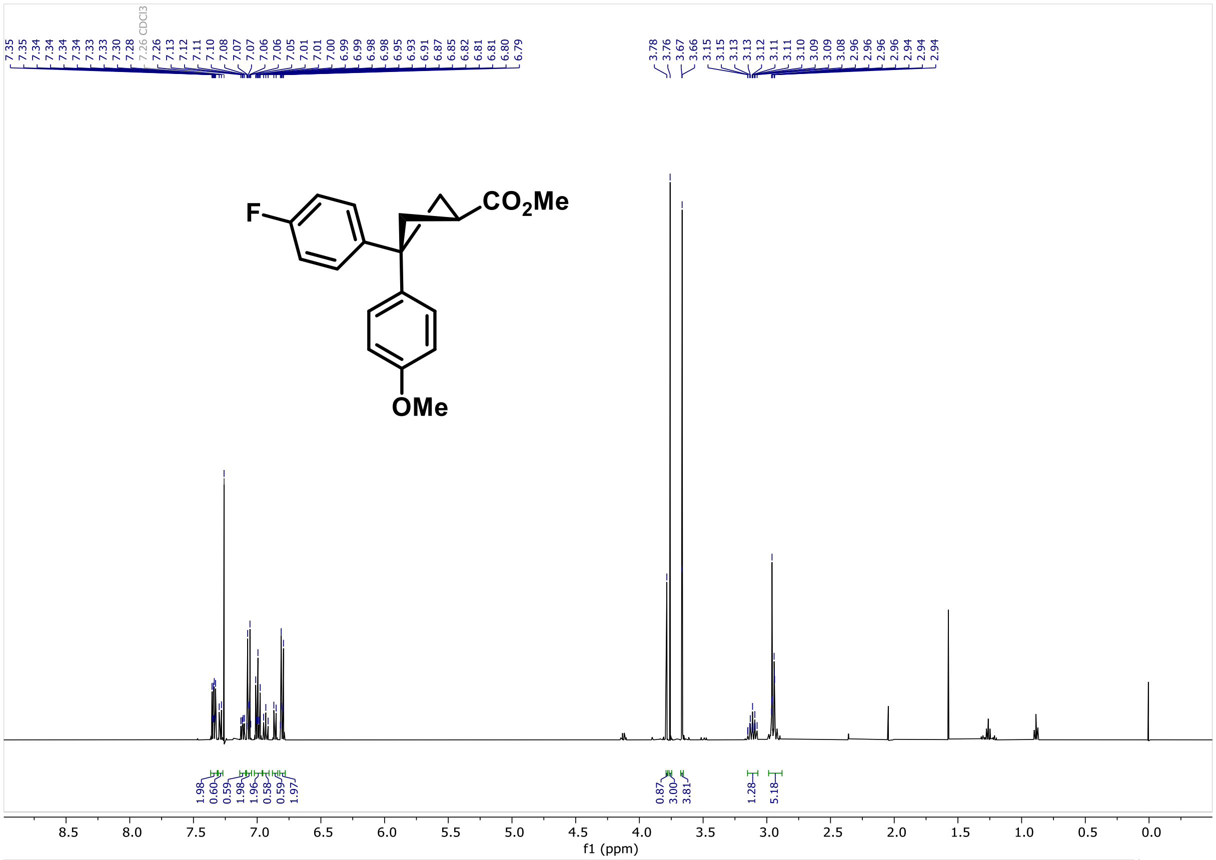

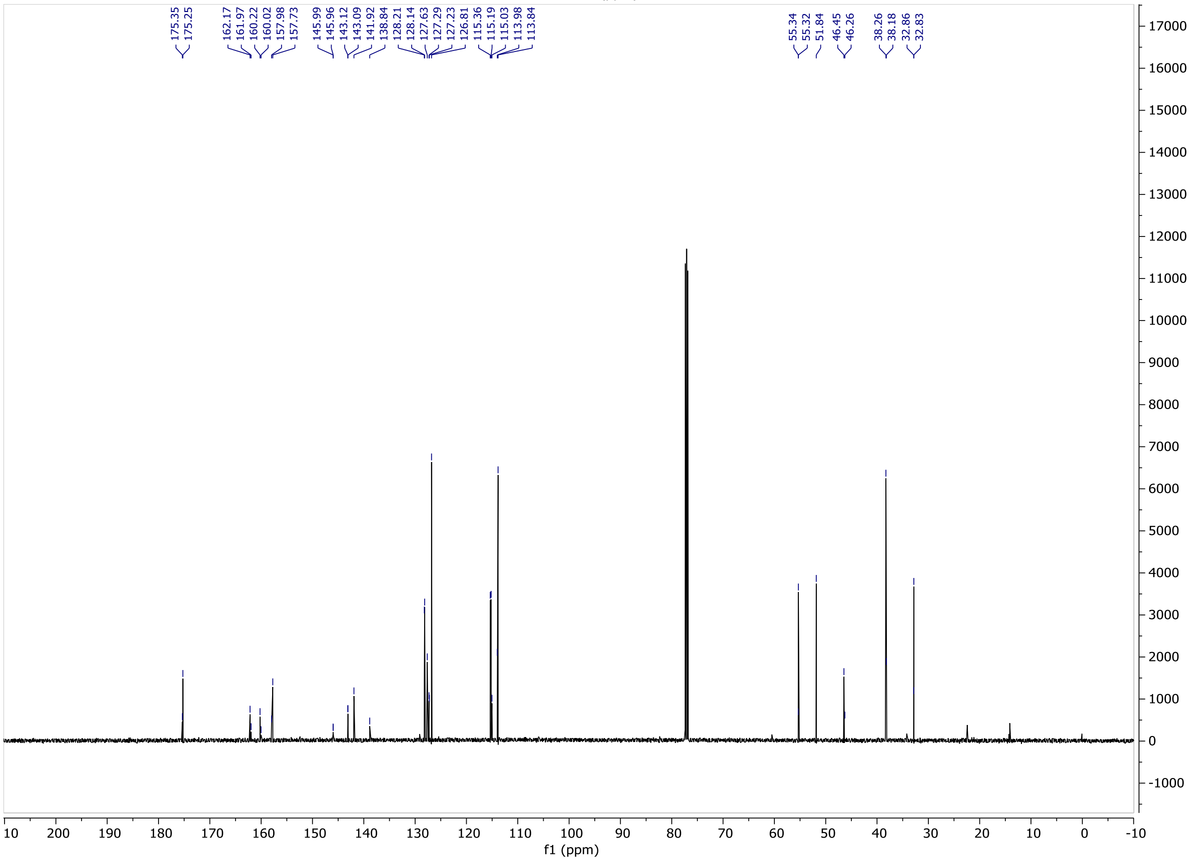

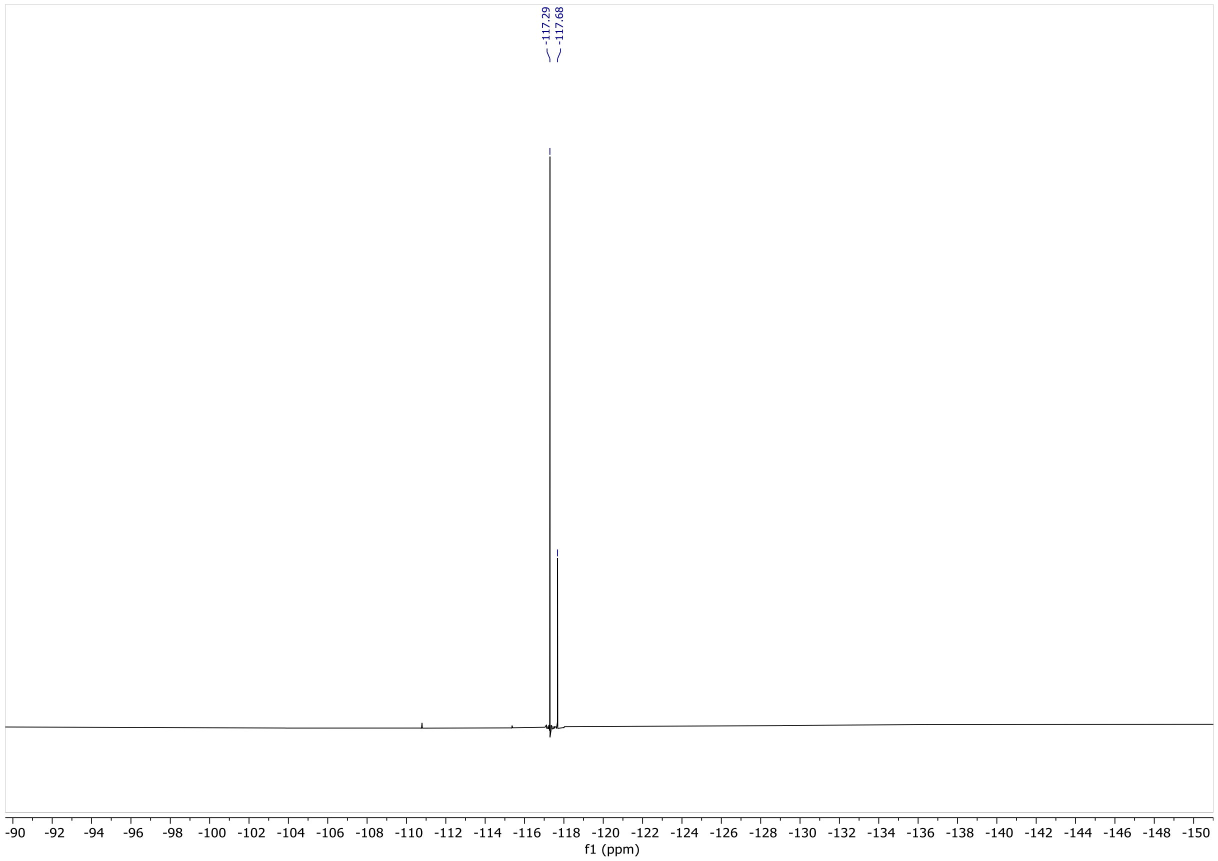

-117.29
-117.68
-90 -92 -94 -96 -98 -100 -102 -104 -106 -108 -110 -112 -114 -116 -118 -120 -122 -124 -126 -128 -130 -132 -134 -136 -138 -140 -142 -144 -146 -148 -150
f1 (ppm)

Methyl 3-(4-chlorophenyl)-3-(4-methoxyphenyl)cyclobutane-1-carboxylate (5c)

[^{1}H-NMR: 700 MHz, ^{13}C-NMR: 176 MHz]

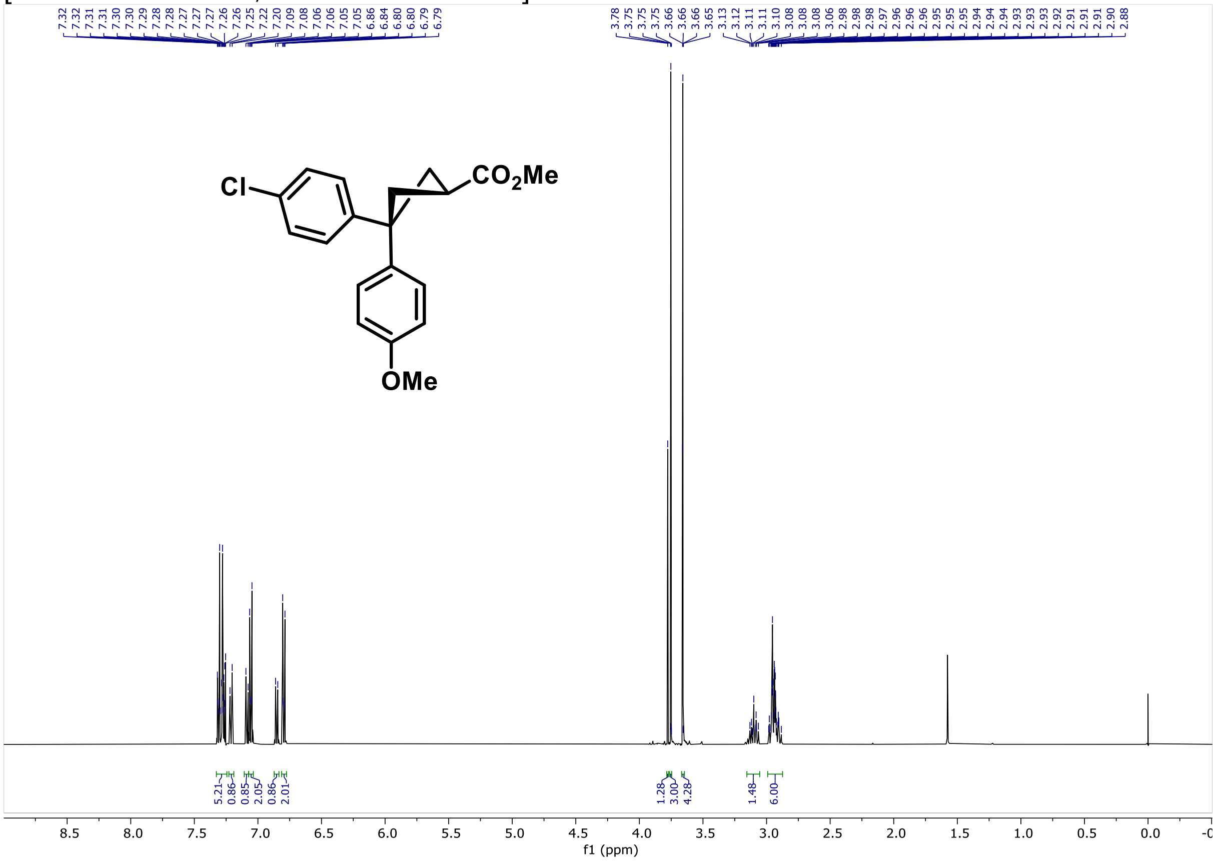

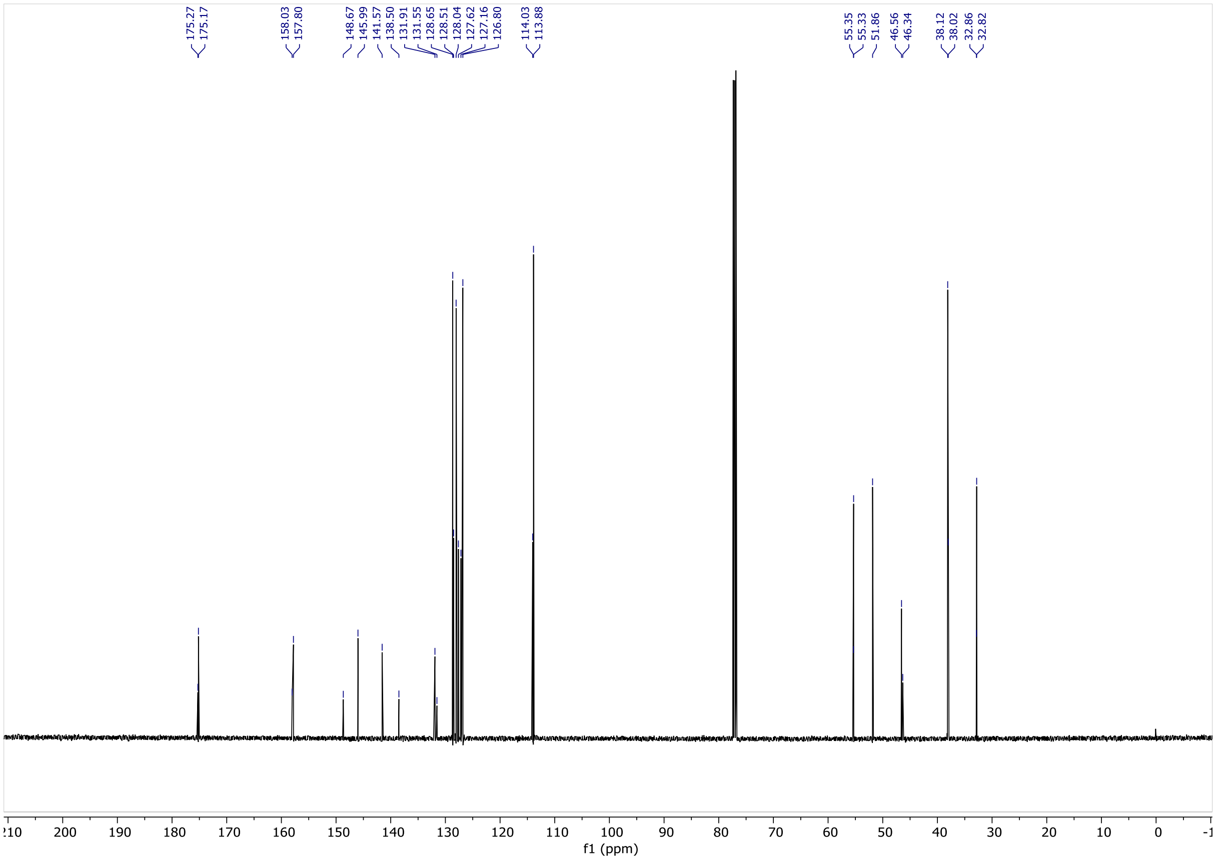

Methyl 3-(4-bromophenyl)-3-(4-methoxyphenyl)cyclobutane-1-carboxylate (5d)
[^{1}H-NMR: 500 MHz, ^{13}C-NMR: 176 MHz]

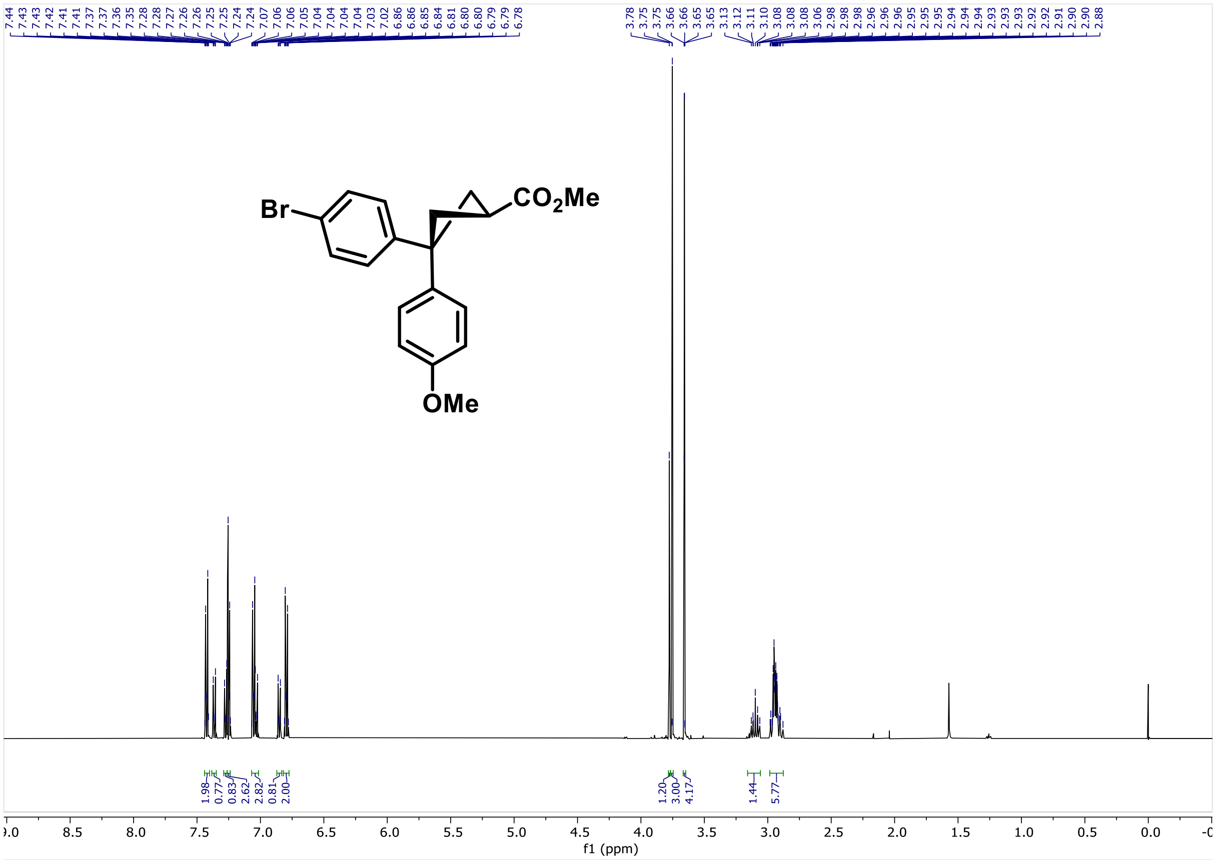

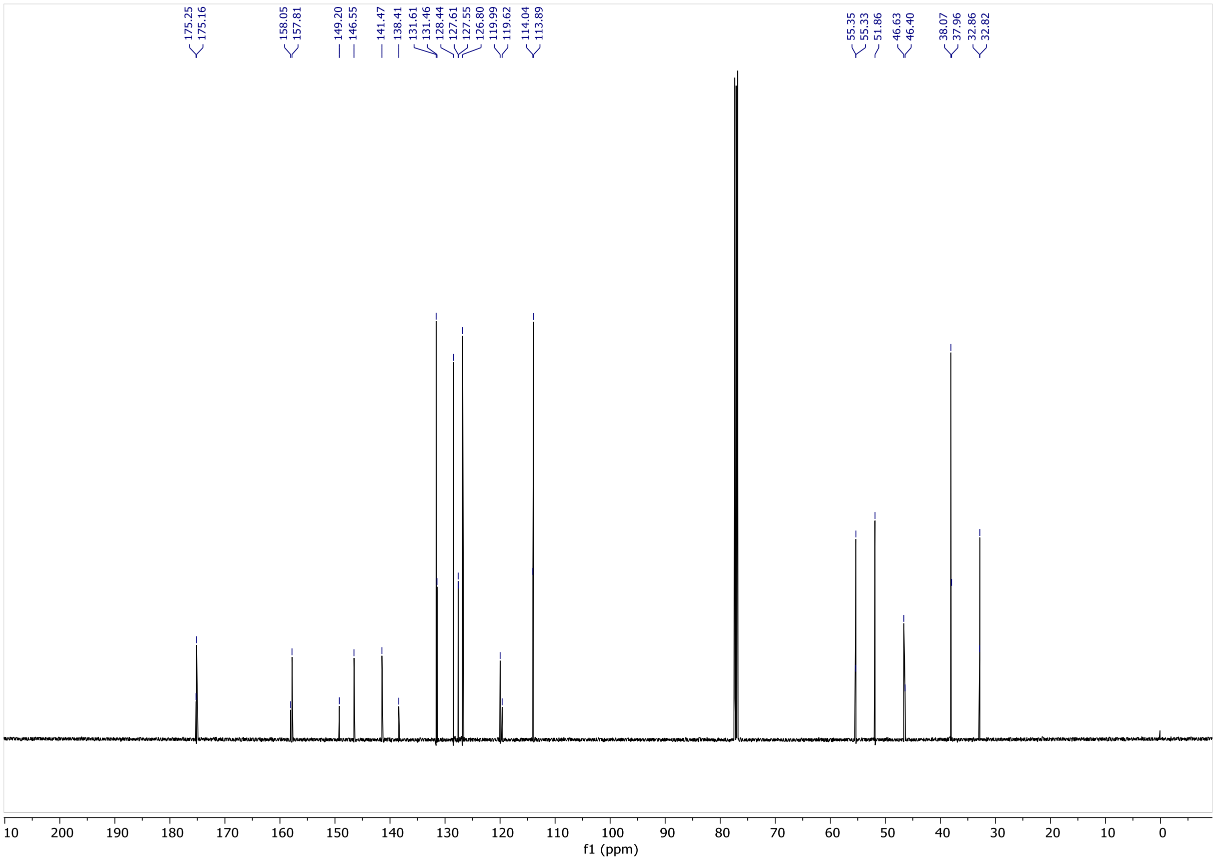

Methyl 3-(4-methoxyphenyl)-3-(4-(trifluoromethyl)phenyl)cyclobutane-1-carboxylate (5e)

[^{1}H-NMR: 500 MHz, ^{13}C-NMR: 126 MHz, ^{19}F-NMR: 471 MHz]

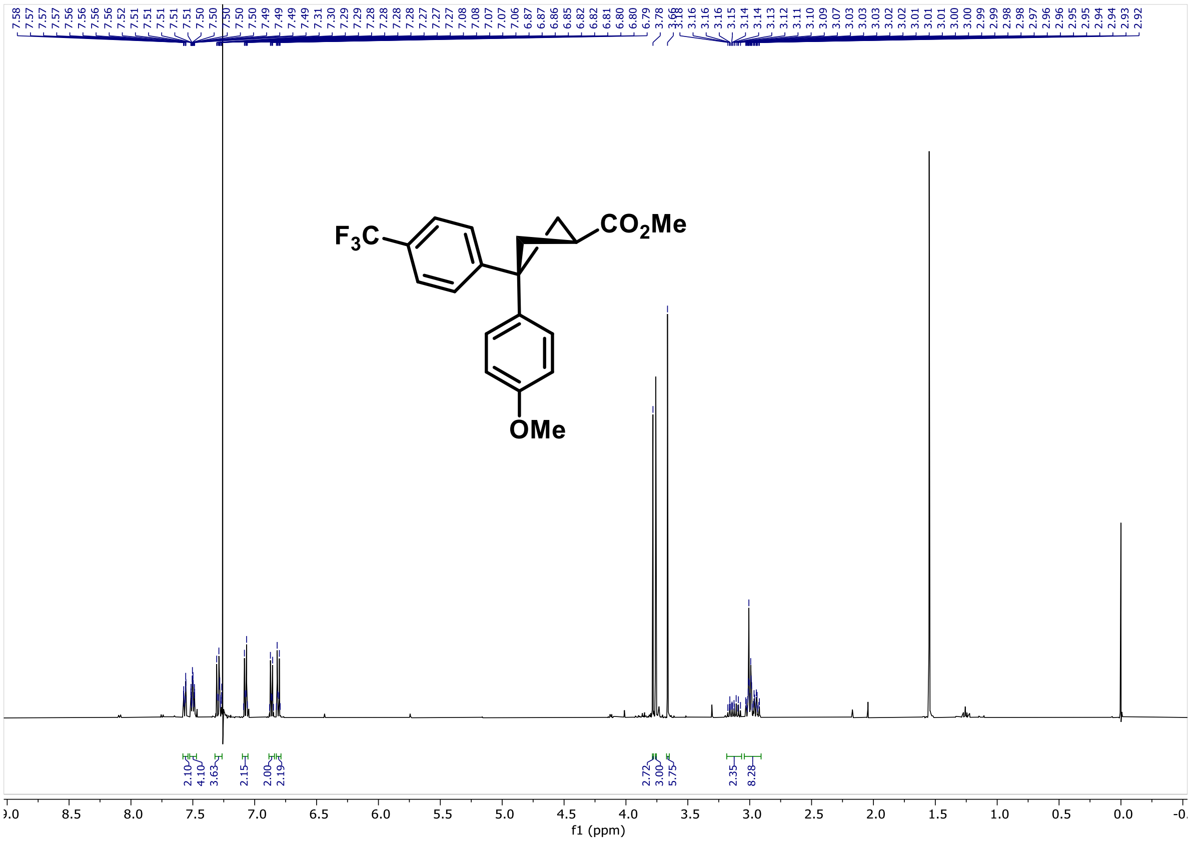

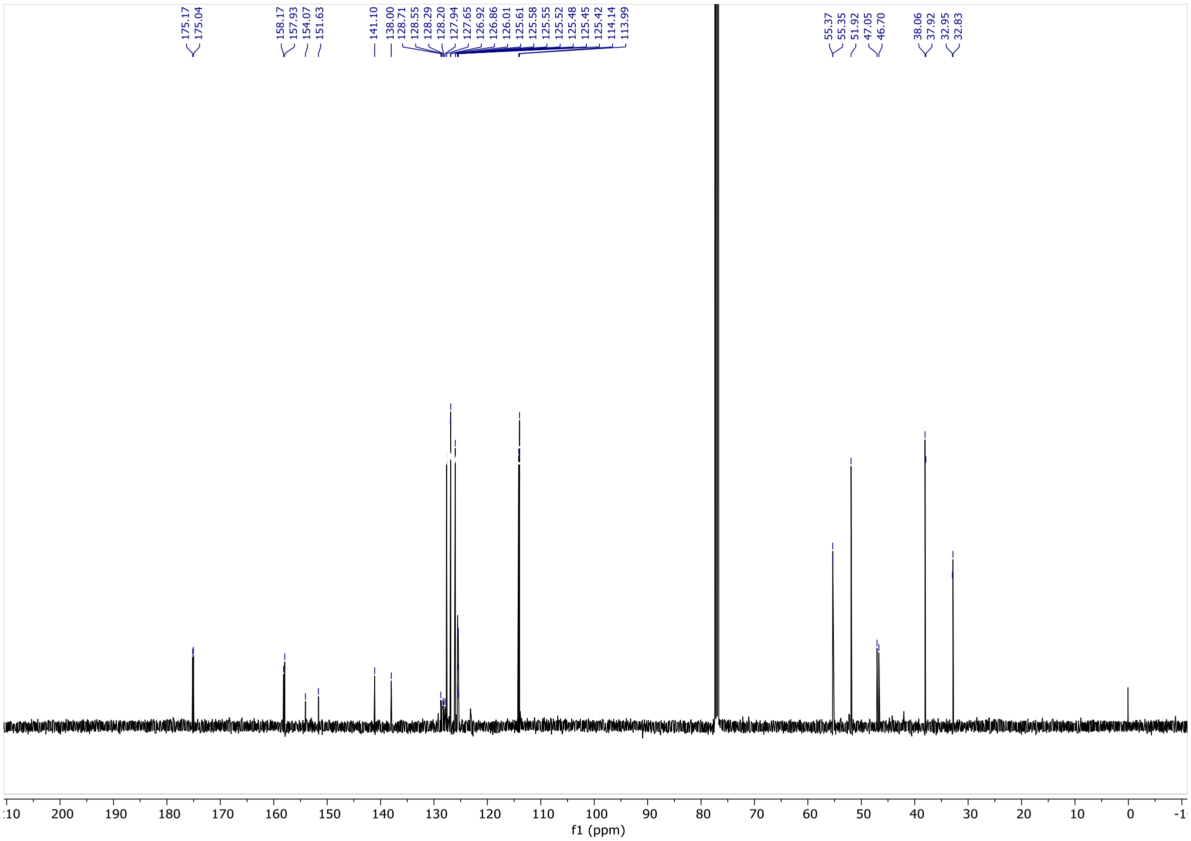

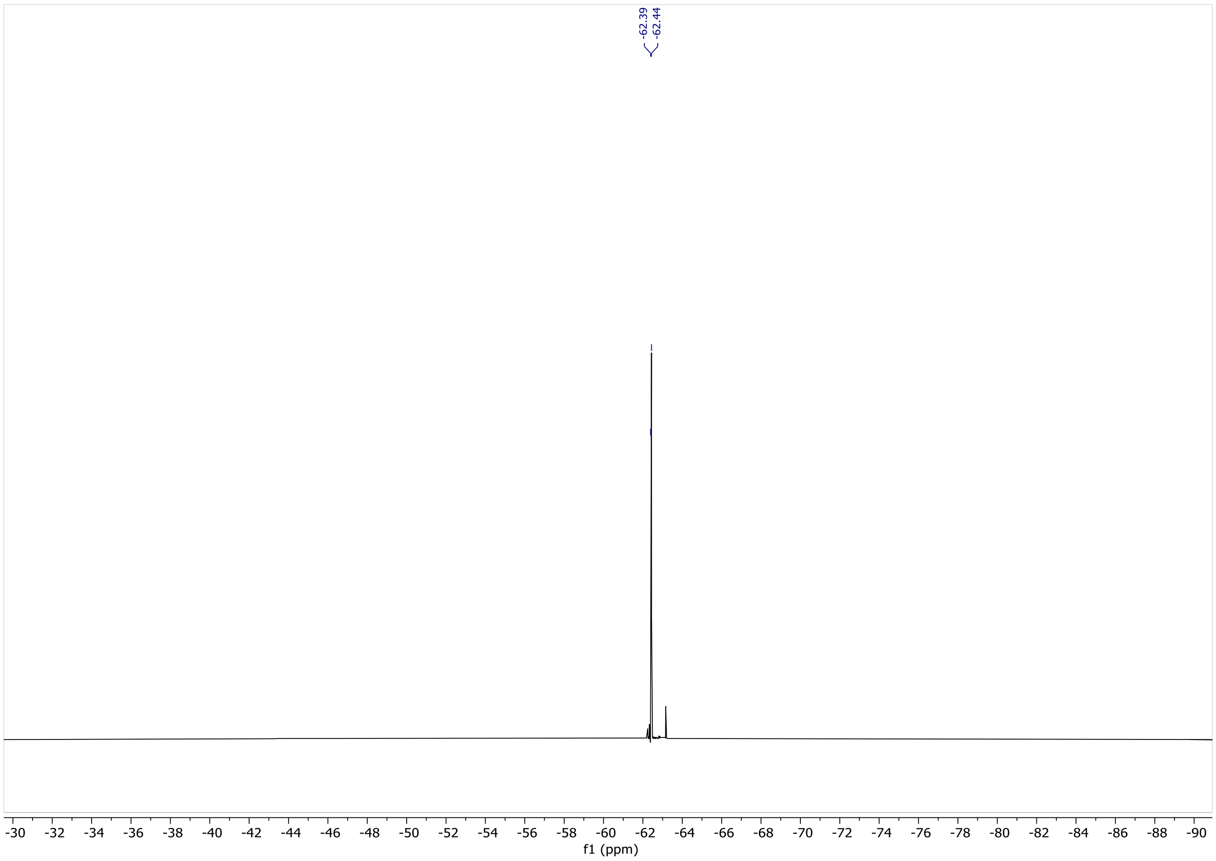
-62.39
-62.44
-30 -32 -34 -36 -38 -40 -42 -44 -46 -48 -50 -52 -54 -56 -58 -60 -62 -64 -66 -68 -70 -72 -74 -76 -78 -80 -82 -84 -86 -88 -90
f1 (ppm)

Methyl 3-(4-methoxyphenyl)-3-(4-(trifluoromethoxy)phenyl)cyclobutane-1-carboxylate (5f)

[^{1}H-NMR: 700 MHz, ^{13}C-NMR: 176 MHz, ^{19}F-NMR: 659 MHz]

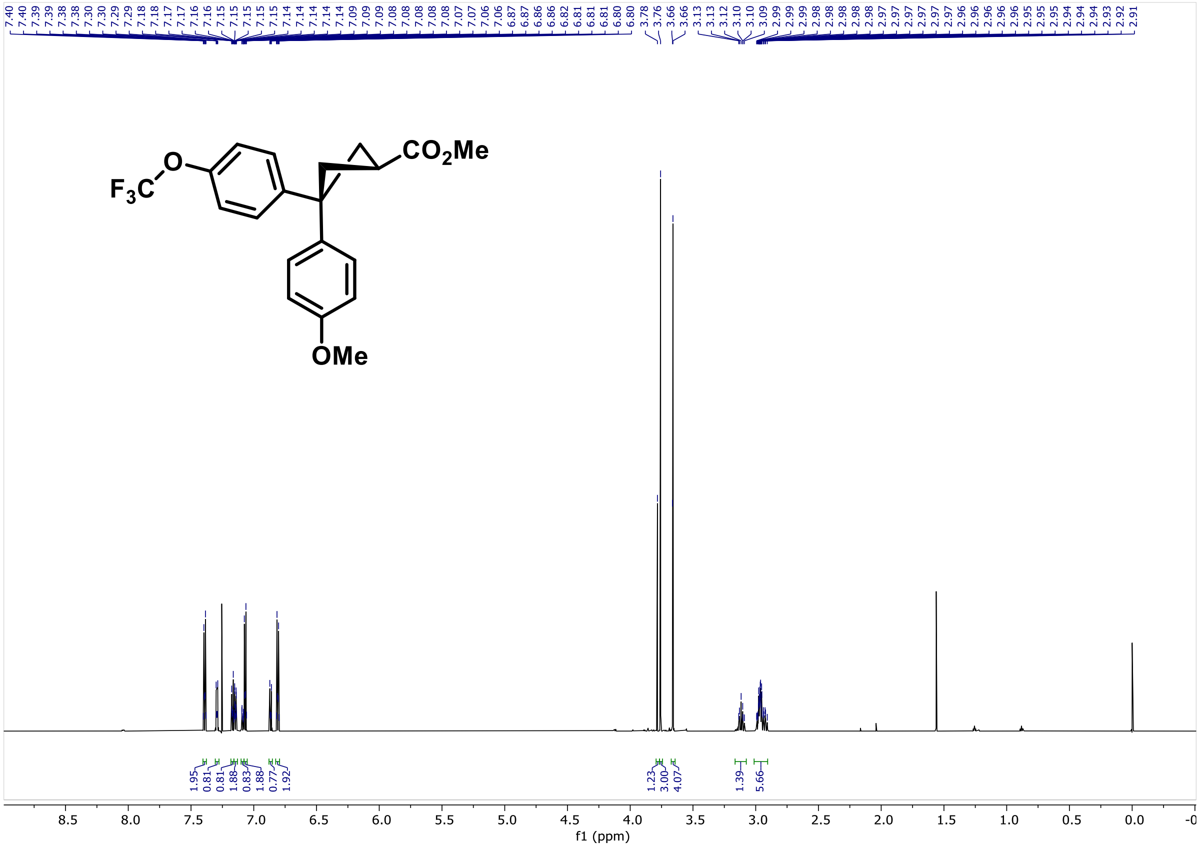

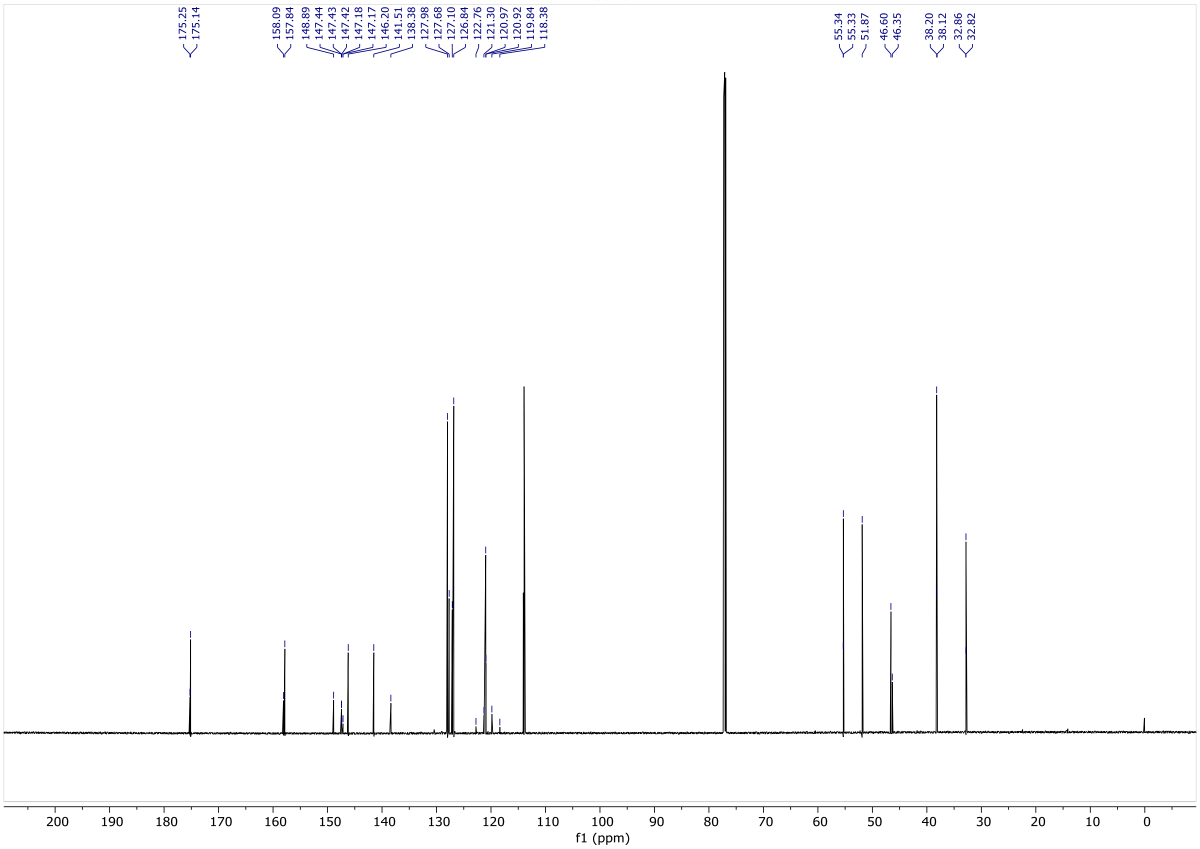

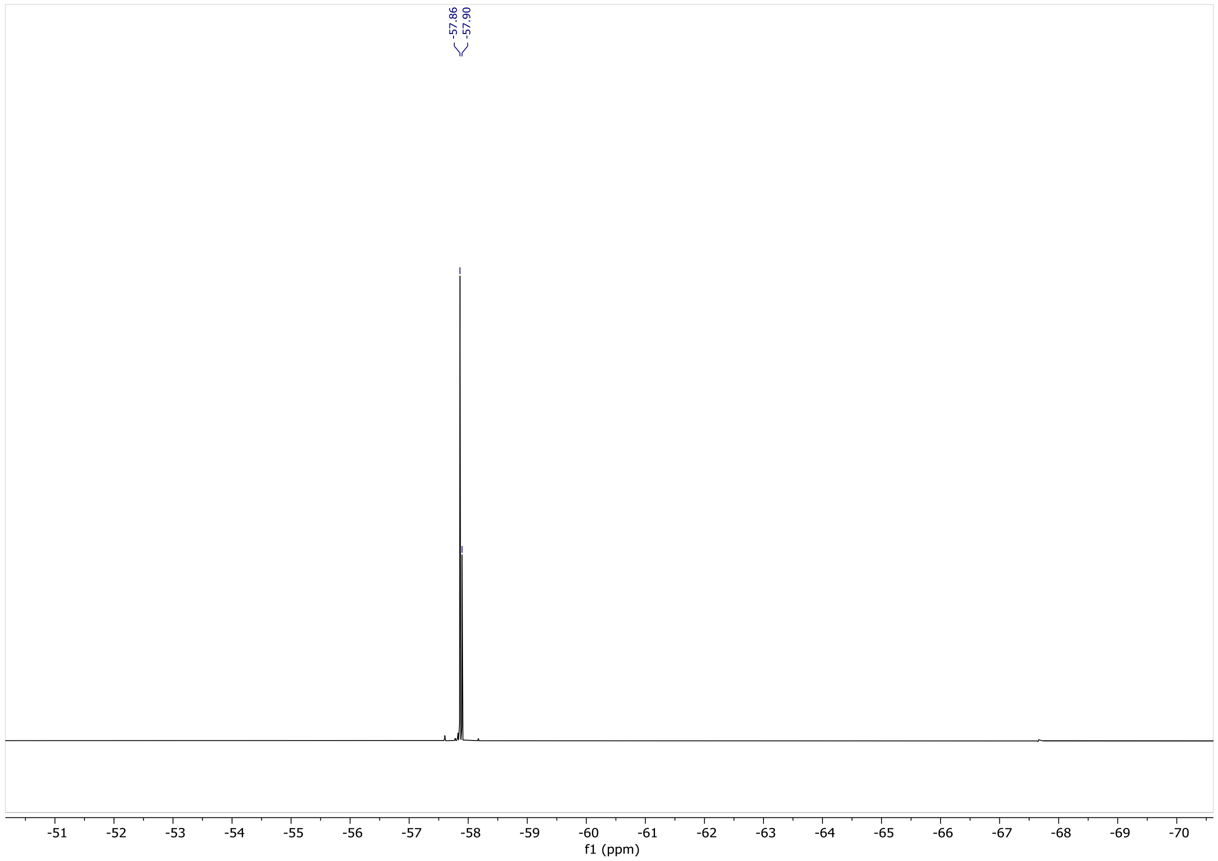
-57.86
-57.90
-51 -52 -53 -54 -55 -56 -57 -58 -59 -60 -61 -62 -63 -64 -65 -66 -67 -68 -69 -70
f1 (ppm)

Methyl 3-(3-methoxyphenyl)-3-(4-methoxyphenyl)cyclobutane-1-carboxylate (5g)
[^{1}H-NMR: 500 MHz, ^{13}C-NMR: 126 MHz]

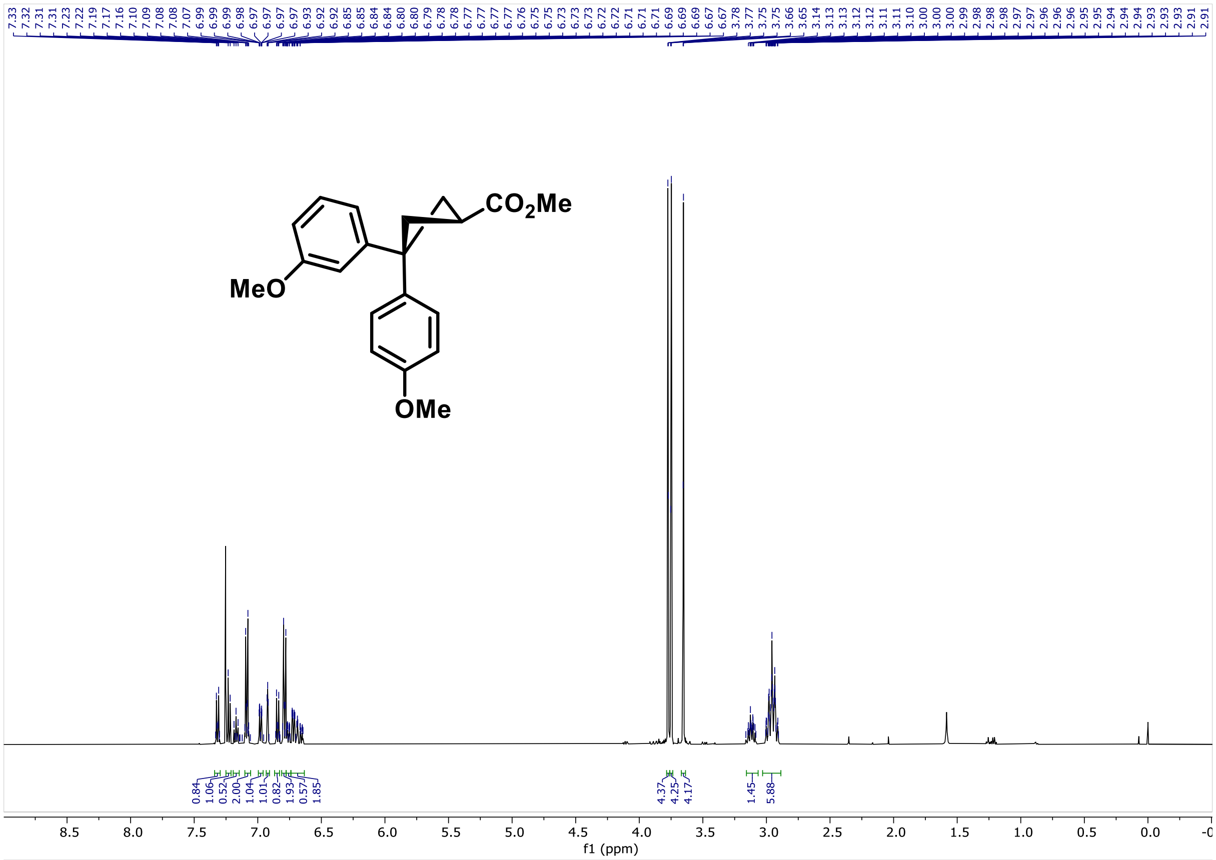

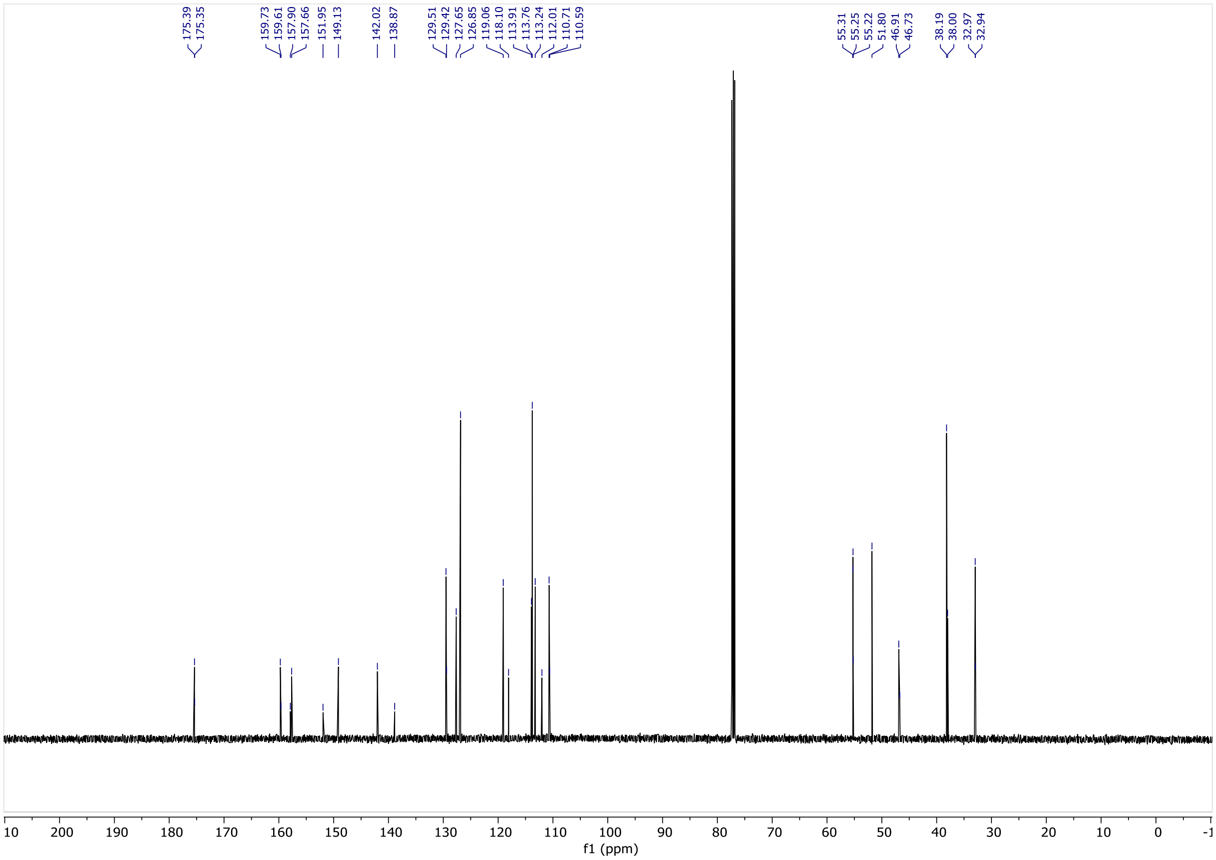

Methyl 3-(4-methoxyphenyl)-3-(m-tolyl)cyclobutane-1-carboxylate (5h)
[^{1}H-NMR: 500 MHz, ^{13}C-NMR: 126 MHz]

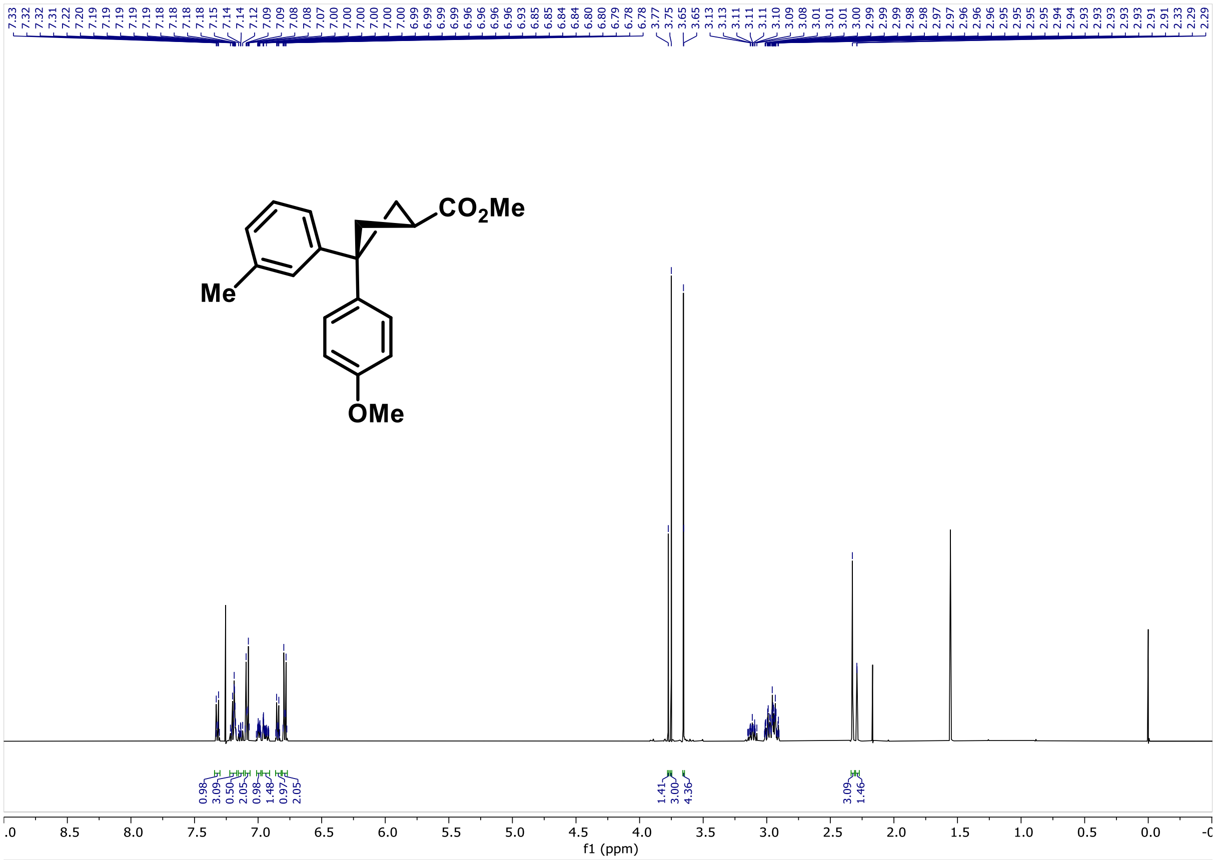

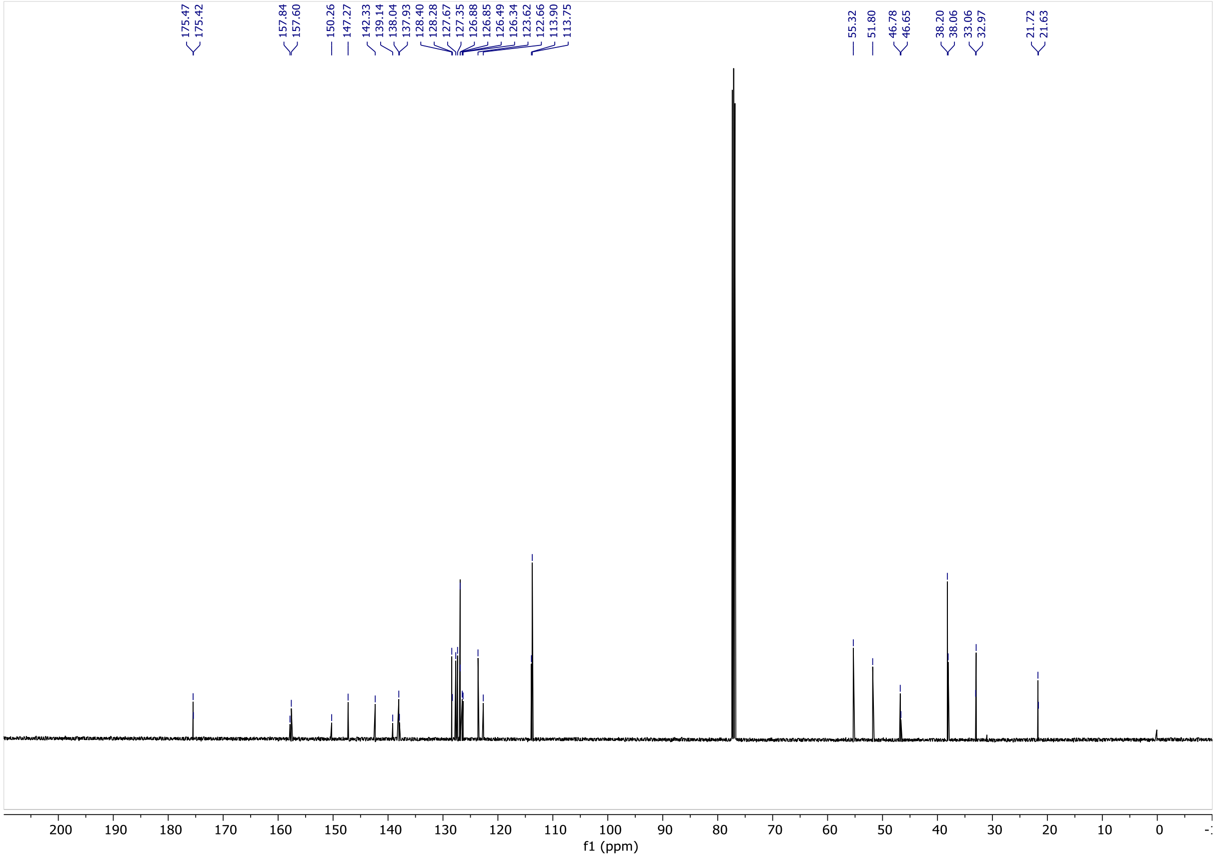

Methyl 3-(4-methoxyphenyl)-3-(naphthalen-2-yl)cyclobutane-1-carboxylate (5i)
[^{1}H-NMR: 500 MHz, ^{13}C-NMR: 126 MHz]

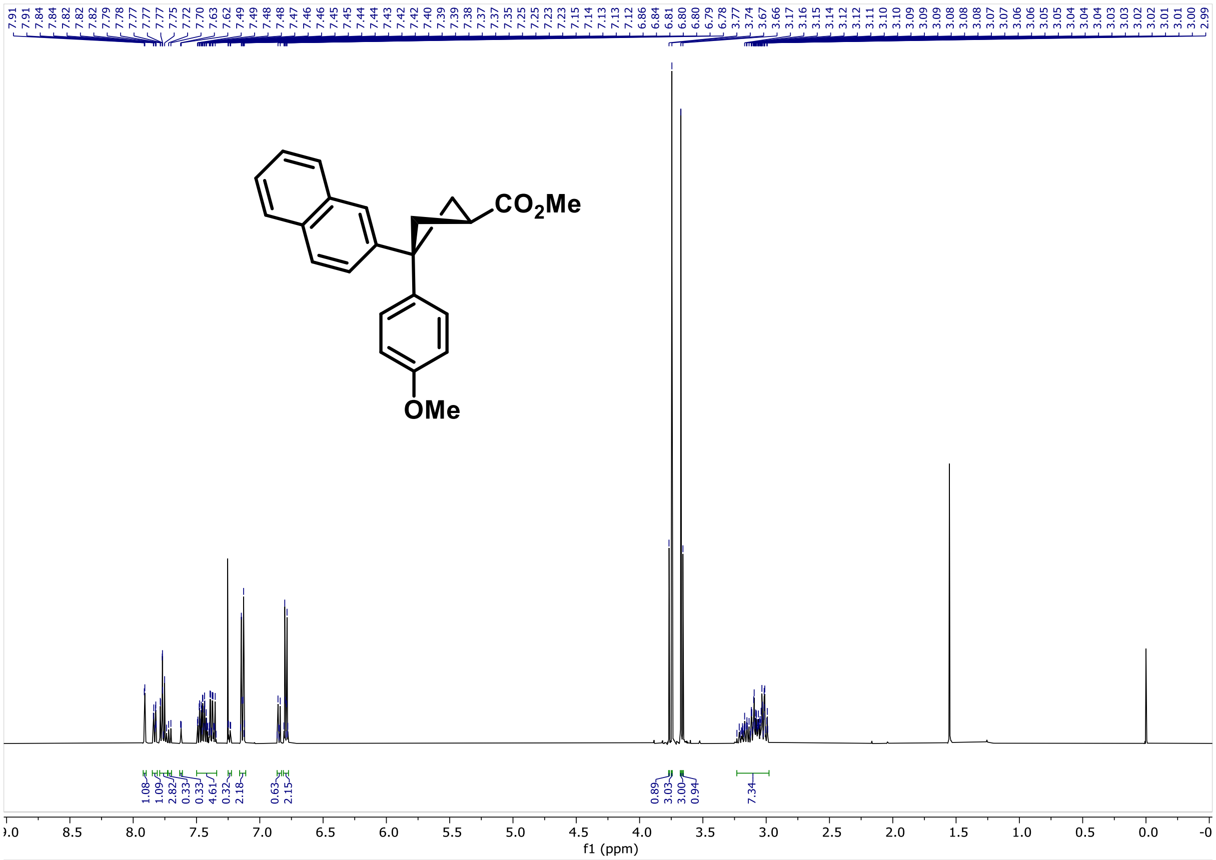

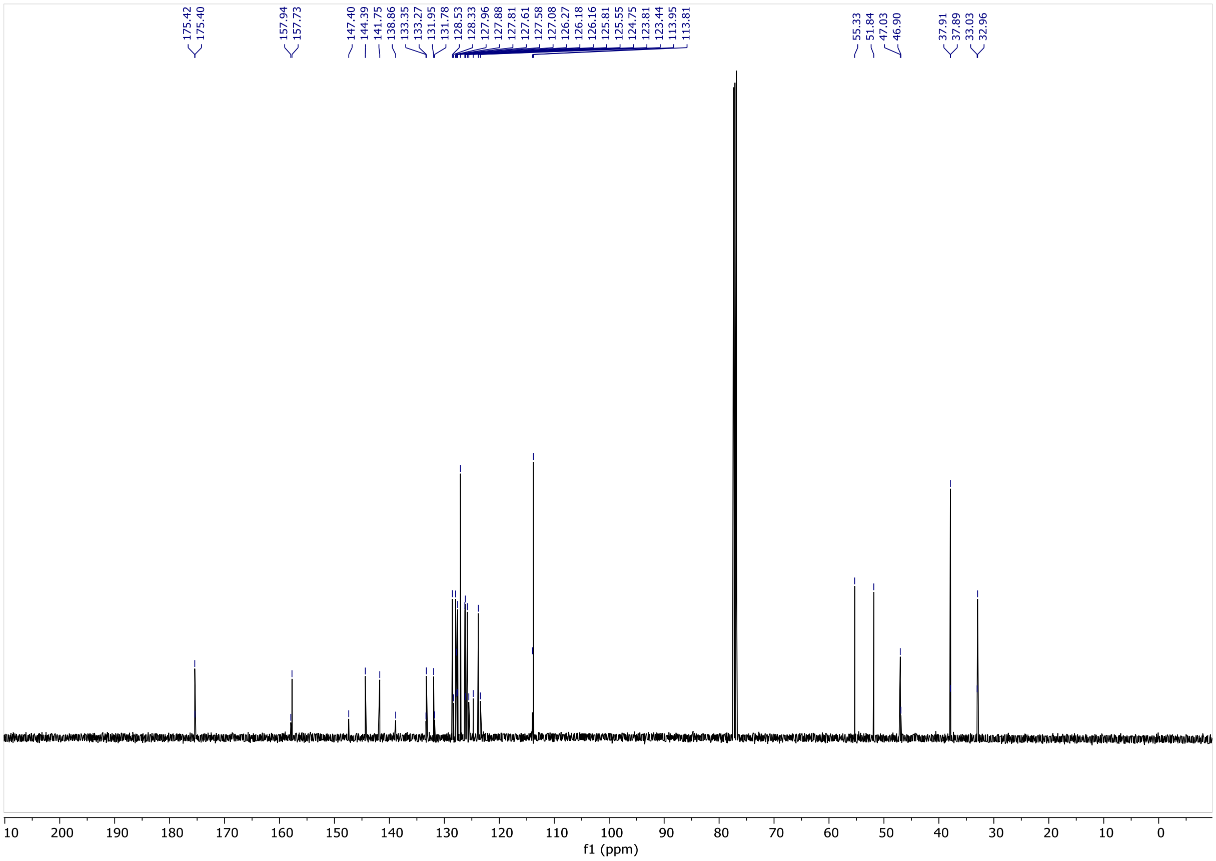

3-(4-Methoxyphenyl)-3-phenylcyclobutyl)(phenyl)methanone (5j)

[^{1}H-NMR: 700 MHz, ^{13}C-NMR: 176 MHz]

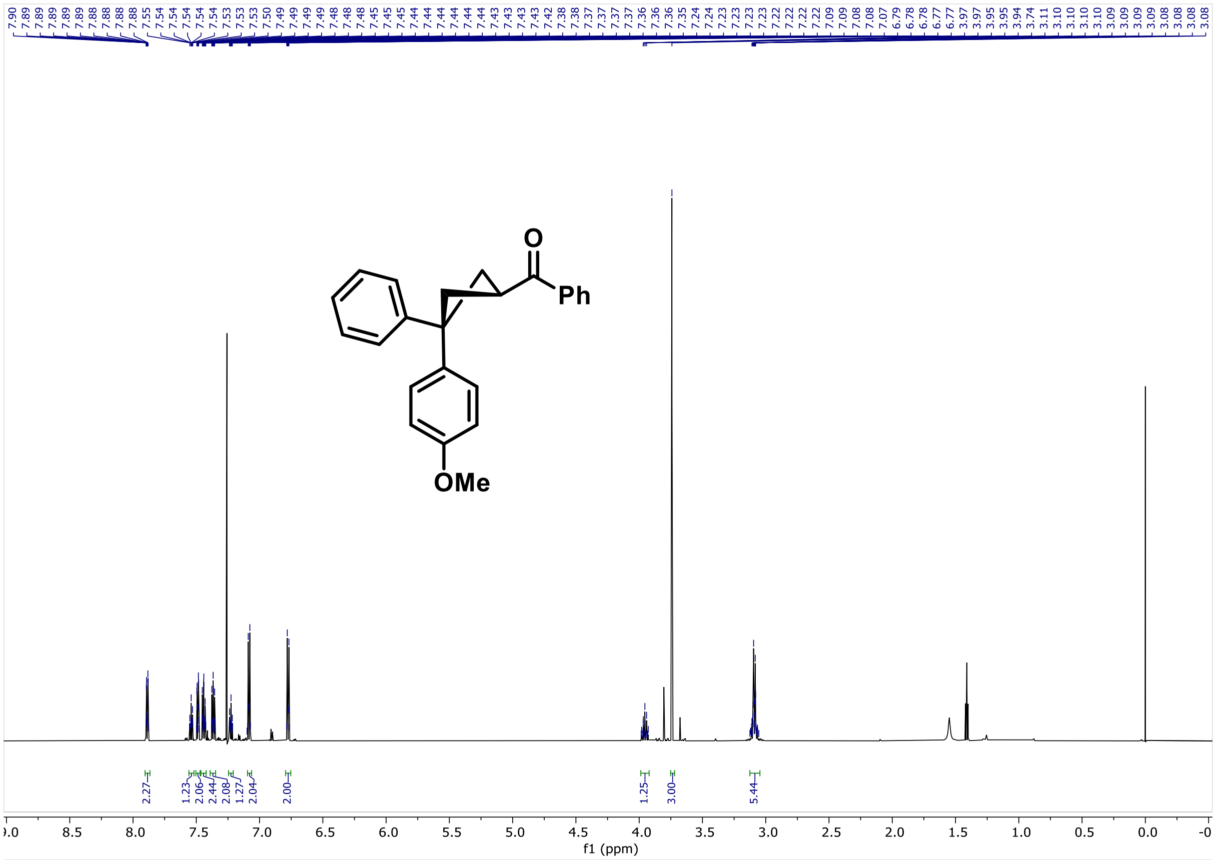

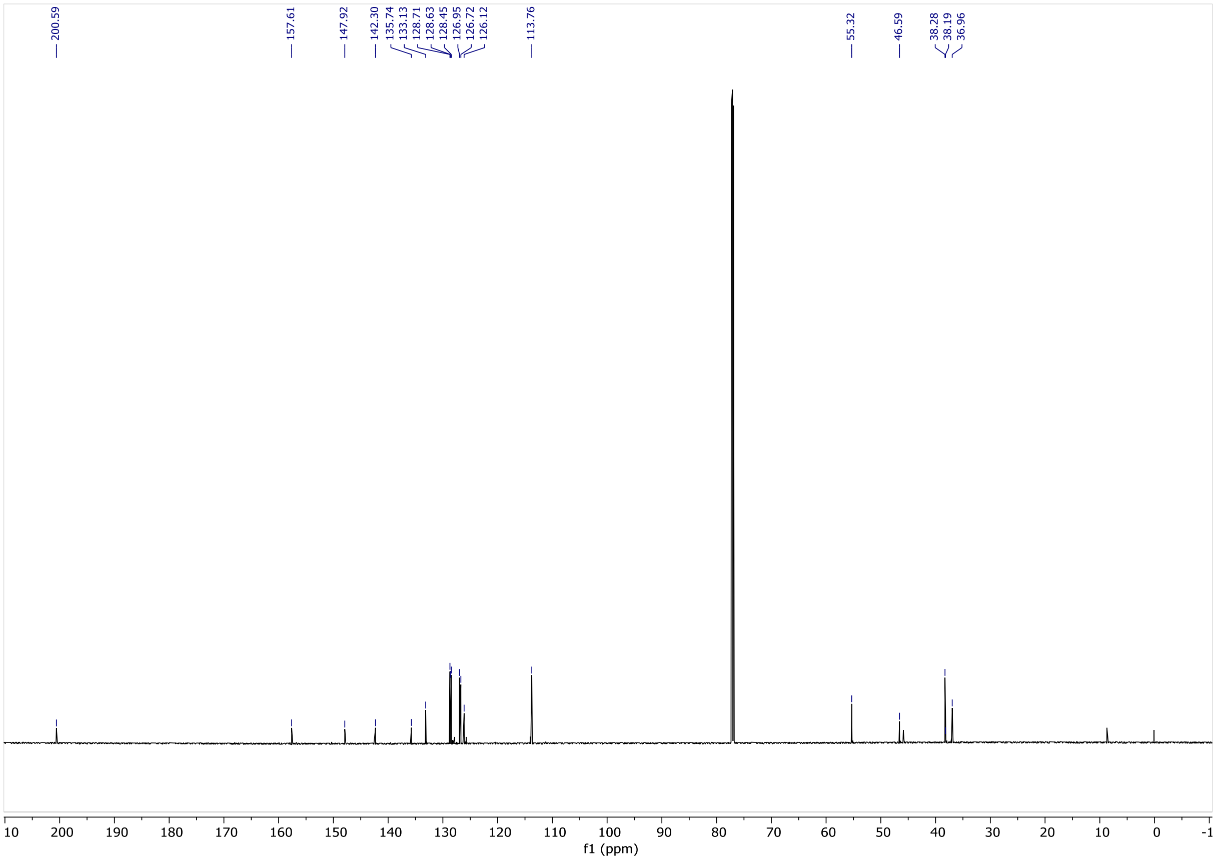

1-(3-(4-Methoxyphenyl)-3-phenylcyclobutyl)ethan-1-one (5k)

[^{1}H-NMR: 700 MHz, ^{13}C-NMR: 176 MHz]

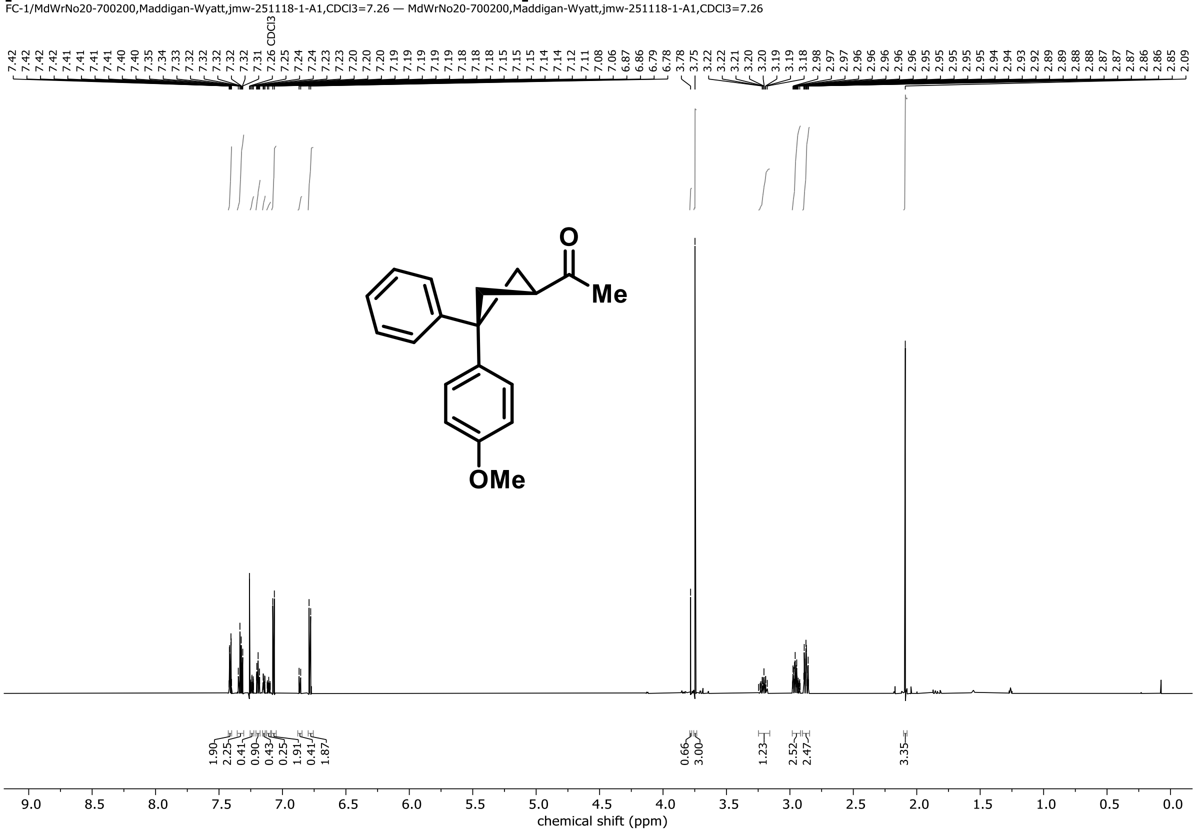

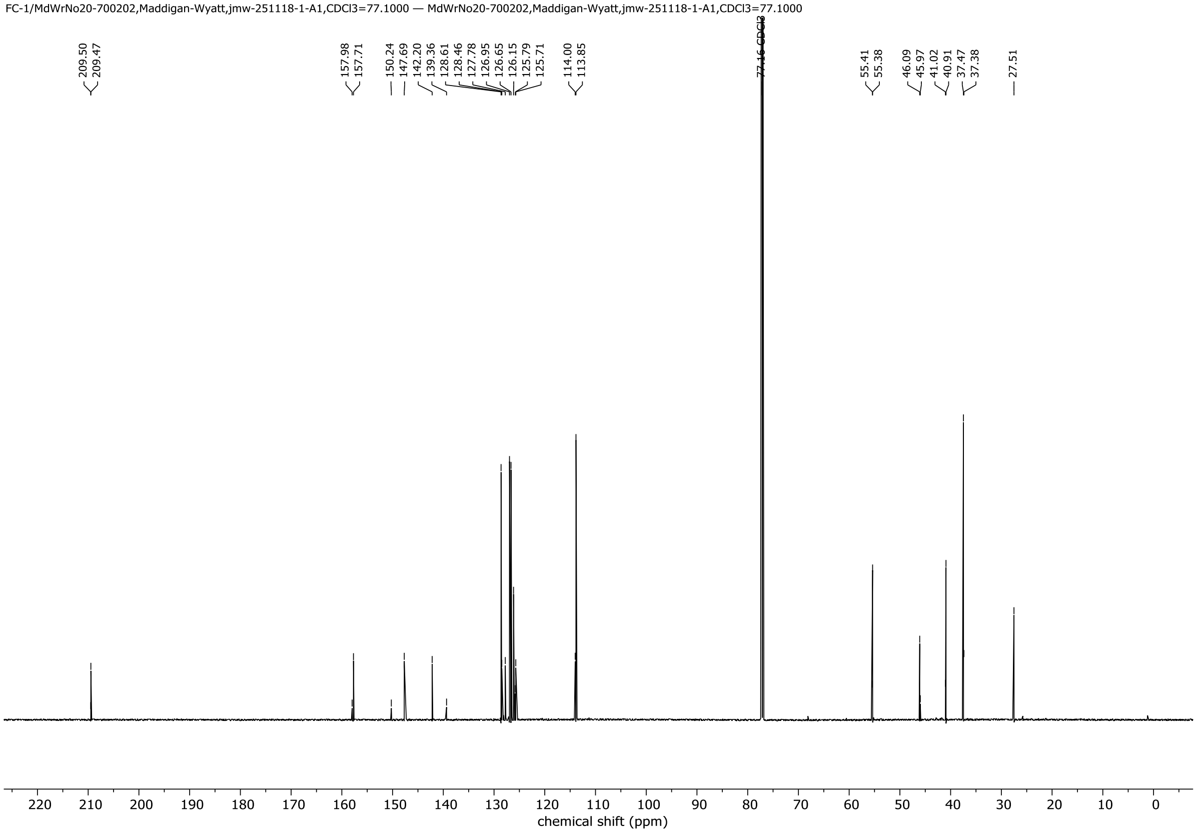

1-(3-(4-Methoxyphenyl)-3-phenylcyclobutyl)pentan-1-one (5l)

[^{1}H-NMR: 700 MHz, ^{13}C-NMR: 176 MHz]

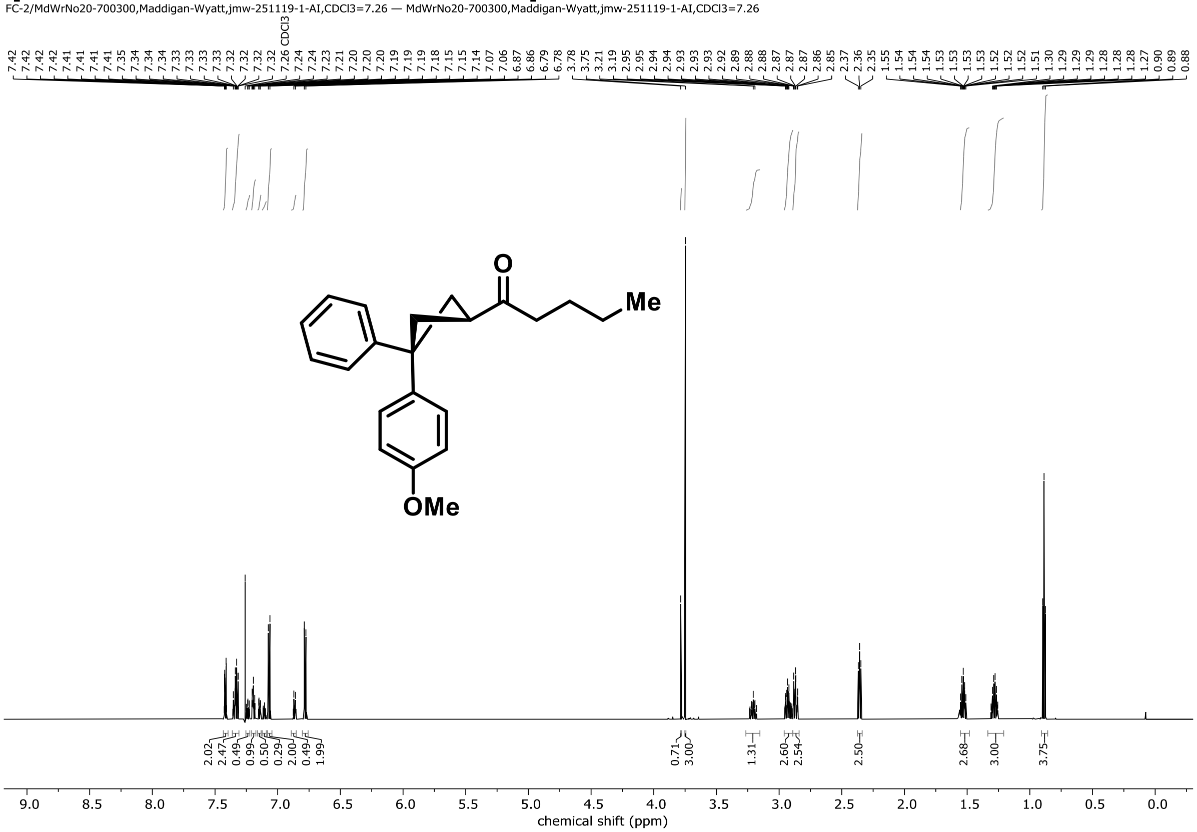

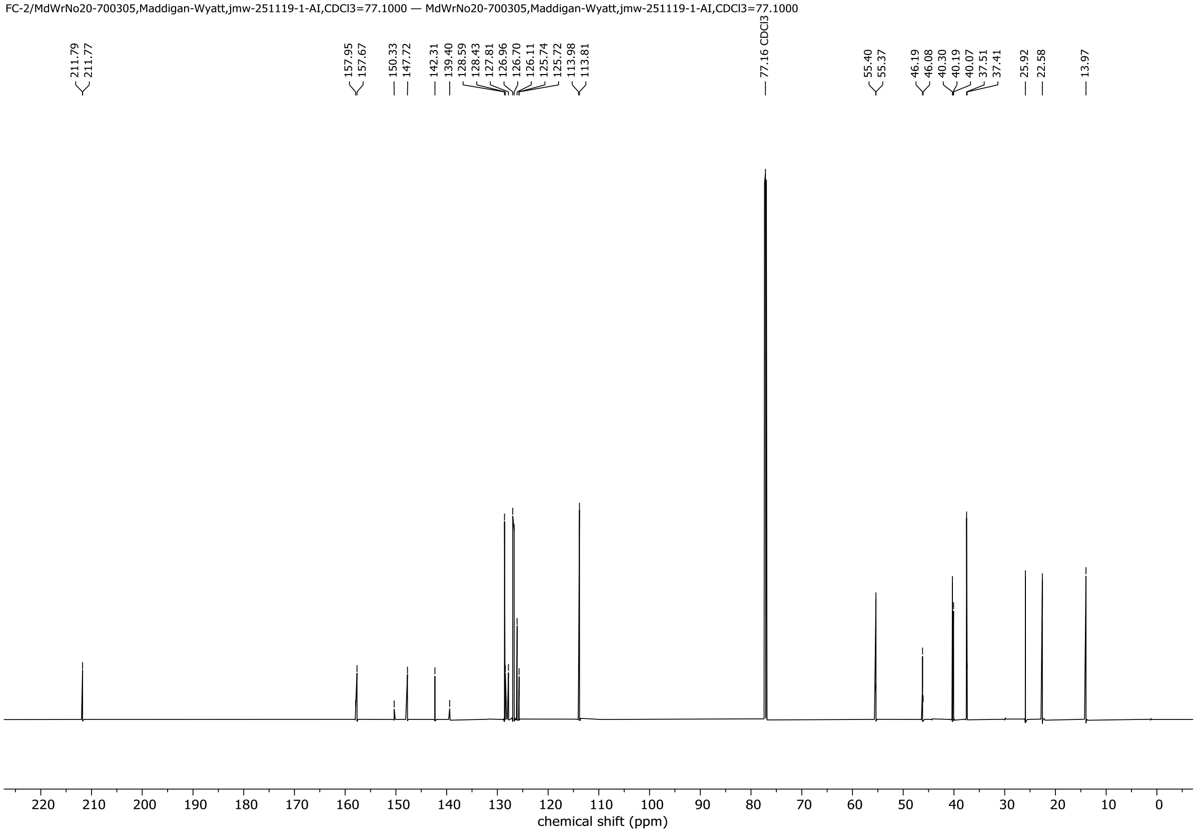

Methyl 3-phenyl-3-(p-tolyl)cyclobutane-1-carboxylate (5m)
[^{1}H-NMR: 500 MHz, ^{13}C-NMR: 126 MHz]

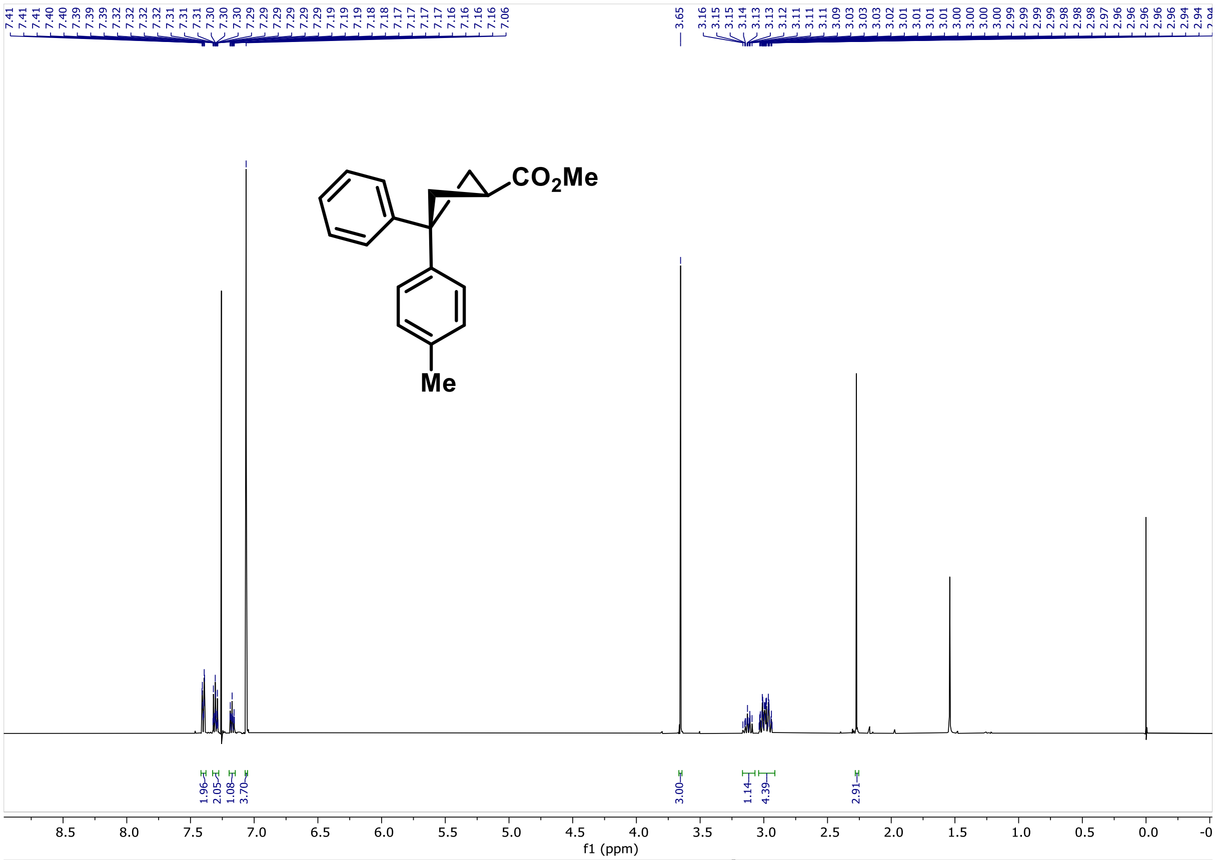

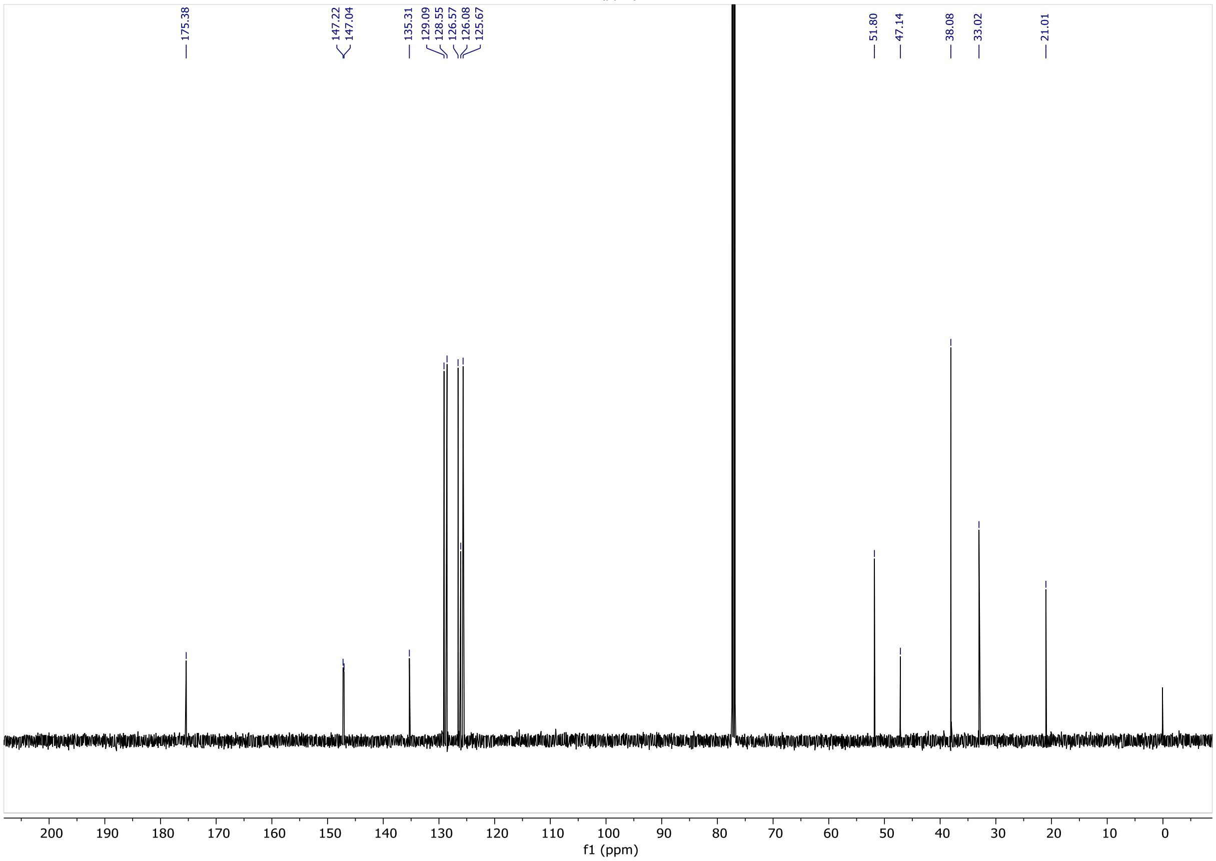

Methyl 3-(4-(dimethylamino)phenyl)-3-phenylcyclobutane-1-carboxylate (5n)
[^{1}H-NMR: 500 MHz, ^{13}C-NMR: 126 MHz]

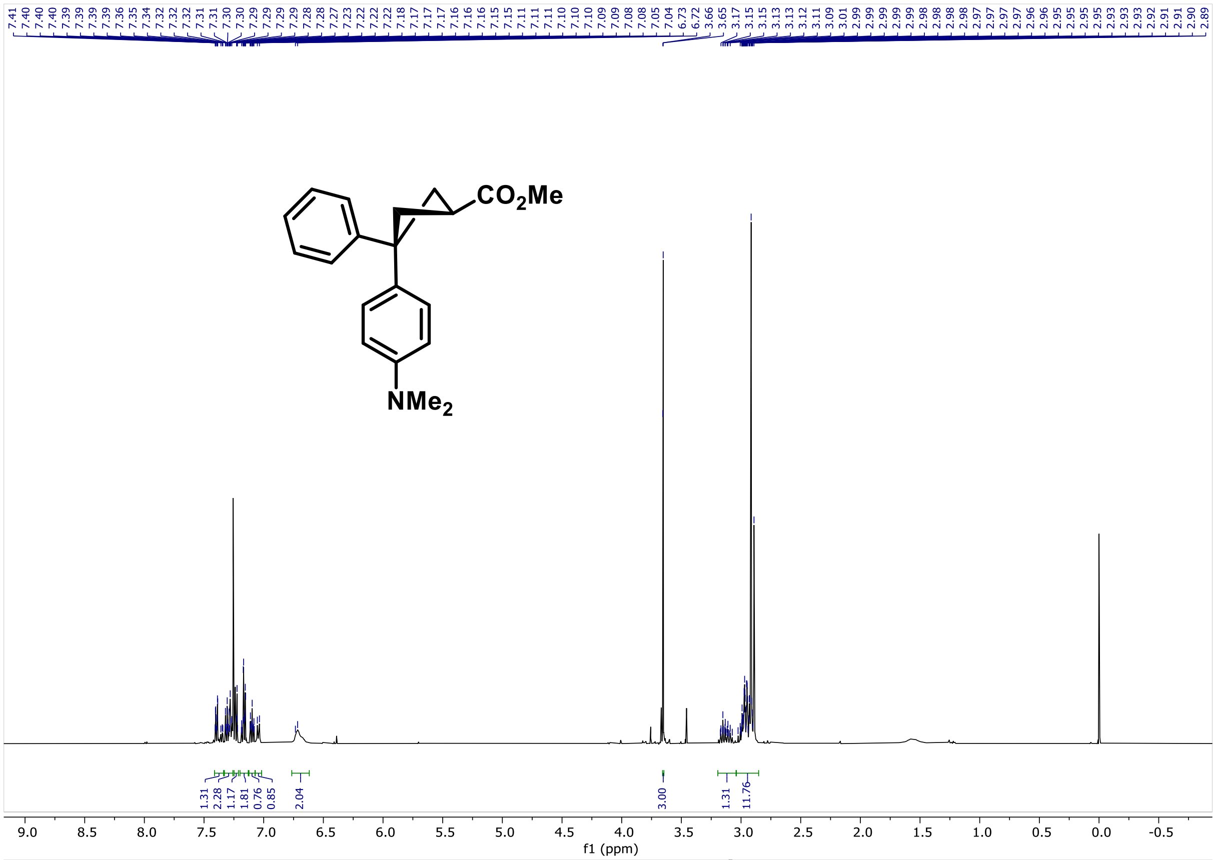

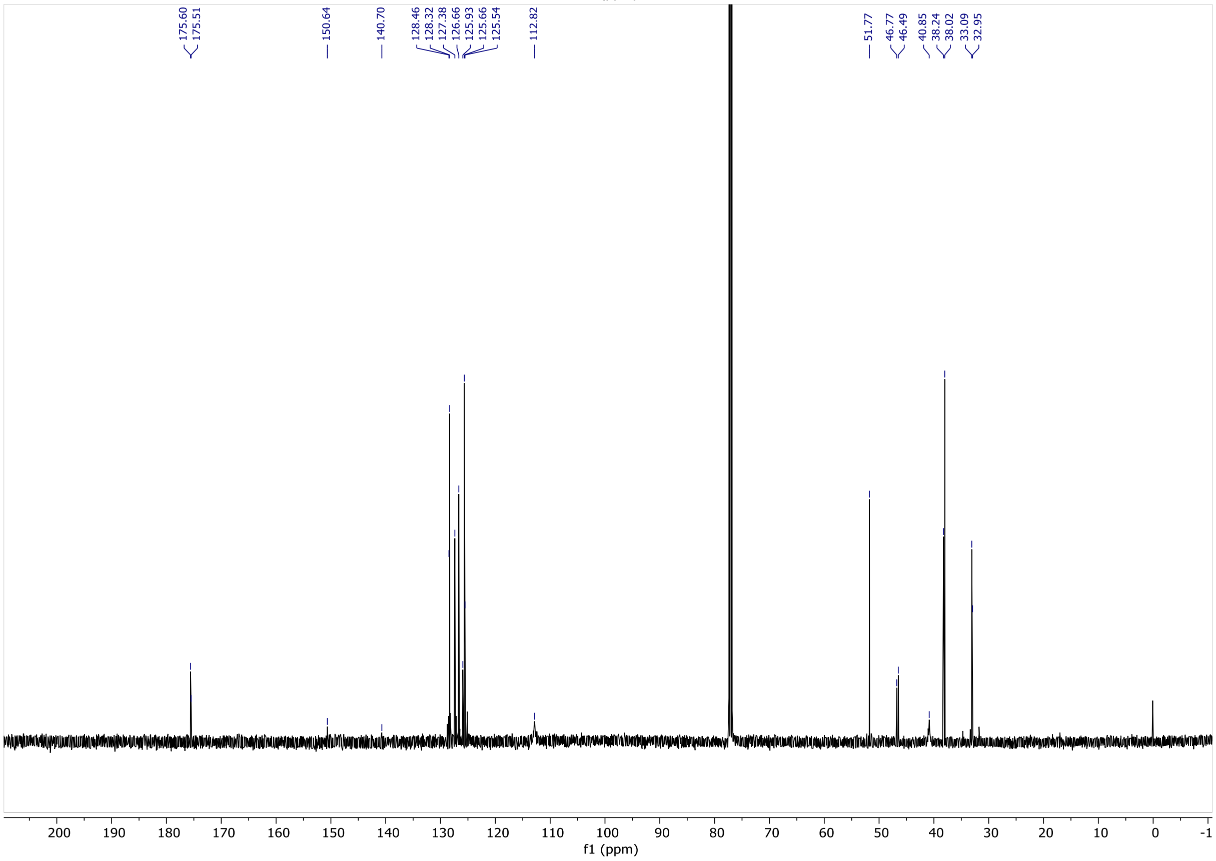

Methyl 3-(5-bromo-2-methoxyphenyl)-3-phenylcyclobutane-1-carboxylate (5p)
[^{1}H-NMR: 700 MHz, ^{13}C-NMR: 176 MHz]

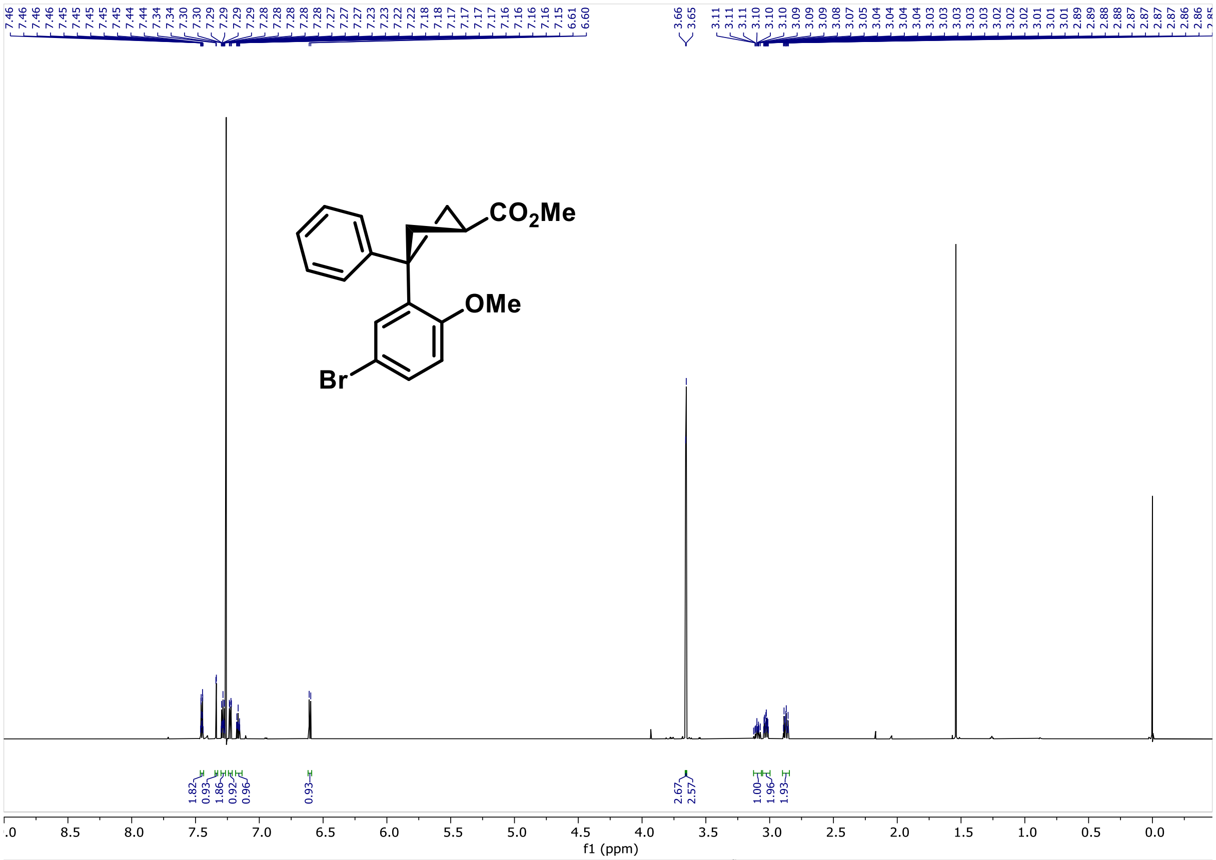

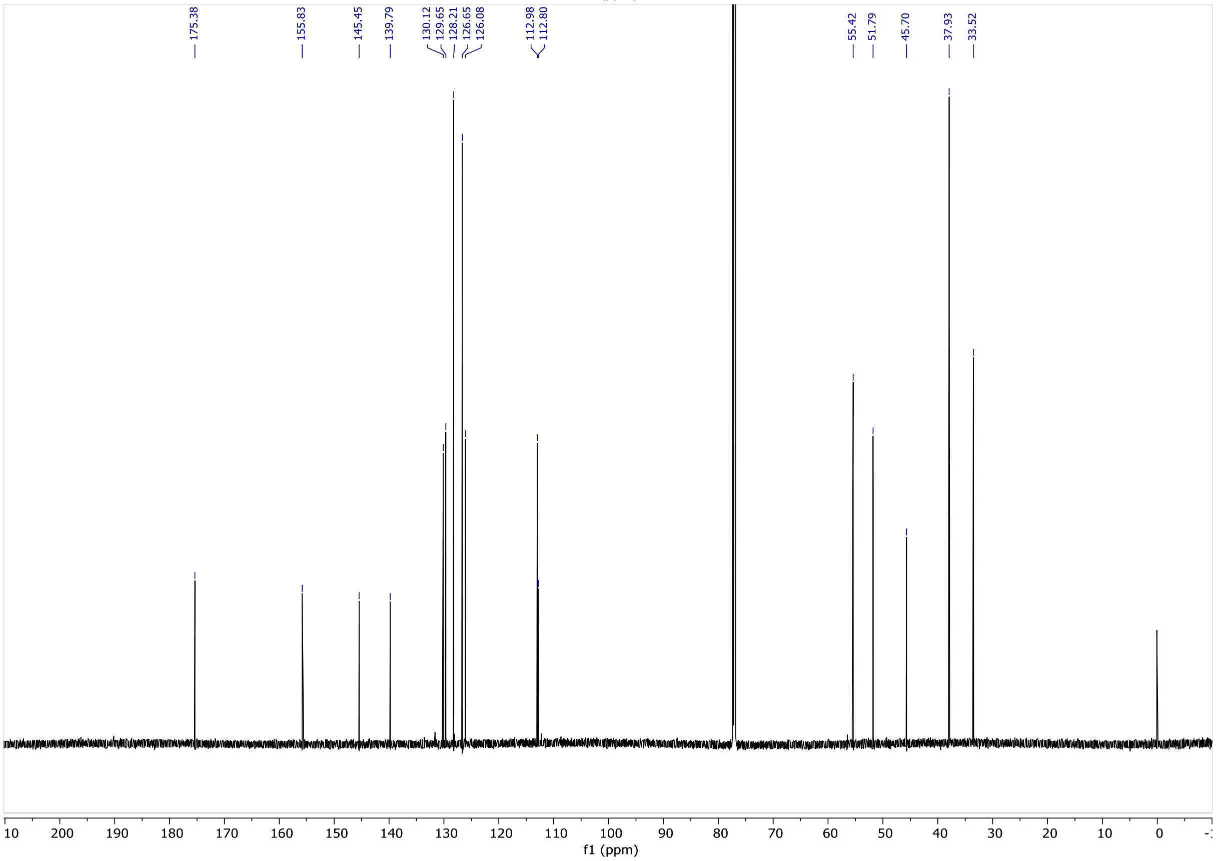

Methyl 3-(2,5-dimethoxyphenyl)-3-phenylcyclobutane-1-carboxylate (5q)
[^{1}H-NMR: 500 MHz, ^{13}C-NMR: 126 MHz]

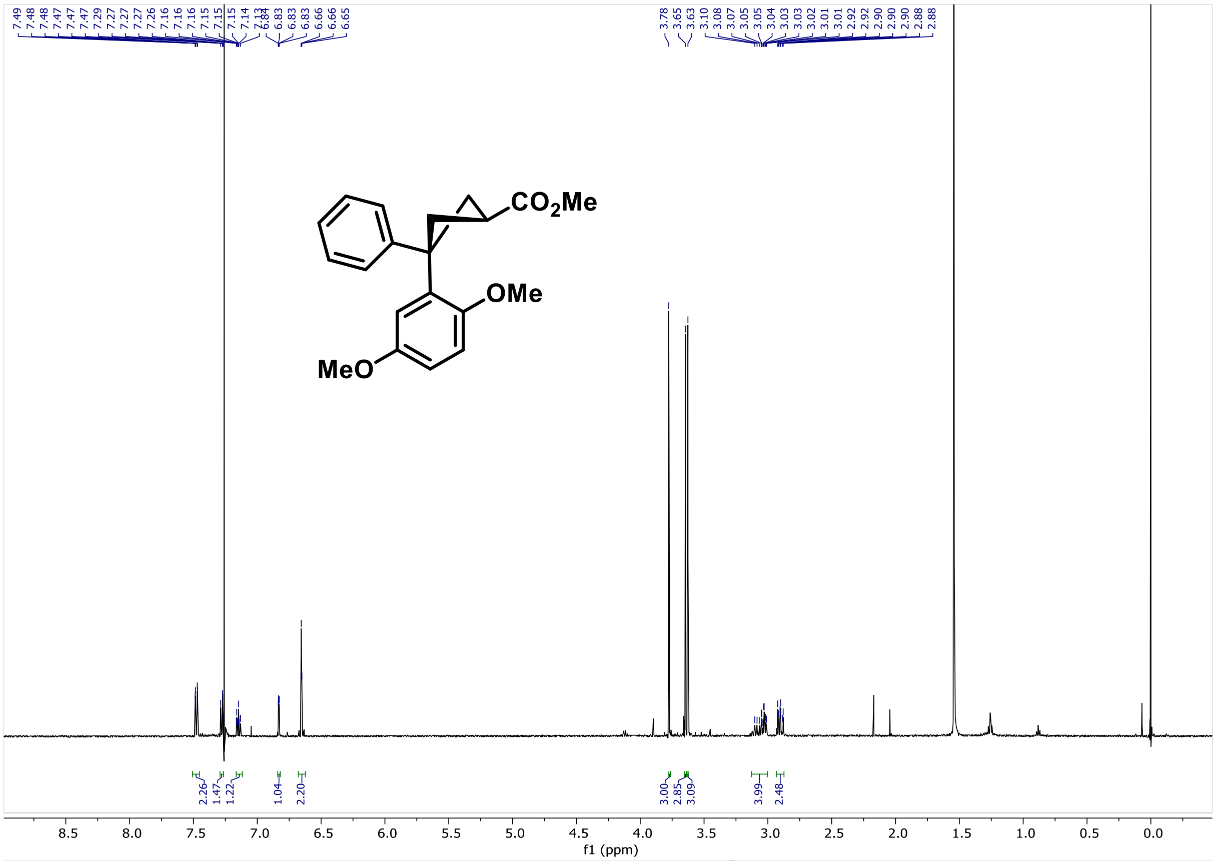

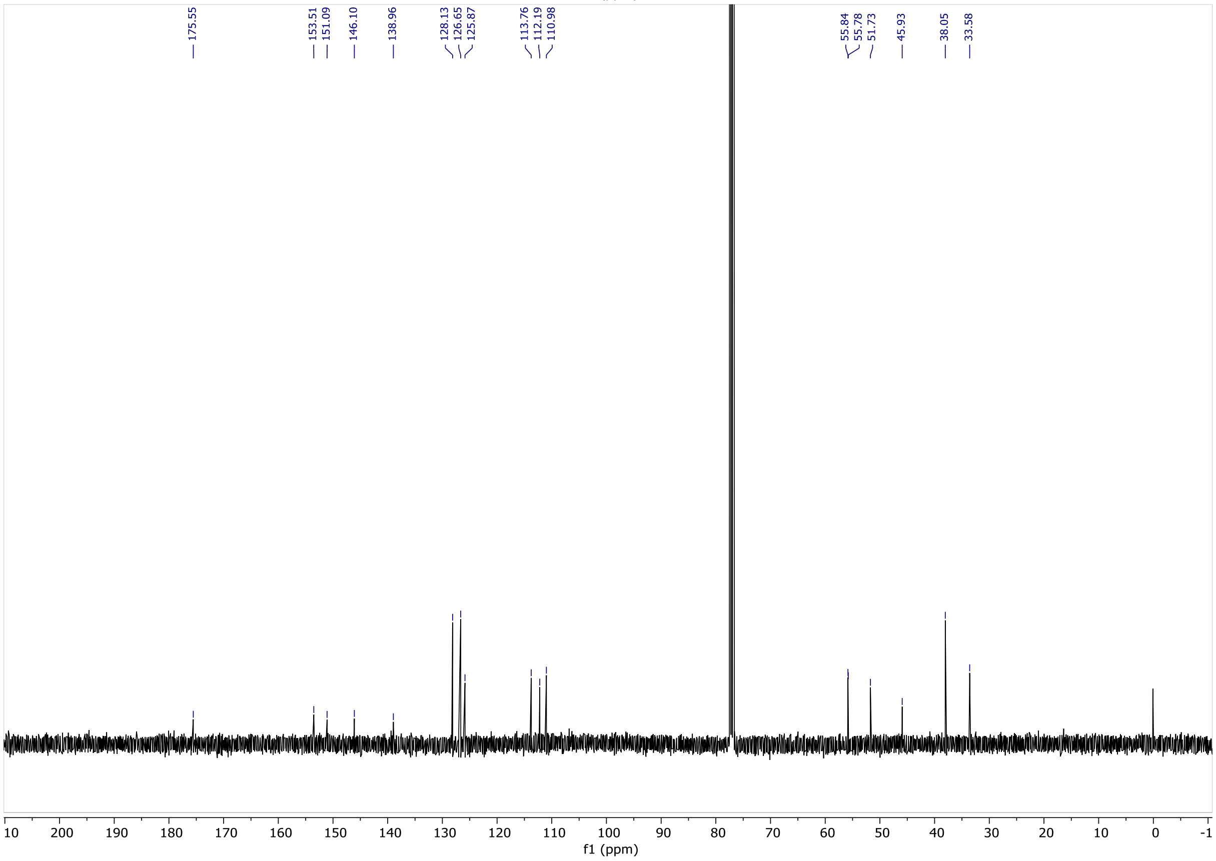

Methyl 3-(2,5-dimethylphenyl)-3-phenylcyclobutane-1-carboxylate (5r)

[^{1}H-NMR: 500 MHz, ^{13}C-NMR: 126 MHz]

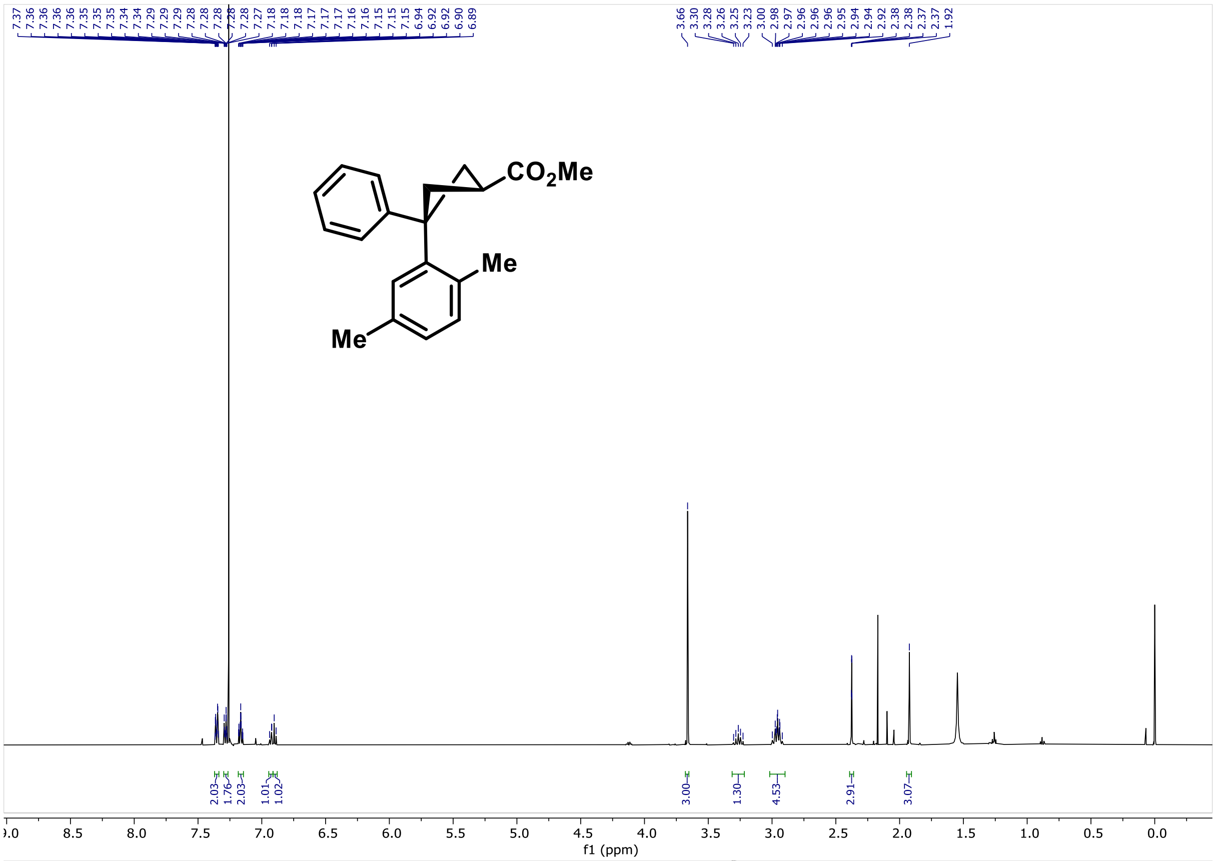

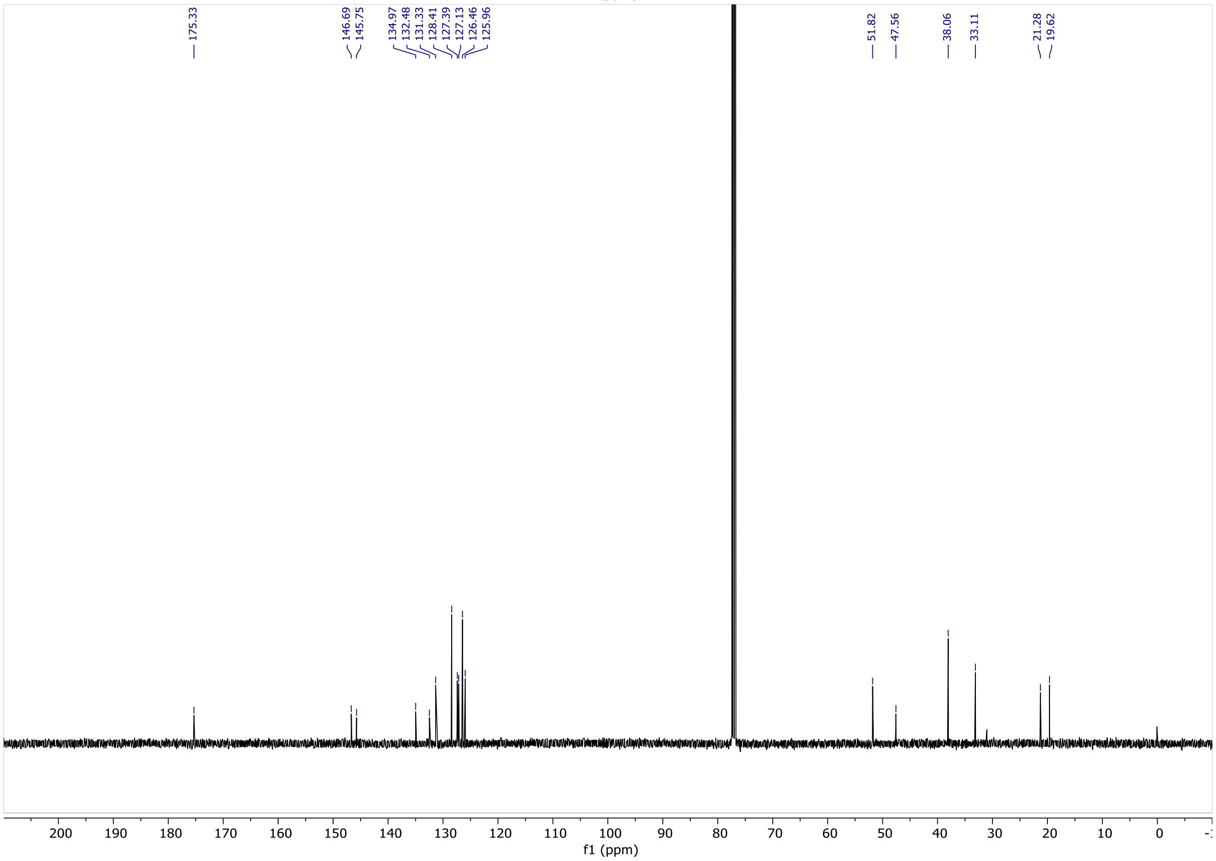

Methyl 3-mesityl-3-phenylcyclobutane-1-carboxylate (5s)
[^{1}H-NMR: 700 MHz, ^{13}C-NMR: 176 MHz]

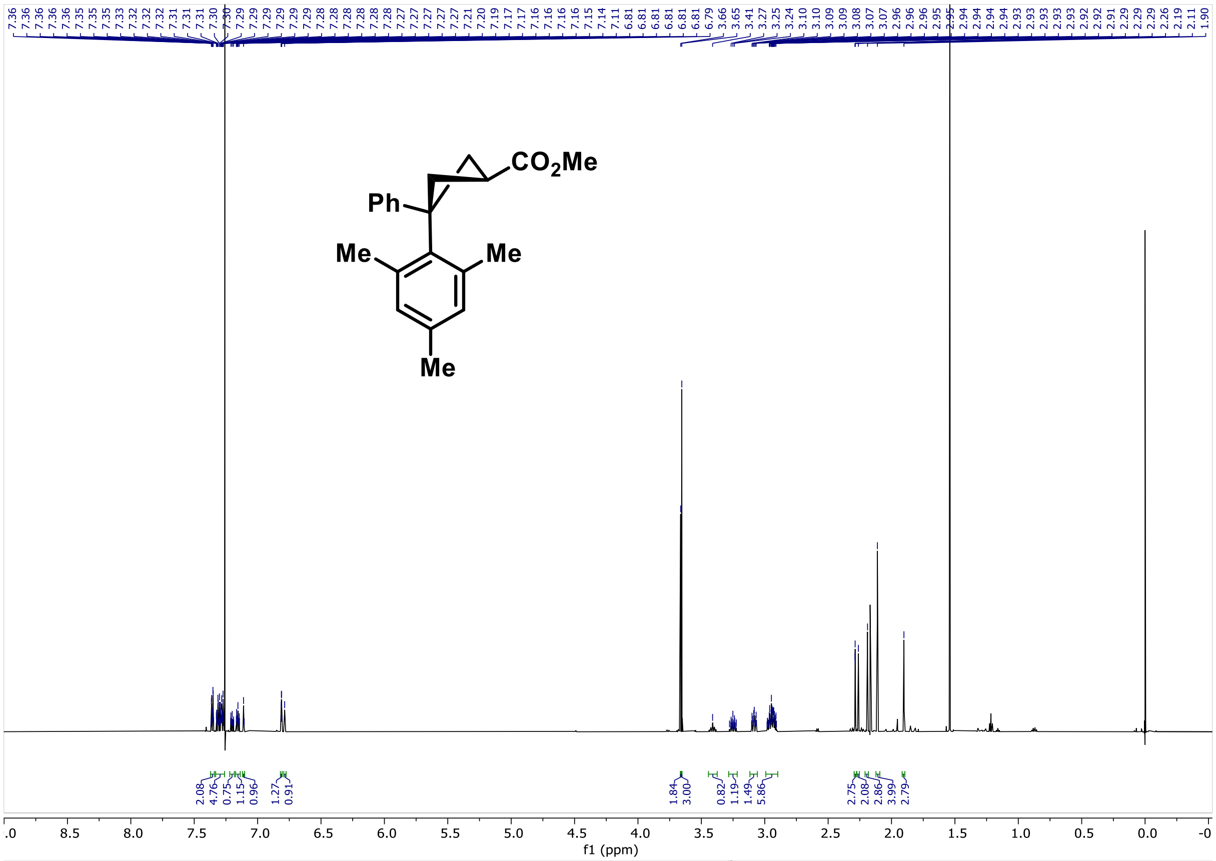

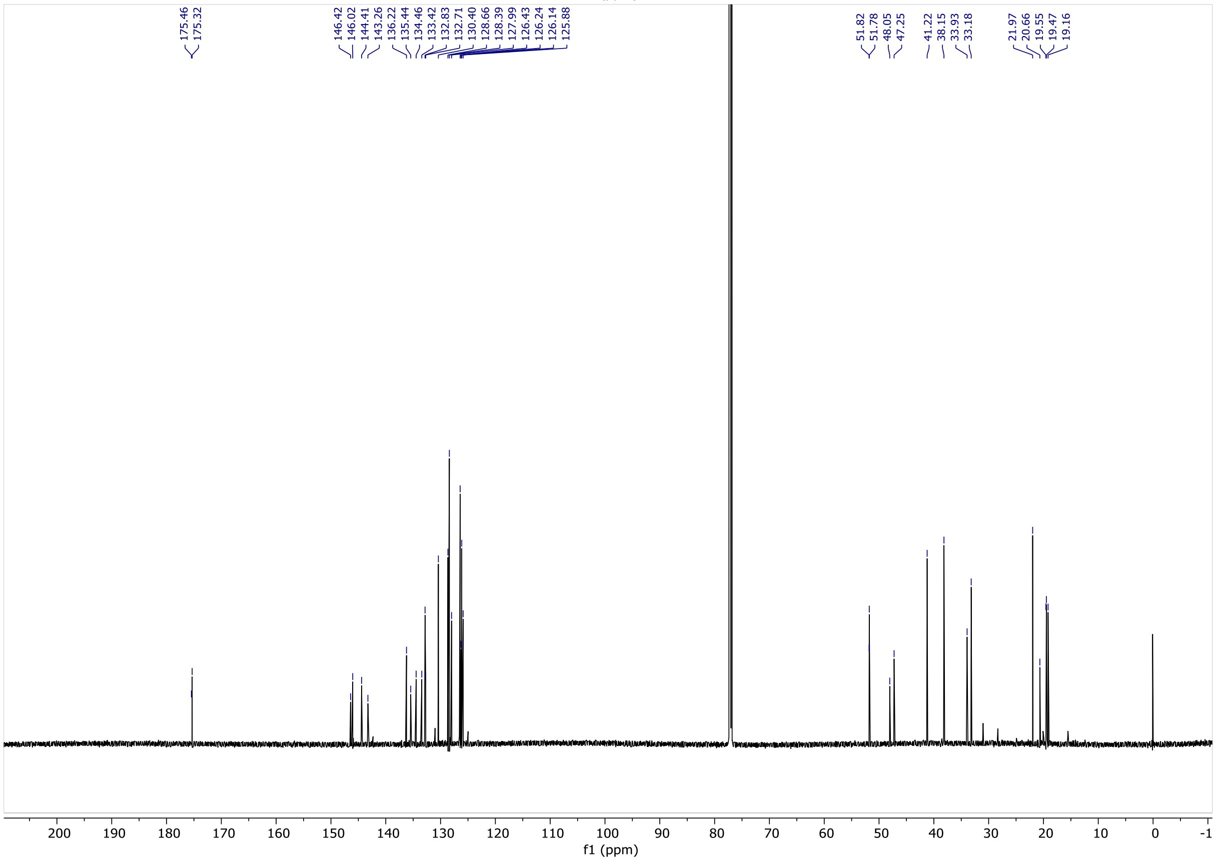

Methyl 3-(benzo[d][1,3]dioxol-5-yl)-3-phenylcyclobutane-1-carboxylate (5t)

[^{1}H-NMR: 500 MHz, ^{13}C-NMR: 126 MHz]

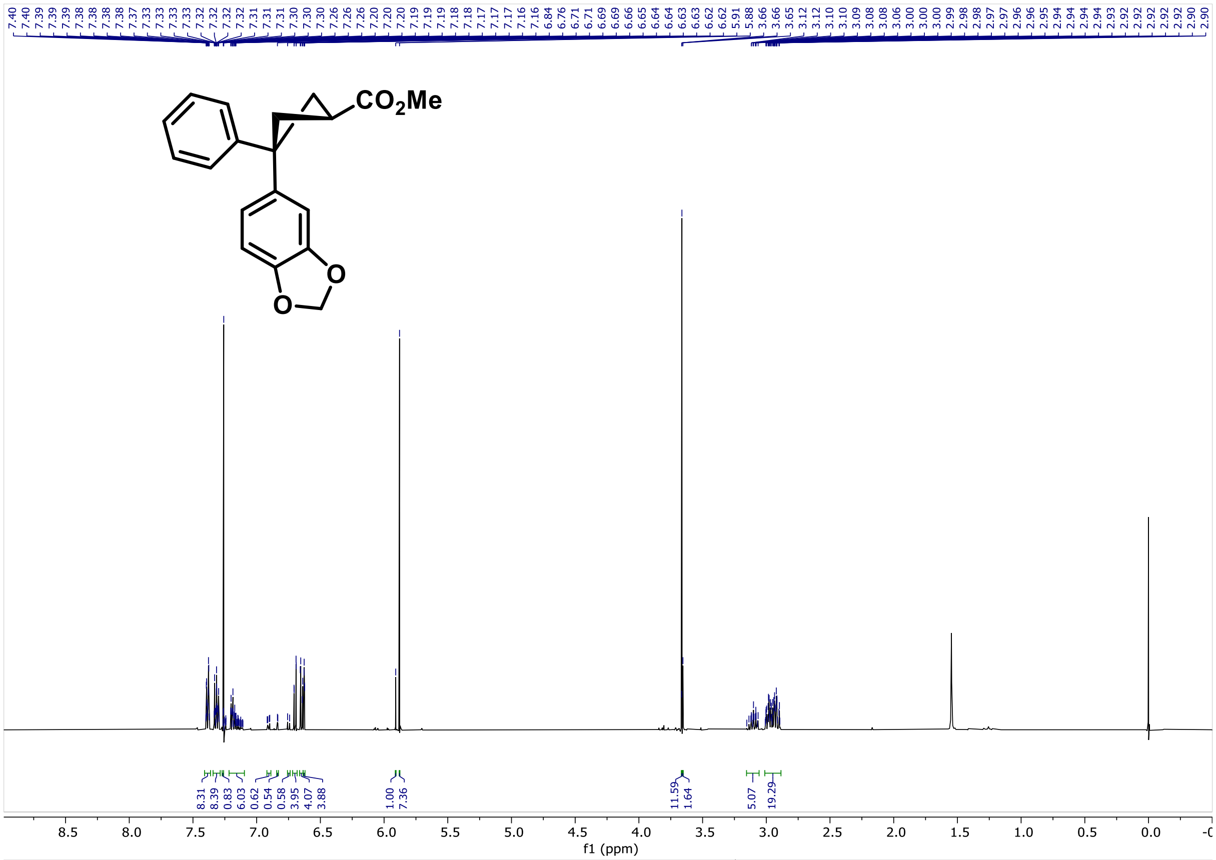

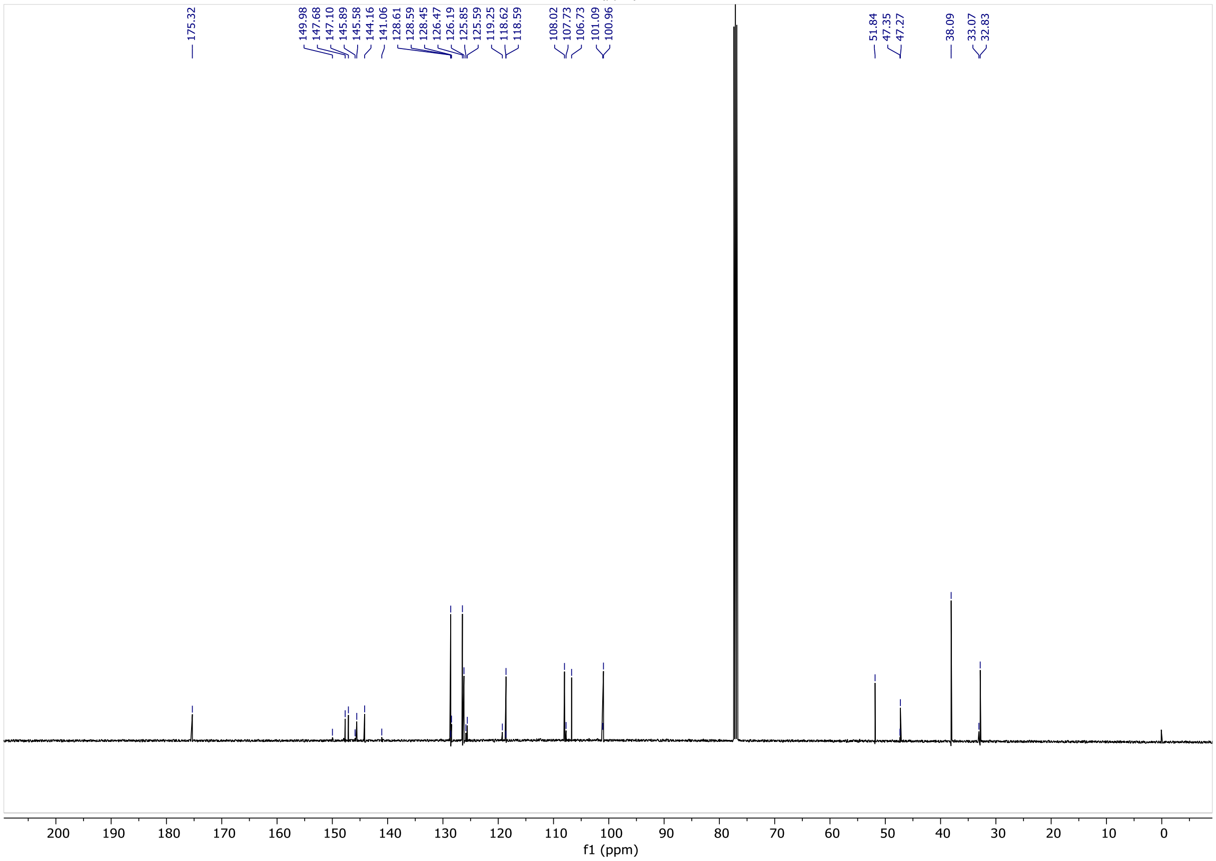

Methyl 3-phenyl-3-(5,6,7,8-tetrahydronaphthalen-2-yl)cyclobutane-1-carboxylate (5u)
[^{1}H-NMR: 700 MHz, ^{13}C-NMR: 176 MHz]

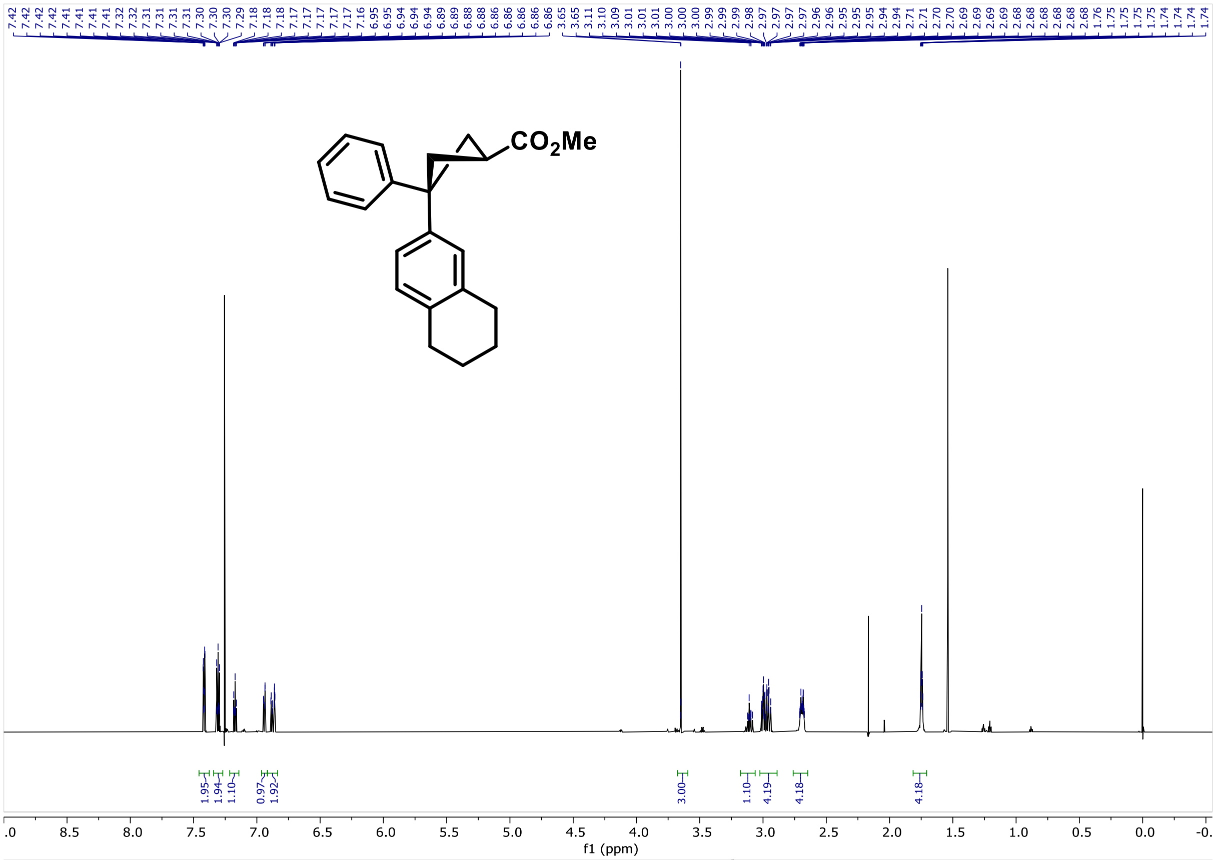

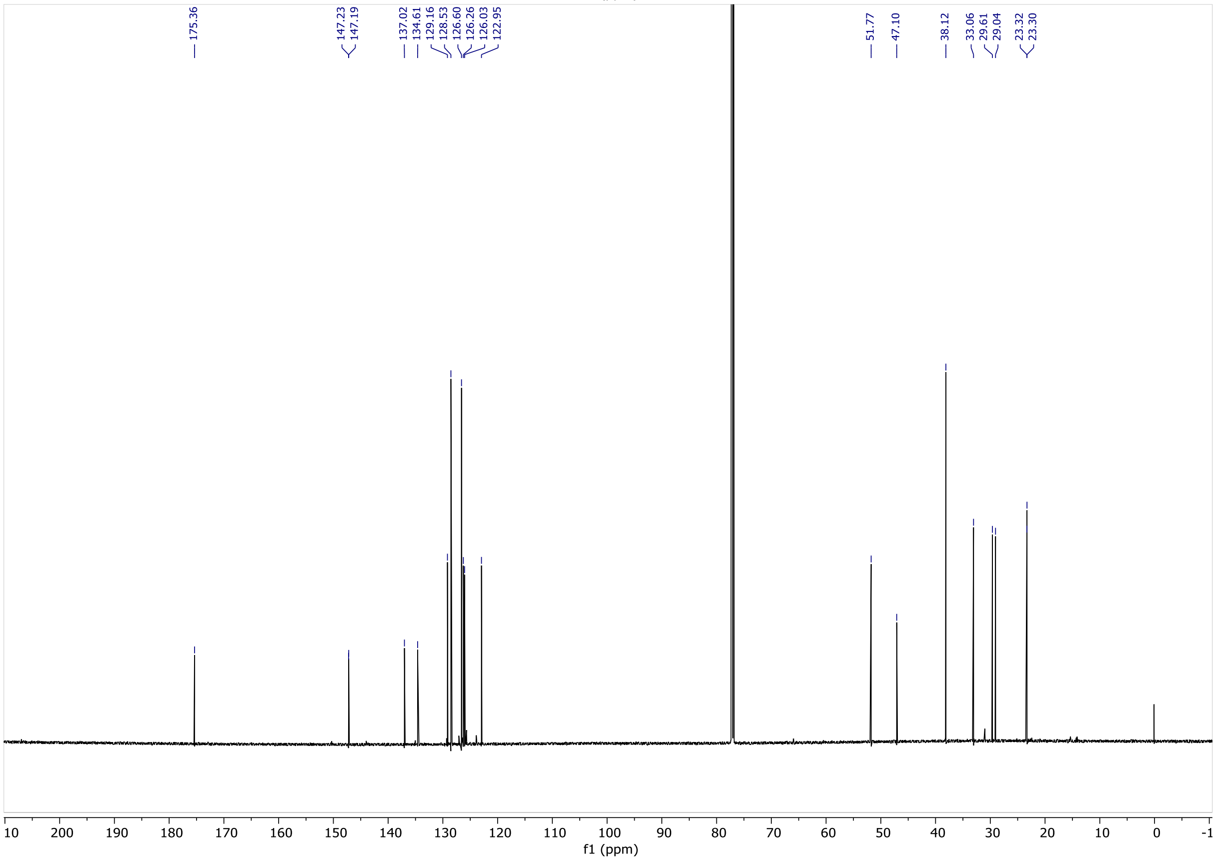

Methyl 3-(naphthalen-2-yl)-3-phenylcyclobutane-1-carboxylate (5v)
[^{1}H-NMR: 700 MHz, ^{13}C-NMR: 176 MHz]

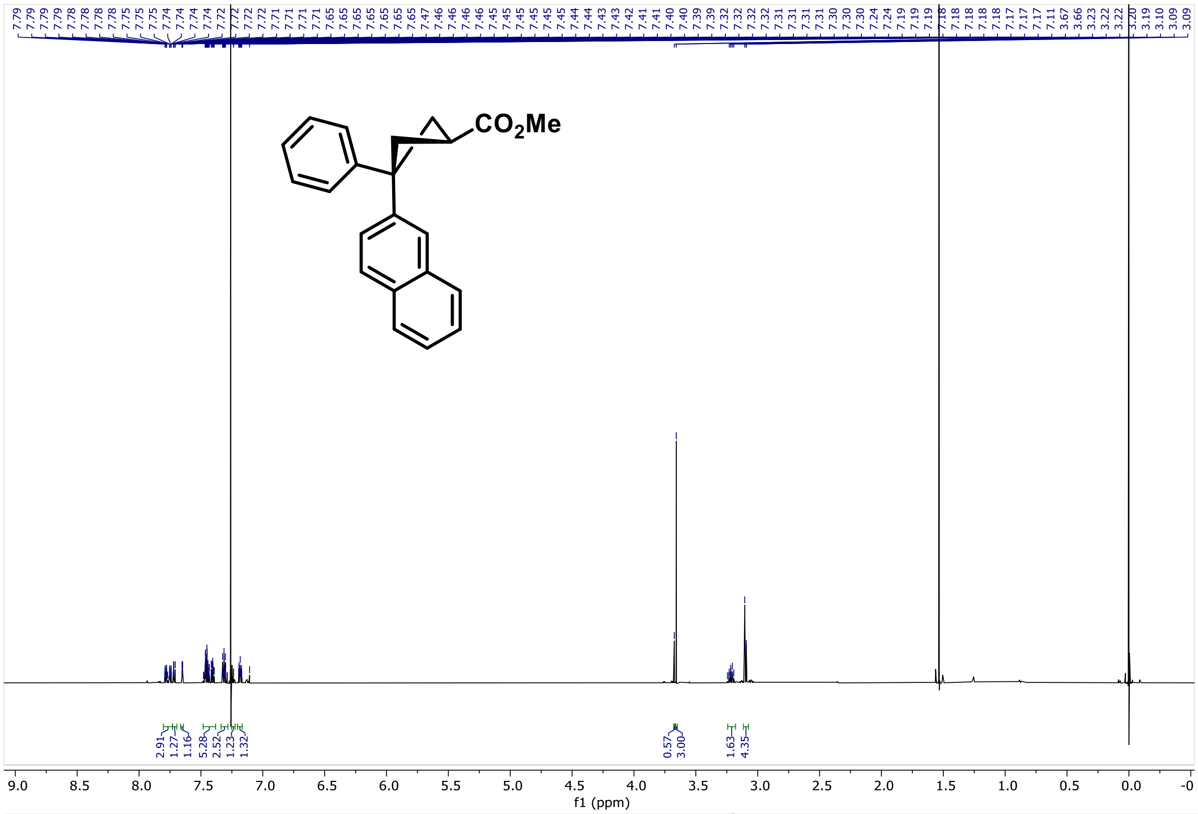

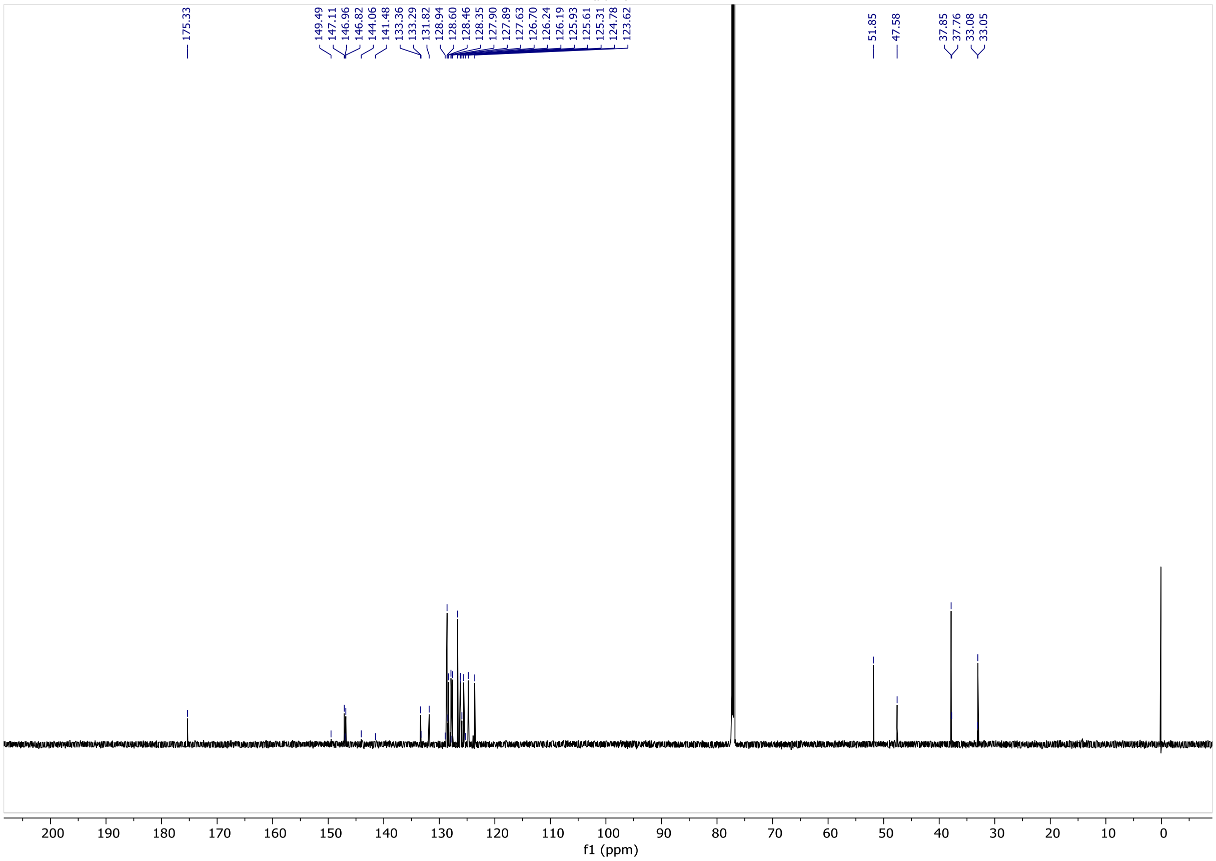

8.

References

(1) Tyler, J. L.; Schäfer, F.; Shao, H.; Stein, C.; Wong, A.; Daniliuc, C. G.; Houk, K. N.; Glorius, F. Bicyclo[1.1.0]butyl Radical Cations: Synthesis and Application to [2π + 2σ] Cycloaddition Reactions. *Journal of the American Chemical Society* **2024**, *146* (23), 16237–16247. DOI: 10.1021/jacs.4c04403.
(2) Golfmann, M.; Reinhold, M.; Steen, J. D.; Deike, M. S.; Rodemann, B.; Golz, C.; Crespi, S.; Walker, J. C. L. Photocatalytic Oxidative Activation of Bicyclo[1.1.0]butanes for Formal [2σ+2π] Cycloadditions. *ACS Catalysis* **2024**, *14* (18), 13987–13998. DOI: 10.1021/acscatal.4c05067.
(3) Tang, S.-Y.; Wang, Z.-J.; Wu, J.-J.; Xing, Z.-X.; Du, Z.-Y.; Huang, H.-M. Photocatalytic synthesis of 2-oxabicyclo[2.1.1]hexanes: cobalt-enhanced efficiency. *Chemical Science* **2025**, *16* (26), 11908–11917. DOI: 10.1039/D5SC02836A.
